# Genome Analysis of *Staphylococcus agnetis*, an Agent of Lameness in Broiler Chickens

**DOI:** 10.1371/journal.pone.0143336

**Published:** 2015-11-25

**Authors:** Adnan A. K. Al-Rubaye, M. Brian Couger, Sohita Ojha, Jeff F. Pummill, Joseph A. Koon, Robert F. Wideman, Douglas D. Rhoads

**Affiliations:** 1 Interdisciplinary Graduate Program in Cell and Molecular Biology, University of Arkansas, Fayetteville, AR, United States of America; 2 Department of Biological Sciences, University of Arkansas, Fayetteville, AR, United States of America; 3 Department of Microbiology, Oklahoma State University, Stillwater, OK, United States of America; 4 Arkansas High Performance Computing Center, University of Arkansas, Fayetteville, AR, United States of America; 5 Department of Biology, Ouachita Baptist University, Arkadelphia, AR, United States of America; 6 Department of Poultry Sciences, University of Arkansas, Fayetteville, AR, United States of America; Wilfrid Laurier University, CANADA

## Abstract

Lameness in broiler chickens is a significant animal welfare and financial issue. Lameness can be enhanced by rearing young broilers on wire flooring. We have identified *Staphylococcus agnetis* as significantly involved in bacterial chondronecrosis with osteomyelitis (BCO) in proximal tibia and femorae, leading to lameness in broiler chickens in the wire floor system. Administration of *S*. *agnetis* in water induces lameness. Previously reported in some cases of cattle mastitis, this is the first report of this poorly described pathogen in chickens. We used long and short read next generation sequencing to assemble single finished contigs for the genome and a large plasmid from the chicken pathogen. Comparison of the *S*. *agnetis* genome to those of other pathogenic Staphylococci shows that *S*.*agnetis* contains a distinct repertoire of virulence determinants. Additionally, the *S*. *agnetis* genome has several regions that differ substantially from the genomes of other pathogenic Staphylococci. Comparison of our finished genome to a recent draft genome for a cattle mastitis isolate suggests that future investigations focus on the evolutionary epidemiology of this emerging pathogen of domestic animals.

## Introduction

Lameness is a significant animal welfare issue resulting in millions of dollars of losses annually for the broiler industry. A model for inducing lameness at high frequency in broilers has been developed using growth on an elevated wire floor [1]. Lameness in this model is predominantly associated with bacterial chondronecrosis with osteomyelitis (BCO) of the proximal tibiae and femora. Different broiler lines have been shown to be susceptible but there may be some line differences and sire-effects [2, 3]. A model for BCO susceptibility based on the vasculature and growth plate dynamics has been described [4, 5]. To investigate this further we have cultured bacteria from lame birds with BCO and used rDNA sequencing to evaluate the species involved in BCO generated in broilers using the wire floor model. Previously, many different opportunistic organisms have been reported from BCO lesions, including *Staphylococcus aureus*, Staphylococcus spp., *Escherichia coli*, and *Enterococcus cecorum*, including mixed infections with Salmonella spp. [6–16]. We now report that for broilers raised on wire-flooring on our research farm, the vast majority of BCO lesions from clinically lame birds yield a single species, *Staphylococcus agnetis*, previously associated with mastitis in cattle [17] and the gut of sheep mites [18]. We also report the sequence, assembly and analysis of the complete genome of this emerging pathogen.

## Materials and Methods

### Ethics Statement

All animal procedures were approved (protocols08036, 11002, and 14005) by the University of Arkansas Institutional Animal Care and Use Committee to ensure compliance with all applicable provisions of the United States of America Animal Welfare Act and other federal statutes and regulations relating to the use of live vertebrate animals in research and teaching. Broilers appear to purposefully avoid exhibiting overt symptoms of lameness in order to avoid being victimized by the predatory behavior of their flock mates. Fast growing and clinically healthy broilers showing no apparent gait abnormalities nevertheless can, within 24 hours, exhibit early symptoms of lameness (hesitancy to stand, eagerness to sit, slight wing-tip dipping). Accordingly, to minimize the birds’ distress, beginning on day 15 and continuing through day 56 all birds were observed walking at least once per day and were humanely euthanized as soon as they exhibited the earliest symptoms of lameness.

### *Gallus gallus* Pathogenicity Model for BCO

Boiler chicks were reared on the wire floor model developed and patented by R. F. Wideman [1, 3]. Overtly lame birds were identified, and blood was collected from wing veins after surface sterilization with 70% ethanol using EDTA-vacutainers. Blood samples (0.1 ml) were directly plated on agar media. Birds were then euthanized by cervical dislocation. To aseptically sample the proximal femora and tibiae the skin was drenched with 70% ethanol. An incision through the dermis covering the inner thigh was made with an ethanol sterilized scalpel and the entire dermal layer was peeled away to expose the leg. The exposed musculature was drenched with 70% ethanol, then incisions were made with an ethanol sterilized scalpel at the joints which were then bent at an acute angle to expose either the articulated surfaces. The proximal femora and tibiae were visually scored for lesion type [2, 3] then sampled with a Sterile Cotton Tip Applicator (Puritan Medical Products, Guilford, MA). Depending on the experiment, the applicator was then used to either inoculate 3 ml of broth or directly rubbed over the surface of agar plates. Media tested for growth included: Brain Heart Infusion Nutrient, BBL Levine Eosin Methylene Blue, Tryptic Soy, BBL Mannitol Salt, and Difco m Staphylococcus (Becton Dickinson, Franklin Lakes, NJ), also Salmonella Shigella, and Selenite (Neogen Acumedia, Lansing MI), and Gelatin Mannitol Salt (Himedia Laboratories, India). Broth inoculums were allowed to grow overnight and then streak plated onto the same medium for individual colonies. Individual colonies were sampled with a sterile toothpick into 50 μl of sterile H_2_O in 200 μl PCR plates, sealed and incubated at 100°C for 15 minutes then cooled to 4°C. These extracts were used to PCR amplify specific regions of the 16S rDNA using universal prokaryote primers Bact-8F (5’-AGAGTTTGATCCTGGCTCAG), paired with either Bact-1391R (5’-GACGGGCGGTGTGTRCA), or Bact-936R (5’- GTGCGGGCCCCCGTCAATTC) adapted from [19]. Sequencing was using the same single primers and performed by the University of Arkansas DNA Resource Center. Sequences were aligned and manually edited using the SeqMan module of the LaserGene software package (DNAStar, Madison, WI). Edited sequences were then identified to species using the Ribosomal Database Project (http://rdp.cme.msu.edu/index.jsp) using Seqmatch (settings were Strain: Both, Source: Isolates, Size: <1200, Quality: Good, Taxonomy: Nomenclatural, KNN matches: 3).

### Administration of bacteria in water

Chicks were obtained as before and reared on wire flooring. Prior to placement the nipple waterers were flushed and scrubbed with dilute bleach, and then rinsed with tap water. On day 7 and 14 the water supply was switched to gravity feed from 20 L carboys containing tap water. Selected carboys were inoculated with ca. 10^7^ cfu/ml of one or more bacterial isolates. The bacterial administration in water was allowed for 2 days, then returned to municipal tap water. Total accumulated lameness due to BCO was evaluated through day 56 [1, 3, 4].

### Culture, Isolation, 16s Analysis of Staphylococcus agnetis

*S*. *agnetis* isolate 908 was from a femoral BCO lesion from a broiler raised on the University of Arkansas Poultry Research Farm. The 16S rDNA gene was PCR amplified and sequenced using the Bact-8F and Bact-1391R primers. Sequence was submitted at the Ribosomal DNA project for best matches and confirmed as *S*. *agnetis*. Cultures were stored at -80°C in 40% glycerol. Mid log phase cultures (40 ml) in Tryptic Soy Broth (Difco, Becton, Dickinson and Company, Franklin Lakes, NJ) were pelleted and resuspended in 0.6 ml TE 10 mM TrisCl pH 7.5, 1 mM EDTA, 0.5% SDS. Glass beads (0.6 g 0.2 mM acid washed and sterilized) and 0.6 ml water saturated phenol (IBI Scientific, Peosta, IA) were added, vortexed, incubated at 65°C for 10 minutes then vortexed for 10 minutes. The slurry was centrifuged at 10,000 x g in a swinging rotor at room temperature. The aqueous phase was successively extracted with 50:48:2 phenol:CHCl_3_:Isoamyl alcohol, then 24:1 CHCl_3_:Isoamyl alcohol. Nucleic acids were ethanol precipitated, digested with RNAse T1 (Life Technologies, Thermo Fisher Scientific, Grand Island, NY), extracted as before, and ethanol precipitated. The quality of the DNA was verified on agarose gels, then submitted for sequencing.

### Genome Sequencing and Assembly

Library construction (Nextera tagmentation) and MiSeq paired end 2x251 sequencing was at the UAMS DNA Sequencing Laboratory. In total we obtained 6,680,349 reads. Pacific Bioscience sequencing (R2C5 chemistry) was at the University of Delaware DNA Sequencing & Genotyping Center. Prior to SMRT cell loading the DNA was size selected (>6 kbp) on a Blue Pippen and sequenced on a single SMRT cell. All sequences were deposited in NCBI under Accession PRJNA246192 and BioSample SAMN02743857.

All Pacific Bioscience reads were initially assembled with the wgs 7.0 (Celera)/pacbiotoCA pipeline. Assembly settings were a minimum overlap of 3,200bp. Contigs from the PacBio assembly were then corrected in a templated alignment with the MiSeq data using the NGen module of LaserGene 11.2 (DNAStar, Madison, WI).

### Phylogenetic Anaylsis of *Staphylococcus agnetis* 908

All 16S rRNA genes of *S*. *agnetis* 908 and 31 16s rRNA genes representing pathogenic and non-pathogenic Staphylococci were aligned using ClustalW as implemented in Mega 6.0. A consensus Neighbour Joining Tree with 2500 bootstrap replications was created based on the alignment. Protein phylogenetic trees were created with the same methods substituting all nucleotide parameters within Mega 6.0 with the default options for protein alignment and tree construction.

### Gene Calling and Genome Annotation Methodology

Protein coding genes in the *S*. *agnetis* 908 genome were identified using the prokaryotic prodigal gene calling algorithm on standard settings [20]. Noncoding RNA was predicted using RNAMMER 1.2 online server [21]. Functional annotation of the protein gene models was achieved using a combination of multiple lines of bioinformatics evidence: BLASTP sequence homology searches against both the NCBI NR and the EMBL Trembl protein databases; putative secreted peptides were predicted using the secretion prediction software SignalP 4.0 [22]; transmembrane proteins were identified using the transmembrane identification program tmHMM [23]; tRNA genes were predicted with ARAGORN tRNA prediction program [24]. All proteins were scanned to identify conserved domains using the hmmscan module of the hmmer 3.1 domain prediction program using PFAM 27.0 as a database [25]. Carbohydrate active enzyme database (CAZy) BLASTP [26] was conducted using the Trembl and NR databases using an e-value cutoff of e-5 or less. Domain identification of the both the carbohydrate active enzyme database (CAZy) and conserved functional domains (PFAM 27.0) were identified using an e-value cuffoff of e-4 or less. CRISPR identifications were using the CRISPRs web server at http://crispr.u-psud.fr [27].

## Results

### Identification of Bacteria Involved in BCO in Broilers

**Fig 1:**
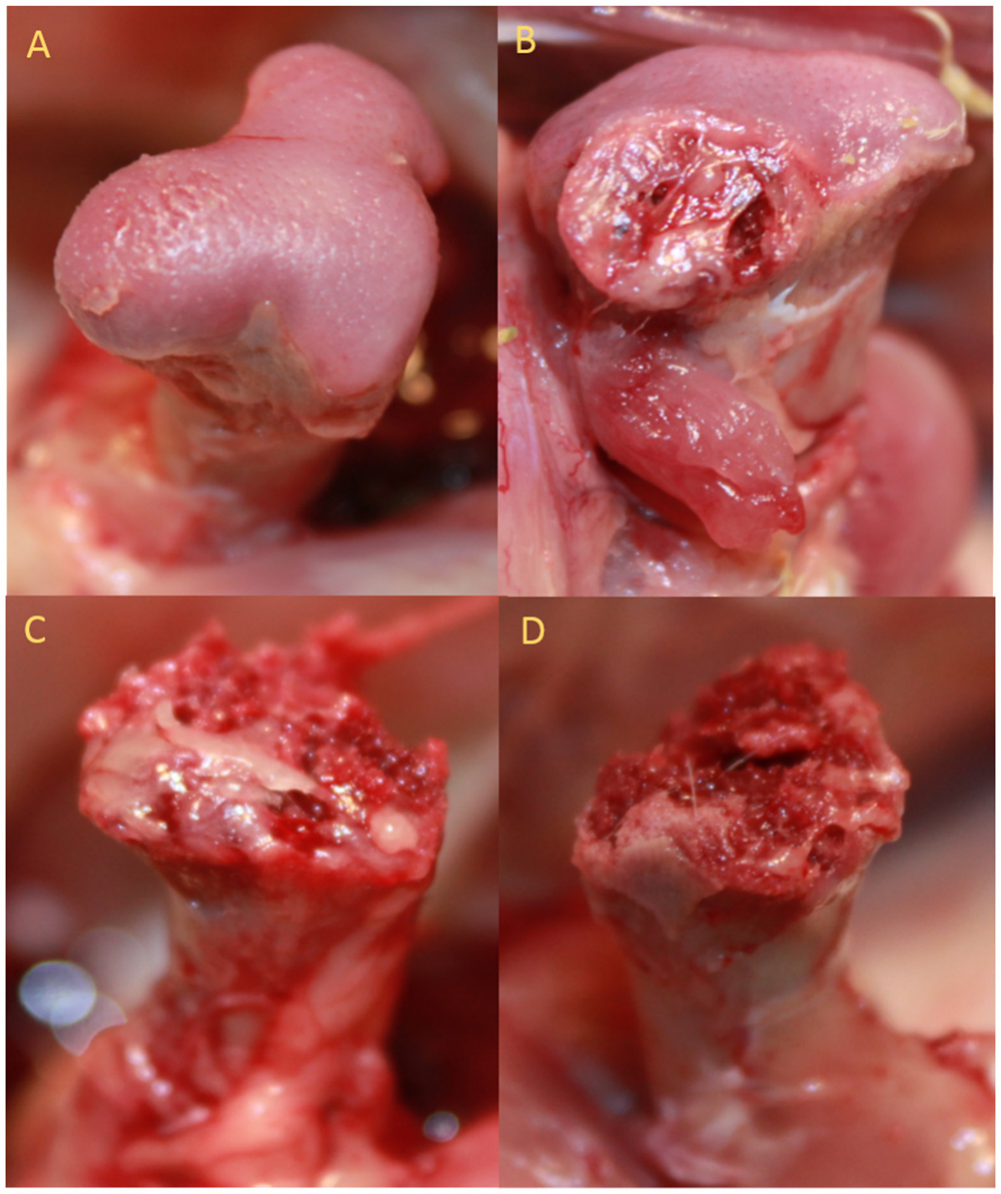
Representative BCO lesions for proximal femora. Severity of the lesions are scored by macroscopic examination: Femoral Head Separation, FHS (Panel A), Femoral Head Transitional degeneration, FHT (Panel B), Femoral Head Necrosis, FHN (Panels C and D) [1].

Commercial broilers were challenged to induce lameness by rearing on suspended wire flooring [1, 2]. Examples of proximal femoral growth plate lesions from lame birds are presented in Fig 1, and were scored for lesion severity as described [1]. We utilized two strategies to identify bacteria from BCO lesions, broth enrichment and direct plating. In initial experiments we used PCR-sequencing on multiple colonies derived from a single lesion. Colony morphologies were uniform and multiple colonies yielded the same species match. In more recent experiments we used sterile swabs to directly sample BCO lesion onto differential media (CHROMagar) to obtain 5 to 50 colonies of consistent color and morphology. We obtained day old chicks from a commercial hatchery, and raised them on wire flooring to induce BCO. For each of 24 birds we sampled the blood and the proximal femoral and tibial growth plates of both legs. Bacterial colonies were screened using a rapid boil method and PCR sequencing using Bact-8F and Bact-1391R primers to amplify and sequence nearly the entire 16S rDNA region. The species identified are presented in Table 1. As indicated we had a significant number of PCR failures or poor sequence reads. We have since modified our strategy to target only the region from Bact-8F through Bact-936R as the 3’ regions of 16S rDNA was not informative for the species spectrum we detected (data not shown). What is apparent from the data in Table 1 is that Staphylococcus is the predominant genus isolated from proximal femora and tibiae of lame broilers with the clear majority being *Staphylococcus agnetis*. In subsequent experiments, when we used media and growth conditions to culture *Enterococcus cecorum* or Campylobacter from BCO lesions we were unable to recover these specific bacteria but could culture *S*. *agnetis*. The analysis of all four bone regions and blood from these birds also demonstrated that for the *S*. *agnetis* positive lesions the species could be isolated from multiple sites in the same bird, which was not true for the other species identified. The same species data was also analysed with respect to the type of lesion. The data in Table 2 indicate that *S*. *agnetis* could be isolated from the spectrum of lesions including macroscopically normal proximal femora and tibiae. It should be noted that in those birds where were able to culture bacteria from apparently healthy femora and tibiae, they were negative for culture of bacteria from blood so the presence of the bacteria in the bone is not from a general sepsis. Previous metagenomics work published by our collaborators, has already shown that DNAs of many bacterial genera can be detected in apparently healthy proximal femorae and tibia [28]. As these experiments utilized chicks obtained from a commercial hatchery we hypothesized that they could have been contaminated with *S*. *agnetis* at hatch by hatchery workers or equipment. We therefore obtained fertile eggs from a different commercial hatchery and hatched the chicks in our university operated hatchery. Chicks were reared on the wire flooring in the same manner and lame birds sampled as described. The spectrum of species was similar and *S*. *agnetis* was the predominant species (data not shown). In analogous experiments we determined that *S*. *agnetis* could be cultured from 75 to 80% of BCO lesions for males vs. females or for four different commercial broiler breeder lines (data not shown).

**Table 1:** Bacterial species from lame birds based on site sampled.

Bacterial Species	Right Femur	Left Femur	Right Tibia	Left Tibia	Blood	Total
*Enterococcus faecalis*	0	0	1	0	0	1
*Staphylococcus agnetis*	13	12	21	20	15	81
*Staphylococcus aureus*	1	1	0	0	1	3
*Staphylococcus epidirmidis*	1	1	0	0	0	2
*Staphylococcus hominis*	1	1	0	1	0	3
*Staphylococcus saprophyticus*	0	1	0	0	0	1
*Staphylococcus xylosus*	1	1	0	0	0	2
Total number of infections diagnosed	17	17	22	21	16	93

A total of 24 lame birds were sampled from all five locations and bacterial colonies diagnosed by PCR-sequencing of a portion of the 16S rDNA. The number of infection sites diagnosed excludes sampled that either did not show bacterial growth, failed in the PCR, or yielded poor sequence data.

**Table 2:** Bacterial species from lame birds based on bone lesion.

Bacteria	Normal	FHS	FHT	FHN	THN	THNs	THNc	Total
*E*. *faecalis*	0	0	0	0	0	0	1	1
*S*. *agnetis*	3	1	12	10	9	16	15	66
*S*. *aureus*	0	0	2	0	0	0	0	2
*S*. *epidirmidis*	0	1	1	0	0	0	0	2
*S*. *hominis*	0	0	1	1	0	1	0	3
*S*. *saprophyticus*	1	0	0	0	0	0	0	1
*S*. *xylosus*	0	0	2	0	0	0	0	2
Total number of infections diagnosed	4	2	18	11	9	17	16	77

Lesion designations were **Normal-** no macroscopic abnormalities of the proximal femur or tibia; **FHS**- proximal femoral head separation; **FHT-**proximal femoral head transitional degeneration; **FHN-**proximal femoral head necrosis; **THN-** mild proximal tibial head necrosis; **THNs**-"severe" THN in which the growth plate was imminently threatened or damaged; and, **THNc**- "caseous" THN in which caseous exudates or bacterial sequestrae were macroscopically evident [1, 29]. Species identification and Total number of infections diagnosed was as for Table 1.

### Inoculation with *S*. *agnetis* induces lameness in broilers

To test Koch’s postulate for the isolation of *S*. *agnetis*, we set up four chambers with 50 chicks per pen to administer bacteria in the water supply for 2 successive days for two treatment dates at 7 and 14 days of age. One pen received no bacteria in the water and had a total BCO-lameness of 5 birds (10%). One pen received an isolate of *Enterococcus faecalis* and had a BCO-lameness incidence of 1 bird (2%). One pen received *S*. *agnetis* (our isolate designated as *S*. *agnetis* 908) with a total BCO-lameness of 20 birds (40%). The final pen received both *E*. *faecalis* and *S*. *agnetis* 908 producing 21 lame birds (42%). It should be noted that lame birds were not identifiable until beginning with day 35 which is 19 days after the final inoculation. Thus, exposure to live *S*. *agnetis* bacteria in the water at an early age can induce BCO lameness. Although the *E*. *faecalis* isolate we used had been obtained from a BCO lesion from the experiment in Table 1, this isolate could not induce lameness, and when simultaneously administered with *S*. *agnetis* did not affect the total incidence of lameness. In a followup experiment, we challenged 40 birds in a similar manner with the human isolate *Staphylococcus aureus* ATCC27661. Total lameness was 20% and cultures from BCO lesions from 6 lame birds yielded only *S*. *agnetis*. Therefore, adding *S*. *agnetis* to the drinking water increases the incidence of lameness and the predominant isolate from BCO lesions is *S*. *agnetis* even when we challenge young chickens with a different Staphylococcus species. However, we have not demonstrated that *S*. *agnetis* is present in the microbial foci associated with BCO [4, 30, 31] and it is highly likely that other bacteria may be present that are not amenable to our culture or sampling conditions [28].

### Genome Sequencing and Assembly

Upon identification of *S*. *agnetis* as strongly associated with BCO-lameness in broilers we inspected the DNA and publication databases and found no information linking this bacterium with poultry, and minimal sequence information. We therefore initiated a project to sequence and assemble the genome. Subsequently a partial genome assembly for an isolate of *S*. *agnetis* from an incidence of mastitis has been deposited [17, 32]. Purified DNA from *S*. *agnetis* 908 was subjected to long read technology sequencing (Pacific Bioscience, Menlo Park, CA). We then used the Celera pacbiotoCA pipeline to assemble initial contigs which were then corrected using 2x251 paired end MiSeq data (Materials and Methodsand Table 3). This produced two contigs devoid of gaps and containing a few ambiguous bases. One contig of 2.4 Mbp representing the main chromosome and one contig of 38 kbp representing a large plasmid. The 2.4 Mbp contig contained 5 rDNA operons. Reasoning that this would be the most likely site for an assembly error due to the size and repetitive nature of the rDNA repeats, we designed PCR primers that flanked all 5 rDNA operons and used the forward and reverse primers in all possible paired combinations. Appropriate sized (app. 5 kbp) products were only obtained with the primer pairs predicted to be compatible from the alignment (data not shown). Therefore, the assembly appeared to be representative of the complete genome and differs from the 6 rDNA operons predicted by Calcutt et al. [32] based on a fragmented (45 contigs) assembly of 454 data from an isolate, CBMRN 20813338, from a case of cow mastitis. Both contigs we assembled included terminal direct repeats that we suspected were circular permutations from the original circular DNAs. The repeats on the 2.4 Mbp contig were 17.2 kbp in length and differed for just 16 bases. Read depths were approximately half for these two regions from the templated MiSeq which would be consistent with these regions representing overlaps for the main chromosome. Inspection of the terminal repeat regions for templated alignment of the MiSeq data with the 2.4 Mbp contig showed that the 5’ terminal 17.2 kbp repeat had 5 short (1–6 bp) regions with no support from the MiSeq reads. For the 3’ terminal repeat there was only one region with low support surrounding a poly T region. We theorized that read errors in the PacBio data led to the terminal repeats, and the MiSeq data could be used to determine which of the terminal repeats was best supported in the MiSeq with greater read depth. We corrected the single region in the 3’ 17.2 kbp repeat with the corresponding region from the 5’ repeat, then deleted the 5’ 17,223 bp repeat. When we used this new 2,474,434 bp contig for a reference based, assembly with the MiSeq data, all regions were fully supported with read depths over 100. Similarly, the 38 kbp contig had 9037 bp terminal direct repeats that differed at 24 positions, but there was no similar indication from the read depth graphs for a circular permutation in the assembly. We suspect this derived from a much higher overall read depth for some regions of the plasmid. At one end of the repeat was an 1800 bp imperfect palindrome (306 mismatches). Total plasmid DNAs were prepared by standard alkaline SDS lysis methods and individually digested with EcoRV, HindIII, EcoRI and BglII, and separated in an agarose gel. Fragment sizes were compared to predicted fragments. The data (not shown) are consistent with circular permutations in the assembly generating the 9 kbp repeats and that this plasmid is most likely 28,965 kbp. All additional analyses were using the two contigs with the terminal repeats corrected and the 5’ repeat removed and these sequences have been deposited in Genbank at NCBI.NLM.NIH.gov as CP009623 and CP009624.

**Table 3:** Next generation sequence data utilized for assembly of the *S*. *agnetis* 908 genome.

Sequence Technology	Parameter	Amount
Pacific Bioscience (R2C5 chemistry)	Number Reads	66332
	n50 Reads	17877
	n90 of Reads	6703
	Number of Bases (Mbp)	231
Illumina MiSeq 2x251 bases	Number of Read Pairs	8,476,030
	Number of Bases (Gbp)	4.23

**Fig 2:**
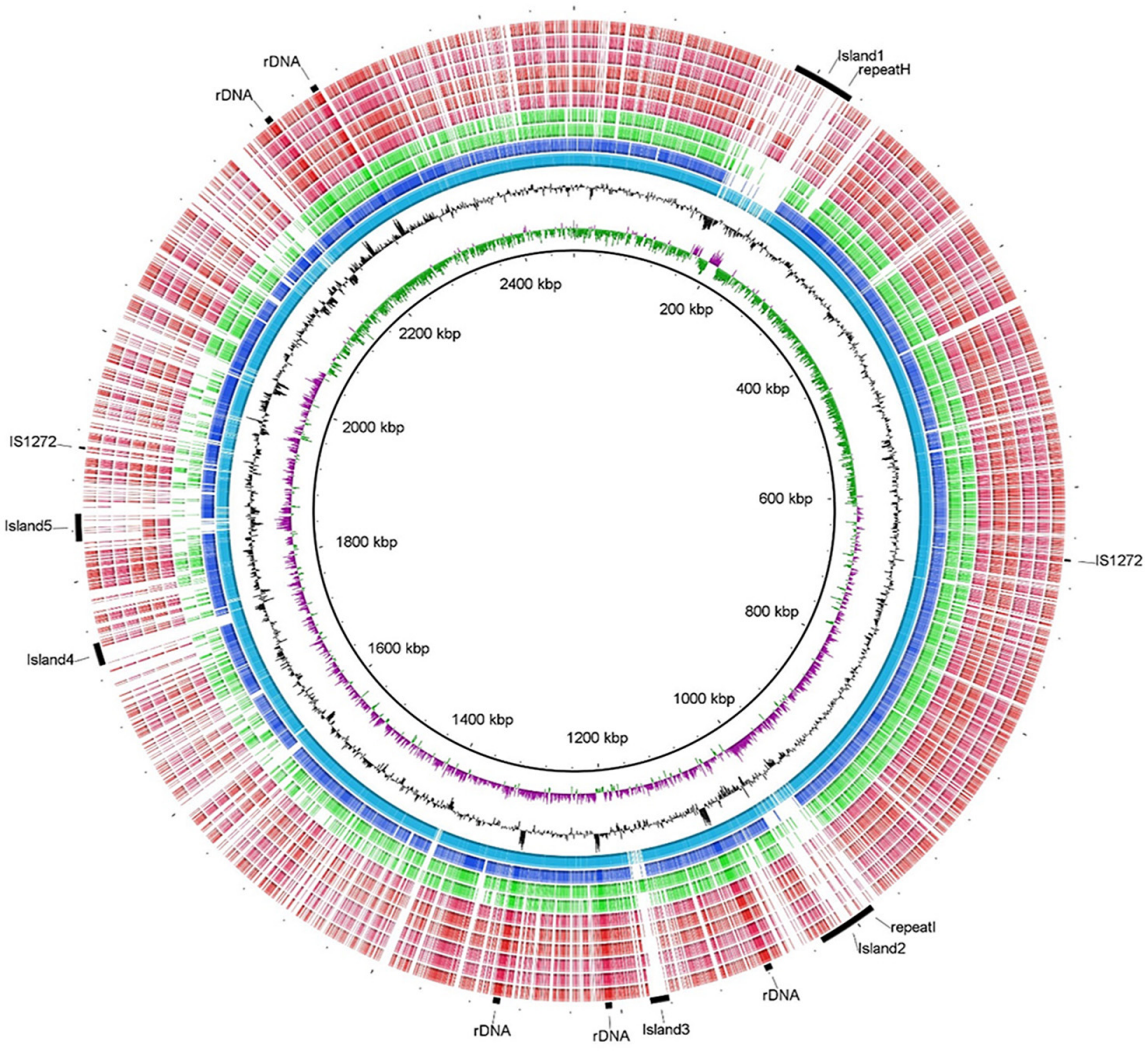
BLAST Ring Image comparing Staphylococcus genomes. BLAST Ring Image Generator version 0.95 [35] was used to analyse the *S*. *agnetis* 908 2.4 Mb contig by position (inner ring 1), GC skew (ring 2), and for GC content (ring 3). BLASTn conservation (minimum threshold 50%) in other Staphylococcus genomes proceed outward *S*. *agnetis* CBRMN20813338, *S*. *hyicus* ATCC11249, *S*. *pseudintermedius* HKU10-03, *S saprophyticus* ATCC15305, and then five *S*. *aureus* genomes for strains FPR3757, TW20, ST228, N315, JH1, and 04–02981. The outer ring indicates features described in the text for the *S*. *agnetis* 908 genome.

The most likely location of the origin of replication in the 2.4 Mbp contig is an intergenic region from 2,057,589 to 2,058,507, that includes a transition in the GC skew (Fig 2), contains three copies of the *DnaA* binding site TTATCCACA, is proximal to DNA Gyrase B (2,050,644 to 2,047,984), and contains highly similar sequence from the *S*. *aureus* JH9 origin [33, 34]. There are three copies of an Insertion Sequence related to the IS1272 family present in many Staphylococcus genomes. One copy is on the 29 kbp plasmid and two copies on the 2.4 Mbp (657,096 and 1,906,928 bp) main chromosome (Fig 2). This repeat sequence is approximately 1800 bases in length with imperfect 48–52 base palindromic repeats on both ends. Contrary to the report of Calcutt et al. [32], we identified a CRISPR system with *Cas2* and *Cas9* encoded homologs (ORF 1815 and 1817) followed by a CRISPR cluster with 33 spacers (S1 Table) BLAST searches of NCBI NR with these 33 spacers indicated 6 of the spacers had compelling matches: two for *S*. *aureus* genome sequences, two for *S*. *aureus* plasmids, and two for bacteriophage EW. In agreement with Calcutt et al. [32], we also identified a cluster of gas vesicle proteins (ORF 1974 and 1977–1985), a hyaluronidase (ORF 2075), and a cluster of five (instead of three) superantigen-like proteins (ORF 1908, 1911, 1913, 1914, and 1915). Our assembly contains an exfoliative toxin A ortholog (ORF 2066) that was not identified in the *S*. *agnetis* CBRMN20813338 draft assembly.

### Phylogenetic classification of *S*. *agnetis* 908

**Fig 3:**
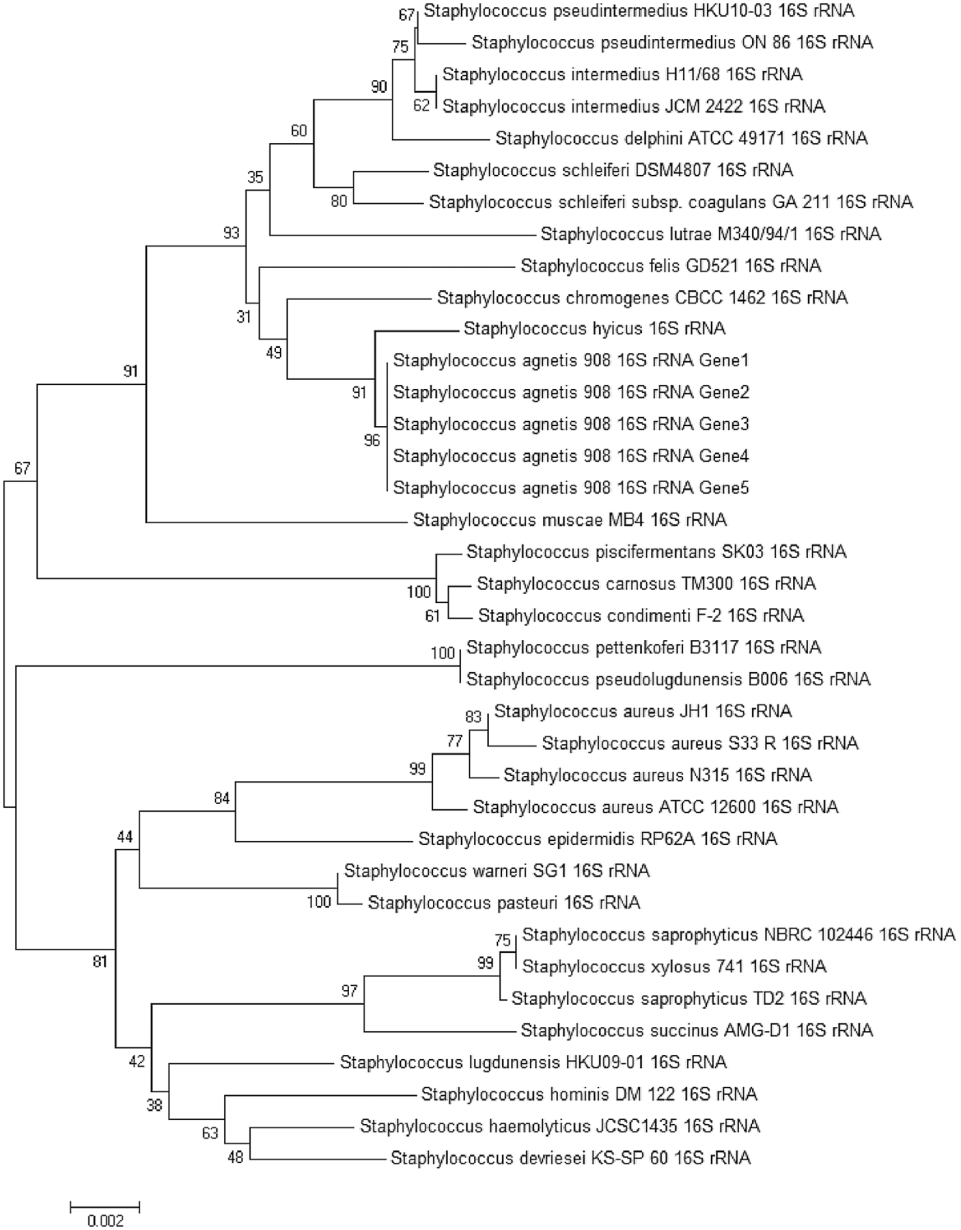
Phylogenetic tree of Staphylococcus species based on 16S rDNA sequences. Full length 16S rDNA sequences for the indicated species and isolates were obtained from NCBI and aligned using ClustalW and used to generate a Neighbor Joining tree (see Materials and Methods).

Initial Ribosomal DNA Project assignment of the amplified 16S region from our cultured isolate indicated the closest match to sequences from *S*. *agnetis* from cattle and sheep mites [17, 18]. In an effort to further classify the relationships of these isolates within the Staphylococci we generated a neighbour joining tree with 2500 bootstrap iterations including all four 16s rRNA genes found in the *S*. *agnetis* 908 genome (Fig 3). The tree demonstrates that *S*. *agnetis* 908 clustered most closely with *Staphylococcus hyicus* and *Staphylococcus chromogenes*. This cluster is distant from that of the well-studied human pathogen *Staphylococcus aureus* and the human commensal *Staphylococcus epidermis*. Species classification using an independent method of digital DNA hybridization with the Genome-to-Genome Distance calculator [36] also supported the classification of S. agnetis as a distinct species of Staphylococcus. This analysis using Formula 2 (sum of all identities found in HSPs divided by overall HSP length) gave distances of 0.2026 and 0.2041 to two *S*. *aureus* genomes (TW20 and ST228), 0.1985 to *S*. *pseudintermedius* HKU10-03, 0.1821 to *S*. *saprophyticus* ATCC15305, 0.1373 to *S*. *hyicus* ATCC11249, and 0.0323 to the *S*. *agnetis* CBMRN20813338 draft assembly. The commercial importance of *S*. *agnetis* and that it clusters with poorly studied species of Staphylococcus with limited genomic information motivated us to conduct further whole genome annotation and genomic comparisons to identify critical features of this pathogens genome.

### Sequence Comparison to Other Staphylococcus Genomes

**Fig 4:**
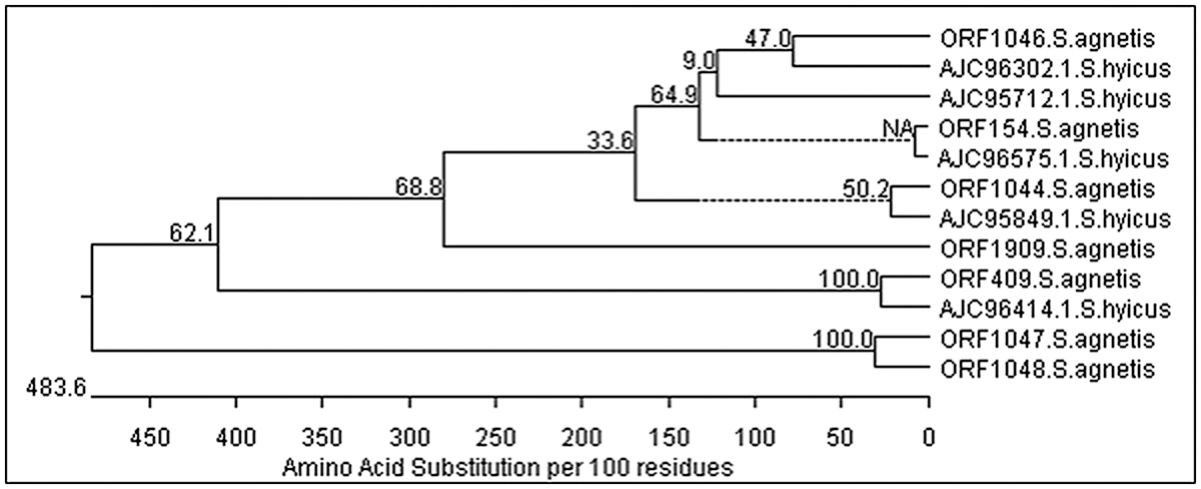
Phylogenetic tree of fibronectin binding proteins from *S*. *agnetis* with homologs from *S*. *hyicus*. The protein sequences predicted for the seven ORFs from *S*. *agnetis* (see text) were aligned using Clustal W to the five predicted fibronectin proteins for the *S*. *hyicus* ATCC11294 genome (AJC entries are accession numbers) representing the closest orthologs in NCBI for the proteins from *S*. *agnetis*. The phylogenetic tree includes bootstrap significance for 2500 iterations.

We performed comparisons of all predicted protein sequences of the *S*. *agnetis* 908 genome to public databases to determine their closest homolog. In agreement with the 16S rDNA phylogenetic tree the majority of predicted coding genes closest homolog (BLASTp of NR with e-value cutoff ≤ e^-5^) were from *S*. *hyicus*. (2106 of 2418; 87.1%). While 5.1% of predicted proteins had a closest homolog in another species (123 of 2148), and 8.5% had no identifiable homolog present in the NR protein database at NCBI (183 of 2148) (S1 Table). Predicted genes with closest homologs not from *S*. *hyicus* were predominantly with Bacillus species (41/123) or other Staphylococci (21/123). Notable genes with more significant matches with *S*. *aureus* than *S*. *hyicus* include 2 collagen adhesion proteins (ORF 446 and 675) involved in pathogenesis, 3 autolysins (ORF 248, 960, 1399) involved in cell wall maintenance and a beta-lactamase (ORF 165). There are also an autolysin (ORF 263) and three beta-lactamases (ORF 717, 1761, 1960) most closely related to homologs in *S*. *hyicus*. Genes showing significant divergence from their ortholog in *S*. *hyicus* include staphostatin A involved in the neutralization of proteases secreted by other Staphylococci, the pathogen associated genes fibronectin binding protein, peptidase T and the immune evasion protein capsular polysaccharide biosynthesis genes. For staphostatin A (ORF 2396) the predicted 106 residue polypeptide is only 54% identical with *S*. *hyicus* and 48% with *S*. *aureus*. The *S*. *agnetis* genome encodes seven predicted fibronectin binding proteins including a cluster of four (ORF 1044, 1046, 1047, 1048) where *S*. *hyicus* ATCC11249 encodes only five, with no clusters. In *S*. *agnetis* the predicted sizes of the fibronectin proteins for ORF 154, 409, 1044, 1046, 1047, 1048, and 1909, are 835, 571, 742, 577, 253, 104, and 698 amino acids, respectively. *S*. *hyicus* encodes fibronectin binding proteins of 82, 138, 290, 371 and 498 amino acids. Phylogenetic tree construction (Fig 4) shows that the fibronectin binding proteins predicted for ORFs 154, 409, 1044 and 1046, are much more similar to orthologs in *S*. *hyicus* than those encoded by ORFs 1047, 1048, and 1909, yet the best match in the NCBI NR protein database is still from *S*. *hyicus* for these three fibronectin binding proteins. Further, despite there being four fibronectin binding genes clustered in the genome they are quite distinct for sequence and size. Therefore, the cluster of fibronectin binding proteins in *S*. *agnetis* is not the result of tandem duplication and diversification but rather appears to be the result of acquisition of distinct fibronectin binding genes since *S*. *agnetis* and *S*. *hyicus* diverged.

When we analysed the location of the coding sequence for the orthologs with best matches not in *S*. *hyicus* (S1 Table) we identified five “islands” of divergence from *S*. *hyicus*. Island 1 (165,731 to 232,722 bp), Island 2 (978,688 to 1,020,928 bp) and Island 3 (1,161,264 to 1,176,428 bp) all contains orthologs to phage genes from a variety of Gram positive and negative bacterial genera. Island 4 (1,731,258 to 1,744,866 bp) contains orthologs to Bacillus proteins for metabolism of glucuronate. Island 5 (1,831,569 to 1,847,837 bp) contains orthologs to *Ydd/Ydc* membrane complexes from Bacillus.

We next excluded the *S*. hyicus genome to identify the next closest homolog for all predicted proteins for *S*. *agnetis*. Homologs related to the canine pathogen *S*. *pseudintermedius* were the most common species found 853/2418) followed by the human commensal skin Staphylococcus member HGB0015 GN (732/2418), with the third most common being the human pathogen *Staphylococcus aureus* (220/2418). These three species along with three others, *S*. *epidermidis*, *Staphylococcus simulans*, *Staphylococcus massiliensis*, made up the majority of homologs for all predicted proteins (1925/2418 79.6%).

Fig 1 graphically depicts sequence conservation between the *S*. *agnetis* 908 2.4 Mb contig with selected other Staphylococcus genome assemblies, including locations of selected genome features GC skew, and GC content. The comparison demonstrates the high conservation relative to the draft mastitis *S*. *agnetis* genome for CBMRN20813338 [32], and the genome of *S*. *hyicus*. The previously described “islands” of encoded proteins that have best orthologs not in *S*. *hyicus* are also apparent. Island 1 contains sequences related to two of the 6 *S*. *aureus* genomes. Interestingly, Island 5 also shows strong sequence conservation with those same two genomes. Island 3 appears to be less similar to the other *S*. *agnetis* assembly, and more similar to a region in *S*. *pseudintermedius*. Interestingly, there are portions of Island 1, 2 and 4 that are not related to any of the other Staphylococci.

### *S*. *agnetis* Virulence Complement is Composed of Homologs for Both Pathogenic and Non-Pathogenic Staphylococci

We next sought to identify what genes in *S*. *agnetis* might mediate infection and sepsis in *Gallus gallus domesticus*. Putative virulence mediators were identified by comparison of predicted *S*. *agnetis* proteins to the Virulence Factor Database (VFDB, [37]). This identified 46 virulence factors belonging to the following classes: 17 for Host Immune Evasion, eleven for host adherence, seven for toxin biosynthesis, and five secretion systems (S2 Table). Of particular interest is the presence in the *S*. *agnetis* genome of genes known to be master regulators of virulence functions in *S*. *aureus* including: staphylococcal accessory regulator family protein (ORF531), an accessory gene regulator BDCA operon (ORF1179-1182), staphylococcal accessory transcriptional regulator family protein (ORF2278), and MgrA (ORF2344). These genes combine to regulate a suite of virulence factors *in vitro* and *in vivo* for *S*. *aureus* [38, 39].

### Toxin Production in *S*. *agnetis* Involves Homologs Closely Related to Other Pathogenic Staphylococci

**Fig 5:**
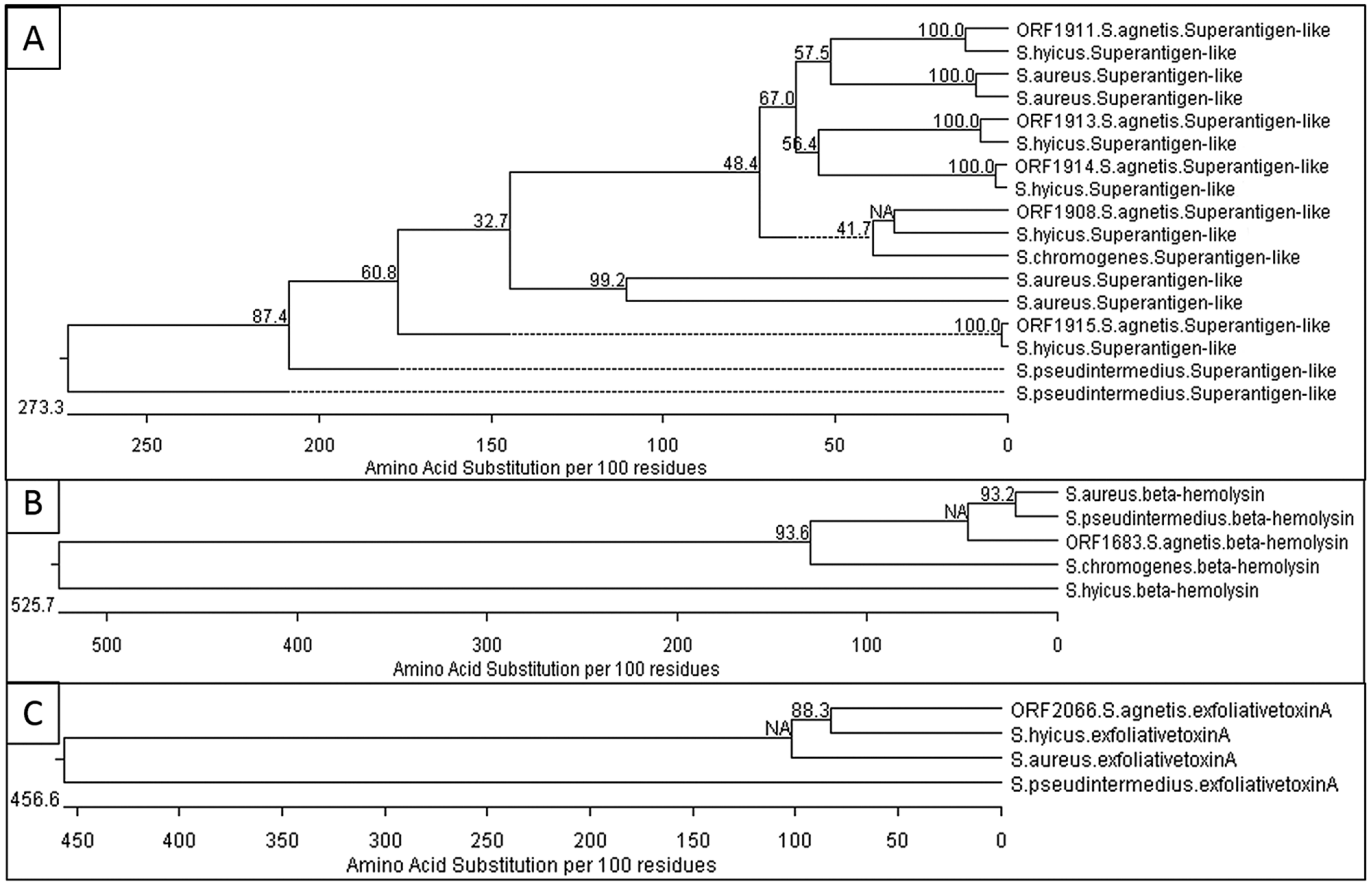
Phylogenetic trees of toxin virulence factors. Trees were constructed using the closest homologs to *S*. *agnetis* toxin proteins from BLASTp searches of refseq proteins from *S*. *hyicus*, S.chromgenes, *S*. *pseudintermedius* and *S*. *aureus*. Tress are for Superantigen-like protein homologs (Panel A), beta hemolysin (Panel B), and exfoliative toxin A (Panel C). The *S*. *agnetis* ortholog are prefixed with the ORF number from S1 Table. Details of the alignment strategy are in Materials and Methods.

We identified seven predicted virulence factors associated with toxin production (S2 Table) that shared high protein sequence conservation to well documented pathogenic Staphylococcus species [8, 13, 40, 41]. We compared these exotoxin polypeptide sequences to *S*. *agnetis* closest relative, *S*. *hyicus* (Fig 3), and three well characterized Staphylococcal pathogens: the human pathogen *S*. *aureus*, *S*. *chromogenes* associated with bovine mastitis, and the canine dermatitis pathogen *S*. *pseudintermedius* [38, 40, 42–44]. Protein based phylogenetic tree characterization suggests that *S*. *agnetis* exotoxins are a mixture with varying relatedness with the other pathogens. The five Superantigen-like homologs appear to cluster most closely with one each of the five Superantigen-like orthologs from *S*. *hyicus* with some of these being more similar to those from *S aureus* than to *S*. *pseudintermedius* (Fig 5A) despite the phylogenetic relationship we see based on 16S sequences. The beta hemolysin in *S*. *agnetis* clusters with homologs from *S*. *aureus*, *S*. *pseudintermedius* and *S*. *chromogenes*, while the ortholog from *S*. *hyicus* is quite distinct (Fig 5B). There was no significant BLASTp match for exfoliative toxin A in *S*. *chromogenes* but the *S*. *agnetis* homolog again clustered with those from *S*. *hyicus* and *S*. *aureus*, and distinct from *S*. *pseudintermedius*. (Fig 5C).

### Infectious Sepsis by *S*. *agnetis* is by a System Distinct from that of *S*. *aureus*

Along with the development of BCO-lameness in broiler chicken we have detected a bacteremia of *S*. *agnetis* in lame birds (Table 1, and unpublished data). *S*. *aureus* sepsis has been well studied previously [42] with three major branches of molecular events necessary for a sepsis infection. The first branch is the initiation of inflammation in host systems to begin infection, the second is eluding the innate immune system to evade immune clearance, the third being the modulation of vascular architecture. We used BLASTp searches of the predicted proteins from the prodigal analysis of the *S*. *agnetis* genome assembly with queries for *S*. *aureus* proteins that mediate early infectious sepsis through targeting immune cells and vascular endothelium [42]. We identified *S*. *agnetis* orthologs for: Staphylococcal protein A (ORF 1941; 84/152 positive residues), coagulase (ORF 2084; 136/270 positive residues), von-Willebrand factor binding protein (ORF 2084; 249/431 positive residues), clumping factor protein A (ORF 1054; 175/323 positive residues), clumping factor B (ORF 1912; 176/314 positive residues), fibronectin-binding protein A (ORF 154; 439/837 positive residues), staphylococcal complement inhibitor (ORF 370; 70/112 positive residues), and phenol soluble modulin (ORF 375; 25/43 positive residues). We did not identify significant matches for: Leukotoxin components E/D, alpha toxin, Panton-Valentine leukocidin S/F, α-hemolysin, γ-hemolysin, or chemotaxis-inhibiting protein. Therefore, either subversion of the chicken immune system by these virulence factors is not necessary for *S*. *agnetis* sepsis, or the chicken targets have diverged with concomitant divergence of the *S*. *agnetis* virulence factors. Sequence comparison of target receptors for some of these missing sepsis proteins between human and *G*. *gallus* showed relatively high conservation (Percent Positive Residues: CXCR1 76.3%, CXCR2 76.5%, C5aR 57.6%) which would argue against the latter possibility. Future expression and gene deletion studies will be required to elucidate the differences in the strategies for inducing sepsis between *S*. *agnetis* and *S*. *aureus*.

### Plasmid Sequence Analyses

As indicated above we conclude that the 29 kbp contig represents a plasmid. Gel analysis of total DNA from multiple isolates of *S*. agnetis showed that all contain additional smaller DNAs that appear to be one or more plasmids in the range of 2.5–6 kbp that we are still investigating (data not shown). BLASTn searches at NCBI did not identify highly similar plasmid DNAs related to our 29 kbp contig in any other Staphylococcus species. Further, MAUVE and Muscle alignments with the 45 contigs from the cattle mastitis isolate, *S*.*agnetis* CBMRN20813338, only detected partial alignments (400–800 bases) with regions in four of the 45 contigs (data not shown), so it is not clear whether the cattle isolate also contains this plasmid. Analysis of the 40 open reading frames on the 29 kbp plasmid revealed that 22 ORFs encoded orthologs of hypothetical polypeptides, with 16 most similar to those from *S*. *aureus* (S3 Table). The plasmid encodes homologs to a replication protein (ORF2), 4 integrases (ORF 3, 23, 37, 40), 4 transposases (ORF 6, 16, 25, 39), and a transposon resolvase (ORF 10). There were also homologs to a *PemK* endoribonuclease growth inhibitor (ORF14), prevent-host-death protein (ORF 8), a lysophosopholipase (ORF 20), and a cobalamin biosynthesis protein (*CobQ*; ORF 27), possibly for scavenging vitamin B12.

The 29 kbp plasmid and the 2.4 Mbp main chromosome differ in overall GC content, 28.6 and 35.8%, respectively (Table 4). We found no GC skew in the plasmid (data not shown) and our preliminary sequence data for two additional small episomal DNAs indicate that these smaller, probable plasmids are also approximately 30% GC (data not shown). For comparison, the GC content of the S. aureus ATCC25923 main chromosome is 32.9% and its 27.5 kbp plasmid is 30.7% [45]. Why *S*. *agnetis* 908 has a greater disparity in GC content between the main chromosome and its episomes is not clear. The GC content of the 29 kbp plasmid is uniform which argues against the GC content difference resulting from any transposable elements or temperate phage lowering the 29 kbp plasmid’s overall GC content. This would be consistent with these plasmids being relatively recent acquisitions (see discussion).

**Table 4:** Summary data of assembly and annotation of the genome of *S*. *agnetis* 908.

Genome Attribute	Value
Number of Chromosomal Contigs	1
Main Chromosome Size	2,474,434
GC Content	35.77%
Ambiguous Nucleotides	2
Gaps	0
Number Protein Coding Genes	2,404
Number of 5S Genes	6
Number of 16S Genes	5
Number of 23S Genes	5
Number of tRNA Genes	59
Number of Plasmid Contigs	1
Plasmid Size	28,965
Plasmid GC Content	28.56%
Number of Protein Coding Genes on Plasmid	40

### Analysis of Genetic Diversity of *S*. *agnetis*

To address the genetic diversity of *S*. *agnetis* isolates infecting the chickens in our experiments we sequenced (5.4 million reads 2x251 MiSeq paired end) for an additional isolate of *S*. *agnetis* from a different bird and then generated an additional 770 thousand MiSeq reads from a pool of 8 additional *S*. *agnetis* isolates. We aligned these to the 2.4 Mbp main chromosome and the 29 kbp plasmid to identify SNPs in the bacterial population at our research farm. For the 2.4 Mbp contig we detected only one A/C SNP supported by a read depth of 496 and a possible InDel that had weak support with a read depth of only 27. Conversely, there were more than 20 variant positions in the 29 kbp plasmid. Even within the MiSeq data for *S*. *agnetis* 908 there were 40 positions that showed probable SNPs for the 29 kbp plasmid, suggesting that this plasmid is hypervariable within the population but that the main chromosome shows little or no variation.

## Discussion

The Staphylococcus genus contains species that have been associated with particular infections in a wide array of vertebrates. Within the Firmicutes the genus Staphylococcus contains a large number of pathogens, as well as many commensal and saprophytic species. Prior to this work, there had been no reports of infections of poultry by *S*. *agnetis*. Previously *S*. *agnetis* had only been identified as an occasional pathogen in mastitis in cattle [17]. Molecular typing had also identified highly similar 16S rDNA sequences in the guts of sheep scab mites [18]. Based on 16S and genome comparisons, *S*. *agnetis* appears to be most closely related to *S*. *hyicus* which is a major agent of exudative dermatitis in swine [40]. However, our assembly differs from that of *S*. *hyicus* for at least five significant regions (Islands 1 through 5) and appears to differ from the draft assembly of the cattle isolate of *S*. *agnetis* for Island 3 (Fig 2). We also do not know if the plasmids we have identified in our *S*. *agnetis* isolates from chicken BCO are also found in cattle mastitis isolates of *S*. *agnetis*. A survey of plasmids in cattle and poultry isolates of *S*. *agnetis* would determine whether pathogenesis in a particular species is facilitated by different plasmids. Owing to high density mixed farming, the complex of the highly related *S*. *hyicus* and *S*. *agnetis* species infecting farmers, cattle, pigs and chickens [8, 17, 18, 41] may have resulted from mild zoonoses that now manifest as more significant pathogens in some of these animals. Since there is evidence from 16S rDNA sequencing that a closely related bacterium is present in sheep cab mites [18], it is possible the bacterium is transmitted by mites or other vectors between cattle and poultry. It will be important to determine whether the *S*. *agnetis* isolates from cattle mastitis have the same pathogenicity in broilers as the isolates we have identified. It will also be critical to examine bacterial species distributions in BCO in other flocks and farms. We have only investigated bacterial species in BCO in broilers at the University of Arkansas poultry research farm which is in close proximity to sheep and cattle.

Wideman et al. [4] have developed a model for BCO where the rapid elongation of the leg bones and blood supply to the growth plate of young broilers should be highly susceptible to colonization by opportunistic bacteria circulating in the blood, thus leading to BCO. Treatment with particular formulations of dietary probiotics can reduce incidence of BCO induced using the wire-flooring model [1, 46]. Therefore, induction of lameness through administration of *S*. *agnetis* in the water is most consistent with bacterial translocation from the gut into the blood as proposed [1, 4, 46]. Recent metagenomic analyses have shown that DNA from a number of different bacterial genera can be detected in apparently healthy proximal femorae and tibia, as well as those diagnosed with BCO [28]. That we predominantly culture *S*. *agnetis* suggests that although many bacterial species may reach the growth plate, this particular species may be best adapted to survive there, multiply there, and induce necrosis. This would likely result from the particular adhesins, and virulence determinants that distinguish *S*. *agnetis* from the other species in the community, and that there may be contributions of other bacterial species that could not be cultured using the methods we employed here.

Although we showed we could induce lameness by administration of *S*. *agnetis* in the water we do not know the actual reservoir for the “natural” bacterial infection. This could be from biofilms in or on the nipple waterers, insect vectored transmission, bird to bird contact, or even vertical transmission through the egg. The reference assembly, functional annotation, and analysis of the *S*. *agnetis* main chromosome and a large plasmid (Table 4) has allowed the initial characterization of virulence factor genes composed of many orthologs to both pathogenic and commensal members of the Staphylococci. Isolation and characterization of this pathogenic Staphylococcus species is critical to understanding the likely route of transmission to broilers, and development of measures for mitigating BCO losses in poultry. This comparison *of S*. *agnetis* with other pathogenic Staphyloccci, including *S aureus*, has contributed to a better overall understanding of the genus Staphylococcus including pathogenicity strategies, conserved and unique enzymes of pathogenesis, and features of the commensal/pathogen bacterial host relationships.

## Supporting Information

S1 Table Significant BLASTp hits of translation products from the *S*. *agnetis* 908 2.4 Mbp contig. ORF indicates the sequential number from the Prodigal analyses, Start and End indicates the starting position (base) in the 2.4 Mbp contig, Len is the length of the predicted translation product, Scr is the score for the Prodigal ORF predictionand Str is the encoding strand. GI/SP indicates either the GI entry for protein record for the most significant match from S. hyicus, or the SwissProt entry where the most significant match was other than *S*. *hyicus*. Significance was defined as an HSP e-value ≤ 10^−5^.
(DOCX)

S2 Table Virulence determinants based on BLASTp matches in the Virulence Factor Database (VFDB) or Tremble database. ORF is the Open Reading Frame as indicated in S1 Table. For each ORF are indicated the most significant match in VFDB the Virulence Category, Gene Index (GI), Description, and Top Hit Species (and Strain), E-value of the match in VFDB, Description of best match for species that is NOT *S*. *hyicus* in Tremble database, with description and E-value.
(DOCX)

S3 Table Significant BLASTp hits of translation products from the *S*. *agnetis* 908 29 kbp plasmid. Details are as for S1 Table.
(DOCX)
