# Supplementary material for: Genome Analysis of Staphylococcus agnetis, an Agent of Lameness in Broiler Chickens
Source: PLoS One. 2015 Nov 25;10(11):e0143336. doi: 10.1371/journal.pone.0143336 (PMC4659636; doi:10.1371/journal.pone.0143336)
Supplement: S1 Table — ORF indicates the sequential number from the Prodigal analyses, Start and End indicates the starting position (base) in the 2.4 Mbp contig, Len is the length of the predicted translation product, Scr is the score for the Prodigal ORF predictionand Str is the encoding strand. GI/SP indicates either the GI entry for protein record for the most significant match from S. hyicus, or the SwissProt entry where the most significant match was other than S. hyicus. Significance was defined as an HSP e-value ≤ 10−5. (DOCX) [file pone.0143336.s001.docx]

| **ORF** | **Start** | **End** | **Len** | **Scr** | **Str** | **GI/SP** | **Top Hit** | **Top Hit Species** | **Hsp_evalue** |
| --- | --- | --- | --- | --- | --- | --- | --- | --- | --- |
| 1 | 3 | 2048 | 682 | 434.8 | + | 746620931 | preprotein translocase subunit SecA | S. hyicus | 0E+00 |
| 2 | 2101 | 3177 | 359 | 154 | + | 744781917 | peptide chain release factor 2 | S. hyicus | 0E+00 |
| 3 | 3257 | 4135 | 293 | 76.9 | + | 744781916 | hypothetical protein SHYC_09955 | S. hyicus | 5E-102 |
| 4 | 4269 | 4910 | 214 | 117.7 | + | 744781915 | putative 5'-deoxynucleotidase YfbR | S. hyicus | 2E-152 |
| 5 | 4916 | 5152 | 79 | 14.2 | + | 744781914 | hypothetical protein SHYC_09945 | S. hyicus | 1E-47 |
| 6 | 5296 | 7278 | 661 | 401 | + | 744781913 | excinuclease ABC subunit B | S. hyicus | 0E+00 |
| 7 | 7284 | 10133 | 950 | 485.5 | + | 744781912 | excinuclease ABC subunit A | S. hyicus | 0E+00 |
| 8 | 10130 | 10534 | 135 | 71 | + | 744781911 | hypothetical protein SHYC_09930 | S. hyicus | 4E-42 |
| 9 | 10702 | 11637 | 312 | 170 | + | 744781910 | HPr(Ser) kinase/phosphatase | S. hyicus | 0E+00 |
| 10 | 11637 | 12491 | 285 | 122.1 | + | 744781909 | prolipoprotein diacylglyceryl transferase | S. hyicus | 0E+00 |
| 11 | 12475 | 12960 | 162 | 43.4 | + | 744781908 | acetyltransferase | S. hyicus | 2E-98 |
| 12 | 12977 | 14413 | 479 | 192.9 | + | 744781907 | hypothetical protein SHYC_09910 | S. hyicus | 0E+00 |
| 13 | 14477 | 15421 | 315 | 231.6 | + | 744781906 | thioredoxin reductase | S. hyicus | 0E+00 |
| 14 | 15587 | 16510 | 308 | 151.1 | + | 744781905 | P-loop ATPase family protein | S. hyicus | 0E+00 |
| 15 | 16479 | 17504 | 342 | 124 | + | 744781904 | CofD family protein | S. hyicus | 0E+00 |
| 16 | 17640 | 18584 | 315 | 163.4 | + | 744781903 | WhiA family transcriptional regulator | S. hyicus | 0E+00 |
| 17 | 18626 | 19207 | 194 | 144.2 | - | 744781902 | ATP-dependent Clp protease proteolytic subunit | S. hyicus | 3E-139 |
| 18 | 19743 | 20009 | 89 | 8.2 | - |  | No Significant Match |  |  |
| 19 | 20420 | 21109 | 230 | 59.6 | + |  | No Significant Match |  |  |
| 20 | 21309 | 21893 | 195 | 107.4 | - | 744781901 | lipoprotein | S. hyicus | 8E-106 |
| 21 | 22305 | 22505 | 67 | 41.6 | + | 744781900 | cold-shock protein | S. hyicus | 1E-40 |
| 22 | 22554 | 22757 | 68 | 33.3 | - | 744781899 | hypothetical protein SHYC_09865 | S. hyicus | 3E-39 |
| 23 | 22927 | 23733 | 269 | 128.7 | + | 744781898 | glyoxalase/bleomycin resistance protein/dioxygenase superfamily protein | S. hyicus | 3E-142 |
| 24 | 23955 | 25460 | 502 | 299.7 | - | 744781897 | zinc metalloproteinase precursor/aureolysin | S. hyicus | 0E+00 |
| 25 | 25663 | 26151 | 163 | 70.2 | + | 744781896 | N-acetyltransferase | S. hyicus | 3E-108 |
| 26 | 26256 | 27158 | 301 | 144.4 | - | 744781895 | NAD dependent epimerase/dehydratase family protein | S. hyicus | 0E+00 |
| 27 | 27704 | 28366 | 221 | 100.2 | + | 744781894 | hypothetical protein SHYC_09840 | S. hyicus | 1E-97 |
| 28 | 28427 | 28684 | 86 | 19.4 | - | 744781893 | hypothetical protein SHYC_09835 | S. hyicus | 3E-37 |
| 29 | 28978 | 29994 | 339 | 140.8 | + | 744781892 | central glycolytic genes regulator | S. hyicus | 0E+00 |
| 30 | 30141 | 31148 | 336 | 232.7 | + | 744781891 | glyceraldehyde-3-phosphate dehydrogenase | S. hyicus | 0E+00 |
| 31 | 31339 | 32529 | 397 | 300.2 | + | 744781890 | phosphoglycerate kinase | S. hyicus | 0E+00 |
| 32 | 32591 | 33352 | 254 | 170.4 | + | 744781889 | triosephosphate isomerase | S. hyicus | 0E+00 |
| 33 | 33354 | 34874 | 507 | 349.3 | + | 744781888 | 2,3-bisphosphoglycerate-independent phosphoglycerate mutase | S. hyicus | 0E+00 |
| 34 | 35017 | 36321 | 435 | 312.6 | + | 744781887 | enolase | S. hyicus | 0E+00 |
| 35 | 36635 | 37102 | 156 | 58.6 | + | 744781885 | membrane protein | S. hyicus | 1E-102 |
| 36 | 37170 | 37403 | 78 | 43.7 | + | 744781884 | preprotein translocase subunit SecG | S. hyicus | 5E-46 |
| 37 | 37556 | 38296 | 247 | 136.4 | + | 744781883 | carboxylesterase | S. hyicus | 0E+00 |
| 38 | 38332 | 40686 | 785 | 461.1 | + | 744781882 | ribonuclease R | S. hyicus | 0E+00 |
| 39 | 40703 | 41167 | 155 | 94.5 | + | 744781881 | tmRNA-binding protein SmpB | S. hyicus | 5E-106 |
| 40 | 41664 | 42086 | 141 | 40.2 | + | 744781880 | hypothetical protein SHYC_09775 | S. hyicus | 3E-55 |
| 41 | 42253 | 43698 | 482 | 127.4 | - | 744781879 | major facilitator transporter | S. hyicus | 0E+00 |
| 42 | 43810 | 44199 | 130 | 41.5 | - | 744781878 | hypothetical protein SHYC_09765 | S. hyicus | 3E-64 |
| 43 | 44196 | 44711 | 172 | 70.5 | - | 744781877 | GNAT family acetyltransferase | S. hyicus | 6E-93 |
| 44 | 44995 | 45705 | 237 | 116 | + | 744781876 | 3-dehydroquinate dehydratase | S. hyicus | 5E-100 |
| 45 | 45771 | 46316 | 182 | 88.6 | + | 744781875 | nitroreductase family protein | S. hyicus | 7E-128 |
| 46 | 46421 | 46741 | 107 | 51.9 | - | 744781874 | thioredoxin | S. hyicus | 1E-70 |
| 47 | 46907 | 47260 | 118 | 78.4 | + | 744781873 | putative transcriptional regulator | S. hyicus | 1E-80 |
| 48 | 47330 | 47710 | 127 | 92.9 | + | 744781872 | glycine cleavage system protein H | S. hyicus | 4E-83 |
| 49 | 47793 | 48188 | 132 | 49.1 | + | 744781871 | TOPRIM domain containing protein | S. hyicus | 1E-90 |
| 50 | 48130 | 48468 | 113 | 29.5 | + | 744781870 | thioredoxin | S. hyicus | 1E-58 |
| 51 | 48712 | 49740 | 343 | 169.7 | + | 744781869 | methionine ABC transporter ATP-binding protein | S. hyicus | 0E+00 |
| 52 | 49733 | 50428 | 232 | 109.6 | + | 744781868 | methionine ABC transporter permease | S. hyicus | 9E-142 |
| 53 | 50453 | 51277 | 275 | 178.5 | + | 744781867 | methionine ABC transporter substrate-binding lipoprotein | S. hyicus | 1E-175 |
| 54 | 51321 | 52172 | 284 | 115.9 | - | 744781866 | membrane protein | S. hyicus | 3E-179 |
| 55 | 52482 | 53252 | 257 | 173.9 | + | 744781865 | FeS assembly ATPase SufC | S. hyicus | 0E+00 |
| 56 | 53274 | 54581 | 436 | 270.8 | + | 744781864 | FeS assembly protein SufD | S. hyicus | 0E+00 |
| 57 | 54689 | 55939 | 417 | 225.5 | + | 744781863 | cysteine desulfurase, SufS subfamily | S. hyicus | 0E+00 |
| 58 | 55929 | 56378 | 150 | 81.2 | + | 744781862 | NifU-like protein | S. hyicus | 5E-108 |
| 59 | 56452 | 57849 | 466 | 275.5 | + | 744781861 | Fe-S cluster assembly protein SufB | S. hyicus | 0E+00 |
| 60 | 58394 | 59389 | 332 | 170.7 | + | 744781860 | CBS domain protein | S. hyicus | 0E+00 |
| 61 | 59605 | 59886 | 94 | 23.3 | + | O05413 | Probable nitronate monooxygenase | Bacillus subtilis | 7E-15 |
| 62 | 60058 | 60747 | 230 | 93.1 | - | A3GGU3 | Kynurenine formamidase | Scheffersomyces stipitis | 2E-05 |
| 63 | 60870 | 61718 | 283 | 119.8 | + | 744781859 | hypothetical protein SHYC_09670 | S. hyicus | 0E+00 |
| 64 | 61734 | 62558 | 275 | 106.4 | + | 744781858 | membrane protein | S. hyicus | 3E-141 |
| 65 | 62571 | 63890 | 440 | 214.5 | + | 744781857 | 5'-nucleotidase | S. hyicus | 0E+00 |
| 66 | 63974 | 64891 | 306 | 202.4 | + | 744781856 | lipoyl synthase | S. hyicus | 0E+00 |
| 67 | 64910 | 65275 | 122 | 46.6 | + | 744781855 | hypothetical protein SHYC_09650 | S. hyicus | 2E-81 |
| 68 | 65563 | 65829 | 89 | 61.9 | - | 744781854 | hypothetical protein SHYC_09645 | S. hyicus | 1E-53 |
| 69 | 65928 | 66365 | 146 | 81.3 | + | 744781853 | hypothetical protein SHYC_09640 | S. hyicus | 1E-90 |
| 70 | 66362 | 67159 | 266 | 144.4 | + | 744781852 | HAD family hydrolase | S. hyicus | 4E-174 |
| 71 | 67152 | 68117 | 322 | 164.8 | + | 744781851 | glyoxylate/hydroxypyruvate reductase | S. hyicus | 0E+00 |
| 72 | 68626 | 70080 | 485 | 261.5 | + | 744781850 | D-alanine--poly(phosphoribitol) ligase subunit 1 | S. hyicus | 0E+00 |
| 73 | 70077 | 71297 | 407 | 136.7 | + | 744781849 | D-alanine transport protein DltB | S. hyicus | 0E+00 |
| 74 | 71314 | 71550 | 79 | 64.4 | + | 744781848 | D-alanine--poly(phosphoribitol) ligase subunit 2 | S. hyicus | 1E-47 |
| 75 | 71547 | 72695 | 383 | 178.5 | + | 744781847 | poly D-alanine transfer protein DltD | S. hyicus | 0E+00 |
| 76 | 72913 | 73155 | 81 | 59.3 | - | 744781846 | NifU family protein | S. hyicus | 2E-50 |
| 77 | 73260 | 73586 | 109 | 56 | + | 744781845 | hypothetical protein SHYC_09595 | S. hyicus | 3E-69 |
| 78 | 73681 | 74745 | 355 | 194.5 | - | 744781844 | pyridine nucleotide-disulfide oxidoreductase family protein | S. hyicus | 0E+00 |
| 79 | 75043 | 75279 | 79 | 29.2 | + | 744781843 | hypothetical protein SHYC_09585 | S. hyicus | 3E-51 |
| 80 | 75292 | 75651 | 120 | 71.9 | + | 744781842 | iron-sulfur cluster insertion protein | S. hyicus | 1E-80 |
| 81 | 75984 | 77186 | 401 | 272.9 | + | 744781841 | NADH dehydrogenase | S. hyicus | 0E+00 |
| 82 | 77279 | 78763 | 495 | 265.9 | + | 744781840 | cytosol aminopeptidase | S. hyicus | 0E+00 |
| 83 | 79004 | 80317 | 438 | 216 | + | 744781839 | Na+/H+ antiporter family protein | S. hyicus | 0E+00 |
| 84 | 80342 | 80719 | 126 | 67.4 | + | 744781838 | thioesterase family protein | S. hyicus | 1E-73 |
| 85 | 80901 | 81905 | 335 | 138.7 | - | 744781837 | hypothetical protein SHYC_09550 | S. hyicus | 0E+00 |
| 86 | 82139 | 82495 | 119 | 62.5 | - | 744781836 | monovalent cation/H+ antiporter subunit G | S. hyicus | 7E-74 |
| 87 | 82473 | 82766 | 98 | 56.1 | - | 744781835 | monovalent cation/H+ antiporter subunit F | S. hyicus | 3E-51 |
| 88 | 82766 | 83245 | 160 | 66.3 | - | 744781834 | monovalent cation/H+ antiporter subunit E | S. hyicus | 4E-109 |
| 89 | 83246 | 84748 | 501 | 209.5 | - | 744781833 | monovalent cation/H+ antiporter subunit D | S. hyicus | 0E+00 |
| 90 | 84735 | 85082 | 116 | 60.7 | - | 744781832 | monovalent cation/H+ antiporter subunit C | S. hyicus | 3E-76 |
| 91 | 85082 | 85510 | 143 | 43.1 | - | 744781831 | monovalent cation/H+ antiporter subunit B | S. hyicus | 1E-97 |
| 92 | 85491 | 87908 | 806 | 300.3 | - | 744781830 | monovalent cation/H+ antiporter subunit A | S. hyicus | 0E+00 |
| 93 | 88048 | 88428 | 127 | 66.5 | - | 744781829 | kinase associated protein B | S. hyicus | 6E-75 |
| 94 | 88502 | 89104 | 201 | 137.8 | + | 744781828 | peptidylprolyl isomerase | S. hyicus | 1E-140 |
| 95 | 89132 | 89389 | 86 | 39.1 | + | 744781827 | hypothetical protein SHYC_09500 | S. hyicus | 3E-49 |
| 96 | 89677 | 90867 | 397 | 205.2 | + | Q50228 | Formamidase | Methylophilus methylotrophus | 2E-176 |
| 97 | 90867 | 91133 | 89 | 14.8 | + |  | No Significant Match |  |  |
| 98 | 91307 | 91696 | 130 | 50.3 | + | 744781826 | general stress protein | S. hyicus | 1E-90 |
| 99 | 92247 | 93791 | 515 | 350.3 | + | 744781825 | 1-pyrroline-5-carboxylate dehydrogenase | S. hyicus | 0E+00 |
| 100 | 94017 | 95219 | 401 | 254.5 | + | 744781824 | ornithine--oxo-acid aminotransferase | S. hyicus | 0E+00 |
| 101 | 95379 | 96623 | 415 | 249.4 | + | 744781823 | NAD-specific glutamate dehydrogenase | S. hyicus | 0E+00 |
| 102 | 96749 | 97690 | 314 | 181.2 | - | 744781822 | glycerophosphodiester phosphodiesterase | S. hyicus | 0E+00 |
| 103 | 97839 | 99212 | 458 | 221.2 | - | 744781821 | argininosuccinate lyase | S. hyicus | 0E+00 |
| 104 | 99205 | 100410 | 402 | 260.7 | - | 744781820 | argininosuccinate synthase | S. hyicus | 0E+00 |
| 105 | 100604 | 101935 | 444 | 302.8 | + | 744781819 | glucose-6-phosphate isomerase | S. hyicus | 0E+00 |
| 106 | 102105 | 102626 | 174 | 55.1 | + | 744781818 | signal peptidase I | S. hyicus | 6E-96 |
| 107 | 102629 | 103210 | 194 | 95.9 | + | 744781817 | signal peptidase IB | S. hyicus | 2E-133 |
| 108 | 103360 | 106812 | 1151 | 518.2 | + | 744781816 | ATP-dependent helicase/nuclease subunit B | S. hyicus | 0E+00 |
| 109 | 106809 | 110450 | 1214 | 563.9 | + | 744781815 | ATP-dependent helicase/nuclease subunit A | S. hyicus | 0E+00 |
| 110 | 110529 | 111431 | 301 | 211 | + | 744781814 | hypothetical protein SHYC_09435 | S. hyicus | 0E+00 |
| 111 | 111682 | 112071 | 130 | 63.6 | + | 744781813 | hypothetical protein SHYC_09430 | S. hyicus | 6E-66 |
| 112 | 112291 | 113610 | 440 | 258.5 | - | 744781812 | CoA-disulfide reductase | S. hyicus | 0E+00 |
| 113 | 113623 | 114447 | 275 | 144.1 | - | 744781811 | Cof family hydrolase | S. hyicus | 0E+00 |
| 114 | 114560 | 114868 | 103 | 71.9 | + | 744781810 | hypothetical protein SHYC_09415 | S. hyicus | 6E-67 |
| 115 | 115102 | 117711 | 870 | 603.7 | + | 744781809 | ATP-dependent chaperone protein ClpB | S. hyicus | 0E+00 |
| 116 | 118061 | 118261 | 67 | 32.7 | - | 744781808 | hypothetical protein SHYC_09405 | S. hyicus | 2E-36 |
| 117 | 118412 | 119353 | 314 | 201.8 | + | 744781807 | 3-oxoacyl-ACP synthase III | S. hyicus | 0E+00 |
| 118 | 119370 | 120614 | 415 | 287 | + | 744781806 | 3-oxoacyl-ACP synthase II | S. hyicus | 0E+00 |
| 119 | 120941 | 121321 | 127 | 31.1 | - | 744781805 | membrane protein | S. hyicus | 8E-77 |
| 120 | 121569 | 122495 | 309 | 115.6 | + | 744781804 | peptide ABC transporter permease | S. hyicus | 0E+00 |
| 121 | 122495 | 123556 | 354 | 181.8 | + | 744781803 | peptide ABC transporter permease | S. hyicus | 0E+00 |
| 122 | 123571 | 124644 | 358 | 211.1 | + | 744781802 | oligopeptide ABC transporter ATP-binding protein | S. hyicus | 0E+00 |
| 123 | 124644 | 125582 | 313 | 163.6 | + | 744781801 | oligopeptide ABC transporter ATP-binding protein | S. hyicus | 0E+00 |
| 124 | 125606 | 127267 | 554 | 322.6 | + | P26906 | Dipeptide-binding protein DppE | Bacillus subtilis | 4E-91 |
| 125 | 127307 | 128299 | 331 | 188.3 | - | 744781800 | tryptophanyl-tRNA synthetase | S. hyicus | 0E+00 |
| 126 | 128605 | 129000 | 132 | 82.4 | + | 744781799 | regulatory protein Spx | S. hyicus | 8E-93 |
| 127 | 129251 | 129970 | 240 | 150 | + | 744781798 | adapter protein MecA | S. hyicus | 4E-152 |
| 128 | 130131 | 131099 | 323 | 77.5 | + | 744781797 | CoiA family protein | S. hyicus | 0E+00 |
| 129 | 131170 | 132978 | 603 | 337.2 | + | 744781796 | oligoendopeptidase F | S. hyicus | 0E+00 |
| 130 | 133244 | 134038 | 265 | 106.9 | - | 744781795 | hypothetical protein SHYC_09335 | S. hyicus | 0E+00 |
| 131 | 134053 | 134424 | 124 | 55.4 | - | 744781794 | globin family protein | S. hyicus | 3E-86 |
| 132 | 134509 | 135105 | 199 | 106.5 | - | 744781793 | adenylate cyclase | S. hyicus | 1E-104 |
| 133 | 135396 | 135743 | 116 | 50 | + | 744781792 | hypothetical protein SHYC_09320 | S. hyicus | 3E-75 |
| 134 | 135760 | 136404 | 215 | 120.8 | + | 744781791 | GTP pyrophosphokinase | S. hyicus | 2E-153 |
| 135 | 136404 | 137213 | 270 | 101 | + | 744781790 | inorganic polyphosphate/ATP-NAD kinase | S. hyicus | 0E+00 |
| 136 | 137210 | 138064 | 285 | 97.8 | + | 744781789 | pseudouridine synthase | S. hyicus | 0E+00 |
| 137 | 138092 | 139477 | 462 | 263.7 | + | 744781788 | magnesium transporter MgtE | S. hyicus | 0E+00 |
| 138 | 139488 | 141323 | 612 | 297.5 | + | 744781787 | sodium:proton antiporter | S. hyicus | 0E+00 |
| 139 | 141468 | 142235 | 256 | 172.3 | + | 744781786 | enoyl-ACP reductase | S. hyicus | 0E+00 |
| 140 | 142303 | 143409 | 369 | 143.7 | - | 744781785 | membrane protein | S. hyicus | 0E+00 |
| 141 | 143601 | 145184 | 528 | 278.9 | + | 744781784 | sodium:alanine symporter family protein | S. hyicus | 0E+00 |
| 142 | 145312 | 146052 | 247 | 107 | + | 744781783 | putative esterase | S. hyicus | 5E-165 |
| 143 | 146103 | 146612 | 170 | 116.8 | + | 744781782 | putative RNA ligase or phosphoesterase | S. hyicus | 2E-116 |
| 144 | 146776 | 147084 | 103 | 30.8 | + | 744781781 | hypothetical protein SHYC_09265 | S. hyicus | 5E-56 |
| 145 | 147107 | 148294 | 396 | 118.3 | - | 744781780 | putative glycolipid permease LtaA | S. hyicus | 0E+00 |
| 146 | 148272 | 149447 | 392 | 180 | - | 744781779 | processive diacylglycerol beta-glucosyltransferase | S. hyicus | 0E+00 |
| 147 | 149959 | 151443 | 495 | 295.9 | + | 744781778 | UDP-N-acetylmuramoylalanyl-D-glutamate--L-lysine ligase | S. hyicus | 0E+00 |
| 148 | 151433 | 151672 | 80 | 31.5 | + | 744781777 | YueH family protein | S. hyicus | 2E-51 |
| 149 | 151685 | 153247 | 521 | 325.4 | + | 744781776 | peptide chain release factor 3 | S. hyicus | 0E+00 |
| 150 | 153552 | 154352 | 267 | 142 | + | 744781775 | TerC family membrane protein | S. hyicus | 2E-173 |
| 151 | 154536 | 156146 | 537 | 291 | + | 744781774 | serine protease HtrA | S. hyicus | 0E+00 |
| 152 | 156163 | 157521 | 453 | 169.4 | + | 744781773 | cation transport protein | S. hyicus | 0E+00 |
| 153 | 157683 | 159176 | 498 | 214.2 | + | 744781772 | 2', 3'-cyclic nucleotide 2'-phosphodiesterase | S. hyicus | 0E+00 |
| 154 | 159604 | 162108 | 835 | 449.8 | + | 744781771 | fibronectin-binding protein | S. hyicus | 0E+00 |
| 155 | 162247 | 162849 | 201 | 56.8 | - | 744781770 | CAAX amino protease family protein | S. hyicus | 4E-135 |
| 156 | 162856 | 163041 | 62 | 26.9 | - | 744781769 | hypothetical protein SHYC_09205 | S. hyicus | 1E-36 |
| 157 | 163217 | 164209 | 331 | 187.6 | + | 744781768 | lipoate-protein ligase LplJ | S. hyicus | 0E+00 |
| 158 | 164265 | 164492 | 76 | 43.5 | - | 744781767 | IDEAL domain protein | S. hyicus | 1E-40 |
| 159 | 164719 | 165357 | 213 | 81.5 | + | 744781766 | competence transcription factor ComK | S. hyicus | 1E-141 |
| 160 | 165478 | 165633 | 52 | 8.4 | + | 744781765 | hypothetical protein SHYC_09185 | S. hyicus | 3E-19 |
| 161 | 165731 | 166180 | 150 | 70.6 | + | O32248 | Uncharacterized N-acetyltransferase YvbK | Bacillus subtilis | 7E-31 |
| 162 | 166613 | 166768 | 52 | 7 | + | 744781764 | YolD family protein | S. hyicus | 2E-22 |
| 163 | 166820 | 167200 | 127 | 41.8 | - | P0A041 | Penicillinase repressor | S. epidermidis | 4E-77 |
| 164 | 167190 | 168947 | 586 | 117.5 | - | P18357 | Regulatory protein BlaR1 | S. aureus | 0E+00 |
| 165 | 169054 | 169899 | 282 | 122.5 | + | P00807 | Beta-lactamase | S. aureus | 0E+00 |
| 166 | 170213 | 170623 | 137 | 54.6 | + | 744781764 | YolD family protein | S. hyicus | 8E-27 |
| 167 | 170666 | 172036 | 457 | 145.1 | - |  | No Significant Match |  |  |
| 168 | 172041 | 172406 | 122 | 57.4 | - | P0A049 | Transposase for transposon Tn554 | S. aureus | 1E-14 |
| 169 | 172415 | 174478 | 688 | 176 | - | P37375 | Transposase B from transposon PsiTn554 | S. aureus | 9E-51 |
| 170 | 174481 | 175593 | 371 | 94.9 | - | P0A052 | Transposase A from transposon Tn554 | S. aureus | 3E-67 |
| 171 | 176446 | 177495 | 350 | 130.4 | - | Q8TZV9 | Probable tyrosine recombinase XerC-like | Pyrococcus furiosus | 1E-09 |
| 172 | 177808 | 178431 | 208 | 42.6 | + |  | No Significant Match |  |  |
| 173 | 178796 | 178957 | 54 | 33.5 | - |  | No Significant Match |  |  |
| 174 | 178970 | 179641 | 224 | 74.6 | - |  | No Significant Match |  |  |
| 175 | 179655 | 179993 | 113 | 60.5 | - | P23789 | HTH-type transcriptional regulator Xre | Bacillus subtilis | 5E-06 |
| 176 | 180257 | 180451 | 65 | 39.3 | + |  | No Significant Match |  |  |
| 177 | 180451 | 181239 | 263 | 142.9 | + | P44193 | Uncharacterized protein HI_1422 | Haemophilus influenzae | 3E-12 |
| 178 | 181251 | 181472 | 74 | 52.8 | + | 686149522 | hypothetical protein | S. aureus | 3E-41 |
| 179 | 181508 | 181678 | 57 | 8.3 | + |  | No Significant Match |  |  |
| 180 | 181606 | 181818 | 71 | 35.1 | - |  | No Significant Match |  |  |
| 181 | 181891 | 182103 | 71 | 25.1 | + |  | No Significant Match |  |  |
| 182 | 182097 | 182270 | 58 | 15.3 | + |  | No Significant Match |  |  |
| 183 | 182350 | 182616 | 89 | 51.6 | + |  | No Significant Match |  |  |
| 184 | 182613 | 182831 | 73 | 29.8 | + |  | No Significant Match |  |  |
| 185 | 182832 | 183494 | 221 | 90.2 | + |  | No Significant Match |  |  |
| 186 | 183494 | 183892 | 133 | 73.1 | + | P59933 | Single-stranded DNA-binding protein | Wolinella succinogenes | 1E-20 |
| 187 | 183904 | 184578 | 225 | 34.5 | + |  | No Significant Match |  |  |
| 188 | 184575 | 184778 | 68 | 2.1 | + |  | No Significant Match |  |  |
| 189 | 184817 | 185617 | 267 | 131.3 | + |  | No Significant Match |  |  |
| 190 | 185614 | 185955 | 114 | 32.8 | + |  | No Significant Match |  |  |
| 191 | 185948 | 187186 | 413 | 149.7 | + | P57611 | Replicative DNA helicase | Buchnera aphidicola | 3E-45 |
| 192 | 187183 | 187401 | 73 | 7.7 | + |  | No Significant Match |  |  |
| 193 | 187388 | 187810 | 141 | 52.1 | + | P45911 | Uncharacterized protein YqaN | Bacillus subtilis | 1E-07 |
| 194 | 187812 | 188234 | 141 | 52.7 | + |  | No Significant Match |  |  |
| 195 | 188231 | 188641 | 137 | 52.1 | + |  | No Significant Match |  |  |
| 196 | 188638 | 189132 | 165 | 84.7 | + | 744781020 | putative nucleoside 2-deoxyribosyltransferase | S. hyicus | 5E-24 |
| 197 | 189125 | 189337 | 71 | 20.5 | + |  | No Significant Match |  |  |
| 198 | 189349 | 189753 | 135 | 63.6 | + | 744780978 | hypothetical protein SHYC_05120 | S. hyicus | 5E-09 |
| 199 | 189750 | 190082 | 111 | 27.9 | + | 744780981 | hypothetical protein SHYC_05135 | S. hyicus | 8E-20 |
| 200 | 190066 | 190362 | 99 | 48.7 | + | Q38441 | Uncharacterized 10.3 kDa protein in GP2-GP6 intergenic region | Bacillus phage | 1E-16 |
| 201 | 190325 | 190540 | 72 | 15 | + | 744780982 | hypothetical protein SHYC_05140 | S. hyicus | 5E-29 |
| 202 | 190533 | 190763 | 77 | 16.4 | + | 744780983 | hypothetical protein SHYC_05145 | S. hyicus | 1E-29 |
| 203 | 190747 | 191106 | 120 | 44 | + | 746621824 | hypothetical protein, partial | S. hyicus | 6E-37 |
| 204 | 191107 | 191625 | 173 | 102.7 | + | Q6BRN7 | Deoxyuridine 5'-triphosphate nucleotidohydrolase | Debaryomyces hansenii | 1E-23 |
| 205 | 191944 | 192432 | 163 | 82.5 | + |  | No Significant Match |  |  |
| 206 | 192443 | 192904 | 154 | 56.5 | + |  | No Significant Match |  |  |
| 207 | 193034 | 193426 | 131 | 3 | + |  | No Significant Match |  |  |
| 208 | 193559 | 194029 | 157 | 69.7 | + |  | No Significant Match |  |  |
| 209 | 194022 | 195155 | 378 | 145.5 | + | P59217 | Putative terminase large subunit | Shigella phage | 3E-40 |
| 210 | 195429 | 195728 | 100 | 5.8 | - | P37248 | Transposase for insertion sequence element IS1086 | Ralstonia metallidurans | 4E-15 |
| 211 | 195883 | 196413 | 177 | 75.6 | - |  | No Significant Match |  |  |
| 212 | 196525 | 196887 | 121 | 76.5 | - |  | No Significant Match |  |  |
| 213 | 196934 | 197527 | 198 | 101.1 | - |  | No Significant Match |  |  |
| 214 | 197533 | 198561 | 343 | 190.1 | - | 746621932 | hypothetical protein, partial | S. hyicus | 7E-23 |
| 215 | 198551 | 200482 | 644 | 303.8 | - | P96644 | Uncharacterized membrane protein YddG | Bacillus subtilis | 9E-46 |
| 216 | 200582 | 200983 | 134 | 71 | - |  | No Significant Match |  |  |
| 217 | 200988 | 202346 | 453 | 247.3 | - | P96634 | Ftsk domain-containing protein YdcQ | Bacillus subtilis | 7E-63 |
| 218 | 202351 | 202683 | 111 | 37.7 | - |  | No Significant Match |  |  |
| 219 | 202680 | 202910 | 77 | 27.6 | - |  | No Significant Match |  |  |
| 220 | 202914 | 203132 | 73 | 27.9 | - |  | No Significant Match |  |  |
| 221 | 203144 | 205639 | 832 | 432.1 | - | P96642 | Uncharacterized protein YddE | Bacillus subtilis | 0E+00 |
| 222 | 205674 | 206063 | 130 | 54 | - | P96641 | Uncharacterized protein YddD | Bacillus subtilis | 2E-05 |
| 223 | 206069 | 206329 | 87 | 41 | - |  | No Significant Match |  |  |
| 224 | 206334 | 207410 | 359 | 159.4 | - | O31491 | Uncharacterized protein YddB | Bacillus subtilis | 2E-20 |
| 225 | 207638 | 208777 | 380 | 111.5 | - | P96635 | Putative DNA relaxase NicK | Bacillus subtilis | 1E-57 |
| 226 | 208943 | 209368 | 142 | 87.6 | - |  | No Significant Match |  |  |
| 227 | 209390 | 209713 | 108 | 56.2 | - |  | No Significant Match |  |  |
| 228 | 209868 | 210152 | 95 | 7.1 | - |  | No Significant Match |  |  |
| 229 | 210239 | 210844 | 202 | 53.9 | + |  | No Significant Match |  |  |
| 230 | 210856 | 211041 | 62 | 20.3 | + |  | No Significant Match |  |  |
| 231 | 211051 | 212280 | 410 | 153.5 | + | P49859 | Portal protein | Enterobacteria phage | 6E-11 |
| 232 | 212267 | 212830 | 188 | 90.3 | + |  | No Significant Match |  |  |
| 233 | 212870 | 214186 | 439 | 273.1 | + |  | No Significant Match |  |  |
| 234 | 214206 | 214379 | 58 | 39.7 | + |  | No Significant Match |  |  |
| 235 | 214388 | 214699 | 104 | 44.6 | + |  | No Significant Match |  |  |
| 236 | 214681 | 215004 | 108 | 42.9 | + |  | No Significant Match |  |  |
| 237 | 215001 | 215426 | 142 | 64.3 | + |  | No Significant Match |  |  |
| 238 | 215416 | 215829 | 138 | 50.9 | + |  | No Significant Match |  |  |
| 239 | 215836 | 216693 | 286 | 179.5 | + |  | No Significant Match |  |  |
| 240 | 216760 | 217299 | 180 | 122.2 | + |  | No Significant Match |  |  |
| 241 | 217500 | 223304 | 1935 | 757.8 | + | Q0PDK7 | Tail tape measure protein gp18 | Bacillus phage | 2E-31 |
| 242 | 223305 | 224795 | 497 | 196 | + |  | No Significant Match |  |  |
| 243 | 224797 | 228279 | 1161 | 544.5 | + |  | No Significant Match |  |  |
| 244 | 228290 | 228439 | 50 | 30.1 | + |  | No Significant Match |  |  |
| 245 | 228482 | 228814 | 111 | 47.2 | + |  | No Significant Match |  |  |
| 246 | 228833 | 229132 | 100 | 48.3 | + |  | No Significant Match |  |  |
| 247 | 229263 | 229697 | 145 | 77.4 | + |  | No Significant Match |  |  |
| 248 | 229678 | 231123 | 482 | 222 | + | P24556 | Autolysin | S. aureus | 0E+00 |
| 249 | 231496 | 232722 | 409 | 134.2 | + |  | No Significant Match |  |  |
| 250 | 233205 | 233699 | 165 | 117.7 | - | 744781073 | alkaline shock protein 23 | S. hyicus | 1E-102 |
| 251 | 233741 | 233983 | 81 | 21.3 | - | 744781072 | membrane protein | S. hyicus | 3E-39 |
| 252 | 234004 | 234549 | 182 | 67.7 | - | 744781071 | hypothetical protein SHYC_05665 | S. hyicus | 7E-105 |
| 253 | 235421 | 235978 | 186 | 95 | + | 744781763 | acetyltransferase | S. hyicus | 5E-110 |
| 254 | 235990 | 236637 | 216 | 110 | + | 744781762 | ABC transporter ATP-binding protein | S. hyicus | 2E-145 |
| 255 | 236634 | 237410 | 259 | 130.7 | + | 744781761 | membrane protein | S. hyicus | 1E-165 |
| 256 | 237438 | 238376 | 313 | 119.5 | - | 744781760 | 1,4-dihydroxy-2-naphthoate octaprenyltransferase | S. hyicus | 0E+00 |
| 257 | 238551 | 239903 | 451 | 180 | + | 744781759 | isochorismate synthase | S. hyicus | 0E+00 |
| 258 | 239905 | 241578 | 558 | 270.5 | + | 744781758 | 2-succinyl-5-enolpyruvyl-6-hydroxy-3- cyclohexene-1-carboxylate synthase | S. hyicus | 0E+00 |
| 259 | 241565 | 242407 | 281 | 117.7 | + | 744781757 | 2-succinyl-6-hydroxy-2, 4-cyclohexadiene-1-carboxylate synthase | S. hyicus | 2E-166 |
| 260 | 242373 | 243191 | 273 | 176.5 | + | 744781756 | dihydroxynaphthoic acid synthetase | S. hyicus | 0E+00 |
| 261 | 243281 | 244447 | 389 | 167.7 | - | 744781755 | N-acetyl-L,L-diaminopimelate aminotransferase | S. hyicus | 0E+00 |
| 262 | 244519 | 245526 | 336 | 102.3 | - | 744781754 | acyltransferase | S. hyicus | 0E+00 |
| 263 | 245892 | 249593 | 1234 | 641.9 | - | 744781753 | autolysin | S. hyicus | 0E+00 |
| 264 | 249791 | 250219 | 143 | 67.2 | - | 744781752 | acetyltransferase | S. hyicus | 2E-87 |
| 265 | 250431 | 250901 | 157 | 68.8 | - | 744781751 | hypothetical protein SHYC_09100 | S. hyicus | 6E-112 |
| 266 | 250919 | 252139 | 407 | 197.6 | - | 744781750 | LytR family transcriptional regulator | S. hyicus | 0E+00 |
| 267 | 252450 | 254078 | 543 | 249.1 | + | 744781749 | phosphoglucomutase | S. hyicus | 0E+00 |
| 268 | 254619 | 255884 | 422 | 224.6 | + | 744781748 | UDP-N-acetyl-D-mannosamine dehydrogenase | S. hyicus | 0E+00 |
| 269 | 255910 | 256722 | 271 | 68.5 | + | 744781747 | putative teichoic acid ABC transporter permease | S. hyicus | 5E-178 |
| 270 | 256743 | 257936 | 398 | 169.6 | + | 744781746 | glycoside hydrolase | S. hyicus | 0E+00 |
| 271 | 257937 | 258497 | 187 | 72.9 | + | 744781745 | hypothetical protein SHYC_09070 | S. hyicus | 3E-104 |
| 272 | 258494 | 261049 | 852 | 360.6 | + | 744781744 | glycosyltransferase | S. hyicus | 0E+00 |
| 273 | 261049 | 262185 | 379 | 176.9 | + | 744781743 | glycosyltransferase | S. hyicus | 0E+00 |
| 274 | 262178 | 262537 | 120 | 60.9 | + | 744781742 | hypothetical protein SHYC_09055 | S. hyicus | 1E-75 |
| 275 | 262553 | 263272 | 240 | 83.3 | + | 744781741 | hypothetical protein SHYC_09050 | S. hyicus | 8E-148 |
| 276 | 263269 | 265272 | 668 | 283.9 | + | 744781740 | hypothetical protein SHYC_09045 | S. hyicus | 0E+00 |
| 277 | 265291 | 266199 | 303 | 135.3 | + | 744781739 | teichoic acid ABC transporter ATP-binding protein | S. hyicus | 0E+00 |
| 278 | 266423 | 267772 | 450 | 220.1 | + | 744781738 | glycoside hydrolase family protein | S. hyicus | 0E+00 |
| 279 | 267980 | 268270 | 97 | 28.2 | - | 744781737 | quinol oxidase subunit IV | S. hyicus | 4E-59 |
| 280 | 268267 | 268872 | 202 | 78.6 | - | 744781736 | quinol oxidase subunit III | S. hyicus | 1E-142 |
| 281 | 268862 | 270853 | 664 | 276 | - | 744781735 | quinol oxidase subunit I | S. hyicus | 0E+00 |
| 282 | 270853 | 271944 | 364 | 169.3 | - | 744781734 | quinol oxidase subunit II | S. hyicus | 0E+00 |
| 283 | 272421 | 272759 | 113 | 69.7 | - | 744781733 | hypothetical protein SHYC_09010 | S. hyicus | 1E-70 |
| 284 | 272967 | 274112 | 382 | 251 | + | 744781732 | nitric oxide dioxygenase | S. hyicus | 0E+00 |
| 285 | 274266 | 275123 | 286 | 188.6 | - | 744781731 | bifunctional 5,10-methylene-tetrahydrofolate dehydrogenase/ 5,10-methylene-tetrahydrofolate cyclohydrolase | S. hyicus | 0E+00 |
| 286 | 275334 | 275816 | 161 | 67.1 | + | 744781730 | phosphoribosylaminoimidazole carboxylase | S. hyicus | 2E-113 |
| 287 | 275803 | 276930 | 376 | 211 | + | 744781729 | phosphoribosylaminoimidazole carboxylase ATPase subunit | S. hyicus | 0E+00 |
| 288 | 276927 | 277634 | 236 | 152.2 | + | 744781728 | phosphoribosylaminoimidazole-succinocarboxamide synthase | S. hyicus | 1E-165 |
| 289 | 277637 | 277906 | 90 | 67.2 | + | 744781727 | phosphoribosylformylglycinamidine synthase subunit PurS | S. hyicus | 2E-57 |
| 290 | 277906 | 278577 | 224 | 130.7 | + | 744781726 | phosphoribosylformylglycinamidine synthase | S. hyicus | 4E-166 |
| 291 | 278570 | 280759 | 730 | 470.7 | + | 744781725 | phosphoribosylformylglycinamidine synthase II | S. hyicus | 0E+00 |
| 292 | 280738 | 282159 | 474 | 233.4 | + | 744781724 | amidophosphoribosyltransferase | S. hyicus | 0E+00 |
| 293 | 282164 | 283192 | 343 | 225.3 | + | 744781723 | phosphoribosylaminoimidazole synthetase | S. hyicus | 0E+00 |
| 294 | 283195 | 283761 | 189 | 76.5 | + | 744781722 | phosphoribosylglycinamide formyltransferase | S. hyicus | 2E-134 |
| 295 | 283774 | 285252 | 493 | 316.3 | + | 744781721 | bifunctional purine biosynthesis protein PurH | S. hyicus | 0E+00 |
| 296 | 285267 | 286511 | 415 | 277 | + | 744781720 | phosphoribosylamine--glycine ligase | S. hyicus | 0E+00 |
| 297 | 286712 | 287503 | 264 | 66.6 | - | 744781719 | ABC transporter permease | S. hyicus | 0E+00 |
| 298 | 287500 | 288894 | 465 | 198.7 | - | 744781718 | ABC transporter ATP-binding protein | S. hyicus | 0E+00 |
| 299 | 288907 | 289491 | 195 | 110.9 | - | 744781717 | ABC transporter permease | S. hyicus | 3E-135 |
| 300 | 289964 | 290095 | 44 | 39.2 | - | 744781716 | hypothetical protein SHYC_08925 | S. hyicus | 1E-19 |
| 301 | 290420 | 291760 | 447 | 153.2 | + | 744781715 | membrane protein | S. hyicus | 0E+00 |
| 302 | 291947 | 293125 | 393 | 183.8 | + | 744781714 | ribosomal RNA large subunit methyltransferase I | S. hyicus | 0E+00 |
| 303 | 293174 | 293704 | 177 | 91.5 | + | 744781713 | hypothetical protein SHYC_08910 | S. hyicus | 5E-122 |
| 304 | 293862 | 294128 | 89 | 65.2 | + | 744781712 | phosphocarrier protein HPr | S. hyicus | 3E-57 |
| 305 | 294128 | 295849 | 574 | 384.8 | + | 744781711 | phosphoenolpyruvate-protein phosphotransferase | S. hyicus | 0E+00 |
| 306 | 295904 | 296131 | 76 | 33 | - | 744781710 | glutaredoxin | S. hyicus | 3E-41 |
| 307 | 296369 | 297721 | 451 | 190.5 | + | 744781709 | cytochrome D ubiquinol oxidase subunit I | S. hyicus | 0E+00 |
| 308 | 297718 | 298737 | 340 | 145.2 | + | 744781708 | cytochrome D ubiquinol oxidase subunit II | S. hyicus | 0E+00 |
| 309 | 298912 | 299571 | 220 | 148.8 | + | 744781707 | potassium transporter TrkA | S. hyicus | 8E-155 |
| 310 | 299971 | 300528 | 186 | 95.8 | + | 744781706 | superantigen-like protein | S. hyicus | 3E-111 |
| 311 | 301006 | 302688 | 561 | 301.9 | - | 744781705 | ribonuclease J | S. hyicus | 0E+00 |
| 312 | 302688 | 302903 | 72 | 46.7 | - | 744781704 | hypothetical protein SHYC_08865 | S. hyicus | 1E-42 |
| 313 | 303371 | 303640 | 90 | 40 | + | 744781703 | hypothetical protein SHYC_08860 | S. hyicus | 1E-40 |
| 314 | 303692 | 304243 | 184 | 96.5 | - | 744781702 | peptide deformylase | S. hyicus | 1E-131 |
| 315 | 304367 | 304999 | 211 | 118.5 | + | 744781701 | YkyA family lipoprotein | S. hyicus | 2E-125 |
| 316 | 305158 | 306267 | 370 | 237 | + | 744781700 | pyruvate dehydrogenase E1 component subunit alpha | S. hyicus | 0E+00 |
| 317 | 306271 | 307248 | 326 | 236.3 | + | 744781699 | pyruvate dehydrogenase E1 component subunit beta | S. hyicus | 0E+00 |
| 318 | 307457 | 308752 | 432 | 295 | + | 744781698 | pyruvate dehydrogenase complex E2 component, dihydrolipoamide acetyltransferase | S. hyicus | 0E+00 |
| 319 | 308756 | 310162 | 469 | 364.6 | + | 744781697 | dihydrolipoamide dehydrogenase | S. hyicus | 0E+00 |
| 320 | 310244 | 310516 | 91 | 38.4 | + | 744781696 | hypothetical protein SHYC_08825 | S. hyicus | 6E-50 |
| 321 | 310619 | 311158 | 180 | 103.4 | + | 744781695 | transcriptional regulator | S. hyicus | 4E-122 |
| 322 | 311170 | 312270 | 367 | 210.3 | + | 744781694 | spermidine/putrescine ABC transporter ATP-binding protein | S. hyicus | 0E+00 |
| 323 | 312257 | 313063 | 269 | 103.8 | + | 744781693 | spermidine/putrescine ABC transporter permease | S. hyicus | 0E+00 |
| 324 | 313060 | 313872 | 271 | 114.2 | + | 744781692 | spermidine/purescine ABC transporter permease | S. hyicus | 0E+00 |
| 325 | 313869 | 314939 | 357 | 193.7 | + | 744781691 | spermidine/putrescine ABC transporter substrate-binding protein | S. hyicus | 0E+00 |
| 326 | 315003 | 316061 | 353 | 172.1 | + | 744781690 | hypothetical protein SHYC_08795 | S. hyicus | 0E+00 |
| 327 | 316231 | 317046 | 272 | 138.3 | + | 744781689 | hypothetical protein SHYC_08790 | S. hyicus | 4E-133 |
| 328 | 317100 | 317711 | 204 | 124.1 | - | 744781688 | hypothetical protein SHYC_08785 | S. hyicus | 2E-137 |
| 329 | 317826 | 318638 | 271 | 122.3 | + | 744781687 | inositol monophosphatase | S. hyicus | 2E-180 |
| 330 | 318694 | 318879 | 62 | 9.8 | - | 744781686 | membrane protein | S. hyicus | 3E-33 |
| 331 | 318977 | 320824 | 616 | 412.7 | + | 744781685 | GTP-binding protein TypA | S. hyicus | 0E+00 |
| 332 | 321010 | 322134 | 375 | 218 | + | 744781684 | NADH-dependent flavin oxidoreductase | S. hyicus | 0E+00 |
| 333 | 322370 | 322573 | 68 | 13.6 | - | 744781683 | hypothetical protein SHYC_08760 | S. hyicus | 4E-25 |
| 334 | 322574 | 323056 | 161 | 75.2 | - | 744781682 | hypothetical protein SHYC_08755 | S. hyicus | 1E-106 |
| 335 | 323192 | 323467 | 92 | 49.8 | + | 744781681 | hypothetical protein SHYC_08750 | S. hyicus | 5E-60 |
| 336 | 323544 | 324764 | 407 | 128.3 | + | 744781680 | cell division protein FtsW | S. hyicus | 0E+00 |
| 337 | 325031 | 328480 | 1150 | 701.9 | + | 744781679 | pyruvate carboxylase | S. hyicus | 0E+00 |
| 338 | 328708 | 329568 | 287 | 71.7 | - | 744781678 | heme A synthase | S. hyicus | 0E+00 |
| 339 | 329829 | 330740 | 304 | 114 | + | 744781677 | protoheme IX farnesyltransferase | S. hyicus | 0E+00 |
| 340 | 330765 | 331226 | 154 | 75.9 | + | 744781676 | membrane protein | S. hyicus | 6E-104 |
| 341 | 331350 | 332423 | 358 | 180 | + | 744781675 | hypothetical protein SHYC_08720 | S. hyicus | 0E+00 |
| 342 | 332416 | 332853 | 146 | 70.4 | + | 744781674 | regulatory protein | S. hyicus | 5E-86 |
| 343 | 332962 | 333894 | 311 | 132 | - | 744781673 | glycerophosphodiester phosphodiesterase | S. hyicus | 8E-155 |
| 344 | 333981 | 334229 | 83 | 37.7 | + | 744781672 | hypothetical protein SHYC_08705 | S. hyicus | 1E-49 |
| 345 | 334230 | 334619 | 130 | 50.4 | - | 744781671 | hypothetical protein SHYC_08700 | S. hyicus | 3E-85 |
| 346 | 334679 | 335230 | 184 | 98.4 | + | 744781670 | ribosomal RNA small subunit methyltransferase D | S. hyicus | 8E-122 |
| 347 | 335223 | 335717 | 165 | 90.4 | + | 744781669 | phosphopantetheine adenylyltransferase | S. hyicus | 6E-110 |
| 348 | 335803 | 336939 | 379 | 94.6 | - | 744781668 | HIGH Nucleotidyl Transferase | S. hyicus | 0E+00 |
| 349 | 337066 | 337608 | 181 | 103.3 | + | 744781667 | hypothetical protein SHYC_08680 | S. hyicus | 1E-129 |
| 350 | 337651 | 337824 | 58 | 20.6 | + | 744781666 | 50S ribosomal protein L32 | S. hyicus | 5E-35 |
| 351 | 337968 | 338714 | 249 | 144.2 | + | 744781665 | TrmH family RNA methyltransferase | S. hyicus | 3E-155 |
| 352 | 339013 | 340077 | 355 | 211.9 | + | 744781664 | phenylalanyl-tRNA synthase subunit alpha | S. hyicus | 0E+00 |
| 353 | 340071 | 342476 | 802 | 498 | + | 744781663 | phenylalanyl-tRNA synthase subunit beta | S. hyicus | 0E+00 |
| 354 | 342695 | 343621 | 309 | 148.8 | - | 744781662 | ribonuclease HIII | S. hyicus | 0E+00 |
| 355 | 343821 | 344087 | 89 | 44.8 | + | 744781661 | cell division protein ZapA | S. hyicus | 1E-56 |
| 356 | 344089 | 344610 | 174 | 40.5 | + | 744781660 | membrane protein | S. hyicus | 2E-99 |
| 357 | 344686 | 346395 | 570 | 320.4 | + | 744781659 | DNA polymerase/3'-5' exonuclease PolX | S. hyicus | 0E+00 |
| 358 | 346576 | 348924 | 783 | 418.6 | + | 744781658 | recombination and DNA strand exchange inhibitor protein | S. hyicus | 0E+00 |
| 359 | 349000 | 349314 | 105 | 74.9 | + | 744781657 | thioredoxin | S. hyicus | 1E-68 |
| 360 | 349577 | 351298 | 574 | 249.4 | + | 746621899 | excinuclease ABC subunit C | S. hyicus | 0E+00 |
| 361 | 351461 | 352072 | 204 | 59.8 | + | 744781655 | succinate dehydrogenase cytochrome b558 subunit | S. hyicus | 5E-144 |
| 362 | 352197 | 353963 | 589 | 354.3 | + | 744781654 | succinate dehydrogenase flavoprotein subunit | S. hyicus | 0E+00 |
| 363 | 353963 | 354784 | 274 | 129.3 | + | 744781653 | succinate dehydrogenase iron-sulfur subunit | S. hyicus | 0E+00 |
| 364 | 354979 | 355779 | 267 | 138.8 | + | 744781652 | glutamate racemase | S. hyicus | 0E+00 |
| 365 | 355793 | 356380 | 196 | 127.1 | + | 744781651 | non-canonical purine NTP pyrophosphatase | S. hyicus | 6E-126 |
| 366 | 356377 | 356877 | 167 | 90.8 | + | 746620454 | phosphoesterase | S. hyicus | 8E-104 |
| 367 | 356987 | 357301 | 105 | 55.2 | - | 746620452 | hypothetical protein | S. hyicus | 5E-64 |
| 368 | 357437 | 358297 | 287 | 107.5 | + | 744781648 | M23 family peptidase | S. hyicus | 0E+00 |
| 369 | 358699 | 359937 | 413 | 143 | - | 744781647 | FemAB family protein | S. hyicus | 0E+00 |
| 370 | 360241 | 360606 | 122 | 62.8 | - | 744781646 | staphylococcal complement inhibitor | S. hyicus | 2E-55 |
| 371 | 361177 | 362337 | 387 | 117.7 | + | P94577 | Uncharacterized MFS-type transporter YwoG | Bacillus subtilis | 4E-37 |
| 372 | 364601 | 364708 | 36 | 12.5 | - | 744781645 | hypothetical protein SHYC_08565 | S. hyicus | 1E-09 |
| 373 | 364748 | 365188 | 147 | 54.9 | - |  | No Significant Match |  |  |
| 374 | 365698 | 367002 | 435 | 169.7 | - | 744781644 | potassium transporter KtrB | S. hyicus | 0E+00 |
| 375 | 367805 | 367939 | 45 | 31.8 | + | 744781643 | putative hemolysin | S. hyicus | 2E-21 |
| 376 | 368037 | 368486 | 150 | 80.5 | - | 744781642 | putative N-acetyltransferase | S. hyicus | 2E-103 |
| 377 | 368905 | 370518 | 538 | 228.7 | + | 744781641 | BshC family protein | S. hyicus | 0E+00 |
| 378 | 370664 | 371095 | 144 | 69.6 | + | 744781640 | protein MraZ | S. hyicus | 6E-98 |
| 379 | 371130 | 372065 | 312 | 128.6 | + | 744781639 | ribosomal RNA small subunit methyltransferase H | S. hyicus | 0E+00 |
| 380 | 372083 | 372496 | 138 | 37.9 | + | 744781638 | cell division protein FtsL | S. hyicus | 9E-89 |
| 381 | 372477 | 374618 | 714 | 368.6 | + | 744781637 | penicillin-binding protein | S. hyicus | 0E+00 |
| 382 | 374781 | 375746 | 322 | 143.9 | + | 744781636 | phospho-N-acetylmuramoyl-pentapeptide- transferase | S. hyicus | 0E+00 |
| 383 | 375747 | 377096 | 450 | 214 | + | 744781635 | UDP-N-acetylmuramoyl-L-alanyl-D-glutamate synthetase | S. hyicus | 0E+00 |
| 384 | 377111 | 377998 | 296 | 160.4 | + | 744781634 | cell division protein FtsQ | S. hyicus | 0E+00 |
| 385 | 378106 | 379545 | 480 | 294.7 | + | 744781633 | cell division protein FtsA | S. hyicus | 0E+00 |
| 386 | 379575 | 380744 | 390 | 283.3 | + | 744781632 | cell division protein FtsZ | S. hyicus | 0E+00 |
| 387 | 380924 | 381715 | 264 | 124.3 | + | 744781631 | laccase | S. hyicus | 5E-169 |
| 388 | 381742 | 382416 | 225 | 115.1 | + | 744781630 | YggS family pyridoxal phosphate enzyme | S. hyicus | 4E-151 |
| 389 | 382422 | 383033 | 204 | 111.5 | + | 744781629 | cell division protein SepF | S. hyicus | 4E-122 |
| 390 | 383045 | 383335 | 97 | 37.1 | + | 744781628 | YggT family cell division protein | S. hyicus | 3E-55 |
| 391 | 383476 | 384246 | 257 | 131.3 | + | 744781627 | RNA-binding S4 domain protein | S. hyicus | 1E-172 |
| 392 | 384270 | 384887 | 206 | 143.7 | + | 744781626 | septum site-determining protein DivIVA | S. hyicus | 2E-128 |
| 393 | 385077 | 387824 | 916 | 565.5 | + | 744781625 | isoleucyl-tRNA synthetase | S. hyicus | 0E+00 |
| 394 | 388272 | 388478 | 69 | 26.3 | + | 744781624 | hypothetical protein SHYC_08455 | S. hyicus | 5E-36 |
| 395 | 388600 | 389079 | 160 | 64.5 | + | 744781623 | lipoprotein signal peptidase | S. hyicus | 3E-105 |
| 396 | 389089 | 390009 | 307 | 166.7 | + | 744781622 | ribosomal large subunit pseudouridine synthase D | S. hyicus | 0E+00 |
| 397 | 390299 | 390823 | 175 | 85.3 | + | 744781621 | pyrimidine regulatory protein PyrR/uracil phosphoribosyltransferase | S. hyicus | 2E-117 |
| 398 | 390986 | 392278 | 431 | 214.9 | + | 744781620 | uracil permease | S. hyicus | 0E+00 |
| 399 | 392299 | 393177 | 293 | 135.7 | + | 744781619 | aspartate carbamoyltransferase | S. hyicus | 0E+00 |
| 400 | 393191 | 394465 | 425 | 275.3 | + | 744781618 | dihydroorotase | S. hyicus | 0E+00 |
| 401 | 394465 | 395559 | 365 | 203.7 | + | 744781617 | carbamoyl phosphate synthase small subunit | S. hyicus | 0E+00 |
| 402 | 395559 | 398732 | 1058 | 597.1 | + | 744781616 | carbamoyl phosphate synthase large subunit | S. hyicus | 0E+00 |
| 403 | 398744 | 399508 | 255 | 112.9 | + | 744781615 | dihydroorotate dehydrogenase electron transfer subunit | S. hyicus | 3E-171 |
| 404 | 399505 | 400422 | 306 | 196.2 | + | 744781614 | dihydroorotate dehydrogenase catalytic subunit | S. hyicus | 0E+00 |
| 405 | 400427 | 401119 | 231 | 128.1 | + | 744781613 | orotidine 5'-phosphate decarboxylase | S. hyicus | 8E-143 |
| 406 | 401119 | 401727 | 203 | 113.8 | + | 744781612 | orotate phosphoribosyltransferase | S. hyicus | 1E-129 |
| 407 | 402008 | 402097 | 30 | 3.7 | - |  | No Significant Match |  |  |
| 408 | 402275 | 402688 | 138 | 78.1 | + | 744781611 | 3-demethylubiquinone-9 3-methyltransferase | S. hyicus | 2E-94 |
| 409 | 403042 | 404754 | 571 | 281 | - | 744781610 | fibronectin-binding protein | S. hyicus | 0E+00 |
| 410 | 404915 | 405538 | 208 | 116.4 | + | 744781609 | guanylate kinase | S. hyicus | 5E-148 |
| 411 | 405538 | 405747 | 70 | 51.9 | + | 744781608 | DNA-directed RNA polymerase subunit omega | S. hyicus | 6E-44 |
| 412 | 406030 | 407247 | 406 | 227.3 | + | 744781607 | phosphopantothenoylcysteine decarboxylase/phosphopantothenate--cysteine ligase | S. hyicus | 0E+00 |
| 413 | 407244 | 409655 | 804 | 367.2 | + | 746620364 | primosomal protein N' | S. hyicus | 0E+00 |
| 414 | 409853 | 410341 | 163 | 104.1 | + | 744781604 | peptide deformylase | S. hyicus | 4E-105 |
| 415 | 410334 | 411266 | 311 | 191.8 | + | 744781603 | methionyl-tRNA formyltransferase | S. hyicus | 0E+00 |
| 416 | 411267 | 412574 | 436 | 175.5 | + | 744781602 | ribosomal RNA small subunit methyltransferase B | S. hyicus | 0E+00 |
| 417 | 412574 | 413668 | 365 | 183.7 | + | 744781601 | ribosomal RNA large subunit methyltransferase N | S. hyicus | 0E+00 |
| 418 | 413674 | 414417 | 248 | 144.5 | + | 744781600 | protein phosphatase | S. hyicus | 2E-171 |
| 419 | 414414 | 416390 | 659 | 352.6 | + | 744781599 | serine/threonine-protein kinase PrkC | S. hyicus | 0E+00 |
| 420 | 416692 | 417969 | 426 | 239.6 | + | 744781598 | cytochrome P450 | S. hyicus | 0E+00 |
| 421 | 418264 | 419139 | 292 | 104.5 | + | 744781597 | ribosome small subunit-dependent GTPase A | S. hyicus | 0E+00 |
| 422 | 419139 | 419789 | 217 | 117.3 | + | 744781596 | ribulose-phosphate 3-epimerase | S. hyicus | 3E-142 |
| 423 | 419793 | 420428 | 212 | 111.5 | + | 744781595 | thiamine pyrophosphokinase | S. hyicus | 7E-122 |
| 424 | 420509 | 420697 | 63 | 25.9 | - | 744781594 | 50S ribosomal protein L28 | S. hyicus | 1E-37 |
| 425 | 421138 | 421512 | 125 | 68.7 | + | 744781593 | alkaline shock protein | S. hyicus | 2E-82 |
| 426 | 421525 | 423183 | 553 | 353.4 | + | 744781592 | fatty acid kinase subunit FakA | S. hyicus | 0E+00 |
| 427 | 423342 | 424010 | 223 | 139.5 | + | 744781591 | serine dehydratase subunit beta | S. hyicus | 8E-157 |
| 428 | 424028 | 424897 | 290 | 197.9 | + | 744781590 | serine dehydratase | S. hyicus | 0E+00 |
| 429 | 424845 | 426944 | 700 | 290.4 | + | 744781589 | ATP-dependent DNA helicase RecG | S. hyicus | 0E+00 |
| 430 | 427203 | 427763 | 187 | 85.8 | + | 744781588 | fatty acid biosynthesis transcriptional regulator FapR | S. hyicus | 3E-130 |
| 431 | 427765 | 428751 | 329 | 222 | + | 744781587 | phosphate acyltransferase | S. hyicus | 0E+00 |
| 432 | 428744 | 429670 | 309 | 194.1 | + | 744781586 | malonyl CoA-ACP transacylase | S. hyicus | 0E+00 |
| 433 | 429663 | 430400 | 246 | 160 | + | 744781585 | 3-ketoacyl-ACP reductase | S. hyicus | 2E-175 |
| 434 | 430717 | 430950 | 78 | 54.5 | + | 744781584 | acyl carrier protein | S. hyicus | 2E-45 |
| 435 | 431062 | 431793 | 244 | 140.8 | + | 744781583 | ribonuclease III | S. hyicus | 5E-174 |
| 436 | 431797 | 435363 | 1189 | 749.3 | + | 744781582 | chromosome segregation protein SMC | S. hyicus | 0E+00 |
| 437 | 435364 | 436695 | 444 | 304 | + | 744781581 | signal recognition particle-docking protein FtsY | S. hyicus | 0E+00 |
| 438 | 436682 | 437014 | 111 | 46.4 | + | 744781580 | DNA-binding protein | S. hyicus | 9E-68 |
| 439 | 437028 | 438395 | 456 | 293.4 | + | 744781579 | signal recognition particle protein | S. hyicus | 0E+00 |
| 440 | 438621 | 438896 | 92 | 64.7 | + | 744781578 | 30S ribosomal protein S16 | S. hyicus | 1E-60 |
| 441 | 439001 | 439504 | 168 | 107 | + | 744781577 | 16S rRNA processing protein RimM | S. hyicus | 5E-112 |
| 442 | 439501 | 440235 | 245 | 128.5 | + | 744781576 | tRNA (guanine-N1)-methyltransferase | S. hyicus | 8E-171 |
| 443 | 440340 | 440690 | 117 | 76.3 | + | 744781575 | 50S ribosomal protein L19 | S. hyicus | 2E-75 |
| 444 | 441033 | 441656 | 208 | 33.4 | + | 744781574 | PAP2 family protein | S. hyicus | 1E-105 |
| 445 | 441797 | 442186 | 130 | 10.9 | + |  | No Significant Match |  |  |
| 446 | 442176 | 443417 | 414 | 189.7 | + | Q53654 | Collagen adhesin | S. aureus | 1E-12 |
| 447 | 443527 | 446121 | 865 | 264.4 | - | 744781573 | YfhO family membrane protein | S. hyicus | 0E+00 |
| 448 | 446118 | 448721 | 868 | 280.4 | - | 744781572 | YfhO family membrane protein | S. hyicus | 0E+00 |
| 449 | 448740 | 449120 | 127 | 23.2 | - | 744781571 | hypothetical protein SHYC_08190 | S. hyicus | 2E-79 |
| 450 | 449328 | 450212 | 295 | 157.1 | + | 744781570 | GTPase | S. hyicus | 0E+00 |
| 451 | 450202 | 450978 | 259 | 108 | + | 744781569 | ribonuclease HII | S. hyicus | 8E-149 |
| 452 | 451065 | 451334 | 90 | 50.5 | + | 744781568 | hypothetical protein SHYC_08175 | S. hyicus | 3E-52 |
| 453 | 451547 | 452713 | 389 | 276.1 | + | 744781567 | succinyl-CoA synthetase subunit beta | S. hyicus | 0E+00 |
| 454 | 452735 | 453643 | 303 | 219.9 | + | 744781566 | succinyl-CoA synthetase subsunit alpha | S. hyicus | 0E+00 |
| 455 | 453856 | 454737 | 294 | 101.7 | + | 744781565 | putative DNA processing factor DprA(SMF) | S. hyicus | 3E-156 |
| 456 | 454832 | 456901 | 690 | 361 | + | 744781564 | DNA topoisomerase I | S. hyicus | 0E+00 |
| 457 | 456914 | 458218 | 435 | 260.4 | + | 744781563 | tRNA (uracil-5-)-methyltransferase | S. hyicus | 0E+00 |
| 458 | 458339 | 459229 | 297 | 104.7 | + | 744781562 | tyrosine recombinase XerC | S. hyicus | 0E+00 |
| 459 | 459233 | 459775 | 181 | 109.6 | + | 744781561 | ATP-dependent protease subunit HslV | S. hyicus | 1E-128 |
| 460 | 459792 | 461201 | 470 | 293.2 | + | 744781560 | ATP-dependent protease ATPase subunit HslU | S. hyicus | 0E+00 |
| 461 | 461216 | 461989 | 258 | 162.9 | + | 744781559 | GTP-sensing transcriptional pleiotropic repressor CodY | S. hyicus | 0E+00 |
| 462 | 462151 | 462927 | 259 | 181.2 | + | 744781558 | 30S ribosomal protein S2 | S. hyicus | 0E+00 |
| 463 | 463101 | 463979 | 293 | 222.6 | + | 744781557 | translation elongation factor Ts | S. hyicus | 0E+00 |
| 464 | 464133 | 464855 | 241 | 157.9 | + | 744781556 | uridylate kinase | S. hyicus | 4E-175 |
| 465 | 464874 | 465428 | 185 | 136.8 | + | 744781555 | ribosome recycling factor | S. hyicus | 3E-126 |
| 466 | 465578 | 466339 | 254 | 135.6 | + | 744781554 | undecaprenyl diphosphate synthase | S. hyicus | 4E-177 |
| 467 | 466353 | 467132 | 260 | 118 | + | 744781553 | phosphatidate cytidylyltransferase | S. hyicus | 3E-180 |
| 468 | 467148 | 468278 | 377 | 205.9 | + | 744781552 | 1-deoxy-D-xylulose 5-phosphate reductoisomerase | S. hyicus | 0E+00 |
| 469 | 468285 | 469565 | 427 | 217.9 | + | 744781551 | membrane-associated zinc metalloprotease | S. hyicus | 0E+00 |
| 470 | 469594 | 471300 | 569 | 342.8 | + | 744781550 | prolyl-tRNA synthetase | S. hyicus | 0E+00 |
| 471 | 471499 | 475809 | 1437 | 785.4 | + | 744781549 | DNA polymerase III PolC type | S. hyicus | 0E+00 |
| 472 | 475937 | 476404 | 156 | 101.7 | + | 744781548 | ribosome maturation protein RimP | S. hyicus | 6E-105 |
| 473 | 476424 | 477602 | 393 | 280 | + | 744781547 | transcription termination factor NusA | S. hyicus | 0E+00 |
| 474 | 477614 | 477901 | 96 | 62.1 | + | 744781546 | YlxR family nucleic-acid-binding protein | S. hyicus | 9E-61 |
| 475 | 477898 | 478200 | 101 | 37 | + | 744781545 | 50S ribosomal protein L7Ae | S. hyicus | 6E-61 |
| 476 | 478217 | 480349 | 711 | 496.7 | + | 744781544 | translation initiation factor IF-2 | S. hyicus | 0E+00 |
| 477 | 480547 | 480930 | 128 | 61.9 | + | 744781543 | glyoxalase/bleomycin resistance family protein | S. hyicus | 6E-72 |
| 478 | 481028 | 481372 | 115 | 72.5 | + | 744781542 | ribosome-binding factor A | S. hyicus | 2E-77 |
| 479 | 481446 | 482363 | 306 | 124.3 | + | 744781541 | tRNA pseudouridine synthase B | S. hyicus | 9E-169 |
| 480 | 482378 | 483349 | 324 | 184.9 | + | 744781540 | riboflavin biosynthesis protein RibF | S. hyicus | 0E+00 |
| 481 | 483453 | 483722 | 90 | 70.3 | + | 744781539 | 30S ribosomal protein S15 | S. hyicus | 7E-56 |
| 482 | 483891 | 485993 | 701 | 496.4 | + | 744781538 | polyribonucleotide phosphorylase | S. hyicus | 0E+00 |
| 483 | 486363 | 487679 | 439 | 227.5 | + | Q08002 | Neutral metalloprotease | S. hyicus | 0E+00 |
| 484 | 488114 | 489787 | 558 | 325.5 | + | 744781536 | mRNA degradation ribonuclease | S. hyicus | 0E+00 |
| 485 | 489987 | 492335 | 783 | 416.2 | + | 744781535 | cell division protein FtsK | S. hyicus | 0E+00 |
| 486 | 492338 | 493054 | 239 | 138.1 | + | 744781534 | transcriptional regulator | S. hyicus | 4E-173 |
| 487 | 493085 | 494371 | 429 | 203.2 | + | 744781533 | zinc protease | S. hyicus | 0E+00 |
| 488 | 494352 | 495635 | 428 | 203.9 | + | 744781532 | zinc protease | S. hyicus | 0E+00 |
| 489 | 495638 | 496342 | 235 | 109.4 | + | 744781531 | 3-oxoacyl-ACP reductase | S. hyicus | 6E-150 |
| 490 | 496394 | 497266 | 291 | 157.4 | + | 744781530 | hypothetical protein SHYC_07980 | S. hyicus | 0E+00 |
| 491 | 497279 | 497674 | 132 | 46.7 | + | 744781529 | transcriptional regulator | S. hyicus | 2E-73 |
| 492 | 497718 | 498302 | 195 | 104.1 | + | 744781528 | CDP-diacylglycerol--glycerol-3-phosphate 3-phosphatidyltransferase | S. hyicus | 1E-127 |
| 493 | 498402 | 499547 | 382 | 218.6 | + | 744781527 | damage-inducible protein CinA | S. hyicus | 0E+00 |
| 494 | 499715 | 500758 | 348 | 240 | + | 744781526 | recombinase RecA | S. hyicus | 0E+00 |
| 495 | 501195 | 502754 | 520 | 332.1 | + | 744781525 | ribonuclease Y | S. hyicus | 0E+00 |
| 496 | 502997 | 503215 | 73 | 32.1 | - | 744781524 | hypothetical protein SHYC_07950 | S. hyicus | 7E-45 |
| 497 | 503404 | 503787 | 128 | 48.8 | + | 744781523 | cadmium efflux system accessory protein | S. hyicus | 2E-86 |
| 498 | 503768 | 505918 | 717 | 336 | + | 744781522 | putative cadmium transporter | S. hyicus | 0E+00 |
| 499 | 506052 | 506843 | 264 | 150.6 | + | 744781521 | phosphoesterase | S. hyicus | 0E+00 |
| 500 | 506956 | 508716 | 587 | 371.3 | + | 744781520 | 2-oxoglutarate ferredoxin oxidoreductase subunit alpha | S. hyicus | 0E+00 |
| 501 | 508717 | 509583 | 289 | 153.3 | + | 744781519 | 2-oxoacid ferredoxin oxidoreductase subunit beta | S. hyicus | 0E+00 |
| 502 | 509654 | 509950 | 99 | 56.9 | + | 744781518 | hypothetical protein SHYC_07920 | S. hyicus | 1E-56 |
| 503 | 510060 | 511601 | 514 | 306.4 | + | 744781517 | (dimethylallyl)adenosine tRNA methylthiotransferase | S. hyicus | 0E+00 |
| 504 | 511601 | 511981 | 127 | 48.8 | + | 744781516 | membrane protein | S. hyicus | 3E-60 |
| 505 | 511986 | 512495 | 170 | 62.4 | + | 744781515 | thiamine biosynthesis protein ThiW | S. hyicus | 5E-103 |
| 506 | 512730 | 515342 | 871 | 448.6 | + | 744781514 | DNA mismatch repair protein MutS | S. hyicus | 0E+00 |
| 507 | 515355 | 517304 | 650 | 329 | + | 744781513 | DNA mismatch repair protein MutL | S. hyicus | 0E+00 |
| 508 | 517315 | 517848 | 178 | 74.8 | + | 744781512 | glycerol uptake operon antiterminator regulatory protein GlpP | S. hyicus | 3E-114 |
| 509 | 517873 | 518904 | 344 | 159.7 | + | 744781511 | LD-carboxypeptidase family protein | S. hyicus | 0E+00 |
| 510 | 519111 | 519926 | 272 | 164.1 | + | 744781510 | glycerol uptake facilitator protein | S. hyicus | 0E+00 |
| 511 | 519995 | 521497 | 501 | 313 | + | Q6GHD5 | Glycerol kinase | S. aureus | 0E+00 |
| 512 | 521621 | 523294 | 558 | 372.7 | + | 744781509 | glycerol-3-phosphate dehydrogenase | S. hyicus | 0E+00 |
| 513 | 523579 | 524511 | 311 | 145.2 | + | 744781508 | putative lysophospholipase | S. hyicus | 0E+00 |
| 514 | 524517 | 525473 | 319 | 138.3 | + | 744781507 | tRNA delta(2)-isopentenylpyrophosphate transferase | S. hyicus | 0E+00 |
| 515 | 525466 | 525672 | 69 | 46.9 | + | 744781506 | RNA-binding protein Hfq | S. hyicus | 3E-43 |
| 516 | 525810 | 526283 | 158 | 59.7 | - | 744781505 | glutathione peroxidase | S. hyicus | 7E-103 |
| 517 | 526419 | 527648 | 410 | 187.2 | + | 744781504 | GTP-binding protein HflX | S. hyicus | 0E+00 |
| 518 | 527666 | 528904 | 413 | 192.4 | + | 744781503 | putative C-S lyase | S. hyicus | 0E+00 |
| 519 | 529019 | 529384 | 122 | 57.5 | + | 744781502 | HTH-type transcriptional regulator GlnR | S. hyicus | 1E-72 |
| 520 | 529406 | 530746 | 447 | 267 | + | 744781501 | glutamine synthetase type I | S. hyicus | 0E+00 |
| 521 | 530860 | 531084 | 75 | 8.5 | - |  | No Significant Match |  |  |
| 522 | 531250 | 531525 | 92 | 24.8 | + |  | No Significant Match |  |  |
| 523 | 531720 | 532175 | 152 | 65.2 | - | 744781497 | lipoprotein | S. hyicus | 1E-60 |
| 524 | 532446 | 533063 | 206 | 80.4 | + | 744781496 | ABC transporter ATP-binding protein | S. hyicus | 3E-57 |
| 525 | 533089 | 533829 | 247 | 67.8 | + | 744781495 | ABC transporter permease | S. hyicus | 1E-55 |
| 526 | 533958 | 534920 | 321 | 93.5 | + | 744781494 | two-component sensor histidine kinase | S. hyicus | 2E-104 |
| 527 | 534917 | 535519 | 201 | 110.7 | + | 744781493 | LuxR family transcriptional regulator | S. hyicus | 1E-109 |
| 528 | 535568 | 535774 | 69 | 28.5 | - | 744781492 | membrane protein | S. hyicus | 2E-23 |
| 529 | 535918 | 536427 | 170 | 95.2 | + | 226490494 | thermonuclease | S. hyicus | 2E-120 |
| 530 | 536462 | 537301 | 280 | 83.4 | - | 744781490 | hypothetical protein SHYC_07775 | S. hyicus | 3E-132 |
| 531 | 537482 | 537847 | 122 | 57.7 | - | 744781489 | staphylococcal accessory regulator family protein | S. hyicus | 6E-78 |
| 532 | 538412 | 539002 | 197 | 65.6 | + | 744781488 | hypothetical protein SHYC_07765 | S. hyicus | 1E-134 |
| 533 | 539106 | 540455 | 450 | 193.2 | - | 744781487 | aspartate kinase | S. hyicus | 0E+00 |
| 534 | 540645 | 541928 | 428 | 272.3 | + | 744781486 | homoserine dehydrogenase | S. hyicus | 0E+00 |
| 535 | 541928 | 542998 | 357 | 225.8 | + | 744781485 | threonine synthase | S. hyicus | 0E+00 |
| 536 | 542995 | 543915 | 307 | 160.4 | + | 744781484 | homoserine kinase | S. hyicus | 0E+00 |
| 537 | 544119 | 544934 | 272 | 162.5 | + | 744781483 | HAD family hydrolase | S. hyicus | 9E-180 |
| 538 | 545215 | 545529 | 105 | 52.7 | - | 744781482 | hypothetical protein SHYC_07735 | S. hyicus | 1E-69 |
| 539 | 545666 | 547126 | 487 | 215.7 | - | 744781481 | amino acid permease | S. hyicus | 0E+00 |
| 540 | 547329 | 548825 | 499 | 305.6 | + | 744781480 | catalase | S. hyicus | 0E+00 |
| 541 | 549181 | 549330 | 50 | 17.9 | - | 744781479 | 50S ribosomal protein L33 | S. hyicus | 8E-29 |
| 542 | 549516 | 549785 | 90 | 60.6 | + | 744781478 | 30S ribosomal protein S14 | S. hyicus | 9E-58 |
| 543 | 549860 | 550837 | 326 | 190.8 | + | 744781477 | guanosine 5'-monophosphate oxidoreductase | S. hyicus | 0E+00 |
| 544 | 550942 | 551865 | 308 | 126.1 | + | 744781476 | secretion protein | S. hyicus | 0E+00 |
| 545 | 551918 | 552022 | 35 | 12.4 | + |  | No Significant Match |  |  |
| 546 | 552586 | 553233 | 216 | 31.9 | + |  | No Significant Match |  |  |
| 547 | 553305 | 553940 | 212 | 40.9 | + |  | No Significant Match |  |  |
| 548 | 554064 | 554711 | 216 | 42.1 | + |  | No Significant Match |  |  |
| 549 | 554775 | 555086 | 104 | 71.3 | + |  | No Significant Match |  |  |
| 550 | 555160 | 555783 | 208 | 122.2 | - | 744781475 | LexA repressor | S. hyicus | 2E-148 |
| 551 | 555927 | 556151 | 75 | 34.6 | + | 744781474 | hypothetical protein SHYC_07685 | S. hyicus | 3E-43 |
| 552 | 556366 | 556602 | 79 | 49 | + | 744781473 | hypothetical protein SHYC_07680 | S. hyicus | 3E-47 |
| 553 | 556691 | 558679 | 663 | 427.6 | + | 744781472 | transketolase | S. hyicus | 0E+00 |
| 554 | 558866 | 559105 | 80 | 29.2 | + | 744781471 | hypothetical protein SHYC_07670 | S. hyicus | 2E-49 |
| 555 | 559268 | 559741 | 158 | 68.8 | + | 744781470 | cytochrome C biogenesis protein CcdC | S. hyicus | 2E-103 |
| 556 | 559872 | 560990 | 373 | 147.9 | + | 744781469 | exonuclease SbcCD subunit D | S. hyicus | 0E+00 |
| 557 | 560993 | 564025 | 1011 | 521.5 | + | 744781468 | exonuclease SbcCD subunit C | S. hyicus | 0E+00 |
| 558 | 564100 | 564465 | 122 | 70.9 | - | 744781467 | mechanosensitive ion channel protein MscL | S. hyicus | 5E-56 |
| 559 | 564701 | 566335 | 545 | 257.8 | + | 744781466 | glycine betaine transporter | S. hyicus | 0E+00 |
| 560 | 566679 | 569378 | 900 | 593.1 | + | 744781465 | aconitate hydratase | S. hyicus | 0E+00 |
| 561 | 569520 | 569975 | 152 | 85.5 | + | 744781464 | thioesterase family protein | S. hyicus | 2E-99 |
| 562 | 570308 | 570607 | 100 | 70.7 | - | 744781463 | hypothetical protein SHYC_07630 | S. hyicus | 1E-61 |
| 563 | 570687 | 571274 | 196 | 65.5 | - | 744781462 | glycerol-3-phosphate acyltransferase | S. hyicus | 6E-127 |
| 564 | 571481 | 573475 | 665 | 387.5 | + | 744781461 | DNA topoisomerase IV subunit B | S. hyicus | 0E+00 |
| 565 | 573475 | 575886 | 804 | 455.7 | + | 744781460 | DNA gyrase subunit A | S. hyicus | 0E+00 |
| 566 | 575992 | 577461 | 490 | 249.7 | + | 744781459 | sodium:alanine symporter | S. hyicus | 0E+00 |
| 567 | 577637 | 578488 | 284 | 132.2 | + | 744781458 | ptsG operon antiterminator GlcT | S. hyicus | 0E+00 |
| 568 | 578597 | 580627 | 677 | 398.3 | + | 744781457 | PTS system glucose-specific transporter subunit IICBA | S. hyicus | 0E+00 |
| 569 | 581115 | 582341 | 409 | 190.9 | + | 744781456 | membrane protein | S. hyicus | 0E+00 |
| 570 | 582491 | 585010 | 840 | 338 | + | 744781455 | phosphatidylglycerol lysyltransferase | S. hyicus | 0E+00 |
| 571 | 585052 | 585564 | 171 | 69.2 | - | 744781454 | peptide methionine sulfoxide reductase MsrA | S. hyicus | 5E-120 |
| 572 | 585708 | 586679 | 324 | 186.5 | + | 744781453 | regulatory protein MsrR | S. hyicus | 0E+00 |
| 573 | 586839 | 587024 | 62 | 48.2 | - | 744781452 | 4-oxalocrotonate tautomerase | S. hyicus | 1E-34 |
| 574 | 587173 | 587583 | 137 | 84.2 | + | 744781451 | hypothetical protein SHYC_07570 | S. hyicus | 1E-87 |
| 575 | 587780 | 589000 | 407 | 163.4 | + | 744781449 | DNA repair protein | S. hyicus | 0E+00 |
| 576 | 589131 | 590225 | 365 | 188 | - | 744781448 | prephenate dehydrogenase | S. hyicus | 0E+00 |
| 577 | 590344 | 591402 | 353 | 162.7 | + | 744781447 | deblocking aminopeptidase, M42 family | S. hyicus | 0E+00 |
| 578 | 591760 | 593019 | 420 | 225.1 | + | 744781446 | aminoacyltransferase FemA | S. hyicus | 0E+00 |
| 579 | 593044 | 594303 | 420 | 229.7 | + | 744781445 | aminoacyltransferase FemB | S. hyicus | 0E+00 |
| 580 | 594666 | 594989 | 108 | 33.9 | - | 744781444 | membrane protein | S. hyicus | 3E-57 |
| 581 | 595120 | 596922 | 601 | 313.1 | + | 744781443 | oligoendopeptidase F | S. hyicus | 0E+00 |
| 582 | 597064 | 598008 | 315 | 158.3 | + | 744781442 | tagatose-6-phosphate kinase | S. hyicus | 0E+00 |
| 583 | 598104 | 599012 | 303 | 171.3 | - | 744781441 | RNA-binding protein | S. hyicus | 0E+00 |
| 584 | 599169 | 600776 | 536 | 330.7 | + | 744781440 | ABC transporter ATP-binding protein | S. hyicus | 0E+00 |
| 585 | 601219 | 602202 | 328 | 175.4 | + | 744781439 | aspartate-semialdehyde dehydrogenase | S. hyicus | 0E+00 |
| 586 | 602226 | 603104 | 293 | 176.2 | + | 744781438 | dihydrodipicolinate synthase | S. hyicus | 0E+00 |
| 587 | 603101 | 603823 | 241 | 111.1 | + | 744781437 | dihydrodipicolinate reductase | S. hyicus | 1E-150 |
| 588 | 603840 | 604559 | 240 | 164.2 | + | 744781436 | 2,3,4,5-tetrahydropyridine-2,6-carboxylate N-succinyltransferase | S. hyicus | 8E-161 |
| 589 | 604698 | 605846 | 383 | 185.8 | + | 744781435 | amidohydrolase family protein | S. hyicus | 0E+00 |
| 590 | 605849 | 606922 | 358 | 147.3 | + | 744781434 | alanine racemase 2 | S. hyicus | 0E+00 |
| 591 | 606919 | 608184 | 422 | 207 | + | 744781433 | diaminopimelate decarboxylase | S. hyicus | 0E+00 |
| 592 | 608425 | 608625 | 67 | 43.3 | - | Q49XK3 | Cold shock protein CspA | S. saprophyticus | 3E-40 |
| 593 | 608804 | 609112 | 103 | 24.8 | - | 744781430 | hypothetical protein SHYC_07465 | S. hyicus | 9E-68 |
| 594 | 609503 | 609772 | 90 | 53.4 | + | 744781429 | acylphosphatase | S. hyicus | 1E-48 |
| 595 | 609795 | 610430 | 212 | 81.8 | + | 744781428 | 5-bromo-4-chloroindolyl phosphate hydrolase | S. hyicus | 3E-144 |
| 596 | 610427 | 611620 | 398 | 251.2 | + | 744781427 | tellurite resistance protein TelA | S. hyicus | 0E+00 |
| 597 | 611678 | 613024 | 449 | 214.2 | - | 744781426 | branched-chain amino acid ABC transporter substrate-binding protein | S. hyicus | 0E+00 |
| 598 | 613144 | 615033 | 630 | 315 | - | 744781425 | NorD family protein | S. hyicus | 0E+00 |
| 599 | 615045 | 615839 | 265 | 138.8 | - | 744781424 | hypothetical protein SHYC_07435 | S. hyicus | 1E-180 |
| 600 | 615914 | 616117 | 68 | 28.6 | - | 744781423 | hypothetical protein SHYC_07430 | S. hyicus | 3E-39 |
| 601 | 616129 | 616944 | 272 | 148.6 | - | 744781422 | putative ring-cleaving dioxygenase | S. hyicus | 1E-163 |
| 602 | 617254 | 618495 | 414 | 280.2 | - | 744781421 | 2-oxoglutarate dehydrogenase, E2 component | S. hyicus | 0E+00 |
| 603 | 618509 | 621298 | 930 | 505.1 | - | 744781420 | 2-oxoglutarate dehydrogenase E1 component | S. hyicus | 0E+00 |
| 604 | 621390 | 622760 | 457 | 184.4 | - | 744781419 | histidine kinase | S. hyicus | 0E+00 |
| 605 | 622757 | 623434 | 226 | 123.9 | - | 744781418 | two-component response regulator ArlR | S. hyicus | 7E-154 |
| 606 | 624012 | 624626 | 205 | 67.4 | - | 744781417 | PAP2 family phosphatase | S. hyicus | 2E-142 |
| 607 | 624638 | 625711 | 358 | 200.2 | - | 744781416 | UDP-diphospho-muramoylpentapeptide beta-N- acetylglucosaminyltransferase | S. hyicus | 0E+00 |
| 608 | 625721 | 626230 | 170 | 85.3 | - | 744781415 | acetyltransferase | S. hyicus | 3E-105 |
| 609 | 626227 | 627669 | 481 | 266.2 | - | 744781414 | carboxy-terminal processing proteinase CtpA | S. hyicus | 0E+00 |
| 610 | 627838 | 628059 | 74 | 30.6 | - | 744781413 | hypothetical protein SHYC_07380 | S. hyicus | 5E-45 |
| 611 | 628059 | 628562 | 168 | 115.6 | - | 744781412 | PTS glucose transporter subunit IIA | S. hyicus | 3E-105 |
| 612 | 628573 | 629001 | 143 | 81.7 | - | 744781411 | peptide methionine sulfoxide reductase B | S. hyicus | 2E-98 |
| 613 | 628994 | 629521 | 176 | 65.5 | - | 744781410 | peptide methionine sulfoxide reductase MsrA | S. hyicus | 8E-123 |
| 614 | 629536 | 630384 | 283 | 164.3 | - | 744781409 | DegV family protein FakB2 | S. hyicus | 0E+00 |
| 615 | 630402 | 630878 | 159 | 76.7 | - | 744781408 | dihydrofolate reductase | S. hyicus | 2E-107 |
| 616 | 630949 | 631902 | 318 | 131.9 | - | 744781407 | thymidylate synthase | S. hyicus | 0E+00 |
| 617 | 632044 | 632484 | 147 | 79.8 | - | 744781406 | disulfide isomerase | S. hyicus | 2E-95 |
| 618 | 632497 | 633612 | 372 | 194.8 | - | 744781405 | virulence factor | S. hyicus | 0E+00 |
| 619 | 633624 | 633809 | 62 | 6.9 | - | 744781404 | hypothetical protein SHYC_07335 | S. hyicus | 1E-35 |
| 620 | 634103 | 634807 | 235 | 85 | + | 744781403 | YhhQ family membrane protein | S. hyicus | 1E-154 |
| 621 | 634895 | 635296 | 134 | 54.1 | + | 744781402 | ribonuclease H-like protein | S. hyicus | 7E-77 |
| 622 | 635483 | 636367 | 295 | 135.8 | - | 744781401 | 5'-3' exonuclease | S. hyicus | 0E+00 |
| 623 | 636360 | 639821 | 1154 | 520.4 | - | 744781400 | hypothetical protein SHYC_07315 | S. hyicus | 0E+00 |
| 624 | 640186 | 641100 | 305 | 102.8 | - | 744781399 | permease RarD | S. hyicus | 0E+00 |
| 625 | 641244 | 642377 | 378 | 234.3 | - | 744781398 | ribosomal RNA large subunit methyltransferase L | S. hyicus | 0E+00 |
| 626 | 642856 | 642948 | 31 | 3.1 | - |  | No Significant Match |  |  |
| 627 | 643030 | 643365 | 112 | 76.1 | - | 744781397 | cell division protein GpsB | S. hyicus | 4E-73 |
| 628 | 643387 | 643944 | 186 | 87.1 | - | 744781396 | hypothetical protein SHYC_07295 | S. hyicus | 6E-122 |
| 629 | 643937 | 644272 | 112 | 42.8 | - | 744781395 | hypothetical protein SHYC_07290 | S. hyicus | 1E-48 |
| 630 | 644645 | 645211 | 189 | 81.8 | + | 744781394 | Holliday junction resolvase | S. hyicus | 1E-125 |
| 631 | 645208 | 647427 | 740 | 392.9 | + | 744781393 | transglycosylase | S. hyicus | 0E+00 |
| 632 | 647516 | 647848 | 111 | 59.2 | - | 744781392 | hypothetical protein SHYC_07275 | S. hyicus | 9E-53 |
| 633 | 647845 | 648516 | 224 | 103.3 | - | 744781391 | endonuclease III | S. hyicus | 1E-158 |
| 634 | 648506 | 649198 | 231 | 113.5 | - | 744781390 | chromosome replication protein DnaD | S. hyicus | 1E-152 |
| 635 | 649279 | 650571 | 431 | 259.3 | - | 744781389 | asparagine--tRNA ligase | S. hyicus | 0E+00 |
| 636 | 650670 | 653357 | 896 | 379.4 | - | 744781388 | DnaQ family exonuclease/DinG family helicase | S. hyicus | 0E+00 |
| 637 | 653380 | 654351 | 324 | 115.2 | - | 744781387 | bifunctional repressor/biotin--acetyl-CoA-carboxylase ligase | S. hyicus | 0E+00 |
| 638 | 654338 | 655540 | 401 | 196 | - | 744781386 | tRNA CCA-pyrophosphorylase | S. hyicus | 0E+00 |
| 639 | 655533 | 656669 | 379 | 198.9 | - | 744781385 | N-acetyl-alpha-D-glucosaminyl L-malate synthase | S. hyicus | 0E+00 |
| 640 | 656684 | 657004 | 107 | 64.2 | - | 744781384 | nucleoside triphosphate pyrophosphohydrolase | S. hyicus | 4E-67 |
| 641 | 657255 | 658811 | 519 | 152.5 | + |  | No Significant Match |  |  |
| 642 | 658929 | 659624 | 232 | 125.9 | - | 744781383 | zinc metallopeptidase | S. hyicus | 4E-166 |
| 643 | 659661 | 660263 | 201 | 36.1 | - | 744781382 | membrane protein | S. hyicus | 2E-92 |
| 644 | 660294 | 660827 | 178 | 64.8 | - | 744781381 | hypothetical protein SHYC_07220 | S. hyicus | 2E-87 |
| 645 | 660837 | 662078 | 414 | 252.7 | - | 744781380 | TPR-repeat-containing protein | S. hyicus | 0E+00 |
| 646 | 662080 | 663369 | 430 | 165.8 | - | 744781379 | 3-phosphoshikimate 1-carboxyvinyltransferase | S. hyicus | 0E+00 |
| 647 | 663369 | 664433 | 355 | 159.4 | - | 744781378 | 3-dehydroquinate synthase | S. hyicus | 0E+00 |
| 648 | 664445 | 665611 | 389 | 225.5 | - | 744781377 | chorismate synthase | S. hyicus | 0E+00 |
| 649 | 665863 | 666312 | 150 | 94.5 | - | 744781376 | nucleoside diphosphate kinase | S. hyicus | 9E-102 |
| 650 | 666394 | 667350 | 319 | 171.5 | - | 744781375 | heptaprenyl diphosphate synthase component 2 | S. hyicus | 0E+00 |
| 651 | 667368 | 668078 | 237 | 109.6 | - | 744781374 | ubiquinone biosynthesis methyltransferase UbiE | S. hyicus | 1E-172 |
| 652 | 668079 | 668657 | 193 | 68.8 | - | 744781373 | heptaprenyl diphosphate synthase subunit 1 | S. hyicus | 4E-104 |
| 653 | 668876 | 669148 | 91 | 75.5 | - | 744781372 | DNA-binding protein HU | S. hyicus | 3E-57 |
| 654 | 669435 | 670433 | 333 | 190.3 | - | 744781371 | glycerol-3-phosphate dehydrogenase | S. hyicus | 0E+00 |
| 655 | 670450 | 671760 | 437 | 244.3 | - | 744781370 | GTP-binding protein Der | S. hyicus | 0E+00 |
| 656 | 671995 | 673182 | 396 | 258.9 | - | 744781369 | 30S ribosomal protein S1 | S. hyicus | 0E+00 |
| 657 | 673410 | 674084 | 225 | 142 | - | 744781368 | cytidylate kinase | S. hyicus | 7E-144 |
| 658 | 674157 | 675119 | 321 | 161.7 | + | 744781367 | asparaginase | S. hyicus | 0E+00 |
| 659 | 675162 | 676148 | 329 | 155.1 | - | 744781366 | pyridine nucleotide-disulfide oxidoreductase family protein | S. hyicus | 0E+00 |
| 660 | 676395 | 678338 | 648 | 338.3 | - | 744781365 | hypothetical protein SHYC_07140 | S. hyicus | 2E-74 |
| 661 | 678519 | 679895 | 459 | 179.2 | - | 744781364 | ATP-dependent DNA helicase RecQ-1 | S. hyicus | 0E+00 |
| 662 | 679885 | 680838 | 318 | 92.2 | - | 744781363 | hypothetical protein SHYC_07130 | S. hyicus | 1E-166 |
| 663 | 680944 | 681192 | 83 | 47.5 | + | 744781362 | ferredoxin | S. hyicus | 8E-51 |
| 664 | 681311 | 681859 | 183 | 73.8 | - | 744781361 | riboflavin transporter RibU | S. hyicus | 4E-113 |
| 665 | 681978 | 683720 | 581 | 283.4 | - | 744781360 | signal transduction histidine kinase SrrB | S. hyicus | 0E+00 |
| 666 | 683704 | 684429 | 242 | 159.1 | - | 744781359 | transcriptional regulatory protein SrrA | S. hyicus | 2E-173 |
| 667 | 684557 | 685297 | 247 | 134.9 | - | 744781358 | pseudouridine synthase | S. hyicus | 8E-172 |
| 668 | 685294 | 685839 | 182 | 114.8 | - | 744781357 | segregation and condensation protein B | S. hyicus | 1E-106 |
| 669 | 685823 | 686557 | 245 | 144.2 | - | 744781356 | segregation and condensation protein A | S. hyicus | 2E-157 |
| 670 | 686654 | 687157 | 168 | 63 | + | 744781355 | putative hydrolase | S. hyicus | 7E-92 |
| 671 | 687178 | 688065 | 296 | 120 | - | 744781354 | tyrosine recombinase XerD | S. hyicus | 0E+00 |
| 672 | 688102 | 688554 | 151 | 68.5 | - | 744781353 | ferric uptake regulation protein Fur | S. hyicus | 5E-104 |
| 673 | 688658 | 689200 | 181 | 110.8 | - | 744781352 | ADP-ribose pyrophosphatase | S. hyicus | 2E-107 |
| 674 | 689279 | 690187 | 303 | 165.2 | + | 744781351 | oxidoreductase | S. hyicus | 0E+00 |
| 675 | 690259 | 694314 | 1352 | 714.1 | - | Q53654 | Collagen adhesin | S. aureus | 0E+00 |
| 676 | 694657 | 695475 | 273 | 147 | + | 744781349 | pyrroline-5-carboxylate reductase | S. hyicus | 0E+00 |
| 677 | 695583 | 696500 | 306 | 163.1 | - | 744781348 | ribonuclease Z | S. hyicus | 0E+00 |
| 678 | 696661 | 698145 | 495 | 259.4 | + | 744781347 | glucose-6-phosphate 1-dehydrogenase | S. hyicus | 0E+00 |
| 679 | 698248 | 699105 | 286 | 123.5 | + | 744781346 | AraC family transcriptional regulator | S. hyicus | 0E+00 |
| 680 | 699153 | 700556 | 468 | 311.5 | - | 744781345 | 6-phosphogluconate dehydrogenase | S. hyicus | 0E+00 |
| 681 | 700633 | 701745 | 371 | 229.9 | - | 744781344 | peptidase T-like protein | S. hyicus | 0E+00 |
| 682 | 702067 | 703044 | 326 | 138.1 | - | 744781343 | membrane protein | S. hyicus | 0E+00 |
| 683 | 703056 | 703493 | 146 | 95.6 | - | 744781342 | disulfide isomerase | S. hyicus | 4E-101 |
| 684 | 703570 | 704850 | 427 | 231.5 | - | 744781341 | 2-oxoglutarate dehydrogenase subunit E2 | S. hyicus | 0E+00 |
| 685 | 704862 | 705845 | 328 | 215.4 | - | 744781340 | 2-oxoisovalerate dehydrogenase subunit beta | S. hyicus | 0E+00 |
| 686 | 705838 | 706836 | 333 | 201.6 | - | 744781339 | 2-oxoisovalerate dehydrogenase subunit alpha | S. hyicus | 0E+00 |
| 687 | 706860 | 708278 | 473 | 274.6 | - | 744781338 | dihydrolipoamide dehydrogenase | S. hyicus | 0E+00 |
| 688 | 708291 | 709349 | 353 | 176.2 | - | 744781337 | butyrate kinase | S. hyicus | 0E+00 |
| 689 | 709402 | 710310 | 303 | 111.2 | - | 744781336 | phosphate butyryltransferase | S. hyicus | 0E+00 |
| 690 | 710320 | 711993 | 558 | 302.1 | - | 744781335 | DNA recombination protein RecN | S. hyicus | 0E+00 |
| 691 | 712006 | 712458 | 151 | 84.3 | - | 744781334 | arginine repressor | S. hyicus | 3E-106 |
| 692 | 712720 | 714609 | 630 | 325.6 | - | 744781333 | 1-deoxy-D-xylulose-5-phosphate synthase | S. hyicus | 0E+00 |
| 693 | 714645 | 715529 | 295 | 171.1 | - | 744781332 | geranyl transferase | S. hyicus | 3E-164 |
| 694 | 715507 | 715731 | 75 | 37.7 | - | 744781331 | exodeoxyribonuclease VII small subunit | S. hyicus | 3E-37 |
| 695 | 715715 | 717061 | 449 | 192.1 | - | 744781330 | exodeoxyribonuclease VII large subunit | S. hyicus | 0E+00 |
| 696 | 717070 | 717462 | 131 | 67.9 | - | 744781329 | transcription antitermination factor NusB | S. hyicus | 3E-87 |
| 697 | 717481 | 717858 | 126 | 62.5 | - | 744781328 | hypothetical protein SHYC_06950 | S. hyicus | 4E-87 |
| 698 | 717880 | 719235 | 452 | 254.4 | - | 744781327 | acetyl-CoA carboxylase biotin carboxylase subunit | S. hyicus | 0E+00 |
| 699 | 719237 | 719692 | 152 | 107.2 | - | 744781326 | acetyl-CoA carboxylase, biotin carboxyl carrier protein | S. hyicus | 5E-96 |
| 700 | 719964 | 720521 | 186 | 118.3 | - | 744781325 | elongation factor P | S. hyicus | 4E-134 |
| 701 | 720550 | 721611 | 354 | 185.3 | - | 744781324 | peptidase M24 | S. hyicus | 0E+00 |
| 702 | 721706 | 722308 | 201 | 103.6 | + | 744781323 | lipoprotein | S. hyicus | 4E-115 |
| 703 | 722321 | 722545 | 75 | 28.8 | + | 744781322 | membrane protein | S. hyicus | 4E-36 |
| 704 | 722608 | 723438 | 277 | 137.8 | - | 744781321 | octanoyltransferase LipM | S. hyicus | 0E+00 |
| 705 | 723577 | 723963 | 129 | 65.2 | + | 744781320 | rhodanese family protein | S. hyicus | 8E-88 |
| 706 | 724096 | 725571 | 492 | 311.4 | - | 744781319 | glycine dehydrogenase subunit 2 | S. hyicus | 0E+00 |
| 707 | 725561 | 726910 | 450 | 260.8 | - | 744781318 | glycine dehydrogenase subunit 1 | S. hyicus | 0E+00 |
| 708 | 726922 | 728013 | 364 | 249.6 | - | 744781317 | glycine cleavage system aminomethyltransferase T | S. hyicus | 0E+00 |
| 709 | 728165 | 728689 | 175 | 63.5 | - | 744781316 | shikimate kinase | S. hyicus | 2E-87 |
| 710 | 728673 | 728840 | 56 | 0.6 | - | 746619818 | hypothetical protein | S. hyicus | 2E-22 |
| 711 | 728854 | 729336 | 161 | 21.2 | - | 744781314 | competence protein ComGF | S. hyicus | 3E-69 |
| 712 | 729528 | 729959 | 144 | 17.3 | - | 744781312 | competence protein ComGD | S. hyicus | 5E-68 |
| 713 | 729949 | 730269 | 107 | 56.7 | - | 744781311 | competence protein ComGC | S. hyicus | 3E-52 |
| 714 | 730281 | 731009 | 243 | 56 | - | 744781310 | competence protein ComGB | S. hyicus | 5E-130 |
| 715 | 730993 | 731322 | 110 | 30.2 | - | 744781310 | competence protein ComGB | S. hyicus | 6E-48 |
| 716 | 731291 | 732265 | 325 | 130.8 | - | 744781309 | competence protein ComGA | S. hyicus | 0E+00 |
| 717 | 732322 | 732945 | 208 | 109.9 | - | 744781308 | metallo-beta-lactamase superfamily protein | S. hyicus | 4E-130 |
| 718 | 732960 | 733271 | 104 | 72.1 | - | 744781307 | hypothetical protein SHYC_06850 | S. hyicus | 6E-63 |
| 719 | 733272 | 734258 | 329 | 172.2 | - | 744781306 | glucokinase | S. hyicus | 0E+00 |
| 720 | 734255 | 734458 | 68 | 18.9 | - | 744781305 | hypothetical protein SHYC_06840 | S. hyicus | 5E-40 |
| 721 | 734460 | 735899 | 480 | 192.1 | - | 744781304 | rhomboid family protein | S. hyicus | 0E+00 |
| 722 | 735917 | 736456 | 180 | 88.4 | - | 744781303 | 5-formyltetrahydrofolate cyclo-ligase | S. hyicus | 1E-98 |
| 723 | 736569 | 736718 | 50 | 21.4 | - | 744781302 | 50S ribosomal protein L33 | S. hyicus | 1E-28 |
| 724 | 736789 | 737448 | 220 | 94 | - | 744781301 | phosphate transport system regulatory protein PhoU | S. hyicus | 2E-143 |
| 725 | 737441 | 738307 | 289 | 146.1 | - | 744781300 | phosphate ABC transporter ATP-binding protein | S. hyicus | 0E+00 |
| 726 | 738340 | 739218 | 293 | 140.2 | - | 744781299 | phosphate ABC transporter permease | S. hyicus | 1E-168 |
| 727 | 739220 | 740146 | 309 | 142.1 | - | 744781298 | phosphate ABC transporter permease | S. hyicus | 0E+00 |
| 728 | 740228 | 741193 | 322 | 208.8 | - | 744781297 | phosphate ABC transporter substrate-binding protein | S. hyicus | 0E+00 |
| 729 | 741388 | 743427 | 680 | 354.9 | - | 744781296 | penicillin-binding protein 3 | S. hyicus | 0E+00 |
| 730 | 743700 | 744299 | 200 | 136.5 | - | 744781295 | superoxide dismutase | S. hyicus | 7E-144 |
| 731 | 744696 | 745778 | 361 | 239.2 | + | 744781294 | 4-hydroxy-3-methylbut-2-en-1-yl diphosphate synthase | S. hyicus | 0E+00 |
| 732 | 745845 | 746255 | 137 | 44.8 | - | 744781293 | zinc-specific metallo-regulatory protein | S. hyicus | 4E-93 |
| 733 | 746218 | 747105 | 296 | 117.6 | - | 744781292 | zinc ABC transporter permease | S. hyicus | 0E+00 |
| 734 | 747102 | 747872 | 257 | 106.3 | - | 744781291 | zinc ABC transporter ATP-binding protein | S. hyicus | 7E-167 |
| 735 | 748127 | 749020 | 298 | 173.2 | - | 744781290 | endonuclease IV | S. hyicus | 0E+00 |
| 736 | 749034 | 750374 | 447 | 261.4 | - | 744781289 | DEAD/DEAH box RNA helicase | S. hyicus | 0E+00 |
| 737 | 750551 | 751528 | 326 | 172.1 | + | 744781288 | 4-hydroxy-3-methylbut-2-enyl diphosphate reductase | S. hyicus | 0E+00 |
| 738 | 751562 | 752665 | 368 | 189.2 | - | 744781287 | putative GTP cyclohydrolase 1 | S. hyicus | 0E+00 |
| 739 | 752652 | 753335 | 228 | 121.9 | - | 744781286 | tRNA (adenine(22)-N(1))-methyltransferase | S. hyicus | 4E-139 |
| 740 | 753462 | 754571 | 370 | 250.5 | - | 744781285 | RNA polymerase sigma factor SigA | S. hyicus | 0E+00 |
| 741 | 754589 | 756373 | 595 | 237.8 | - | 744781284 | DNA primase | S. hyicus | 0E+00 |
| 742 | 756440 | 757264 | 275 | 146.7 | - | 744781283 | phosphotransferase | S. hyicus | 0E+00 |
| 743 | 757280 | 757771 | 164 | 47.3 | - | 744781282 | transcriptional repressor CcpN | S. hyicus | 4E-105 |
| 744 | 758325 | 759716 | 464 | 272.1 | + | 744781281 | glycyl-tRNA synthetase | S. hyicus | 0E+00 |
| 745 | 759916 | 760698 | 261 | 100.5 | - | 744781280 | DNA repair protein RecO | S. hyicus | 4E-152 |
| 746 | 760722 | 761618 | 299 | 171.8 | - | 744781279 | GTPase Era | S. hyicus | 0E+00 |
| 747 | 761620 | 762024 | 135 | 44.7 | - | 744781278 | cytidine deaminase | S. hyicus | 5E-95 |
| 748 | 762037 | 762375 | 113 | 46.1 | - | 744781277 | diacylglycerol kinase | S. hyicus | 3E-56 |
| 749 | 762372 | 762836 | 155 | 102.1 | - | 744781276 | rRNA maturation factor YbeY | S. hyicus | 2E-98 |
| 750 | 762836 | 763783 | 316 | 196.5 | - | 744781275 | phosphate starvation protein PhoH | S. hyicus | 0E+00 |
| 751 | 764027 | 764638 | 204 | 103.4 | - | 744781274 | hypothetical protein SHYC_06685 | S. hyicus | 2E-82 |
| 752 | 764651 | 765649 | 333 | 237.8 | - | 744781273 | hypothetical protein SHYC_06680 | S. hyicus | 0E+00 |
| 753 | 765664 | 766371 | 236 | 94.9 | - | 744781272 | serine protease | S. hyicus | 3E-136 |
| 754 | 766556 | 766732 | 59 | 33.2 | - | 744781271 | 30S ribosomal protein S21 | S. hyicus | 1E-31 |
| 755 | 766863 | 768209 | 449 | 234.9 | - | 744781270 | threonylcarbamoyladenosine tRNA methylthiotransferase MtaB | S. hyicus | 0E+00 |
| 756 | 768228 | 768977 | 250 | 97.5 | - | 744781269 | ribosomal RNA small subunit methyltransferase E | S. hyicus | 4E-154 |
| 757 | 768983 | 769912 | 310 | 151.8 | - | 744781268 | 50S ribosomal protein L11 methyltransferase | S. hyicus | 0E+00 |
| 758 | 769916 | 771046 | 377 | 211.4 | - | 744781267 | molecular chaperone DnaJ | S. hyicus | 0E+00 |
| 759 | 771164 | 772993 | 610 | 462.6 | - | 744781266 | molecular chaperone DnaK | S. hyicus | 0E+00 |
| 760 | 773058 | 773687 | 210 | 147 | - | 744781265 | heat shock protein GrpE | S. hyicus | 3E-144 |
| 761 | 773724 | 774707 | 328 | 128.9 | - | 744781264 | heat-inducible transcription repressor HrcA | S. hyicus | 0E+00 |
| 762 | 774808 | 775929 | 374 | 163.8 | - | 744781263 | coproporphyrinogen III oxidase | S. hyicus | 0E+00 |
| 763 | 775995 | 777818 | 608 | 340.6 | - | 744781262 | translation elongation factor LepA | S. hyicus | 0E+00 |
| 764 | 778059 | 778307 | 83 | 48.8 | + | 744781261 | 30S ribosomal protein S20 | S. hyicus | 6E-50 |
| 765 | 778470 | 779447 | 326 | 169.6 | - | 744781260 | DNA polymerase III subunit delta | S. hyicus | 0E+00 |
| 766 | 779526 | 781757 | 744 | 180 | - | 744781259 | ComEC family protein | S. hyicus | 0E+00 |
| 767 | 781758 | 782225 | 156 | 61.7 | - | 744781258 | deoxycytidylate deaminase | S. hyicus | 3E-109 |
| 768 | 782327 | 782968 | 214 | 78.9 | - | 744781257 | ComEA family DNA binding protein | S. hyicus | 9E-84 |
| 769 | 783018 | 783740 | 241 | 77 | - | 744781256 | methyltransferase | S. hyicus | 5E-136 |
| 770 | 783740 | 784096 | 119 | 68.8 | - | 744781255 | ribosomal silencing factor RsfS | S. hyicus | 2E-74 |
| 771 | 784108 | 784680 | 191 | 102.1 | - | 744781254 | NAD metabolism-related HD hydrolase | S. hyicus | 8E-130 |
| 772 | 784673 | 785239 | 189 | 67.7 | - | 744781253 | nicotinate-nucleotide adenylyltransferase | S. hyicus | 4E-111 |
| 773 | 785242 | 785535 | 98 | 64.5 | - | 744781252 | YhbY family RNA-binding protein | S. hyicus | 8E-58 |
| 774 | 785545 | 786351 | 269 | 142.3 | - | 744781251 | shikimate 5-dehydrogenase | S. hyicus | 1E-164 |
| 775 | 786363 | 787463 | 367 | 181 | - | 744781250 | ribosome biogenesis GTPase YqeH | S. hyicus | 0E+00 |
| 776 | 787467 | 787991 | 175 | 92.7 | - | 744781249 | HAD superfamily hydrolase | S. hyicus | 1E-117 |
| 777 | 788059 | 788751 | 231 | 127.4 | - | 744781248 | 5'-methylthioadenosine/S-adenosylhomocysteine nucleosidase | S. hyicus | 6E-138 |
| 778 | 788928 | 790166 | 413 | 224.6 | - | 744781247 | transporter MntH | S. hyicus | 0E+00 |
| 779 | 790170 | 790931 | 254 | 149 | - | 744781246 | LamB/YcsF family protein | S. hyicus | 4E-157 |
| 780 | 790924 | 792288 | 455 | 258.7 | - | 744781245 | acetyl-CoA carboxylase biotin carboxylase subunit | S. hyicus | 0E+00 |
| 781 | 792301 | 792744 | 148 | 96.1 | - | 744781244 | acetyl-CoA carboxylase biotin carboxyl carrier protein | S. hyicus | 2E-69 |
| 782 | 792735 | 793760 | 342 | 136.6 | - | 744781243 | allophanate hydrolase subunit 2 | S. hyicus | 0E+00 |
| 783 | 793750 | 794481 | 244 | 123.6 | - | 744781242 | allophanate hydrolase subunit 1 | S. hyicus | 2E-154 |
| 784 | 794845 | 795321 | 159 | 97.4 | - | 744781241 | transcription elongation factor GreA | S. hyicus | 4E-109 |
| 785 | 795349 | 795975 | 209 | 113.5 | - | 744781240 | uridine kinase | S. hyicus | 7E-151 |
| 786 | 795980 | 797245 | 422 | 220.9 | - | 744781239 | peptidase U32 family protein | S. hyicus | 0E+00 |
| 787 | 797281 | 798210 | 310 | 177.2 | - | 744781238 | peptidase U32 family protein | S. hyicus | 0E+00 |
| 788 | 798203 | 798847 | 215 | 83.4 | - | 744781237 | O-methyltransferase | S. hyicus | 4E-137 |
| 789 | 799178 | 799498 | 107 | 95.2 | - | 744781236 | hypothetical protein SHYC_06495 | S. hyicus | 7E-66 |
| 790 | 799513 | 799941 | 143 | 74.6 | - | 744781235 | putative Holliday junction resolvase | S. hyicus | 8E-94 |
| 791 | 799934 | 800206 | 91 | 54.9 | - | 744781234 | hypothetical protein SHYC_06485 | S. hyicus | 4E-61 |
| 792 | 800270 | 802900 | 877 | 535.1 | - | 744781233 | alanyl-tRNA synthetase | S. hyicus | 0E+00 |
| 793 | 803133 | 805505 | 791 | 405 | - | 744781232 | RecD/TraA family helicase | S. hyicus | 0E+00 |
| 794 | 805509 | 806183 | 225 | 135.1 | - | 744781231 | TPR domain-containing protein | S. hyicus | 6E-134 |
| 795 | 806222 | 807331 | 370 | 204.4 | - | 744781230 | thiouridylase | S. hyicus | 0E+00 |
| 796 | 807334 | 808473 | 380 | 191.9 | - | 744781229 | cysteine desulfurase | S. hyicus | 0E+00 |
| 797 | 808694 | 809704 | 337 | 161 | + | 744781228 | luciferase family oxidoreductase | S. hyicus | 0E+00 |
| 798 | 809756 | 809920 | 55 | 41.6 | - | 744781227 | hypothetical protein SHYC_06450 | S. hyicus | 2E-17 |
| 799 | 810006 | 810191 | 62 | 43.7 | - | 744781226 | CsbD-like protein | S. hyicus | 2E-29 |
| 800 | 810258 | 811088 | 277 | 137.4 | - | 744781225 | 2,5-diketo-D-gluconic acid reductase | S. hyicus | 5E-174 |
| 801 | 811163 | 811582 | 140 | 90.7 | - | 744781224 | HTH-type transcriptional regulator CymR | S. hyicus | 2E-93 |
| 802 | 811725 | 812981 | 419 | 227.5 | + | 744781223 | recombinase RarA | S. hyicus | 0E+00 |
| 803 | 813090 | 813857 | 256 | 123.5 | - | 744781222 | tRNA threonylcarbamoyladenosine dehydratase | S. hyicus | 8E-172 |
| 804 | 814164 | 815930 | 589 | 384.6 | - | 744781221 | aspartyl-tRNA synthase | S. hyicus | 0E+00 |
| 805 | 815930 | 817201 | 424 | 238.8 | - | 744781220 | histidyl-tRNA synthetase | S. hyicus | 0E+00 |
| 806 | 817526 | 818401 | 292 | 150 | - | 744781219 | N-acetylmuramoyl-L-alanine amidase | S. hyicus | 0E+00 |
| 807 | 818398 | 818850 | 151 | 74.2 | - | 744781218 | D-tyrosyl-tRNA(Tyr) deacylase | S. hyicus | 2E-94 |
| 808 | 818862 | 821051 | 730 | 377.6 | - | 744781217 | GTP pyrophosphokinase | S. hyicus | 0E+00 |
| 809 | 821463 | 821981 | 173 | 122.5 | - | 744781216 | adenine phosphoribosyltransferase | S. hyicus | 9E-121 |
| 810 | 821993 | 824275 | 761 | 390.2 | - | 744781215 | single-stranded-DNA exonuclease RecJ | S. hyicus | 0E+00 |
| 811 | 824368 | 826647 | 760 | 383.1 | - | 744781214 | preprotein translocase subunit SecD/SecF | S. hyicus | 0E+00 |
| 812 | 826956 | 827219 | 88 | 65.2 | - | 744781213 | preprotein translocase subunit YajC | S. hyicus | 9E-47 |
| 813 | 827236 | 828375 | 380 | 178.5 | - | 744781212 | queuine tRNA-ribosyltransferase | S. hyicus | 0E+00 |
| 814 | 828389 | 829414 | 342 | 155 | - | 744781211 | S-adenosylmethionine tRNA ribosyltransferase-isomerase | S. hyicus | 0E+00 |
| 815 | 829428 | 830423 | 332 | 177.6 | - | 744781210 | Holliday junction ATP-dependent DNA helicase RuvB | S. hyicus | 0E+00 |
| 816 | 830439 | 831038 | 200 | 120.4 | - | 744781209 | Holliday junction ATP-dependent DNA helicase RuvA | S. hyicus | 3E-122 |
| 817 | 831051 | 831494 | 148 | 56.5 | - | 744781208 | ACT domain-containing protein | S. hyicus | 8E-97 |
| 818 | 831510 | 832799 | 430 | 253.8 | - | 744781207 | Obg family GTPase CgtA | S. hyicus | 0E+00 |
| 819 | 832968 | 833252 | 95 | 72.2 | - | 744781206 | 50S ribosomal protein L27 | S. hyicus | 1E-60 |
| 820 | 833263 | 833583 | 107 | 37.5 | - | 744781205 | phage-related ribosomal protease Prp | S. hyicus | 1E-71 |
| 821 | 833586 | 833894 | 103 | 66.3 | - | 744781204 | 50S ribosomal protein L21 | S. hyicus | 1E-67 |
| 822 | 834008 | 834532 | 175 | 37.4 | - | 744781203 | rod shape-determining protein MreD | S. hyicus | 6E-84 |
| 823 | 834529 | 835365 | 279 | 131.8 | - | 744781202 | cell shape-determining protein MreC | S. hyicus | 0E+00 |
| 824 | 835639 | 835782 | 48 | 15.4 | - | 744781201 | hypothetical protein SHYC_06320 | S. hyicus | 1E-19 |
| 825 | 835853 | 836311 | 153 | 79.8 | - | 744781200 | hypothetical protein SHYC_06315 | S. hyicus | 6E-90 |
| 826 | 836648 | 837331 | 228 | 103.2 | - | 744781198 | DNA repair protein RadC | S. hyicus | 1E-145 |
| 827 | 837328 | 838041 | 238 | 29.7 | - | 744781197 | peptidase | S. hyicus | 9E-101 |
| 828 | 838212 | 839474 | 421 | 237.1 | - | 744781196 | folylpolyglutamate synthase | S. hyicus | 0E+00 |
| 829 | 839486 | 842116 | 877 | 526.6 | - | 744781195 | valyl-tRNA synthetase | S. hyicus | 0E+00 |
| 830 | 842424 | 842975 | 184 | 80.8 | + | 744781194 | DNA-3-methyladenine glycosylase | S. hyicus | 1E-111 |
| 831 | 843031 | 844101 | 357 | 121.5 | - | 744781193 | putative ammonia monooxygenase | S. hyicus | 0E+00 |
| 832 | 844317 | 845603 | 429 | 252.9 | - | 744781192 | glutamate-1-semialdehyde aminotransferase | S. hyicus | 0E+00 |
| 833 | 845619 | 846596 | 326 | 188.5 | - | 744781191 | delta-aminolevulinic acid dehydratase | S. hyicus | 0E+00 |
| 834 | 846593 | 847273 | 227 | 96.9 | - | 744781190 | uroporphyrinogen III synthase | S. hyicus | 9E-109 |
| 835 | 847284 | 848210 | 309 | 172.3 | - | 744781189 | porphobilinogen deaminase | S. hyicus | 0E+00 |
| 836 | 848231 | 849067 | 279 | 35.3 | - | 744781188 | cytochrome C assembly protein | S. hyicus | 2E-176 |
| 837 | 849082 | 850422 | 447 | 241.6 | - | 744781187 | glutamyl-tRNA reductase | S. hyicus | 0E+00 |
| 838 | 850545 | 851132 | 196 | 98.1 | - | 744781186 | GTP-binding protein YsxC (EngB) | S. hyicus | 3E-125 |
| 839 | 851202 | 852464 | 421 | 276.9 | - | 744781185 | ATP-dependent Clp protease ATP-binding subunit ClpX | S. hyicus | 0E+00 |
| 840 | 852677 | 853990 | 438 | 380.6 | - | 744781184 | trigger factor | S. hyicus | 0E+00 |
| 841 | 854075 | 854998 | 308 | 134 | - | 744781183 | hypothetical protein SHYC_06235 | S. hyicus | 0E+00 |
| 842 | 855008 | 855619 | 204 | 110.5 | - | 744781182 | MutT/nudix family protein | S. hyicus | 5E-144 |
| 843 | 855839 | 856195 | 119 | 76.9 | - | 744781181 | 50S ribosomal protein L20 | S. hyicus | 6E-78 |
| 844 | 856255 | 856455 | 67 | 31.1 | - | 744781180 | 50S ribosomal protein L35 | S. hyicus | 4E-39 |
| 845 | 856483 | 857058 | 192 | 102.7 | - | 744781179 | translation initiation factor IF-3 | S. hyicus | 2E-136 |
| 846 | 857212 | 858720 | 503 | 231.7 | - | 744781178 | lysine permease | S. hyicus | 0E+00 |
| 847 | 859070 | 861007 | 646 | 401.1 | - | 744781177 | threonyl-tRNA synthetase | S. hyicus | 0E+00 |
| 848 | 861300 | 862223 | 308 | 137.8 | - | 744781176 | primosomal protein DnaI | S. hyicus | 0E+00 |
| 849 | 862220 | 863587 | 456 | 212.1 | - | 744781175 | chromosome replication protein DnaB | S. hyicus | 0E+00 |
| 850 | 863589 | 864056 | 156 | 45.8 | - | 744781174 | transcriptional regulator NrdR | S. hyicus | 3E-111 |
| 851 | 864223 | 865233 | 337 | 216.7 | - | 744781173 | glyceraldehyde-3-phosphate dehydrogenase | S. hyicus | 0E+00 |
| 852 | 865551 | 866159 | 203 | 133.5 | - | 744781172 | dephospho-CoA kinase | S. hyicus | 5E-137 |
| 853 | 866176 | 867048 | 291 | 122.2 | - | 744781171 | formamidopyrimidine-DNA glycosylase | S. hyicus | 0E+00 |
| 854 | 867064 | 869691 | 876 | 499 | - | 744781170 | DNA polymerase I | S. hyicus | 0E+00 |
| 855 | 869812 | 871512 | 567 | 267.8 | - | 744781169 | alkaline phosphatase synthesis sensor protein PhoR | S. hyicus | 0E+00 |
| 856 | 871513 | 872217 | 235 | 156.5 | - | 744781168 | PhoP family transcriptional regulator | S. hyicus | 5E-166 |
| 857 | 872520 | 873782 | 421 | 285 | - | 744781167 | isocitrate dehydrogenase | S. hyicus | 0E+00 |
| 858 | 873832 | 874950 | 373 | 201.7 | - | 744781166 | citrate synthase | S. hyicus | 0E+00 |
| 859 | 875348 | 877108 | 587 | 388 | - | 744781165 | pyruvate kinase | S. hyicus | 0E+00 |
| 860 | 877125 | 878096 | 324 | 198.2 | - | 744781164 | 6-phosphofructokinase | S. hyicus | 0E+00 |
| 861 | 878435 | 879382 | 316 | 207.9 | - | 744781163 | acetyl-CoA carboxylase subunit alpha | S. hyicus | 0E+00 |
| 862 | 879382 | 880242 | 287 | 150.7 | - | 744781162 | acetyl-CoA carboxyl transferase subunit beta | S. hyicus | 0E+00 |
| 863 | 880275 | 881504 | 410 | 258.8 | - | 744781161 | NAD-dependent malic enzyme | S. hyicus | 0E+00 |
| 864 | 881622 | 884813 | 1064 | 490.5 | - | 744781160 | DNA polymerase III subunit alpha | S. hyicus | 0E+00 |
| 865 | 884828 | 885763 | 312 | 148.2 | - | 744781159 | bifunctional oligoribonuclease and PAP phosphatase NrnA | S. hyicus | 0E+00 |
| 866 | 885776 | 887071 | 432 | 227.3 | - | 744781158 | CBS domain protein | S. hyicus | 0E+00 |
| 867 | 887258 | 887671 | 138 | 86 | + | 744781157 | universal stress protein family protein | S. hyicus | 6E-88 |
| 868 | 887796 | 888488 | 231 | 126.1 | - | 744781156 | metal-dependent hydrolase | S. hyicus | 6E-153 |
| 869 | 888608 | 889663 | 352 | 190.3 | + | 744781155 | metallopeptidase | S. hyicus | 0E+00 |
| 870 | 889989 | 891104 | 372 | 254.9 | - | 744781154 | alanine dehydrogenase 2 | S. hyicus | 0E+00 |
| 871 | 891252 | 891752 | 167 | 109 | + | 744781153 | universal stress protein UspA | S. hyicus | 6E-119 |
| 872 | 891843 | 893039 | 399 | 265.1 | - | 744781152 | acetate kinase | S. hyicus | 0E+00 |
| 873 | 893123 | 894070 | 316 | 165.3 | - | 744781151 | adenine DNA methyltransferase | S. hyicus | 0E+00 |
| 874 | 894205 | 894702 | 166 | 104.9 | - | 744781150 | thiol peroxidase | S. hyicus | 4E-112 |
| 875 | 894771 | 895325 | 185 | 70 | - | 746621850 | RDD family protein | S. hyicus | 1E-92 |
| 876 | 895335 | 896324 | 330 | 210.7 | - | 744781148 | protease | S. hyicus | 0E+00 |
| 877 | 896501 | 897274 | 258 | 96.7 | - | 744781147 | membrane protein | S. hyicus | 6E-139 |
| 878 | 897296 | 898516 | 407 | 255.9 | - | 744781146 | putative tRNA sulfurtransferase | S. hyicus | 0E+00 |
| 879 | 898513 | 899652 | 380 | 190 | - | 744781145 | aminotransferase | S. hyicus | 0E+00 |
| 880 | 899820 | 901523 | 568 | 353 | - | 744781144 | septation ring formation regulator EzrA | S. hyicus | 0E+00 |
| 881 | 901703 | 902158 | 152 | 80.6 | + | 744781143 | free methionine-R-sulfoxide reductase | S. hyicus | 1E-90 |
| 882 | 902386 | 902988 | 201 | 140.7 | + | 744781142 | 30S ribosomal protein S4 | S. hyicus | 1E-144 |
| 883 | 903110 | 904066 | 319 | 158.7 | + | 744781141 | ornithine cyclodeaminase | S. hyicus | 0E+00 |
| 884 | 904196 | 904939 | 248 | 134.7 | - | 744781140 | glycerophosphodiester phosphodiesterase | S. hyicus | 9E-167 |
| 885 | 905016 | 905462 | 149 | 57.6 | + | 744781139 | peroxiredoxin | S. hyicus | 7E-71 |
| 886 | 905552 | 906712 | 387 | 183.8 | + | 744781138 | aminotransferase class V | S. hyicus | 0E+00 |
| 887 | 906702 | 908294 | 531 | 304.2 | + | 744781137 | D-3-phosphoglycerate dehydrogenase | S. hyicus | 0E+00 |
| 888 | 908384 | 909517 | 378 | 208.5 | - | 744781136 | HAD family hydrolase | S. hyicus | 0E+00 |
| 889 | 909638 | 910255 | 206 | 109.2 | - | 744781135 | acyl-phosphate glycerol 3-phosphate acyltransferase | S. hyicus | 7E-135 |
| 890 | 910411 | 911637 | 409 | 225.6 | + | 744781134 | serine protease | S. hyicus | 0E+00 |
| 891 | 911684 | 912946 | 421 | 266.1 | - | 744781133 | tyrosyl-tRNA synthetase | S. hyicus | 0E+00 |
| 892 | 913291 | 914172 | 294 | 131.7 | + | 744781132 | transglycosylase | S. hyicus | 0E+00 |
| 893 | 914325 | 915992 | 556 | 386.9 | - | 744781131 | formate--tetrahydrofolate ligase | S. hyicus | 0E+00 |
| 894 | 916209 | 917918 | 570 | 310.7 | - | 744781130 | acetyl-CoA synthetase | S. hyicus | 0E+00 |
| 895 | 918073 | 918705 | 211 | 105.7 | + | 744781129 | acetyltransferase AcuA | S. hyicus | 1E-148 |
| 896 | 918720 | 919877 | 386 | 183.5 | + | 744781128 | acetoin utilization protein AcuC | S. hyicus | 0E+00 |
| 897 | 919973 | 920965 | 331 | 189.7 | - | 744781127 | catabolite control protein A | S. hyicus | 0E+00 |
| 898 | 921163 | 922254 | 364 | 197.5 | - | 744781126 | 3-deoxy-7-phosphoheptulonate synthase/chorismate mutase | S. hyicus | 0E+00 |
| 899 | 922520 | 923515 | 332 | 223 | - | 744781125 | hypothetical protein SHYC_05945 | S. hyicus | 2E-132 |
| 900 | 923608 | 924213 | 202 | 114 | - | 744781124 | hypothetical protein SHYC_05940 | S. hyicus | 2E-119 |
| 901 | 924290 | 925603 | 438 | 246.5 | - | 744781123 | UDP-N-acetylmuramate--alanine ligase | S. hyicus | 0E+00 |
| 902 | 925626 | 928841 | 1072 | 562 | - | 744781122 | FtsK/SpoIIIE family protein | S. hyicus | 0E+00 |
| 903 | 928856 | 929455 | 200 | 121.2 | - | 744781121 | tRNA-binding protein | S. hyicus | 1E-124 |
| 904 | 929472 | 930332 | 287 | 154.2 | - | 744781120 | hypothetical protein SHYC_05920 | S. hyicus | 0E+00 |
| 905 | 930425 | 930733 | 103 | 57 | - | 744781119 | thioredoxin-like protein | S. hyicus | 8E-63 |
| 906 | 930748 | 931818 | 357 | 207.1 | - | 744781118 | peptidase M42-family protein | S. hyicus | 0E+00 |
| 907 | 931912 | 932238 | 109 | 49.6 | + | 744781117 | hypothetical protein SHYC_05905 | S. hyicus | 1E-66 |
| 908 | 932306 | 933133 | 276 | 130.2 | - | 744781116 | lactonase YtnP family protein | S. hyicus | 2E-176 |
| 909 | 933233 | 933898 | 222 | 104.2 | - | 744781115 | tRNA (guanine-N7)-methyltransferase | S. hyicus | 4E-148 |
| 910 | 933913 | 934704 | 264 | 111.7 | - | 744781114 | phosphotransferase family protein | S. hyicus | 0E+00 |
| 911 | 934933 | 935781 | 283 | 159.7 | - | 744781113 | D-alanine aminotransferase | S. hyicus | 0E+00 |
| 912 | 935783 | 937192 | 470 | 282.6 | - | 744781112 | dipeptidase PepV | S. hyicus | 0E+00 |
| 913 | 937377 | 937796 | 140 | 91.3 | - | 744781111 | hypothetical protein SHYC_05875 | S. hyicus | 2E-87 |
| 914 | 937868 | 938575 | 236 | 109.8 | - | 744781110 | ribosomal small subunit pseudouridine synthase A | S. hyicus | 5E-123 |
| 915 | 938575 | 940215 | 547 | 214.3 | - | 744781109 | O-antigen, teichoic acid lipoteichoic acids export-related membrane protein | S. hyicus | 0E+00 |
| 916 | 940332 | 941594 | 421 | 197.9 | + | 744781108 | NAD(FAD)-utilizing dehydrogenase | S. hyicus | 0E+00 |
| 917 | 941637 | 941948 | 104 | 57.6 | - | 744781107 | putative sulfurtransferase | S. hyicus | 6E-64 |
| 918 | 941971 | 944382 | 804 | 461.1 | - | 744781106 | leucyl-tRNA synthetase | S. hyicus | 0E+00 |
| 919 | 944743 | 945924 | 394 | 167.4 | - | 744781105 | MFS transporter | S. hyicus | 0E+00 |
| 920 | 946036 | 946995 | 320 | 131.4 | + | 744781104 | radical SAM protein | S. hyicus | 0E+00 |
| 921 | 946992 | 947555 | 188 | 65.4 | + | 744781103 | rRNA methyltransferase | S. hyicus | 4E-107 |
| 922 | 947653 | 948063 | 137 | 61 | - | 744781102 | HTH-type transcriptional regulator Rot | S. hyicus | 8E-86 |
| 923 | 948206 | 949159 | 318 | 222.4 | - | 744781101 | lactate dehydrogenase | S. hyicus | 0E+00 |
| 924 | 949288 | 950109 | 274 | 126.8 | - | 744781100 | lysophospholipase | S. hyicus | 0E+00 |
| 925 | 950245 | 950577 | 111 | 43.9 | - | 744781099 | hypothetical protein SHYC_05810 | S. hyicus | 9E-62 |
| 926 | 950781 | 950894 | 38 | 0.8 | + |  | No Significant Match |  |  |
| 927 | 951112 | 952152 | 347 | 173.1 | + | 744781098 | proline dehydrogenase | S. hyicus | 0E+00 |
| 928 | 952250 | 952708 | 153 | 108.6 | - | 744781097 | 6,7-dimethyl-8-ribityllumazine synthase | S. hyicus | 2E-101 |
| 929 | 952720 | 953901 | 394 | 229.8 | - | 744781096 | 3,4-dihydroxy-2-butanone 4- phosphate synthase/GTP cyclohydrolase II | S. hyicus | 0E+00 |
| 930 | 953915 | 954544 | 210 | 102.9 | - | 744781095 | riboflavin synthase subunit alpha | S. hyicus | 2E-135 |
| 931 | 954549 | 955595 | 349 | 129.9 | - | 744781094 | diaminohydroxyphosphoribosylaminopyrimidine deaminase | S. hyicus | 0E+00 |
| 932 | 955981 | 957705 | 575 | 261.9 | - | 744781093 | pyridine nucleotide-disulfide oxidoreductase | S. hyicus | 0E+00 |
| 933 | 958012 | 958878 | 289 | 151.7 | - | 744781092 | glucosaminidase | S. hyicus | 0E+00 |
| 934 | 959042 | 959266 | 75 | 39.6 | - | 744781091 | hypothetical protein SHYC_05765 | S. hyicus | 2E-43 |
| 935 | 959467 | 959928 | 154 | 21.9 | + | 744781090 | RNA polymerase sigma factor SigS | S. hyicus | 5E-64 |
| 936 | 959944 | 960378 | 145 | 21.1 | + | 744781089 | hypothetical protein SHYC_05755 | S. hyicus | 5E-67 |
| 937 | 960361 | 960810 | 150 | 55.4 | - | 744781088 | membrane protein | S. hyicus | 2E-65 |
| 938 | 960904 | 961614 | 237 | 167 | - | 744781087 | transaldolase | S. hyicus | 3E-162 |
| 939 | 961768 | 962070 | 101 | 33 | + | 744781086 | membrane protein | S. hyicus | 1E-44 |
| 940 | 962091 | 962456 | 122 | 33.8 | + | 744781085 | CrcB-like protein | S. hyicus | 1E-48 |
| 941 | 962453 | 962818 | 122 | 22.1 | + | 744781084 | CrcB-like protein | S. hyicus | 3E-39 |
| 942 | 963003 | 963839 | 279 | 135.6 | - | 744781083 | oxidoreductase, aldo/keto reductase family | S. hyicus | 5E-154 |
| 943 | 964053 | 964961 | 303 | 145.8 | - | 744781082 | hypothetical protein SHYC_05720 | S. hyicus | 0E+00 |
| 944 | 965050 | 966249 | 400 | 265.7 | - | 744781081 | S-adenosylmethionine synthetase | S. hyicus | 0E+00 |
| 945 | 966681 | 968261 | 527 | 295.7 | + | 744781080 | phosphoenolpyruvate carboxykinase | S. hyicus | 0E+00 |
| 946 | 968286 | 969071 | 262 | 116.2 | - | 744781079 | dipeptidyl aminopeptidase | S. hyicus | 3E-143 |
| 947 | 969052 | 969522 | 157 | 46.8 | - | 744781078 | nucleoside triphosphatase | S. hyicus | 5E-87 |
| 948 | 969585 | 969815 | 77 | 9.2 | + | 744781077 | putative membrane protein insertion efficiency factor | S. hyicus | 1E-46 |
| 949 | 969812 | 970801 | 330 | 156.1 | - | 744781076 | O-succinylbenzoic acid synthetase | S. hyicus | 1E-172 |
| 950 | 970798 | 972204 | 469 | 211.3 | - | 744781075 | 2-succinylbenzoate--CoA ligase | S. hyicus | 0E+00 |
| 951 | 972481 | 972774 | 98 | 64.7 | + | 744781074 | hypothetical protein SHYC_05680 | S. hyicus | 5E-56 |
| 952 | 973281 | 973772 | 164 | 122.5 | - | 744781073 | alkaline shock protein 23 | S. hyicus | 6E-114 |
| 953 | 973810 | 974052 | 81 | 23.9 | - | 744781072 | membrane protein | S. hyicus | 5E-48 |
| 954 | 974073 | 974618 | 182 | 71.2 | - | 744781071 | hypothetical protein SHYC_05665 | S. hyicus | 6E-111 |
| 955 | 975180 | 976034 | 285 | 105.7 | - | 744781070 | membrane protein | S. hyicus | 0E+00 |
| 956 | 976748 | 977593 | 282 | 94.8 | - | 744781069 | membrane protein | S. hyicus | 0E+00 |
| 957 | 978688 | 979131 | 148 | 69.1 | - |  | No Significant Match |  |  |
| 958 | 979121 | 979345 | 75 | 22 | - |  | No Significant Match |  |  |
| 959 | 979637 | 980374 | 246 | 65.2 | - | 746621664 | hypothetical protein, partial | S. hyicus | 1E-08 |
| 960 | 980956 | 982425 | 490 | 223.7 | - | P24556 | Autolysin | S. aureus | 2E-59 |
| 961 | 982427 | 982675 | 83 | 48.2 | - |  | No Significant Match |  |  |
| 962 | 982815 | 983159 | 115 | 36.8 | - |  | No Significant Match |  |  |
| 963 | 983208 | 983522 | 105 | 66.9 | - |  | No Significant Match |  |  |
| 964 | 983565 | 983714 | 50 | 31.5 | - |  | No Significant Match |  |  |
| 965 | 983724 | 987206 | 1161 | 557.2 | - |  | No Significant Match |  |  |
| 966 | 987208 | 988695 | 496 | 219.1 | - |  | No Significant Match |  |  |
| 967 | 988692 | 993503 | 1604 | 702.4 | - | E7DNB6 | Tape measure protein | Pneumococcus phage | 3E-81 |
| 968 | 993520 | 993663 | 48 | 1.5 | - |  | No Significant Match |  |  |
| 969 | 993723 | 994094 | 124 | 86.4 | - |  | No Significant Match |  |  |
| 970 | 994162 | 994341 | 60 | 31.4 | - |  | No Significant Match |  |  |
| 971 | 994359 | 994982 | 208 | 149.7 | - |  | No Significant Match |  |  |
| 972 | 994995 | 995390 | 132 | 47 | - |  | No Significant Match |  |  |
| 973 | 995411 | 995800 | 130 | 70.7 | - |  | No Significant Match |  |  |
| 974 | 995790 | 996158 | 123 | 37.8 | - |  | No Significant Match |  |  |
| 975 | 996142 | 996429 | 96 | 24.8 | - |  | No Significant Match |  |  |
| 976 | 996447 | 997646 | 400 | 246.7 | - |  | No Significant Match |  |  |
| 977 | 997658 | 998377 | 240 | 148.1 | - | Q6ME32 | ATP-dependent Clp protease proteolytic subunit 1 | Protochlamydia amoebophila | 5E-13 |
| 978 | 998334 | 999512 | 393 | 170 | - |  | No Significant Match |  |  |
| 979 | 999532 | 1001199 | 556 | 247.3 | - | P59217 | Putative terminase large subunit | Shigella phage | 2E-13 |
| 980 | 1001186 | 1001569 | 128 | 63.2 | - |  | No Significant Match |  |  |
| 981 | 1001706 | 1002434 | 243 | 62.5 | - |  | No Significant Match |  |  |
| 982 | 1002488 | 1002883 | 132 | 68.2 | - | Q7MZD8 | Antitoxin HicB 2 | Photorhabdus luminescens | 5E-05 |
| 983 | 1002918 | 1003103 | 62 | 25.6 | - |  | No Significant Match |  |  |
| 984 | 1003276 | 1003704 | 143 | 42.5 | - |  | No Significant Match |  |  |
| 985 | 1003722 | 1003832 | 37 | 10.1 | - |  | No Significant Match |  |  |
| 986 | 1004002 | 1004517 | 172 | 67.8 | - |  | No Significant Match |  |  |
| 987 | 1004696 | 1004791 | 32 | 4.7 | - |  | No Significant Match |  |  |
| 988 | 1004796 | 1004984 | 63 | 17.6 | - |  | No Significant Match |  |  |
| 989 | 1005035 | 1005337 | 101 | 50.4 | - | P51714 | Uncharacterized 18.2 kDa protein in rep-hol intergenic region | Haemophilus phage | 4E-10 |
| 990 | 1005338 | 1005697 | 120 | 63.1 | - | 746621824 | hypothetical protein, partial | S. hyicus | 1E-35 |
| 991 | 1005684 | 1006004 | 107 | 29.5 | - | 744780981 | hypothetical protein SHYC_05135 | S. hyicus | 7E-18 |
| 992 | 1006001 | 1006396 | 132 | 56.5 | - | 744780979 | hypothetical protein SHYC_05125 | S. hyicus | 1E-15 |
| 993 | 1006409 | 1006621 | 71 | 18.7 | - |  | No Significant Match |  |  |
| 994 | 1006618 | 1007109 | 164 | 80.4 | - | 744781020 | putative nucleoside 2-deoxyribosyltransferase | S. hyicus | 6E-24 |
| 995 | 1007106 | 1007336 | 77 | 35.7 | - |  | No Significant Match |  |  |
| 996 | 1007329 | 1007514 | 62 | 21.7 | - |  | No Significant Match |  |  |
| 997 | 1007511 | 1007933 | 141 | 49.5 | - |  | No Significant Match |  |  |
| 998 | 1007935 | 1008357 | 141 | 36.9 | - | P45911 | Uncharacterized protein YqaN | Bacillus subtilis | 4E-07 |
| 999 | 1008344 | 1008562 | 73 | 25 | - | 739689061 | hypothetical protein | S. pseudintermedius | 8E-32 |
| 1000 | 1008562 | 1009809 | 416 | 139.7 | - | P45256 | Replicative DNA helicase | Haemophilus influenzae | 4E-47 |
| 1001 | 1009796 | 1010146 | 117 | 41.4 | - |  | No Significant Match |  |  |
| 1002 | 1010146 | 1010871 | 242 | 108.4 | - |  | No Significant Match |  |  |
| 1003 | 1010858 | 1011541 | 228 | 40.5 | - |  | No Significant Match |  |  |
| 1004 | 1011556 | 1011954 | 133 | 74.8 | - | P59933 | Single-stranded DNA-binding protein | Wolinella succinogenes | 3E-19 |
| 1005 | 1011954 | 1012616 | 221 | 86.7 | - |  | No Significant Match |  |  |
| 1006 | 1012617 | 1013102 | 162 | 79.9 | - | 744781027 | hypothetical protein SHYC_05375 | S. hyicus | 4E-09 |
| 1007 | 1013099 | 1013365 | 89 | 51.9 | - |  | No Significant Match |  |  |
| 1008 | 1013446 | 1013619 | 58 | 8.4 | - |  | No Significant Match |  |  |
| 1009 | 1013616 | 1013942 | 109 | 35.5 | - |  | No Significant Match |  |  |
| 1010 | 1013955 | 1014236 | 94 | 45.8 | - | 505961606 | hypothetical protein | S. epidermidis | 2E-10 |
| 1011 | 1014277 | 1014519 | 81 | 37.7 | - |  | No Significant Match |  |  |
| 1012 | 1014568 | 1014903 | 112 | 52.9 | + |  | No Significant Match |  |  |
| 1013 | 1014889 | 1015122 | 78 | 27.8 | - |  | No Significant Match |  |  |
| 1014 | 1015164 | 1015478 | 105 | 46.6 | - |  | No Significant Match |  |  |
| 1015 | 1015489 | 1016076 | 196 | 105.5 | - |  | No Significant Match |  |  |
| 1016 | 1016079 | 1017002 | 308 | 128.9 | - |  | No Significant Match |  |  |
| 1017 | 1017018 | 1017287 | 90 | 38.2 | - | O28646 | Uncharacterized HTH-type transcriptional regulator AF_1627 | Archaeoglobus fulgidus | 2E-05 |
| 1018 | 1017422 | 1018126 | 235 | 111.9 | + | Q314F2 | LexA repressor | Desulfovibrio desulfuricans | 2E-07 |
| 1019 | 1018691 | 1018882 | 64 | 36.7 | + |  | No Significant Match |  |  |
| 1020 | 1018987 | 1019703 | 239 | 51.5 | + |  | No Significant Match |  |  |
| 1021 | 1019897 | 1020928 | 344 | 109.3 | + | P96629 | ICEBs1 integrase | Bacillus subtilis | 2E-10 |
| 1022 | 1022173 | 1022727 | 185 | 73.6 | - | 744781065 | putative hydrolase | S. hyicus | 1E-119 |
| 1023 | 1022810 | 1024213 | 468 | 213.9 | - | 744781064 | protoporphyrinogen oxidase | S. hyicus | 0E+00 |
| 1024 | 1024227 | 1025150 | 308 | 161.1 | - | 744781063 | ferrochelatase | S. hyicus | 0E+00 |
| 1025 | 1025173 | 1026216 | 348 | 175.7 | - | 744781062 | uroporphyrinogen decarboxylase | S. hyicus | 0E+00 |
| 1026 | 1026413 | 1026943 | 177 | 92.7 | + | 744781061 | signal transduction protein TRAP | S. hyicus | 9E-118 |
| 1027 | 1026991 | 1028205 | 405 | 127.1 | - | 744781060 | EcsB-related ABC transporter protein | S. hyicus | 0E+00 |
| 1028 | 1028202 | 1028939 | 246 | 119.3 | - | 744781059 | ABC transporter ATP-binding protein | S. hyicus | 3E-171 |
| 1029 | 1029071 | 1029499 | 143 | 78.9 | + | 744781058 | histidine triad (HIT) protein | S. hyicus | 3E-93 |
| 1030 | 1029579 | 1029935 | 119 | 68.1 | + | 744781057 | hypothetical protein SHYC_05535 | S. hyicus | 2E-74 |
| 1031 | 1030035 | 1030565 | 177 | 81 | + | 744781056 | HTH-type transcriptional regulator Hpr | S. hyicus | 1E-127 |
| 1032 | 1030618 | 1031157 | 180 | 38.6 | + | 744781055 | hypothetical protein SHYC_05525 | S. hyicus | 2E-106 |
| 1033 | 1031154 | 1031246 | 31 | 0.2 | + |  | No Significant Match |  |  |
| 1034 | 1031370 | 1032338 | 323 | 218.8 | + | 744781054 | foldase protein PrsA | S. hyicus | 0E+00 |
| 1035 | 1032499 | 1033440 | 314 | 167 | - | 744781053 | 3'-5' exonuclease YhaM | S. hyicus | 0E+00 |
| 1036 | 1033459 | 1036386 | 976 | 546.7 | - | 744781052 | hypothetical protein SHYC_05510 | S. hyicus | 0E+00 |
| 1037 | 1036390 | 1037577 | 396 | 221.6 | - | 744781051 | DNA repair exonuclease | S. hyicus | 0E+00 |
| 1038 | 1037811 | 1038155 | 115 | 84.3 | - | 744781050 | putative regulatory protein | S. hyicus | 5E-73 |
| 1039 | 1038241 | 1039371 | 377 | 190.6 | - | 744781049 | membrane protein | S. hyicus | 0E+00 |
| 1040 | 1039524 | 1039988 | 155 | 66.7 | - | 744781048 | transcriptional regulator | S. hyicus | 1E-111 |
| 1041 | 1040379 | 1040621 | 81 | 18.8 | + | 744781047 | hypothetical protein SHYC_05485 | S. hyicus | 3E-36 |
| 1042 | 1040761 | 1040979 | 73 | 11.2 | - | 744781605 | membrane protein | S. hyicus | 2E-13 |
| 1043 | 1041058 | 1041351 | 98 | 35.7 | - | 744781046 | hypothetical protein SHYC_05475 | S. hyicus | 1E-22 |
| 1044 | 1041617 | 1043842 | 742 | 382.7 | - | 744781045 | fibronectin-binding protein | S. hyicus | 0E+00 |
| 1045 | 1044230 | 1044808 | 193 | 104.6 | - | 744781044 | thioredoxin family protein | S. hyicus | 3E-115 |
| 1046 | 1045034 | 1046764 | 577 | 291.2 | - | 744781771 | fibronectin-binding protein | S. hyicus | 9E-95 |
| 1047 | 1046704 | 1047462 | 253 | 98.7 | - | 744781771 | fibronectin-binding protein | S. hyicus | 8E-37 |
| 1048 | 1047428 | 1047739 | 104 | 38.9 | - | 744781498 | fibronectin-binding protein | S. hyicus | 2E-13 |
| 1049 | 1048309 | 1049595 | 429 | 231.7 | - | 744780916 | sodium:dicarboxylate symporter family protein | S. hyicus | 0E+00 |
| 1050 | 1049772 | 1050329 | 186 | 63.3 | - | 744780911 | signal peptidase IB | S. hyicus | 2E-104 |
| 1051 | 1050365 | 1050580 | 72 | 15.9 | - |  | No Significant Match |  |  |
| 1052 | 1050767 | 1052758 | 664 | 369.4 | - | 744780910 | hypothetical protein SHYC_04765 | S. hyicus | 0E+00 |
| 1053 | 1053057 | 1053926 | 290 | 125.9 | + | 744780909 | cation efflux family protein | S. hyicus | 0E+00 |
| 1054 | 1054004 | 1057660 | 1219 | 655.4 | - | 746619106 | hypothetical protein | S. hyicus | 0E+00 |
| 1055 | 1058070 | 1058696 | 209 | 112.2 | - | 744780907 | response regulator protein | S. hyicus | 3E-137 |
| 1056 | 1058697 | 1059818 | 374 | 172.4 | - | 744780906 | signal transduction histidine kinase | S. hyicus | 0E+00 |
| 1057 | 1059998 | 1060828 | 277 | 163.1 | + | 744780905 | RluD subfamily ribosomal large subunit pseudouridine synthase | S. hyicus | 0E+00 |
| 1058 | 1060866 | 1063028 | 721 | 349.6 | - | 744780904 | polyphosphate kinase | S. hyicus | 0E+00 |
| 1059 | 1063123 | 1064649 | 509 | 268.3 | - | 744780903 | exopolyphosphatase | S. hyicus | 0E+00 |
| 1060 | 1064671 | 1066056 | 462 | 278.8 | - | 744780902 | fumarate hydratase | S. hyicus | 0E+00 |
| 1061 | 1066213 | 1066614 | 134 | 35.6 | - | 744780901 | membrane protein | S. hyicus | 2E-70 |
| 1062 | 1066773 | 1066928 | 52 | 6.4 | - | 744780900 | hypothetical protein SHYC_04715 | S. hyicus | 3E-23 |
| 1063 | 1066947 | 1067546 | 200 | 132.3 | - | 744780899 | glucosamine-6-phosphate isomerase | S. hyicus | 1E-134 |
| 1064 | 1067564 | 1068733 | 390 | 221.1 | - | 744780898 | N-acetylglucosamine-6-phosphate deacetylase | S. hyicus | 0E+00 |
| 1065 | 1068748 | 1069218 | 157 | 89.6 | - | 744780897 | tRNA (cytidine(34)-2'-O)-methyltransferase | S. hyicus | 6E-113 |
| 1066 | 1069221 | 1070348 | 376 | 187.2 | - | 744780896 | epoxyqueuosine reductase | S. hyicus | 0E+00 |
| 1067 | 1070521 | 1071243 | 241 | 130.6 | - | 744780895 | glutamine ABC transporter ATP-binding protein | S. hyicus | 1E-153 |
| 1068 | 1071236 | 1072693 | 486 | 268.3 | - | 744780894 | amino acid ABC transporter substrate-binding/permease protein | S. hyicus | 0E+00 |
| 1069 | 1072811 | 1073869 | 353 | 152.5 | - | 744780893 | PfoR family protein | S. hyicus | 0E+00 |
| 1070 | 1082112 | 1082573 | 154 | 64.8 | - | 744780892 | peroxide-responsive repressor PerR | S. hyicus | 9E-109 |
| 1071 | 1082658 | 1083608 | 317 | 165.6 | - | 744780891 | 2-hydroxyacid dehydrogenase | S. hyicus | 1E-175 |
| 1072 | 1083614 | 1084072 | 153 | 88.6 | - | 744780890 | putative peroxiredoxin | S. hyicus | 6E-97 |
| 1073 | 1084153 | 1085454 | 434 | 279.3 | + | 744780889 | glutamate-1-semialdehyde-2,1-aminomutase | S. hyicus | 0E+00 |
| 1074 | 1085789 | 1086886 | 366 | 140.3 | + | 744780888 | membrane protein | S. hyicus | 0E+00 |
| 1075 | 1086991 | 1088571 | 527 | 263.5 | - | 744780887 | glutamate synthase domain protein | S. hyicus | 0E+00 |
| 1076 | 1088732 | 1090468 | 579 | 292.3 | - | 744780886 | ABC transporter ATP-binding protein | S. hyicus | 0E+00 |
| 1077 | 1090583 | 1091137 | 185 | 43.1 | - | 744780885 | RNAse G and E associated protein | S. hyicus | 8E-133 |
| 1078 | 1091185 | 1092237 | 351 | 133 | - | 744780884 | adenine glycosylase | S. hyicus | 0E+00 |
| 1079 | 1092326 | 1093303 | 326 | 132.8 | + | 744780883 | membrane bound metal dependent hydrolase | S. hyicus | 0E+00 |
| 1080 | 1093704 | 1094474 | 257 | 59.2 | - | 744780882 | teichoic acid translocation permease | S. hyicus | 1E-149 |
| 1081 | 1094483 | 1096009 | 509 | 242.2 | - | 744780881 | teichoic acid ABC transporter ATP-binding protein | S. hyicus | 0E+00 |
| 1082 | 1096024 | 1096335 | 104 | 50.7 | - | 744780880 | hypothetical protein SHYC_04475 | S. hyicus | 2E-62 |
| 1083 | 1096325 | 1097131 | 269 | 133.9 | - | 744780879 | RecX family transcriptional regulator | S. hyicus | 1E-142 |
| 1084 | 1097221 | 1098027 | 269 | 125.3 | - | 744780878 | glycosyltransferase | S. hyicus | 2E-172 |
| 1085 | 1098195 | 1099940 | 582 | 303.8 | + | 744780877 | pyruvate oxidase | S. hyicus | 0E+00 |
| 1086 | 1100158 | 1100676 | 173 | 113.3 | - | 744780875 | PfpI family protein | S. hyicus | 5E-112 |
| 1087 | 1100744 | 1100905 | 54 | 30.5 | - | 744780874 | hypothetical protein SHYC_04445 | S. hyicus | 5E-29 |
| 1088 | 1101017 | 1102150 | 378 | 133 | + | 744780873 | hypothetical protein SHYC_04440 | S. hyicus | 0E+00 |
| 1089 | 1102292 | 1102810 | 173 | 86.2 | - | 744780872 | acyl-CoA hydrolase | S. hyicus | 2E-118 |
| 1090 | 1102823 | 1104058 | 412 | 232.9 | - | 744780871 | aminopeptidase PepS | S. hyicus | 0E+00 |
| 1091 | 1104075 | 1104284 | 70 | 41.1 | - | 744780870 | hypothetical protein SHYC_04425 | S. hyicus | 1E-31 |
| 1092 | 1104417 | 1104875 | 153 | 63.6 | + | 744780869 | protein tyrosine phosphatase | S. hyicus | 1E-104 |
| 1093 | 1104883 | 1105158 | 92 | 51.5 | + | 744780868 | hypothetical protein SHYC_04415 | S. hyicus | 2E-58 |
| 1094 | 1105274 | 1106545 | 424 | 199.3 | + | 744780867 | BrkB family protein | S. hyicus | 0E+00 |
| 1095 | 1106581 | 1107150 | 190 | 112.5 | + | 744780866 | carbonic anhydrase | S. hyicus | 1E-130 |
| 1096 | 1107216 | 1107845 | 210 | 129 | - | 744780865 | response regulator protein VraR | S. hyicus | 6E-145 |
| 1097 | 1107835 | 1108878 | 348 | 183.2 | - | 744780864 | sensor protein VraS | S. hyicus | 0E+00 |
| 1098 | 1108875 | 1109576 | 234 | 71.6 | - | 744780863 | hypothetical protein SHYC_04390 | S. hyicus | 3E-162 |
| 1099 | 1109589 | 1109975 | 129 | 46 | - | 746619020 | hypothetical protein | S. hyicus | 1E-63 |
| 1100 | 1110074 | 1110832 | 253 | 161.9 | - | 744780861 | methionine aminopeptidase | S. hyicus | 2E-177 |
| 1101 | 1111061 | 1111378 | 106 | 0.4 | + |  | No Significant Match |  |  |
| 1102 | 1111498 | 1112529 | 344 | 143.7 | + | 744780860 | membrane protein | S. hyicus | 0E+00 |
| 1103 | 1112619 | 1113350 | 244 | 141.6 | - | 744780859 | CobB/CobQ-like glutamine amidotransferase | S. hyicus | 3E-169 |
| 1104 | 1113351 | 1114661 | 437 | 239.7 | - | 744780858 | UDP-N-acetylmuramate--alanine ligase | S. hyicus | 0E+00 |
| 1105 | 1114809 | 1115312 | 168 | 94.9 | + | 744780857 | ferritin | S. hyicus | 1E-106 |
| 1106 | 1115411 | 1115971 | 187 | 68 | + | 744780856 | DNA polymerase III subunit epsilon | S. hyicus | 4E-126 |
| 1107 | 1115966 | 1117036 | 357 | 166.4 | - | 744780855 | DNA polymerase IV | S. hyicus | 0E+00 |
| 1108 | 1117195 | 1117734 | 180 | 53.4 | - | 744780854 | hypothetical protein SHYC_04345 | S. hyicus | 9E-94 |
| 1109 | 1117821 | 1119134 | 438 | 212.8 | - | 744780853 | carboxylesterase | S. hyicus | 0E+00 |
| 1110 | 1119147 | 1120517 | 457 | 204.2 | - | 744780852 | TrmA family RNA methyltransferase | S. hyicus | 0E+00 |
| 1111 | 1120613 | 1121545 | 311 | 191.8 | - | 744780851 | diacylglycerol kinase | S. hyicus | 0E+00 |
| 1112 | 1121786 | 1123213 | 476 | 327.6 | - | 744780850 | glutamyl-tRNA amidotransferase subunit B | S. hyicus | 0E+00 |
| 1113 | 1123227 | 1124687 | 487 | 314.5 | - | 744780849 | glutamyl-tRNA amidotransferase subunit A | S. hyicus | 0E+00 |
| 1114 | 1124690 | 1124992 | 101 | 72.9 | - | 744780848 | glutamyl-tRNA amidotransferase subunit C | S. hyicus | 3E-67 |
| 1115 | 1125390 | 1126934 | 515 | 278.4 | + | 744780847 | proline:sodium symporter PutP | S. hyicus | 0E+00 |
| 1116 | 1127079 | 1128275 | 399 | 232.5 | - | 744780846 | CamS precursor related lipoprotein | S. hyicus | 0E+00 |
| 1117 | 1128293 | 1130290 | 666 | 346.6 | - | 744780845 | NAD-dependent DNA ligase LigA | S. hyicus | 0E+00 |
| 1118 | 1130296 | 1132485 | 730 | 389.1 | - | 744780844 | ATP-dependent DNA helicase PcrA | S. hyicus | 0E+00 |
| 1119 | 1132485 | 1133174 | 230 | 114.3 | - | 744780843 | geranylgeranylglyceryl phosphate synthase | S. hyicus | 7E-152 |
| 1120 | 1133335 | 1133637 | 101 | 44.4 | - | 744780842 | Trp repressor family protein | S. hyicus | 2E-68 |
| 1121 | 1133708 | 1135003 | 432 | 257.8 | - | 744780841 | adenylosuccinate lyase | S. hyicus | 0E+00 |
| 1122 | 1135268 | 1135444 | 59 | 21.2 | - | 744780840 | NETI family protein | S. hyicus | 2E-29 |
| 1123 | 1135434 | 1136003 | 190 | 102.1 | - | 744780839 | membrane protein | S. hyicus | 2E-134 |
| 1124 | 1136070 | 1136897 | 276 | 174.7 | - | 744780838 | NAD synthetase | S. hyicus | 1E-177 |
| 1125 | 1136884 | 1138359 | 492 | 274.7 | - | 744780837 | nicotinate phosphoribosyltransferase | S. hyicus | 0E+00 |
| 1126 | 1138517 | 1139587 | 357 | 131.2 | + | 744780836 | nitric oxide synthase | S. hyicus | 0E+00 |
| 1127 | 1139632 | 1140426 | 265 | 126.4 | + | 744780835 | prephenate dehydratase | S. hyicus | 2E-164 |
| 1128 | 1140511 | 1141620 | 370 | 161.7 | - | 744780834 | hypothetical protein SHYC_04245 | S. hyicus | 0E+00 |
| 1129 | 1141703 | 1142254 | 184 | 89.9 | + | 744780833 | isochorismatase family protein | S. hyicus | 9E-130 |
| 1130 | 1142317 | 1143240 | 308 | 203.9 | + | 744780832 | manganese-dependent inorganic pyrophosphatase | S. hyicus | 0E+00 |
| 1131 | 1143261 | 1143542 | 94 | 48 | + | 744780831 | GNAT family acetyltransferase | S. hyicus | 7E-52 |
| 1132 | 1143613 | 1144995 | 461 | 200.1 | + | 744780830 | aldehyde dehydrogenase | S. hyicus | 0E+00 |
| 1133 | 1145105 | 1146154 | 350 | 179.3 | - | 744780829 | 6-phosphogluconolactonase | S. hyicus | 0E+00 |
| 1134 | 1146221 | 1146796 | 192 | 76.6 | - | 744780828 | hypothetical protein SHYC_04215 | S. hyicus | 4E-113 |
| 1135 | 1146915 | 1147088 | 58 | 32.8 | - | 744780827 | hypothetical protein SHYC_04210 | S. hyicus | 4E-31 |
| 1136 | 1147261 | 1147641 | 127 | 58.5 | + | 744780826 | hypothetical protein SHYC_04205 | S. hyicus | 2E-73 |
| 1137 | 1147909 | 1148949 | 347 | 136.3 | + | 744780825 | Ntn-choloylglycine hydrolase family protein | S. hyicus | 0E+00 |
| 1138 | 1149013 | 1149855 | 281 | 114.1 | - | 744780824 | hypothetical protein SHYC_04195 | S. hyicus | 1E-163 |
| 1139 | 1149852 | 1150415 | 188 | 84.2 | - | 744780823 | hypothetical protein SHYC_04190 | S. hyicus | 8E-109 |
| 1140 | 1150599 | 1151150 | 184 | 89.8 | + | 744780822 | thioredoxin-like protein | S. hyicus | 2E-118 |
| 1141 | 1151200 | 1151610 | 137 | 75.8 | - | 744780821 | hypothetical protein SHYC_04180 | S. hyicus | 2E-73 |
| 1142 | 1151661 | 1153022 | 454 | 200.8 | - | 744780820 | gluconate permease | S. hyicus | 0E+00 |
| 1143 | 1153012 | 1154583 | 524 | 214.2 | - | 744780819 | gluconate kinase | S. hyicus | 0E+00 |
| 1144 | 1154583 | 1155263 | 227 | 85.3 | - | 744780818 | gluconate operon transcriptional repressor | S. hyicus | 6E-145 |
| 1145 | 1155470 | 1156198 | 243 | 98.6 | - | 744780817 | membrane protein | S. hyicus | 2E-145 |
| 1146 | 1156195 | 1157070 | 292 | 141.3 | - | 744780816 | ABC transporter ATP-binding protein | S. hyicus | 0E+00 |
| 1147 | 1157638 | 1157814 | 59 | 10.4 | - | 744780815 | membrane protein | S. hyicus | 3E-27 |
| 1148 | 1157974 | 1159263 | 430 | 206.6 | + | 744780814 | aspartate aminotransferase | S. hyicus | 0E+00 |
| 1149 | 1159347 | 1160162 | 272 | 105.3 | + | 744780813 | DNA photolyase | S. hyicus | 3E-171 |
| 1150 | 1160177 | 1160701 | 175 | 76.6 | + | 744780812 | methylated-DNA--protein-cysteine methyltransferase | S. hyicus | 5E-68 |
| 1151 | 1160817 | 1161257 | 147 | 64.8 | - | 744780811 | ASC domain protein | S. hyicus | 5E-61 |
| 1152 | 1161264 | 1161368 | 35 | 2 | - |  | No Significant Match |  |  |
| 1153 | 1161694 | 1162203 | 170 | 68.1 | + | P0AAA1 | Inner membrane protein YagU | Escherichia coli | 3E-18 |
| 1154 | 1162666 | 1162899 | 78 | 47 | - | 686412286 | hypothetical protein | S. aureus | 3E-42 |
| 1155 | 1162989 | 1163297 | 103 | 37.5 | - |  | No Significant Match |  |  |
| 1156 | 1163684 | 1165327 | 548 | 202.6 | - | P59217 | Putative terminase large subunit | Shigella phage | 1E-56 |
| 1157 | 1165320 | 1165640 | 107 | 46.5 | - |  | No Significant Match |  |  |
| 1158 | 1165780 | 1166070 | 97 | 0.3 | - |  | No Significant Match |  |  |
| 1159 | 1166063 | 1166344 | 94 | 25.5 | - |  | No Significant Match |  |  |
| 1160 | 1166404 | 1167633 | 410 | 249.6 | - |  | No Significant Match |  |  |
| 1161 | 1167635 | 1168201 | 189 | 83.4 | - | P49860 | Putative prohead protease | Enterobacteria phage | 6E-07 |
| 1162 | 1168188 | 1169432 | 415 | 120.3 | - | P49859 | Portal protein | Enterobacteria phage | 5E-06 |
| 1163 | 1169480 | 1169812 | 111 | 37.1 | - |  | No Significant Match |  |  |
| 1164 | 1170123 | 1171571 | 483 | 168.8 | - | Q196T9 | Putative helicase 121R | Invertebrate iridescent | 3E-08 |
| 1165 | 1171564 | 1172466 | 301 | 83.5 | - |  | No Significant Match |  |  |
| 1166 | 1172498 | 1172794 | 99 | 49.1 | - |  | No Significant Match |  |  |
| 1167 | 1172794 | 1173207 | 138 | 75.6 | - |  | No Significant Match |  |  |
| 1168 | 1173209 | 1173412 | 68 | 29.3 | - |  | No Significant Match |  |  |
| 1169 | 1173409 | 1173714 | 102 | 40.9 | - |  | No Significant Match |  |  |
| 1170 | 1173715 | 1174344 | 210 | 101.7 | - | P44189 | Uncharacterized protein HI_1418 | Haemophilus influenzae | 2E-11 |
| 1171 | 1174348 | 1174566 | 73 | 26.8 | - | Q58564 | Putative HTH-type transcriptional regulatory protein MJ1164 | Methanocaldococcus jannaschii | 1E-05 |
| 1172 | 1174662 | 1175249 | 196 | 83.6 | + |  | No Significant Match |  |  |
| 1173 | 1175259 | 1176428 | 390 | 151.4 | + | P20709 | Integrase | S. phage L54a | 6E-29 |
| 1174 | 1176497 | 1178116 | 540 | 424.5 | - | 744780809 | molecular chaperone GroEL | S. hyicus | 0E+00 |
| 1175 | 1178181 | 1178468 | 96 | 70.9 | - | 744780808 | molecular chaperone GroES | S. hyicus | 6E-54 |
| 1176 | 1178639 | 1179391 | 251 | 94.9 | + | 744780807 | metal-dependent membrane protease | S. hyicus | 1E-155 |
| 1177 | 1179472 | 1180635 | 388 | 119.9 | - | 744780806 | hypothetical protein SHYC_04105 | S. hyicus | 1E-143 |
| 1178 | 1180806 | 1181603 | 266 | 115.3 | + | 744780805 | hydrolase | S. hyicus | 2E-159 |
| 1179 | 1182251 | 1182814 | 188 | 51.1 | + | 744780803 | accessory regulator protein B | S. hyicus | 4E-117 |
| 1180 | 1182817 | 1182951 | 45 | 6.9 | + | 744780802 | accessory gene regulator AgrD | S. hyicus | 1E-19 |
| 1181 | 1182981 | 1184279 | 433 | 142.4 | + | 744780801 | accessory gene regulator protein C | S. hyicus | 0E+00 |
| 1182 | 1184293 | 1185015 | 241 | 93.8 | + | 744780800 | accessory gene regulator protein A | S. hyicus | 1E-174 |
| 1183 | 1185082 | 1186572 | 497 | 235.4 | - | 744780799 | sucrose-6-phosphate hydrolase | S. hyicus | 0E+00 |
| 1184 | 1186925 | 1187152 | 76 | 48.5 | - | 744780798 | hypothetical protein SHYC_04065 | S. hyicus | 3E-47 |
| 1185 | 1187175 | 1188260 | 362 | 161.7 | - | 744780797 | membrane protein | S. hyicus | 0E+00 |
| 1186 | 1188443 | 1189078 | 212 | 127 | - | 744780796 | redox-sensing transcriptional repressor Rex | S. hyicus | 5E-148 |
| 1187 | 1189354 | 1191294 | 647 | 342.9 | + | 744780795 | ABC transporter ATP-binding protein | S. hyicus | 0E+00 |
| 1188 | 1191639 | 1193270 | 544 | 207.2 | + | 746621774 | DNA mismatch repair protein MutS, partial | S. hyicus | 0E+00 |
| 1189 | 1193287 | 1194309 | 341 | 177 | - | 744780794 | tRNA N6-adenosine threonylcarbamoyltransferase | S. hyicus | 0E+00 |
| 1190 | 1194306 | 1194770 | 155 | 74.1 | - | 744780793 | ribosomal-protein-alanine acetyltransferase | S. hyicus | 1E-90 |
| 1191 | 1194740 | 1195405 | 222 | 94.2 | - | 744780792 | tRNA threonylcarbamoyladenosine biosynthesis protein TsaB | S. hyicus | 5E-120 |
| 1192 | 1195398 | 1195847 | 150 | 77.2 | - | 744780791 | ATPase | S. hyicus | 8E-94 |
| 1193 | 1196275 | 1197963 | 563 | 322.5 | + | 744780790 | dihydroxy-acid dehydratase | S. hyicus | 0E+00 |
| 1194 | 1197981 | 1199735 | 585 | 314.3 | + | 744780789 | acetolactate synthase catalytic subunit | S. hyicus | 0E+00 |
| 1195 | 1199732 | 1200205 | 158 | 83.4 | + | 744780788 | acetolactate synthase small subunit | S. hyicus | 5E-93 |
| 1196 | 1200240 | 1201244 | 335 | 211.9 | + | 744780787 | ketol-acid reductoisomerase | S. hyicus | 0E+00 |
| 1197 | 1201272 | 1202807 | 512 | 270.2 | + | 744780786 | 2-isopropylmalate synthase | S. hyicus | 0E+00 |
| 1198 | 1202800 | 1203849 | 350 | 149.3 | + | 744780785 | 3-isopropylmalate dehydrogenase | S. hyicus | 0E+00 |
| 1199 | 1203852 | 1205228 | 459 | 250.7 | + | 744780784 | 3-isopropylmalate dehydratase large subunit | S. hyicus | 0E+00 |
| 1200 | 1205225 | 1205806 | 194 | 80.6 | + | 744780783 | isopropylmalate isomerase | S. hyicus | 6E-117 |
| 1201 | 1205812 | 1207080 | 423 | 200.2 | + | 744780782 | threonine dehydratase | S. hyicus | 0E+00 |
| 1202 | 1213374 | 1215539 | 722 | 383.5 | - | 744780780 | S1 RNA-binding domain-containing transcription accessory protein | S. hyicus | 0E+00 |
| 1203 | 1215761 | 1216531 | 257 | 159.6 | - | 744780779 | RNA polymerase sigma factor SigB | S. hyicus | 0E+00 |
| 1204 | 1216506 | 1216985 | 160 | 91.1 | - | 744780778 | serine-protein kinase RsbW | S. hyicus | 1E-110 |
| 1205 | 1216988 | 1217314 | 109 | 59.5 | - | 744780777 | anti-sigma-B factor antagonist | S. hyicus | 7E-71 |
| 1206 | 1217395 | 1218399 | 335 | 155.2 | - | 744780776 | phosphoserine phosphatase RsbU | S. hyicus | 0E+00 |
| 1207 | 1218459 | 1218812 | 118 | 57.5 | - | 744780775 | mRNA interferase MazF | S. hyicus | 2E-79 |
| 1208 | 1218813 | 1218983 | 57 | 18.7 | - | 744780774 | antitoxin MazE | S. hyicus | 8E-32 |
| 1209 | 1219068 | 1220216 | 383 | 162 | - | 744780773 | alanine racemase | S. hyicus | 0E+00 |
| 1210 | 1220322 | 1220672 | 117 | 69.7 | - | 744780772 | 4'-phosphopantetheinyl transferase | S. hyicus | 1E-73 |
| 1211 | 1220744 | 1221250 | 169 | 54.2 | - | 744780771 | membrane protein | S. hyicus | 3E-85 |
| 1212 | 1221234 | 1222721 | 496 | 170.5 | - | 744780770 | hypothetical protein SHYC_03895 | S. hyicus | 0E+00 |
| 1213 | 1222714 | 1223190 | 159 | 46.6 | - | 744780769 | hypothetical protein SHYC_03890 | S. hyicus | 7E-81 |
| 1214 | 1223674 | 1225161 | 496 | 306.8 | - | 744780768 | RNA helicase | S. hyicus | 0E+00 |
| 1215 | 1225469 | 1226839 | 457 | 251.1 | - | 744780767 | UDP-N-acetylmuramoylalanyl-D-glutamyl-2, 6-diaminopimelate--D-alanyl-D-alanine ligase | S. hyicus | 0E+00 |
| 1216 | 1226851 | 1227921 | 357 | 202.8 | - | 744780766 | D-alanine--D-alanine ligase | S. hyicus | 0E+00 |
| 1217 | 1228086 | 1229285 | 400 | 146.2 | + | 744780765 | cell division protein FtsW | S. hyicus | 0E+00 |
| 1218 | 1229440 | 1229595 | 52 | 2.9 | - | 744780764 | hypothetical protein SHYC_03865 | S. hyicus | 2E-29 |
| 1219 | 1229665 | 1231845 | 727 | 382.4 | - | 744780763 | copper-exporting P-type ATPase A | S. hyicus | 0E+00 |
| 1220 | 1231970 | 1232173 | 68 | 50 | - | 744780762 | copper chaperone CopZ | S. hyicus | 1E-37 |
| 1221 | 1232166 | 1232399 | 78 | 44.6 | - | 744780761 | hypothetical protein SHYC_03850 | S. hyicus | 4E-32 |
| 1222 | 1232412 | 1232699 | 96 | 42.9 | - | 744780760 | copper-sensing transcriptional repressor CsoR | S. hyicus | 6E-62 |
| 1223 | 1232835 | 1234325 | 497 | 199.8 | + | 744780759 | cardiolipin synthase | S. hyicus | 0E+00 |
| 1224 | 1234338 | 1234970 | 211 | 98.2 | + | 744780758 | HD superfamily hydrolase | S. hyicus | 2E-118 |
| 1225 | 1235018 | 1235884 | 289 | 120.9 | - | 744780757 | preprotein translocase subunit YidC | S. hyicus | 8E-168 |
| 1226 | 1235973 | 1236611 | 213 | 97.5 | - | 744780756 | thiamine-phosphate pyrophosphorylase | S. hyicus | 3E-139 |
| 1227 | 1236608 | 1237405 | 266 | 135.6 | - | 744780755 | hydroxyethylthiazole kinase | S. hyicus | 2E-167 |
| 1228 | 1237407 | 1238213 | 269 | 133.9 | - | 744780754 | phosphomethylpyrimidine kinase | S. hyicus | 2E-174 |
| 1229 | 1238206 | 1238895 | 230 | 122.2 | - | 744780753 | thiaminase | S. hyicus | 5E-135 |
| 1230 | 1239145 | 1239888 | 248 | 157.3 | - | 744780752 | hypothetical protein SHYC_03805 | S. hyicus | 1E-162 |
| 1231 | 1240502 | 1240864 | 121 | 6.3 | - | 744780751 | single-stranded DNA-binding protein | S. hyicus | 1E-55 |
| 1232 | 1241054 | 1241494 | 147 | 81.8 | + | 744780750 | YwpF family protein | S. hyicus | 2E-99 |
| 1233 | 1241726 | 1242166 | 147 | 84.6 | - | 744780749 | 3-hydroxyacyl-ACP dehydratase | S. hyicus | 9E-101 |
| 1234 | 1242356 | 1243627 | 424 | 282.6 | - | 744780747 | UDP-N-acetylglucosamine 1-carboxyvinyltransferase | S. hyicus | 0E+00 |
| 1235 | 1243710 | 1243943 | 78 | 13.3 | - | 744780746 | membrane protein | S. hyicus | 2E-44 |
| 1236 | 1244194 | 1244598 | 135 | 94.7 | - | 744780745 | F0F1 ATP synthase subunit epsilon | S. hyicus | 2E-93 |
| 1237 | 1244620 | 1246032 | 471 | 345.9 | - | 744780744 | F0F1 ATP synthase subunit beta | S. hyicus | 0E+00 |
| 1238 | 1246057 | 1246923 | 289 | 149.5 | - | 744780743 | F0F1 ATP synthase subunit gamma | S. hyicus | 0E+00 |
| 1239 | 1246958 | 1248466 | 503 | 342.7 | - | 744780742 | F0F1 ATP synthase subunit alpha | S. hyicus | 0E+00 |
| 1240 | 1248494 | 1249033 | 180 | 87.6 | - | 744780741 | F0F1 ATP synthase subunit delta | S. hyicus | 4E-120 |
| 1241 | 1249033 | 1249551 | 173 | 108.6 | - | 744780740 | F0F1 ATP synthase subunit B | S. hyicus | 4E-121 |
| 1242 | 1249849 | 1250061 | 71 | 55 | - | 744780739 | F0F1 ATP synthase subunit C | S. hyicus | 6E-39 |
| 1243 | 1250101 | 1250820 | 240 | 92.1 | - | 744780738 | F0F1 ATP synthase subunit A | S. hyicus | 9E-171 |
| 1244 | 1250839 | 1251204 | 122 | 15.6 | - | 744780737 | ATP synthase I chain | S. hyicus | 3E-79 |
| 1245 | 1251363 | 1252502 | 380 | 219.3 | - | 744780736 | UDP-N-acetylglucosamine 2-epimerase | S. hyicus | 0E+00 |
| 1246 | 1252529 | 1253158 | 210 | 152.2 | - | 744780735 | uracil phosphoribosyltransferase | S. hyicus | 3E-149 |
| 1247 | 1253181 | 1254422 | 414 | 246.9 | - | 744780734 | serine hydroxymethyltransferase | S. hyicus | 0E+00 |
| 1248 | 1254445 | 1254972 | 176 | 98.1 | - | 744780733 | hypothetical protein SHYC_03710 | S. hyicus | 2E-117 |
| 1249 | 1255099 | 1255518 | 140 | 65.4 | - | 744780732 | low molecular weight protein-tyrosine-phosphatase PtpB | S. hyicus | 4E-76 |
| 1250 | 1255515 | 1256567 | 351 | 161.7 | - | 744780731 | Sua5/YciO/YrdC/YwlC family protein | S. hyicus | 0E+00 |
| 1251 | 1256651 | 1257493 | 281 | 126 | - | 744780730 | release factor-specific (glutamine-N5) methyltransferase | S. hyicus | 1E-137 |
| 1252 | 1257483 | 1258559 | 359 | 241.4 | - | 744780729 | peptide chain release factor 1 | S. hyicus | 0E+00 |
| 1253 | 1258559 | 1259161 | 201 | 104.8 | - | 744780728 | thymidine kinase | S. hyicus | 2E-146 |
| 1254 | 1259334 | 1259588 | 85 | 40.7 | - | 744780727 | 50S ribosomal protein L31 type B | S. hyicus | 2E-57 |
| 1255 | 1259710 | 1261026 | 439 | 227.9 | - | 744780726 | transcription termination factor Rho | S. hyicus | 0E+00 |
| 1256 | 1261205 | 1262632 | 476 | 320.4 | - | 744780725 | aldehyde dehydrogenase | S. hyicus | 0E+00 |
| 1257 | 1262816 | 1263142 | 109 | 55.8 | - | 744780724 | transcriptional regulator | S. hyicus | 4E-69 |
| 1258 | 1263188 | 1264453 | 422 | 254 | - | 744780723 | UDP-N-acetylglucosamine 1-carboxyvinyltransferase | S. hyicus | 0E+00 |
| 1259 | 1264779 | 1265651 | 291 | 197.2 | - | 744780722 | fructose-bisphosphate aldolase | S. hyicus | 0E+00 |
| 1260 | 1265867 | 1266388 | 174 | 84.2 | + | 744780721 | hypothetical protein SHYC_03650 | S. hyicus | 2E-111 |
| 1261 | 1266577 | 1268184 | 536 | 337.8 | - | 744780720 | CTP synthetase | S. hyicus | 0E+00 |
| 1262 | 1268533 | 1269063 | 177 | 135 | - | 744780719 | DNA-directed RNA polymerase subunit delta | S. hyicus | 4E-87 |
| 1263 | 1269219 | 1270079 | 287 | 137.3 | - | 744780718 | acetyltransferase | S. hyicus | 0E+00 |
| 1264 | 1270168 | 1271766 | 533 | 194.7 | + | 744780717 | betaine/carnitine/choline transporter (BCCT) family transporter | S. hyicus | 0E+00 |
| 1265 | 1271878 | 1272246 | 123 | 59.2 | - | 744780716 | hypothetical protein SHYC_03625 | S. hyicus | 2E-72 |
| 1266 | 1272354 | 1273151 | 266 | 156 | + | 744780715 | pantothenate kinase | S. hyicus | 1E-171 |
| 1267 | 1273233 | 1273904 | 224 | 124.4 | - | 744780714 | hypothetical protein SHYC_03615 | S. hyicus | 2E-159 |
| 1268 | 1274060 | 1275241 | 394 | 222.8 | - | 744780713 | hypothetical protein SHYC_03610 | S. hyicus | 0E+00 |
| 1269 | 1275242 | 1276426 | 395 | 208.2 | - | 744780712 | amidohydrolase | S. hyicus | 0E+00 |
| 1270 | 1276858 | 1277328 | 157 | 92.9 | + | 744780711 | S-ribosylhomocysteinase | S. hyicus | 3E-107 |
| 1271 | 1277713 | 1278066 | 118 | 29 | - | 744780710 | membrane protein | S. hyicus | 1E-60 |
| 1272 | 1278076 | 1279377 | 434 | 323.1 | - | 744780709 | pyrimidine-nucleoside phosphorylase | S. hyicus | 0E+00 |
| 1273 | 1279583 | 1280293 | 237 | 156.6 | + | 744780708 | purine nucleoside phosphorylase | S. hyicus | 9E-175 |
| 1274 | 1280421 | 1280846 | 142 | 39 | - | 744780707 | hypothetical protein SHYC_03580 | S. hyicus | 3E-92 |
| 1275 | 1280959 | 1282386 | 476 | 212.9 | - | 744780706 | Na+/H+ antiporter NhaC | S. hyicus | 0E+00 |
| 1276 | 1282745 | 1284115 | 457 | 259.8 | - | 744780705 | hypothetical protein SHYC_03570 | S. hyicus | 0E+00 |
| 1277 | 1284160 | 1284402 | 81 | 29.7 | - | 744780704 | membrane protein | S. hyicus | 2E-40 |
| 1278 | 1284418 | 1285356 | 313 | 167.4 | - | 744780703 | mannose-6-phosphate isomerase | S. hyicus | 0E+00 |
| 1279 | 1285520 | 1286218 | 233 | 126 | - | 744780702 | transcriptional regulator | S. hyicus | 5E-157 |
| 1280 | 1286436 | 1287323 | 296 | 83.8 | + | 744780701 | hypothetical protein SHYC_03550 | S. hyicus | 2E-109 |
| 1281 | 1287399 | 1288250 | 284 | 131.5 | - | 744780700 | HAD family hydrolase | S. hyicus | 0E+00 |
| 1282 | 1288330 | 1289109 | 260 | 117.1 | - | 744780699 | ABC transporter ATP-binding protein | S. hyicus | 6E-175 |
| 1283 | 1289214 | 1291016 | 601 | 407.9 | - | 744780698 | glucosamine--fructose-6-phosphate aminotransferase | S. hyicus | 0E+00 |
| 1284 | 1291551 | 1292900 | 450 | 316.1 | - | 744780697 | phosphoglucosamine mutase | S. hyicus | 0E+00 |
| 1285 | 1292927 | 1293859 | 311 | 163.4 | - | 744780696 | hypothetical protein SHYC_03525 | S. hyicus | 0E+00 |
| 1286 | 1293860 | 1294666 | 269 | 147.4 | - | 744780695 | DisA domain protein | S. hyicus | 0E+00 |
| 1287 | 1294830 | 1295735 | 302 | 184.3 | - | 744780694 | arginase | S. hyicus | 0E+00 |
| 1288 | 1302329 | 1303393 | 355 | 225.2 | - | 744780693 | ATP-binding protein | S. hyicus | 0E+00 |
| 1289 | 1303496 | 1304926 | 477 | 185.2 | - | 744780692 | MFS transporter | S. hyicus | 0E+00 |
| 1290 | 1305266 | 1305733 | 156 | 39 | - | 744780691 | hypothetical protein SHYC_03455 | S. hyicus | 4E-101 |
| 1291 | 1305778 | 1307097 | 440 | 131 | - | 744780690 | MFS transporter | S. hyicus | 0E+00 |
| 1292 | 1307198 | 1307395 | 66 | 10.6 | - | 744780689 | hypothetical protein SHYC_03447 | S. hyicus | 5E-17 |
| 1293 | 1307408 | 1308094 | 229 | 69.3 | - | 744780688 | hemolysin III family protein | S. hyicus | 3E-152 |
| 1294 | 1308105 | 1309295 | 397 | 234.3 | - | 744780687 | putative UDP-N-acetylglucosamine pyrophosphorylase | S. hyicus | 0E+00 |
| 1295 | 1309345 | 1309848 | 168 | 51.3 | - | 744780686 | membrane protein | S. hyicus | 1E-105 |
| 1296 | 1310036 | 1310296 | 87 | 40.1 | + | 744780685 | hypothetical protein SHYC_03430 | S. hyicus | 2E-55 |
| 1297 | 1310439 | 1311401 | 321 | 144.6 | - | 744780684 | iron compound ABC transporter permease | S. hyicus | 0E+00 |
| 1298 | 1311398 | 1312423 | 342 | 135.8 | - | 744780683 | iron compound ABC transporter permease | S. hyicus | 0E+00 |
| 1299 | 1312478 | 1313464 | 329 | 213.9 | - | 744780682 | iron compound ABC transporter substrate-binding protein | S. hyicus | 0E+00 |
| 1300 | 1313530 | 1314600 | 357 | 171.2 | - | 744780681 | amino acid racemase | S. hyicus | 0E+00 |
| 1301 | 1314604 | 1316367 | 588 | 252.9 | - | 744780680 | siderophore synthetase component | S. hyicus | 0E+00 |
| 1302 | 1316354 | 1317535 | 394 | 90.7 | - | 744780679 | major facilitator family transporter | S. hyicus | 0E+00 |
| 1303 | 1317637 | 1319583 | 649 | 235.1 | + | 744780678 | IucA/IucC family siderophore biosynthesis protein | S. hyicus | 0E+00 |
| 1304 | 1319821 | 1320969 | 383 | 166.3 | + | 744780677 | major facilitator family transporter | S. hyicus | 0E+00 |
| 1305 | 1321138 | 1322664 | 509 | 191.6 | - | 744780676 | betaine/carnitine/choline transporter (BCCT) family transporter | S. hyicus | 0E+00 |
| 1306 | 1322736 | 1323743 | 336 | 164.1 | - | 744780675 | zinc-binding alcohol dehydrogenase family protein | S. hyicus | 0E+00 |
| 1307 | 1323911 | 1325314 | 468 | 278.5 | - | 744780674 | 6-phospho-beta-galactosidase | S. hyicus | 0E+00 |
| 1308 | 1325330 | 1327057 | 576 | 334.4 | - | 744780673 | PTS system lactose-specific transporter subunits IICB | S. hyicus | 0E+00 |
| 1309 | 1327063 | 1327377 | 105 | 79.9 | - | 744780672 | PTS system lactose-specific transporter subunit IIA | S. hyicus | 9E-70 |
| 1310 | 1327417 | 1328382 | 322 | 225.1 | - | 744780671 | tagatose-bisphosphate aldolase | S. hyicus | 0E+00 |
| 1311 | 1328489 | 1329004 | 172 | 110.6 | - | 744780670 | galactose-6-phosphate isomerase subunit LacB | S. hyicus | 5E-121 |
| 1312 | 1329020 | 1329448 | 143 | 80 | - | 744780669 | galactose-6-phosphate isomerase subunit LacA | S. hyicus | 2E-97 |
| 1313 | 1329721 | 1330473 | 251 | 104.9 | - | 744780668 | lactose phosphotransferase system repressor | S. hyicus | 2E-173 |
| 1314 | 1330655 | 1331233 | 193 | 101.4 | - | 744780667 | hypothetical protein SHYC_03340 | S. hyicus | 3E-115 |
| 1315 | 1331505 | 1332200 | 232 | 107.8 | - | 744780666 | hydrolase | S. hyicus | 3E-139 |
| 1316 | 1332308 | 1332934 | 209 | 91 | - | Q8GXU8 | 1-acyl-sn-glycerol-3-phosphate acyltransferase 1, chloroplastic | Arabidopsis thaliana | 2E-09 |
| 1317 | 1332952 | 1333890 | 313 | 113 | - | P76091 | Uncharacterized protein YnbB | Escherichia coli | 6E-77 |
| 1318 | 1333877 | 1334494 | 206 | 90.7 | - | P76090 | Inner membrane protein YnbA | Escherichia coli | 4E-36 |
| 1319 | 1334610 | 1335146 | 179 | 59.3 | + |  | No Significant Match |  |  |
| 1320 | 1335302 | 1336063 | 254 | 111.3 | + | 744780665 | SAM-dependent methyltransferase | S. hyicus | 2E-178 |
| 1321 | 1336134 | 1336862 | 243 | 97.4 | - | 744780664 | MerR family transcriptional regulator | S. hyicus | 3E-159 |
| 1322 | 1336841 | 1337746 | 302 | 118.5 | - | 744780663 | metallopeptidase | S. hyicus | 0E+00 |
| 1323 | 1337904 | 1338302 | 133 | 89.5 | - | 744780662 | 30S ribosomal protein S9 | S. hyicus | 1E-89 |
| 1324 | 1338316 | 1338753 | 146 | 87.4 | - | 744780661 | 50S ribosomal protein L13 | S. hyicus | 1E-104 |
| 1325 | 1338942 | 1339772 | 277 | 99.1 | - | 744780660 | tRNA pseudouridine synthase A | S. hyicus | 0E+00 |
| 1326 | 1339787 | 1340590 | 268 | 68.8 | - | 744780659 | cobalt ABC transporter permease | S. hyicus | 2E-163 |
| 1327 | 1340580 | 1341446 | 289 | 136.2 | - | 746618707 | ATP-binding protein | S. hyicus | 0E+00 |
| 1328 | 1341443 | 1342246 | 268 | 151.2 | - | 744780657 | cobalt transporter ATP-binding subunit | S. hyicus | 4E-177 |
| 1329 | 1342639 | 1343007 | 123 | 96.7 | - | 744780656 | 50S ribosomal protein L17 | S. hyicus | 3E-80 |
| 1330 | 1343024 | 1343968 | 315 | 206.3 | - | 744780655 | DNA-directed RNA polymerase subunit alpha | S. hyicus | 0E+00 |
| 1331 | 1344052 | 1344441 | 130 | 82.2 | - | 744780654 | 30S ribosomal protein S11 | S. hyicus | 1E-89 |
| 1332 | 1344466 | 1344831 | 122 | 83.1 | - | 744780653 | 30S ribosomal protein S13 | S. hyicus | 3E-80 |
| 1333 | 1344854 | 1344967 | 38 | 10.1 | - | Q18CI1 | 50S ribosomal protein L36 | Clostridium difficile | 2E-17 |
| 1334 | 1345000 | 1345218 | 73 | 50.7 | - | Q49ZE6 | Translation initiation factor IF-1 | S. saprophyticus | 1E-46 |
| 1335 | 1345386 | 1346036 | 217 | 141.9 | - | 744780650 | adenylate kinase | S. hyicus | 1E-154 |
| 1336 | 1346054 | 1347346 | 431 | 223.7 | - | 744780649 | preprotein translocase subunit SecY | S. hyicus | 0E+00 |
| 1337 | 1347346 | 1347786 | 147 | 113.5 | - | 744780648 | 50S ribosomal protein L15 | S. hyicus | 4E-99 |
| 1338 | 1347803 | 1347982 | 60 | 40.1 | - | 744780647 | 50S ribosomal protein L30 | S. hyicus | 4E-34 |
| 1339 | 1348000 | 1348500 | 167 | 130.5 | - | 744780646 | 30S ribosomal protein S5 | S. hyicus | 1E-113 |
| 1340 | 1348522 | 1348881 | 120 | 69.2 | - | 744780645 | 50S ribosomal protein L18 | S. hyicus | 4E-80 |
| 1341 | 1348912 | 1349448 | 179 | 131.8 | - | 744780644 | 50S ribosomal protein L6 | S. hyicus | 2E-123 |
| 1342 | 1349478 | 1349876 | 133 | 97.8 | - | 744780643 | 30S ribosomal protein S8 | S. hyicus | 3E-90 |
| 1343 | 1349908 | 1350093 | 62 | 17.7 | - | 744780642 | 30S ribosomal protein S14 | S. hyicus | 1E-37 |
| 1344 | 1350115 | 1350654 | 180 | 110.4 | - | 744780641 | 50S ribosomal protein L5 | S. hyicus | 9E-127 |
| 1345 | 1350679 | 1350996 | 106 | 76 | - | 744780640 | 50S ribosomal protein L24 | S. hyicus | 3E-67 |
| 1346 | 1351035 | 1351403 | 123 | 89.7 | - | 744780639 | 50S ribosomal protein L14 | S. hyicus | 1E-80 |
| 1347 | 1351434 | 1351697 | 88 | 41.7 | - | 744780638 | 30S ribosomal protein S17 | S. hyicus | 2E-55 |
| 1348 | 1351722 | 1351931 | 70 | 36.3 | - | 744780637 | 50S ribosomal protein L29 | S. hyicus | 3E-40 |
| 1349 | 1351921 | 1352355 | 145 | 100.5 | - | 744780636 | 50S ribosomal protein L16 | S. hyicus | 2E-100 |
| 1350 | 1352358 | 1353011 | 218 | 137.6 | - | 744780635 | 30S ribosomal protein S3 | S. hyicus | 1E-155 |
| 1351 | 1353034 | 1353387 | 118 | 68.2 | - | 744780634 | 50S ribosomal protein L22 | S. hyicus | 6E-79 |
| 1352 | 1353420 | 1353698 | 93 | 45.6 | - | 744780633 | 30S ribosomal protein S19 | S. hyicus | 2E-62 |
| 1353 | 1353763 | 1354596 | 278 | 184 | - | 744780632 | 50S ribosomal protein L2 | S. hyicus | 0E+00 |
| 1354 | 1354626 | 1354901 | 92 | 55.4 | - | 744780631 | 50S ribosomal protein L23 | S. hyicus | 2E-59 |
| 1355 | 1354901 | 1355524 | 208 | 133.9 | - | 744780630 | 50S ribosomal protein L4 | S. hyicus | 1E-148 |
| 1356 | 1355551 | 1356210 | 220 | 135.7 | - | 744780629 | 50S ribosomal protein L3 | S. hyicus | 2E-157 |
| 1357 | 1356238 | 1356546 | 103 | 63.4 | - | P66336 | 30S ribosomal protein S10 | S. epidermidis | 1E-68 |
| 1358 | 1357002 | 1358336 | 445 | 224.8 | - | 744780627 | permease | S. hyicus | 0E+00 |
| 1359 | 1358450 | 1360582 | 711 | 340.4 | - | 744780626 | DNA topoisomerase III | S. hyicus | 0E+00 |
| 1360 | 1360704 | 1361564 | 287 | 123.3 | + | 744780625 | GNAT family acetyltransferase | S. hyicus | 0E+00 |
| 1361 | 1361679 | 1362596 | 306 | 97.7 | - | 744780624 | transporter | S. hyicus | 6E-176 |
| 1362 | 1362721 | 1362921 | 67 | 43 | + | 744780623 | hypothetical protein SHYC_03115 | S. hyicus | 5E-24 |
| 1363 | 1362941 | 1363258 | 106 | 32.7 | + | 744780622 | membrane protein | S. hyicus | 1E-49 |
| 1364 | 1363371 | 1364525 | 385 | 188.4 | - | 744780621 | N-succinyldiaminopimelate aminotransferase | S. hyicus | 0E+00 |
| 1365 | 1364722 | 1367883 | 1054 | 483 | - | 744780620 | RND efflux transporter | S. hyicus | 0E+00 |
| 1366 | 1368034 | 1369284 | 417 | 227.6 | - | 744780619 | FemAB family protein | S. hyicus | 0E+00 |
| 1367 | 1369402 | 1369656 | 85 | 36.3 | + | 744780618 | hypothetical protein SHYC_03090 | S. hyicus | 4E-31 |
| 1368 | 1369653 | 1370414 | 254 | 117.5 | - | 744780617 | hypothetical protein SHYC_03085 | S. hyicus | 8E-176 |
| 1369 | 1370585 | 1371034 | 150 | 61.8 | + | 744780616 | MarR family transcriptional regulator | S. hyicus | 2E-96 |
| 1370 | 1371353 | 1373191 | 613 | 291.6 | + | 744780615 | endonuclease/exonuclease/phosphatase family protein | S. hyicus | 0E+00 |
| 1371 | 1373362 | 1374384 | 341 | 160.3 | - | 744780614 | molybdenum cofactor biosynthesis protein A | S. hyicus | 0E+00 |
| 1372 | 1374405 | 1375007 | 201 | 86.2 | - | 744780613 | molybdopterin-guanine dinucleotide biosynthesis protein A | S. hyicus | 6E-121 |
| 1373 | 1375017 | 1375250 | 78 | 40.7 | - | 744780612 | molybdopterin synthase small subunit | S. hyicus | 2E-45 |
| 1374 | 1375247 | 1375702 | 152 | 67.3 | - | 744780611 | molybdopterin synthase subunit 2 | S. hyicus | 3E-101 |
| 1375 | 1375704 | 1376183 | 160 | 85.8 | - | 744780610 | molybdopterin-guanine dinucleotide biosynthesis protein B | S. hyicus | 2E-91 |
| 1376 | 1376180 | 1377439 | 420 | 229.6 | - | 744780609 | molybdopterin molybdenumtransferase | S. hyicus | 0E+00 |
| 1377 | 1377507 | 1377995 | 163 | 78.5 | + | 744780608 | molybdenum cofactor biosynthesis protein C | S. hyicus | 4E-110 |
| 1378 | 1378087 | 1378587 | 167 | 81.2 | - | 744780607 | molybdenum cofactor biosynthesis protein B | S. hyicus | 2E-106 |
| 1379 | 1378599 | 1379603 | 335 | 142.4 | - | 746621756 | molybdopterin biosynthesis protein MoeB | S. hyicus | 0E+00 |
| 1380 | 1379871 | 1380485 | 205 | 81.7 | - | 744780605 | molybdenum ABC transporter ATP-binding protein | S. hyicus | 5E-118 |
| 1381 | 1380486 | 1381157 | 224 | 80 | - | 744780604 | molybdenum ABC transporter permease | S. hyicus | 2E-146 |
| 1382 | 1381169 | 1381921 | 251 | 124.1 | - | 744780603 | molybdenum ABC transporter substrate-binding protein | S. hyicus | 1E-141 |
| 1383 | 1382101 | 1382898 | 266 | 110.6 | + | 744780602 | formate dehydrogenase family accessory protein FdhD | S. hyicus | 6E-180 |
| 1384 | 1383020 | 1383775 | 252 | 123.8 | - | 744780601 | hypothetical protein SHYC_03005 | S. hyicus | 9E-133 |
| 1385 | 1383798 | 1384361 | 188 | 84.8 | - | 744780600 | BioY family protein | S. hyicus | 6E-115 |
| 1386 | 1384483 | 1385343 | 287 | 125.3 | - | 744780599 | N-acetylmannosamine kinase | S. hyicus | 3E-154 |
| 1387 | 1385324 | 1385980 | 219 | 97.7 | - | 744780598 | transcriptional regulator | S. hyicus | 6E-136 |
| 1388 | 1386140 | 1387021 | 294 | 184.2 | + | 744780597 | N-acetylneuraminate lyase | S. hyicus | 0E+00 |
| 1389 | 1387091 | 1388605 | 505 | 199.3 | + | 744780596 | putative sialic acid transporter | S. hyicus | 0E+00 |
| 1390 | 1388809 | 1389726 | 306 | 193.1 | - | 744780595 | iron compound ABC transporter substrate-binding lipoprotein | S. hyicus | 0E+00 |
| 1391 | 1390010 | 1390402 | 131 | 46.6 | - | 744780594 | hypothetical protein SHYC_02970 | S. hyicus | 1E-74 |
| 1392 | 1390886 | 1391302 | 139 | 44.9 | - |  | No Significant Match |  |  |
| 1393 | 1391427 | 1391849 | 141 | 28.6 | - |  | No Significant Match |  |  |
| 1394 | 1392067 | 1393329 | 421 | 94.1 | - | Q57772 | Putative permease MJ0326 | Methanocaldococcus jannaschii | 5E-69 |
| 1395 | 1393319 | 1393600 | 94 | 0.3 | - |  | No Significant Match |  |  |
| 1396 | 1393581 | 1394492 | 304 | 85.8 | - | O34926 | Putative cytochrome P450 CypX | Bacillus subtilis | 3E-06 |
| 1397 | 1394511 | 1395254 | 248 | 78.5 | - |  | No Significant Match |  |  |
| 1398 | 1395683 | 1396189 | 169 | 70.6 | - | P10548 | Lysostaphin | S. staphylolyticus | 6E-20 |
| 1399 | 1396301 | 1396696 | 132 | 28.6 | - | P24556 | Autolysin | S. aureus | 6E-21 |
| 1400 | 1396653 | 1396913 | 87 | 30.4 | - | O34433 | Putative phage-related protein YobO | Bacillus subtilis | 4E-13 |
| 1401 | 1397188 | 1397535 | 116 | 45.3 | - | 744780592 | MarR family transcriptional regulator | S. hyicus | 5E-67 |
| 1402 | 1397799 | 1398152 | 118 | 85.3 | - | 744780591 | hypothetical protein SHYC_02955 | S. hyicus | 1E-64 |
| 1403 | 1398177 | 1399226 | 350 | 190.5 | - | 744780590 | LLM family oxidoreductase | S. hyicus | 0E+00 |
| 1404 | 1399630 | 1399953 | 108 | 61 | - | 744780589 | hypothetical protein SHYC_02945 | S. hyicus | 3E-58 |
| 1405 | 1400415 | 1401143 | 243 | 104.1 | + | 744780588 | hypothetical protein SHYC_02940 | S. hyicus | 8E-138 |
| 1406 | 1401186 | 1401659 | 158 | 26.3 | - | 744780587 | hypothetical protein SHYC_02935 | S. hyicus | 2E-86 |
| 1407 | 1401766 | 1403175 | 470 | 202.9 | - | 744780586 | sodium:proton antiporter | S. hyicus | 0E+00 |
| 1408 | 1403195 | 1404271 | 359 | 197.9 | - | 744780585 | NAD/NADP octopine/nopaline dehydrogenase family protein | S. hyicus | 0E+00 |
| 1409 | 1404467 | 1404847 | 127 | 47.1 | + | 744780584 | membrane protein | S. hyicus | 2E-63 |
| 1410 | 1405258 | 1405827 | 190 | 98.2 | + | 744780583 | hypothetical protein SHYC_02915 | S. hyicus | 1E-101 |
| 1411 | 1405993 | 1406949 | 319 | 203 | + | 744780582 | 2-hydroxyacid dehydrogenase | S. hyicus | 0E+00 |
| 1412 | 1407010 | 1408134 | 375 | 201.9 | - | 744780581 | hypothetical protein SHYC_02905 | S. hyicus | 0E+00 |
| 1413 | 1408193 | 1408975 | 261 | 138.1 | - | 744780580 | glucosaminidase family protein | S. hyicus | 7E-149 |
| 1414 | 1409006 | 1409248 | 81 | 30.6 | - | 744780579 | membrane protein | S. hyicus | 3E-21 |
| 1415 | 1409521 | 1409985 | 155 | 93.5 | - | 744780578 | hypothetical protein SHYC_02890 | S. hyicus | 7E-104 |
| 1416 | 1409985 | 1412921 | 979 | 502.2 | - | 744780577 | putative formate dehydrogenase | S. hyicus | 0E+00 |
| 1417 | 1413098 | 1413739 | 214 | 83.5 | - | 744780576 | pyrrolidone-carboxylate peptidase | S. hyicus | 6E-101 |
| 1418 | 1413832 | 1414785 | 318 | 176.1 | - | 744780575 | LytR family transcriptional regulator | S. hyicus | 0E+00 |
| 1419 | 1414894 | 1415685 | 264 | 132.6 | - | 744780574 | inositol monophosphatase family protein | S. hyicus | 1E-179 |
| 1420 | 1416169 | 1416864 | 232 | 103.1 | + | 744780573 | DeoR faimly transcriptional regulator | S. hyicus | 2E-138 |
| 1421 | 1417087 | 1417818 | 244 | 47.3 | - | 744780572 | putative metal-dependent membrane protease | S. hyicus | 4E-114 |
| 1422 | 1417913 | 1418248 | 112 | 17.9 | + | 744780571 | hypothetical protein SHYC_02855 | S. hyicus | 7E-44 |
| 1423 | 1418332 | 1419378 | 349 | 110.8 | + |  | No Significant Match |  |  |
| 1424 | 1419378 | 1420274 | 299 | 156.8 | + | Q88SC3 | N-acetylmuramic acid 6-phosphate etherase 1 | Lactobacillus plantarum | 8E-112 |
| 1425 | 1420288 | 1421697 | 470 | 226.1 | + | Q4L8H5 | PTS system EIIBC component SH0741 | S. haemolyticus | 0E+00 |
| 1426 | 1421791 | 1423182 | 464 | 217.3 | - | 744780570 | gamma-aminobutyrate permease family protein | S. hyicus | 0E+00 |
| 1427 | 1423512 | 1424342 | 277 | 118.4 | + | 744780569 | formate/nitrite transporter | S. hyicus | 0E+00 |
| 1428 | 1424396 | 1424734 | 113 | 32.8 | + | 744780568 | membrane protein | S. hyicus | 2E-41 |
| 1429 | 1424734 | 1424919 | 62 | 30.3 | + | 744780567 | membrane protein | S. hyicus | 7E-22 |
| 1430 | 1424972 | 1425163 | 64 | 21.3 | - | 744780566 | membrane protein | S. hyicus | 2E-34 |
| 1431 | 1425160 | 1425804 | 215 | 115.8 | - | 744780565 | HAD family hydrolase | S. hyicus | 2E-126 |
| 1432 | 1425870 | 1426697 | 276 | 124.2 | - | 746621747 | glyoxalase | S. hyicus | 2E-134 |
| 1433 | 1426760 | 1427029 | 90 | 6.8 | - | 744780563 | hypothetical protein SHYC_02815 | S. hyicus | 3E-39 |
| 1434 | 1427353 | 1428315 | 321 | 129.7 | - | 744780562 | sodium bile acid symporter family protein | S. hyicus | 0E+00 |
| 1435 | 1428496 | 1429026 | 177 | 77.3 | + | 744780561 | hypothetical protein SHYC_02805 | S. hyicus | 1E-100 |
| 1436 | 1429184 | 1429693 | 170 | 93.2 | - | 744780560 | hypothetical protein SHYC_02800 | S. hyicus | 5E-103 |
| 1437 | 1429952 | 1431250 | 433 | 216.1 | - | 744780559 | sodium:proton antiporter | S. hyicus | 0E+00 |
| 1438 | 1431492 | 1432619 | 376 | 191 | - | 744780558 | N-acetyl-L,L-diaminopimelate deacetylase | S. hyicus | 0E+00 |
| 1439 | 1432846 | 1434081 | 412 | 233.9 | - | 744780557 | imidazolonepropionase | S. hyicus | 0E+00 |
| 1440 | 1434081 | 1435748 | 556 | 365.9 | - | 744780556 | urocanate hydratase | S. hyicus | 0E+00 |
| 1441 | 1435768 | 1437096 | 443 | 133.6 | - | 744780259 | membrane protein | S. hyicus | 9E-48 |
| 1442 | 1437298 | 1438176 | 293 | 115.8 | + | 744780555 | LysR family transcriptional regulator | S. hyicus | 0E+00 |
| 1443 | 1438414 | 1439346 | 311 | 130 | - | 744780554 | formimidoylglutamase | S. hyicus | 0E+00 |
| 1444 | 1439660 | 1440694 | 345 | 146.9 | + | 744780553 | hypothetical protein SHYC_02765 | S. hyicus | 0E+00 |
| 1445 | 1440822 | 1441508 | 229 | 163.2 | - | 744780552 | ribose 5-phosphate isomerase | S. hyicus | 2E-151 |
| 1446 | 1441639 | 1442289 | 217 | 107.6 | + | 744780551 | hypothetical protein SHYC_02755 | S. hyicus | 4E-141 |
| 1447 | 1442392 | 1442592 | 67 | 33.6 | - | 744780550 | XRE family transcriptional regulator | S. hyicus | 1E-36 |
| 1448 | 1442624 | 1443631 | 336 | 144.4 | - | 744780549 | aldose 1-epimerase | S. hyicus | 0E+00 |
| 1449 | 1443664 | 1443996 | 111 | 48.4 | - | 744780548 | membrane protein | S. hyicus | 1E-57 |
| 1450 | 1444165 | 1445397 | 411 | 172.7 | - | 744780547 | sodium ABC transporter permease | S. hyicus | 0E+00 |
| 1451 | 1445390 | 1446289 | 300 | 148.3 | - | 744780546 | ABC transporter ATP-binding protein | S. hyicus | 0E+00 |
| 1452 | 1446463 | 1447110 | 216 | 60.3 | - | 744780545 | membrane protein | S. hyicus | 6E-64 |
| 1453 | 1447311 | 1447919 | 203 | 115.2 | + | 744780544 | 3-methyladenine DNA glycosylase | S. hyicus | 5E-138 |
| 1454 | 1447959 | 1449281 | 441 | 244.3 | - | 744780543 | sodium:proton antiporter | S. hyicus | 0E+00 |
| 1455 | 1449417 | 1450262 | 282 | 124.7 | - | 744780542 | hypothetical protein SHYC_02710 | S. hyicus | 9E-147 |
| 1456 | 1450392 | 1451216 | 275 | 85.2 | + | 744780541 | AraC family transcriptional regulator | S. hyicus | 1E-180 |
| 1457 | 1451369 | 1452580 | 404 | 183 | + | 744780540 | sodium:glutamate symporter | S. hyicus | 0E+00 |
| 1458 | 1452610 | 1453812 | 401 | 249.4 | - | 744780539 | cobalamin biosynthesis protein CobW | S. hyicus | 0E+00 |
| 1459 | 1454136 | 1455170 | 345 | 150.6 | - | 744780538 | isopentenyl pyrophosphate isomerase | S. hyicus | 0E+00 |
| 1460 | 1455294 | 1455773 | 160 | 93.2 | + | 744780537 | 3-hydroxyacyl-CoA dehydrogenase | S. hyicus | 4E-101 |
| 1461 | 1456115 | 1457146 | 344 | 188.4 | - | 744780536 | transcriptional regulator | S. hyicus | 0E+00 |
| 1462 | 1457385 | 1458290 | 302 | 117.3 | - | 744780535 | esterase | S. hyicus | 0E+00 |
| 1463 | 1458620 | 1459240 | 207 | 56.1 | + | 744780534 | membrane protein | S. hyicus | 4E-103 |
| 1464 | 1459331 | 1459888 | 186 | 50.7 | - | 744780533 | TetR family transcriptional regulator | S. hyicus | 9E-122 |
| 1465 | 1460297 | 1461691 | 465 | 212.2 | - | 744780532 | membrane protein | S. hyicus | 0E+00 |
| 1466 | 1461736 | 1462251 | 172 | 74.6 | - | 746618497 | MarR family transcriptional regulator | S. hyicus | 4E-103 |
| 1467 | 1462456 | 1463400 | 315 | 72.8 | + | 744780530 | YdcF family membrane protein | S. hyicus | 3E-143 |
| 1468 | 1463477 | 1465015 | 513 | 230.8 | + | P46133 | p-aminobenzoyl-glutamate transport protein | Escherichia coli | 3E-109 |
| 1469 | 1465169 | 1465840 | 224 | 134.8 | - | 744780529 | ABC transporter ATP-binding protein | S. hyicus | 4E-155 |
| 1470 | 1465840 | 1466901 | 354 | 192.6 | - | 744780528 | ABC transporter permease | S. hyicus | 0E+00 |
| 1471 | 1467126 | 1467803 | 226 | 132.1 | + | 744780527 | heme response regulator HssR | S. hyicus | 4E-160 |
| 1472 | 1467796 | 1469169 | 458 | 187.5 | + | 744780526 | signal transduction histidine kinase HssS | S. hyicus | 0E+00 |
| 1473 | 1469708 | 1471210 | 501 | 259.9 | - | 744780525 | malate:quinone oxidoreductase | S. hyicus | 0E+00 |
| 1474 | 1471484 | 1472647 | 388 | 184.4 | + | 744780524 | glycosyltransferase | S. hyicus | 0E+00 |
| 1475 | 1472761 | 1474362 | 534 | 289.9 | - | 744780523 | L-lactate permease | S. hyicus | 0E+00 |
| 1476 | 1474714 | 1475331 | 206 | 105.8 | - | 744780522 | lipoprotein | S. hyicus | 3E-97 |
| 1477 | 1475411 | 1475926 | 172 | 79.2 | - | 744780521 | hypothetical protein SHYC_02605 | S. hyicus | 2E-89 |
| 1478 | 1476050 | 1477036 | 329 | 180.6 | + | 744780520 | putative quinone oxidoreductase | S. hyicus | 0E+00 |
| 1479 | 1477210 | 1478544 | 445 | 247.1 | + | 744780519 | major facilitator transporter | S. hyicus | 0E+00 |
| 1480 | 1478679 | 1479707 | 343 | 162.3 | + | 744780518 | ferredoxin--NADP reductase | S. hyicus | 9E-150 |
| 1481 | 1479938 | 1480381 | 148 | 63.6 | - | 744780516 | membrane protein | S. hyicus | 4E-90 |
| 1482 | 1480448 | 1481074 | 209 | 103.5 | - | 744780515 | hypothetical protein SHYC_02575 | S. hyicus | 2E-96 |
| 1483 | 1481087 | 1482328 | 414 | 97.6 | - | 744780514 | copper resistance CopC family protein | S. hyicus | 2E-151 |
| 1484 | 1482523 | 1484190 | 556 | 329.9 | - | 744780513 | Na/Pi cotransporter | S. hyicus | 0E+00 |
| 1485 | 1484471 | 1484950 | 160 | 63.9 | + | 744780512 | MarR family transcriptional regulator | S. hyicus | 2E-109 |
| 1486 | 1484931 | 1485869 | 313 | 114.7 | + | 744780511 | magnesium transporter CorA | S. hyicus | 0E+00 |
| 1487 | 1486376 | 1488502 | 709 | 233.2 | + | 744780510 | AraC family transcriptional regulator | S. hyicus | 0E+00 |
| 1488 | 1488590 | 1489477 | 296 | 196.3 | + | 744780509 | UTP--glucose-1-phosphate uridylyltransferase | S. hyicus | 0E+00 |
| 1489 | 1489518 | 1490723 | 402 | 130.2 | - | 744780508 | permease | S. hyicus | 0E+00 |
| 1490 | 1490926 | 1491144 | 73 | 12.1 | - | 744780507 | hypothetical protein SHYC_02535 | S. hyicus | 3E-40 |
| 1491 | 1491340 | 1491684 | 115 | 77.4 | + | 744780506 | hypothetical protein SHYC_02530 | S. hyicus | 1E-72 |
| 1492 | 1492073 | 1492972 | 300 | 118.7 | - | 744780505 | transporter | S. hyicus | 0E+00 |
| 1493 | 1493136 | 1494209 | 358 | 204.8 | - | 744780504 | M42 family peptidase | S. hyicus | 0E+00 |
| 1494 | 1494380 | 1494736 | 119 | 52.6 | - | 744780503 | hypothetical protein SHYC_02515 | S. hyicus | 2E-77 |
| 1495 | 1494936 | 1496537 | 534 | 265.4 | + | 744780502 | arginine utilization protein RocB | S. hyicus | 0E+00 |
| 1496 | 1496686 | 1497846 | 387 | 172.1 | - | 744780501 | nitrate transporter NarT | S. hyicus | 0E+00 |
| 1497 | 1498112 | 1498786 | 225 | 106.4 | - | 744780500 | LuxR family transcriptional regulator | S. hyicus | 3E-154 |
| 1498 | 1498740 | 1499822 | 361 | 160.6 | - | 744780499 | two-component sensor histidine kinase NreB | S. hyicus | 0E+00 |
| 1499 | 1499822 | 1500289 | 156 | 40.8 | - | 744780498 | nitrogen regulation-related NreA protein | S. hyicus | 1E-105 |
| 1500 | 1500304 | 1500981 | 226 | 67.7 | - | 744780497 | nitrate reductase subunit gamma | S. hyicus | 2E-160 |
| 1501 | 1500974 | 1501549 | 192 | 87.5 | - | 744780496 | nitrate reductase subunit delta | S. hyicus | 2E-139 |
| 1502 | 1501542 | 1503098 | 519 | 256.6 | - | 744780495 | nitrate reductase subunit beta | S. hyicus | 0E+00 |
| 1503 | 1503088 | 1506759 | 1224 | 637.7 | - | 744780494 | respiratory nitrate reductase subunit alpha | S. hyicus | 0E+00 |
| 1504 | 1507054 | 1509348 | 765 | 383.9 | - | 744780493 | 5'-nucleotidase | S. hyicus | 0E+00 |
| 1505 | 1509632 | 1510570 | 313 | 132.9 | - | 744780492 | uroporphyrin-III methyltransferase | S. hyicus | 0E+00 |
| 1506 | 1510561 | 1510878 | 106 | 54.8 | - | 746618424 | nitrite reductase | S. hyicus | 3E-66 |
| 1507 | 1510881 | 1513286 | 802 | 498.6 | - | 744780490 | nitrite reductase | S. hyicus | 0E+00 |
| 1508 | 1513279 | 1513740 | 154 | 69.7 | - | 744780489 | siroheme synthase | S. hyicus | 2E-97 |
| 1509 | 1513709 | 1514461 | 251 | 57.9 | - | 744780488 | putative sirohydrochlorin ferrochelatase | S. hyicus | 3E-142 |
| 1510 | 1514584 | 1515132 | 183 | 89.6 | - | 744780487 | acetyltransferase | S. hyicus | 3E-119 |
| 1511 | 1515235 | 1516053 | 273 | 122.9 | - | 744780486 | formate/nitrite transporter | S. hyicus | 5E-166 |
| 1512 | 1516305 | 1516487 | 61 | 26.7 | - | 744780485 | membrane protein | S. hyicus | 9E-32 |
| 1513 | 1516810 | 1518336 | 509 | 251.6 | - | 744780484 | transporter | S. hyicus | 0E+00 |
| 1514 | 1518506 | 1519522 | 339 | 153.9 | - | 744780483 | PTS sugar transporter subunit IIC | S. hyicus | 0E+00 |
| 1515 | 1520286 | 1521773 | 496 | 280.7 | + | 744780482 | aldehyde dehydrogenase | S. hyicus | 0E+00 |
| 1516 | 1522366 | 1523397 | 344 | 181.8 | + | 744780481 | transcriptional regulator | S. hyicus | 0E+00 |
| 1517 | 1523412 | 1524089 | 226 | 129.8 | + | 744780480 | L-serine dehydratase subunit beta | S. hyicus | 4E-159 |
| 1518 | 1524105 | 1525004 | 300 | 195.4 | + | 744780479 | serine dehydratase subunit alpha | S. hyicus | 0E+00 |
| 1519 | 1525048 | 1525305 | 86 | 19.5 | - | 744780478 | hypothetical protein SHYC_02385 | S. hyicus | 2E-31 |
| 1520 | 1525562 | 1526698 | 379 | 167.1 | - | 744780477 | hypothetical protein SHYC_02380 | S. hyicus | 0E+00 |
| 1521 | 1526917 | 1527810 | 298 | 153 | - | 744780476 | peptide ABC transporter permease | S. hyicus | 0E+00 |
| 1522 | 1527821 | 1528783 | 321 | 127.3 | - | 744780475 | peptide ABC transporter permease | S. hyicus | 0E+00 |
| 1523 | 1528780 | 1529769 | 330 | 174.5 | - | 744780474 | peptide ABC transporter ATP-binding protein | S. hyicus | 0E+00 |
| 1524 | 1529762 | 1530772 | 337 | 165.1 | - | 744780473 | peptide ABC transporter ATP-binding protein | S. hyicus | 0E+00 |
| 1525 | 1530898 | 1532619 | 574 | 369.5 | - | 744780472 | ABC transporter substrate-binding lipoprotein | S. hyicus | 0E+00 |
| 1526 | 1532951 | 1533616 | 222 | 130.6 | - | 744780471 | oxidoreductase | S. hyicus | 5E-141 |
| 1527 | 1533986 | 1536235 | 750 | 431.4 | + | 744780470 | formate acetyltransferase | S. hyicus | 0E+00 |
| 1528 | 1536277 | 1537041 | 255 | 79.5 | + | 744780469 | pyruvate formate lyase-activating protein | S. hyicus | 1E-175 |
| 1529 | 1537614 | 1538666 | 351 | 173 | - | 744780468 | putative regulator | S. hyicus | 0E+00 |
| 1530 | 1538910 | 1540001 | 364 | 212.2 | - | 744780467 | leucine dehydrogenase | S. hyicus | 0E+00 |
| 1531 | 1540329 | 1541006 | 226 | 129.7 | + | 744780466 | iron-sulfur cluster repair di-iron protein | S. hyicus | 7E-152 |
| 1532 | 1541142 | 1542281 | 380 | 197.9 | - | 744780465 | glycerate kinase | S. hyicus | 0E+00 |
| 1533 | 1542349 | 1542951 | 201 | 109 | - | 744780464 | sortase A | S. hyicus | 6E-137 |
| 1534 | 1543501 | 1544784 | 428 | 181.1 | - | 744780463 | major facilitator superfamily protein | S. hyicus | 0E+00 |
| 1535 | 1545018 | 1546199 | 394 | 148.2 | - | 744780462 | major facilitator superfamily protein | S. hyicus | 0E+00 |
| 1536 | 1546458 | 1548416 | 653 | 323.5 | + | 744780461 | fructose 1,6-bisphosphatase | S. hyicus | 0E+00 |
| 1537 | 1549053 | 1550441 | 463 | 230.4 | + | 744780460 | amino acid transporter | S. hyicus | 0E+00 |
| 1538 | 1550531 | 1550959 | 143 | 90.2 | + | 744780459 | general stress protein | S. hyicus | 2E-80 |
| 1539 | 1551004 | 1551375 | 124 | 76.1 | + | 744780458 | glyoxalase | S. hyicus | 1E-76 |
| 1540 | 1551534 | 1553309 | 592 | 263.4 | - | 744780457 | ABC transporter ATP-binding protein | S. hyicus | 0E+00 |
| 1541 | 1553302 | 1555083 | 594 | 277.3 | - | 744780456 | ABC transporter ATP-binding protein | S. hyicus | 0E+00 |
| 1542 | 1555183 | 1556454 | 424 | 140.5 | - | 744780455 | Fe-S oxidoreductase | S. hyicus | 0E+00 |
| 1543 | 1556447 | 1557856 | 470 | 247.7 | - | 744780454 | FAD-binding protein | S. hyicus | 0E+00 |
| 1544 | 1558073 | 1560352 | 760 | 323.8 | - | 744780453 | nitric oxide reductase large subunit | S. hyicus | 0E+00 |
| 1545 | 1560820 | 1561248 | 143 | 53.6 | + | 744780452 | 6-pyruvoyl tetrahydrobiopterin synthase | S. hyicus | 3E-93 |
| 1546 | 1561355 | 1562068 | 238 | 111.8 | - | 744780451 | HAD superfamily hydrolase | S. hyicus | 2E-157 |
| 1547 | 1562186 | 1562926 | 247 | 127.5 | - | 744780450 | tryptophan synthase subunit alpha | S. hyicus | 2E-139 |
| 1548 | 1562919 | 1564133 | 405 | 262.4 | - | 744780449 | tryptophan synthase subunit beta | S. hyicus | 0E+00 |
| 1549 | 1564130 | 1564759 | 210 | 76.1 | - | 744780448 | N-(5'-phosphoribosyl)anthranilate isomerase | S. hyicus | 1E-91 |
| 1550 | 1564750 | 1565541 | 264 | 100.8 | - | 744780447 | indole-3-glycerol phosphate synthase | S. hyicus | 3E-144 |
| 1551 | 1565525 | 1566541 | 339 | 145.7 | - | 744780446 | anthranilate phosphoribosyltransferase | S. hyicus | 0E+00 |
| 1552 | 1566543 | 1567109 | 189 | 86.5 | - | 744780445 | anthranilate synthase subunit II | S. hyicus | 6E-102 |
| 1553 | 1567106 | 1568509 | 468 | 199.4 | - | 744780444 | anthranilate synthase component I | S. hyicus | 0E+00 |
| 1554 | 1568991 | 1570628 | 546 | 320.7 | - | 744780443 | putative pyruvate decarboxylase | S. hyicus | 0E+00 |
| 1555 | 1571385 | 1572512 | 376 | 145.7 | + | 744780442 | sugar diacid utilization regulator | S. hyicus | 0E+00 |
| 1556 | 1572651 | 1573058 | 136 | 75.4 | + | 744780441 | hypothetical protein SHYC_02200 | S. hyicus | 7E-79 |
| 1557 | 1573091 | 1573474 | 128 | 65.4 | - | 744780440 | MutT/NUDIX family protein | S. hyicus | 5E-66 |
| 1558 | 1573541 | 1574689 | 383 | 173.9 | - | 744780439 | pyridine nucleotide-disulfide oxidoreductase | S. hyicus | 0E+00 |
| 1559 | 1574813 | 1575667 | 285 | 141.5 | - | Q56623 | UDP-glucose 4-epimerase | Vibrio cholerae | 2E-22 |
| 1560 | 1575667 | 1576230 | 188 | 91.3 | - | P71062 | Uncharacterized sugar transferase EpsL | Bacillus subtilis | 7E-47 |
| 1561 | 1576245 | 1577450 | 402 | 163.9 | - | Q8KIU8 | Probable glycosyltransferase WbjE | Pseudomonas aeruginosa | 1E-20 |
| 1562 | 1577460 | 1578698 | 413 | 70 | - |  | No Significant Match |  |  |
| 1563 | 1578685 | 1579257 | 191 | 48.9 | - | Q552S7 | Putative acetyltransferase DDB_G0275913 | Dictyostelium discoideum | 5E-21 |
| 1564 | 1579232 | 1580647 | 472 | 103 | - |  | No Significant Match |  |  |
| 1565 | 1580634 | 1581725 | 364 | 86.1 | - |  | No Significant Match |  |  |
| 1566 | 1581722 | 1582849 | 376 | 182.6 | - | 744780431 | UDP-N-acetylglucosamine 2-epimerase | S. hyicus | 0E+00 |
| 1567 | 1582852 | 1583961 | 370 | 154.4 | - | 744780430 | capsular biosynthesis protein | S. hyicus | 0E+00 |
| 1568 | 1583965 | 1584999 | 345 | 187.2 | - | 744780429 | UDP-glucose 4-epimerase | S. hyicus | 0E+00 |
| 1569 | 1585010 | 1586818 | 603 | 269.3 | - | 744780428 | polysaccharide biosynthesis protein | S. hyicus | 0E+00 |
| 1570 | 1586847 | 1587617 | 257 | 127.4 | - | 744780427 | capsular polysaccharide biosynthesis protein | S. hyicus | 1E-176 |
| 1571 | 1587614 | 1588321 | 236 | 107.5 | - | 744780426 | capsular polysaccharide biosynthesis protein | S. hyicus | 2E-143 |
| 1572 | 1588338 | 1589000 | 221 | 122.9 | - | 744780425 | capsular polysaccharide biosynthesis protein | S. hyicus | 2E-88 |
| 1573 | 1589461 | 1590918 | 486 | 115.2 | - | 744780423 | hypothetical protein SHYC_02110 | S. hyicus | 0E+00 |
| 1574 | 1591301 | 1592182 | 294 | 193.2 | - | 744780422 | oxidoreductase | S. hyicus | 0E+00 |
| 1575 | 1592314 | 1592514 | 67 | 33.6 | + | 744780421 | hypothetical protein SHYC_02100 | S. hyicus | 5E-38 |
| 1576 | 1592724 | 1592951 | 76 | 30.2 | + | 744780420 | hypothetical protein SHYC_02095 | S. hyicus | 9E-27 |
| 1577 | 1593075 | 1594220 | 382 | 206.9 | - | 744780419 | Sodium:dicarboxylate symporter family protein | S. hyicus | 0E+00 |
| 1578 | 1594447 | 1595571 | 375 | 181.7 | - | 744780418 | hypothetical protein SHYC_02085 | S. hyicus | 0E+00 |
| 1579 | 1595793 | 1597784 | 664 | 360.1 | + | 744780417 | catalase | S. hyicus | 0E+00 |
| 1580 | 1597812 | 1598678 | 289 | 177.3 | + | 744780416 | short-chain dehydrogenase | S. hyicus | 0E+00 |
| 1581 | 1599092 | 1599340 | 83 | 18.1 | - |  | No Significant Match |  |  |
| 1582 | 1599344 | 1600918 | 525 | 259.2 | - | 744780415 | hypothetical protein SHYC_02070 | S. hyicus | 6E-65 |
| 1583 | 1601166 | 1602380 | 405 | 153.8 | - | 744780414 | major facilitator superfamily protein | S. hyicus | 0E+00 |
| 1584 | 1602441 | 1603058 | 206 | 110.5 | - | 744780413 | kynurenine formamidase | S. hyicus | 9E-119 |
| 1585 | 1603148 | 1604050 | 301 | 144.8 | - | 744780412 | esterase | S. hyicus | 0E+00 |
| 1586 | 1604165 | 1605583 | 473 | 225 | - | 744780411 | phosphate:AMP phosphotransferase | S. hyicus | 0E+00 |
| 1587 | 1606001 | 1606675 | 225 | 128.9 | + | 744780410 | putative transglycosylase | S. hyicus | 1E-151 |
| 1588 | 1607460 | 1608314 | 285 | 107 | + | 744780409 | secretory antigen precursor SsaA | S. hyicus | 1E-153 |
| 1589 | 1608475 | 1609035 | 187 | 72.7 | + | 744780408 | M23 family peptidase | S. hyicus | 5E-106 |
| 1590 | 1609083 | 1609796 | 238 | 93 | - | 744780407 | GTP pyrophosphokinase | S. hyicus | 1E-174 |
| 1591 | 1609901 | 1610521 | 207 | 116 | - | 744780406 | NAD(P)H nitroreductase | S. hyicus | 3E-147 |
| 1592 | 1610654 | 1611094 | 147 | 67.5 | + | 744780405 | transcriptional regulator | S. hyicus | 4E-99 |
| 1593 | 1611392 | 1612420 | 343 | 238.6 | - | 744780404 | iron ABC transporter substrate-binding lipoprotein | S. hyicus | 0E+00 |
| 1594 | 1612435 | 1613238 | 268 | 136 | - | 744780403 | iron ABC transporter ATP-binding protein | S. hyicus | 0E+00 |
| 1595 | 1613235 | 1614194 | 320 | 113 | - | 744780402 | iron ABC transporter permease | S. hyicus | 0E+00 |
| 1596 | 1614187 | 1615152 | 322 | 122 | - | 744780401 | iron ABC transporter permease | S. hyicus | 0E+00 |
| 1597 | 1615313 | 1615840 | 176 | 45.7 | - | 744780400 | hypothetical protein SHYC_01990 | S. hyicus | 3E-116 |
| 1598 | 1616003 | 1616182 | 60 | 22.2 | - | 744780399 | hypothetical protein SHYC_01985 | S. hyicus | 2E-24 |
| 1599 | 1616352 | 1617539 | 396 | 144.5 | + | 744780398 | membrane protein | S. hyicus | 0E+00 |
| 1600 | 1617584 | 1617937 | 118 | 46.3 | + | 744780397 | hypothetical protein SHYC_01975 | S. hyicus | 2E-58 |
| 1601 | 1618061 | 1619368 | 436 | 126.4 | - | 744780396 | branched-chain amino acid transporter II carrier protein | S. hyicus | 0E+00 |
| 1602 | 1619653 | 1620075 | 141 | 59.9 | - | 744780395 | universal stress protein | S. hyicus | 1E-83 |
| 1603 | 1620347 | 1621246 | 300 | 146.8 | - | 744780394 | CobW/HypB/UreG family protein | S. hyicus | 0E+00 |
| 1604 | 1621246 | 1621791 | 182 | 90.9 | - | 744780393 | deoxycytidine triphosphate deaminase | S. hyicus | 6E-128 |
| 1605 | 1622077 | 1622619 | 181 | 95.9 | - | 744780392 | lipoprotein | S. hyicus | 5E-86 |
| 1606 | 1622633 | 1623193 | 187 | 100.4 | - | 744780391 | hypothetical protein SHYC_01945 | S. hyicus | 8E-95 |
| 1607 | 1623404 | 1625119 | 572 | 344.7 | - | 744780390 | urease subunit alpha | S. hyicus | 0E+00 |
| 1608 | 1625116 | 1625472 | 119 | 63.5 | - | 744780389 | urease subunit beta | S. hyicus | 1E-76 |
| 1609 | 1625483 | 1625785 | 101 | 55.3 | - | 744780388 | urease subunit gamma | S. hyicus | 4E-67 |
| 1610 | 1626005 | 1626919 | 305 | 85.8 | + | 744780387 | urea transporter | S. hyicus | 1E-167 |
| 1611 | 1627093 | 1628028 | 312 | 131.2 | - | 744780386 | cation transporter | S. hyicus | 0E+00 |
| 1612 | 1628042 | 1628389 | 116 | 40 | - | 744780384 | histidine kinase | S. hyicus | 1E-53 |
| 1613 | 1628534 | 1628938 | 135 | 59.8 | - | 744780383 | two component transcriptional regulator | S. hyicus | 2E-70 |
| 1614 | 1629082 | 1629216 | 45 | 19.7 | - | 744780382 | hypothetical protein SHYC_01900 | S. hyicus | 1E-14 |
| 1615 | 1629430 | 1630602 | 391 | 155 | - | 744780381 | 0utative penicillin-binding protein | S. hyicus | 0E+00 |
| 1616 | 1630652 | 1631629 | 326 | 136.4 | - | 744780380 | ABC transporter permease | S. hyicus | 0E+00 |
| 1617 | 1631944 | 1632429 | 162 | 33.5 | - | 744780376 | hypothetical protein SHYC_01870 | S. hyicus | 5E-62 |
| 1618 | 1632446 | 1633315 | 290 | 158.5 | - | 744780375 | fructosamine kinase | S. hyicus | 1E-179 |
| 1619 | 1633468 | 1634133 | 222 | 106.6 | + | 744780374 | NAD(P)H nitroreductase | S. hyicus | 5E-145 |
| 1620 | 1634229 | 1636205 | 659 | 337.7 | - | 744780373 | lipase | S. hyicus | 0E+00 |
| 1621 | 1636729 | 1638198 | 490 | 209.2 | + | 744780372 | PTS transporter subunit IIBC | S. hyicus | 0E+00 |
| 1622 | 1638346 | 1639830 | 495 | 135.7 | - | 744780371 | membrane protein | S. hyicus | 0E+00 |
| 1623 | 1639925 | 1641337 | 471 | 172.4 | - | 744780370 | glycosyltransferase | S. hyicus | 0E+00 |
| 1624 | 1641443 | 1644643 | 1067 | 432.2 | + | 744780369 | CDP-glycerol:glycerophosphate glycerophosphotransferase | S. hyicus | 0E+00 |
| 1625 | 1644822 | 1646990 | 723 | 295.5 | + | 744780368 | CDP-glycerol:glycerophosphate glycerophosphotransferase | S. hyicus | 0E+00 |
| 1626 | 1647053 | 1647757 | 235 | 67.6 | - | Q8NWT6 | Putative oligopeptide transport ATP-binding protein oppF2 | S. aureus | 4E-52 |
| 1627 | 1647754 | 1648530 | 259 | 108.2 | - | Q7A5Q8 | Putative oligopeptide transport ATP-binding protein oppD2 | S. aureus | 1E-66 |
| 1628 | 1648532 | 1649335 | 268 | 107.1 | - | Q6GH26 | Putative oligopeptide transport system permease protein oppC2 | S. aureus | 3E-103 |
| 1629 | 1649335 | 1650318 | 328 | 105.9 | - | Q7A5Q6 | Putative oligopeptide transport system permease protein oppB2 | S. aureus | 5E-87 |
| 1630 | 1650308 | 1651795 | 496 | 222.7 | - | Q8X6V9 | Glutathione-binding protein GsiB | Escherichia coli | 4E-21 |
| 1631 | 1651933 | 1652655 | 241 | 120.1 | - | 744780367 | hypothetical protein SHYC_01825 | S. hyicus | 3E-177 |
| 1632 | 1652903 | 1653121 | 73 | 11.7 | + | 744780366 | hypothetical protein SHYC_01820 | S. hyicus | 4E-45 |
| 1633 | 1653352 | 1653810 | 153 | 81.6 | + | 744780365 | hypothetical protein SHYC_01815 | S. hyicus | 3E-53 |
| 1634 | 1653885 | 1655633 | 583 | 242.5 | - | 744780364 | YhgE/Pip domain-containing protein | S. hyicus | 0E+00 |
| 1635 | 1655830 | 1657146 | 439 | 214.2 | + | 744780363 | N-acetylmuramoyl-L-alanine amidase domain-containing protein | S. hyicus | 0E+00 |
| 1636 | 1657248 | 1657829 | 194 | 85.6 | - | 744780362 | hypothetical protein SHYC_01800 | S. hyicus | 1E-113 |
| 1637 | 1657955 | 1659151 | 399 | 127.2 | - | 744780361 | major facilitator family transporter | S. hyicus | 0E+00 |
| 1638 | 1659331 | 1661277 | 649 | 351.1 | - | 744780360 | PTS transporter subunit IIABC | S. hyicus | 0E+00 |
| 1639 | 1661496 | 1662221 | 242 | 108.8 | - | 744780359 | transcriptional regulator | S. hyicus | 4E-158 |
| 1640 | 1662253 | 1663206 | 318 | 119.5 | - | 744780358 | alpha/beta hydrolase | S. hyicus | 0E+00 |
| 1641 | 1663653 | 1663955 | 101 | 8.4 | - | 744780356 | hypothetical protein SHYC_01770 | S. hyicus | 9E-60 |
| 1642 | 1663981 | 1665360 | 460 | 253.1 | - | 744780355 | succinate-semialdehyde dehydrogenase | S. hyicus | 0E+00 |
| 1643 | 1665532 | 1665882 | 117 | 71.7 | + | 744780354 | hypothetical protein SHYC_01760 | S. hyicus | 8E-81 |
| 1644 | 1666036 | 1667277 | 414 | 181 | - | 744780353 | aminoacyltransferase FemB | S. hyicus | 0E+00 |
| 1645 | 1667581 | 1667946 | 122 | 80.5 | - | 744780352 | dihydroxyacetone kinase, phosphotransfer subunit | S. hyicus | 2E-78 |
| 1646 | 1667948 | 1668523 | 192 | 134.4 | - | 744780351 | dihydroxyacetone kinase subunit L | S. hyicus | 5E-129 |
| 1647 | 1668537 | 1669505 | 323 | 206.5 | - | 744780350 | dihydroxyacetone kinase | S. hyicus | 0E+00 |
| 1648 | 1670001 | 1671362 | 454 | 208.2 | + | 744780349 | glycerol-3-phosphate ABC transporter | S. hyicus | 0E+00 |
| 1649 | 1671470 | 1672645 | 392 | 242.7 | - | 744780348 | phosphopentomutase | S. hyicus | 0E+00 |
| 1650 | 1672675 | 1673337 | 221 | 161.5 | - | 744780164 | deoxyribose-phosphate aldolase | S. hyicus | 1E-152 |
| 1651 | 1673572 | 1674408 | 279 | 118.9 | + | 744780346 | ATP phosphoribosyltransferase regulatory subunit | S. hyicus | 3E-167 |
| 1652 | 1674396 | 1675010 | 205 | 94.7 | + | 744780345 | ATP phosphoribosyltransferase | S. hyicus | 4E-130 |
| 1653 | 1675000 | 1676250 | 417 | 185.3 | + | 744780344 | histidinol dehydrogenase | S. hyicus | 0E+00 |
| 1654 | 1676256 | 1677272 | 339 | 124.1 | + | 744780343 | histidinol-phosphate aminotransferase | S. hyicus | 0E+00 |
| 1655 | 1677250 | 1677825 | 192 | 88.3 | + | 744780342 | imidazoleglycerol-phosphate dehydratase | S. hyicus | 3E-136 |
| 1656 | 1677822 | 1678415 | 198 | 107.3 | + | 744780341 | imidazole glycerol phosphate synthase | S. hyicus | 3E-117 |
| 1657 | 1678390 | 1679094 | 235 | 106.3 | + | 746618142 | 1-(5-phosphoribosyl)-5-[(5-phosphoribosylamino)methylideneamino] imidazole-4-carboxamide isomerase | S. hyicus | 2E-147 |
| 1658 | 1679091 | 1679855 | 255 | 128.2 | + | 744780339 | imidazole glycerol phosphate synthase | S. hyicus | 4E-166 |
| 1659 | 1679846 | 1680475 | 210 | 101.8 | + | 744780338 | phosphoribosyl-AMP cyclohydrolase/phosphoribosyl-ATP pyrophosphatase | S. hyicus | 2E-141 |
| 1660 | 1680542 | 1681300 | 253 | 52.8 | - | Q8CU21 | Uncharacterized lipoprotein SE_0142 | S. epidermidis | 9E-27 |
| 1661 | 1681257 | 1682222 | 322 | 127.1 | - | 744780337 | ornithine carbamoyltransferase | S. hyicus | 0E+00 |
| 1662 | 1682484 | 1683101 | 206 | 61 | - | 744780327 | hypothetical protein SHYC_01615 | S. hyicus | 2E-23 |
| 1663 | 1683257 | 1684006 | 250 | 54.2 | + |  | No Significant Match |  |  |
| 1664 | 1684026 | 1685720 | 565 | 347.9 | - | 744780336 | choline dehydrogenase | S. hyicus | 0E+00 |
| 1665 | 1685742 | 1687241 | 500 | 334.7 | - | 744780335 | betaine-aldehyde dehydrogenase | S. hyicus | 0E+00 |
| 1666 | 1687525 | 1688085 | 187 | 112.1 | + | 744780334 | transcriptional regulator | S. hyicus | 8E-121 |
| 1667 | 1688333 | 1689967 | 545 | 229.9 | - | 744780333 | choline-glycine betaine transporter family protein | S. hyicus | 0E+00 |
| 1668 | 1690895 | 1691371 | 159 | 86.6 | - | 746618124 | hypothetical protein | S. hyicus | 7E-48 |
| 1669 | 1691395 | 1691493 | 33 | 3.5 | - |  | No Significant Match |  |  |
| 1670 | 1691628 | 1692335 | 236 | 115.2 | + | 744780331 | alpha-acetolactate decarboxylase | S. hyicus | 9E-167 |
| 1671 | 1692418 | 1693857 | 480 | 294.4 | - | 744780329 | alkaline phosphatase | S. hyicus | 0E+00 |
| 1672 | 1694132 | 1695571 | 480 | 298.1 | - | 744780328 | alkaline phosphatase | S. hyicus | 0E+00 |
| 1673 | 1695790 | 1696440 | 217 | 63.4 | + | 744780327 | hypothetical protein SHYC_01615 | S. hyicus | 5E-111 |
| 1674 | 1696513 | 1697127 | 205 | 72 | - | 744780326 | putative alkaline phosphatase | S. hyicus | 1E-140 |
| 1675 | 1697137 | 1697241 | 35 | 2 | - |  | No Significant Match |  |  |
| 1676 | 1697245 | 1697655 | 137 | 57.9 | - | 744780325 | hypothetical protein SHYC_01605 | S. hyicus | 2E-78 |
| 1677 | 1697917 | 1698189 | 91 | 33.5 | + | 744780324 | hypothetical protein SHYC_01600 | S. hyicus | 8E-40 |
| 1678 | 1698594 | 1699604 | 337 | 113.8 | - | 744780322 | high-affinity nickel transport protein | S. hyicus | 0E+00 |
| 1679 | 1699818 | 1700495 | 226 | 110.6 | + | 744780321 | two-component system response regulator | S. hyicus | 5E-165 |
| 1680 | 1700488 | 1701972 | 495 | 196.9 | + | 744780320 | two-component system sensor histidine kinase | S. hyicus | 0E+00 |
| 1681 | 1702193 | 1702633 | 147 | 64.1 | - | 744780319 | hypothetical protein SHYC_01575 | S. hyicus | 5E-78 |
| 1682 | 1702818 | 1704308 | 497 | 245.8 | - | 744780318 | phospholipase D/cardiolipin synthetase | S. hyicus | 0E+00 |
| 1683 | 1704746 | 1705705 | 320 | 151.3 | + | Q9RLV9 | Sphingomyelinase C | Listeria ivanovii | 2E-114 |
| 1684 | 1706415 | 1707575 | 387 | 179.6 | + | 744780317 | poly-gamma-glutamate synthase PgsB | S. hyicus | 0E+00 |
| 1685 | 1707578 | 1708030 | 151 | 53.1 | + | 744780316 | capsule biosynthesis protein CapC | S. hyicus | 3E-98 |
| 1686 | 1708045 | 1709115 | 357 | 166.1 | + | 744780315 | capsule biosynthesis protein CapA | S. hyicus | 0E+00 |
| 1687 | 1709105 | 1709398 | 98 | 39.8 | + | 744780314 | hypothetical protein SHYC_01540 | S. hyicus | 1E-57 |
| 1688 | 1709382 | 1710980 | 533 | 300.3 | + | 744780313 | gamma-glutamyltranspeptidase | S. hyicus | 0E+00 |
| 1689 | 1711027 | 1711275 | 83 | 25.7 | - | 744780312 | hypothetical protein SHYC_01530 | S. hyicus | 1E-16 |
| 1690 | 1711506 | 1714073 | 856 | 316.3 | - | 744780311 | membrane protein | S. hyicus | 0E+00 |
| 1691 | 1714364 | 1715071 | 236 | 96.1 | - | 744780310 | hypothetical protein SHYC_01520 | S. hyicus | 3E-173 |
| 1692 | 1715659 | 1715892 | 78 | 34.2 | + | 744780309 | hypothetical protein SHYC_01515 | S. hyicus | 2E-46 |
| 1693 | 1715950 | 1716192 | 81 | 20.5 | - | 744780308 | hypothetical protein SHYC_01510 | S. hyicus | 2E-07 |
| 1694 | 1716569 | 1717057 | 163 | 72.3 | + | 744780307 | membrane protein | S. hyicus | 3E-112 |
| 1695 | 1717110 | 1718375 | 422 | 283.6 | - | 744780306 | membrane protein | S. hyicus | 0E+00 |
| 1696 | 1718493 | 1719677 | 395 | 167.8 | - | 744780305 | MFS transporter permease | S. hyicus | 0E+00 |
| 1697 | 1719953 | 1720309 | 119 | 87.9 | + | 744780304 | hypothetical protein SHYC_01490 | S. hyicus | 2E-78 |
| 1698 | 1720504 | 1722102 | 533 | 261.9 | - | 744780303 | FGGY family carbohydrate kinase | S. hyicus | 0E+00 |
| 1699 | 1722703 | 1724061 | 453 | 228.1 | + | 744780302 | Na+/alanine symporter | S. hyicus | 0E+00 |
| 1700 | 1724333 | 1725727 | 465 | 209.7 | - | 744780301 | major facilitator superfamily permease | S. hyicus | 0E+00 |
| 1701 | 1725989 | 1727218 | 410 | 231 | - | 744780300 | ArgE/DapE family peptidase | S. hyicus | 0E+00 |
| 1702 | 1727244 | 1728668 | 475 | 202.2 | - | 744780299 | MFS transporter | S. hyicus | 0E+00 |
| 1703 | 1728871 | 1729806 | 312 | 206.1 | + | 744780298 | 2-dehydropantoate 2-reductase | S. hyicus | 0E+00 |
| 1704 | 1731258 | 1731533 | 92 | 20.7 | - | Q5WKY5 | 6-phospho-5-dehydro-2-deoxy-D-gluconate aldolase | Bacillus clausii | 9E-18 |
| 1705 | 1731693 | 1732097 | 135 | 63.2 | - | Q5WKY5 | 6-phospho-5-dehydro-2-deoxy-D-gluconate aldolase | Bacillus clausii | 7E-44 |
| 1706 | 1732117 | 1733112 | 332 | 175.5 | - | O05389 | Uncharacterized oxidoreductase YrbE | Bacillus subtilis | 2E-128 |
| 1707 | 1733114 | 1734718 | 535 | 217.4 | - | P31448 | Uncharacterized symporter YidK | Escherichia coli | 2E-138 |
| 1708 | 1734766 | 1735647 | 294 | 146.6 | - | B3W8L5 | Inosose dehydratase | Lactobacillus casei | 8E-146 |
| 1709 | 1735658 | 1736674 | 339 | 156.6 | - | P40332 | Uncharacterized oxidoreductase YisS | Bacillus subtilis | 9E-47 |
| 1710 | 1736693 | 1738603 | 637 | 326.2 | - | Q65D03 | 3D-(3,5/4)-trihydroxycyclohexane-1,2-dione hydrolase | Bacillus licheniformis | 0E+00 |
| 1711 | 1738609 | 1739577 | 323 | 163.4 | - | Q65D02 | 5-dehydro-2-deoxygluconokinase | Bacillus licheniformis | 7E-145 |
| 1712 | 1739590 | 1740405 | 272 | 134.3 | - | A7ZAI0 | 5-deoxy-glucuronate isomerase | Bacillus amyloliquefaciens | 5E-119 |
| 1713 | 1740417 | 1741871 | 485 | 281.1 | - | Q65D00 | methylmalonate-semialdehyde dehydrogenase | S. aureus | 0E+00 |
| 1714 | 1742070 | 1742828 | 253 | 111 | + | P46337 | HTH-type transcriptional regulator IolR | Bacillus subtilis | 2E-64 |
| 1715 | 1743197 | 1743769 | 191 | 67.3 | - | 744780297 | hypothetical protein SHYC_01445 | S. hyicus | 2E-112 |
| 1716 | 1744229 | 1744471 | 81 | 10.6 | - |  | No Significant Match |  |  |
| 1717 | 1744543 | 1744866 | 108 | 17.7 | - |  | No Significant Match |  |  |
| 1718 | 1745319 | 1747121 | 601 | 230.9 | + | 744780296 | O-acetyltransferase OatA | S. hyicus | 0E+00 |
| 1719 | 1747271 | 1747981 | 237 | 119.3 | + | 744780295 | hypothetical protein SHYC_01435 | S. hyicus | 1E-130 |
| 1720 | 1748059 | 1749438 | 460 | 203.1 | - | P0AGC2 | Hexose phosphate transport protein | Shigella flexneri | 2E-139 |
| 1721 | 1749772 | 1750335 | 188 | 90.4 | - | 744780294 | transcriptional regulator | S. hyicus | 7E-93 |
| 1722 | 1750319 | 1751128 | 270 | 127.2 | - | 744780293 | N-acetyltransferase | S. hyicus | 5E-147 |
| 1723 | 1751395 | 1751946 | 184 | 106.4 | + | 744780292 | isochorismatase family protein | S. hyicus | 5E-120 |
| 1724 | 1751957 | 1753150 | 398 | 104.7 | + | 744780291 | Bcr/CflA subfamily MFS protein | S. hyicus | 0E+00 |
| 1725 | 1753225 | 1753524 | 100 | 43.9 | - | 744780290 | hypothetical protein SHYC_01410 | S. hyicus | 2E-34 |
| 1726 | 1753529 | 1753855 | 109 | 50.3 | - | 744780289 | hypothetical protein SHYC_01405 | S. hyicus | 9E-63 |
| 1727 | 1753852 | 1755177 | 442 | 210.9 | - | 744780288 | membrane protein | S. hyicus | 0E+00 |
| 1728 | 1755170 | 1755925 | 252 | 77.1 | - | 744780285 | staphylococcus tandem lipoprotein family protein | S. hyicus | 2E-131 |
| 1729 | 1756123 | 1756575 | 151 | 60.9 | - | 746621707 | hypothetical protein, partial | S. hyicus | 2E-80 |
| 1730 | 1756568 | 1757314 | 249 | 61.6 | - | 744780285 | staphylococcus tandem lipoprotein family protein | S. hyicus | 3E-116 |
| 1731 | 1757549 | 1758442 | 298 | 200.4 | + | 744780279 | fructose-1,6-bisphosphate aldolase | S. hyicus | 0E+00 |
| 1732 | 1758481 | 1759539 | 353 | 235 | - | 744780278 | ABC transporter substrate-binding lipoprotein | S. hyicus | 0E+00 |
| 1733 | 1759739 | 1760755 | 339 | 147 | + | 744780277 | putative lipase LipA | S. hyicus | 0E+00 |
| 1734 | 1760775 | 1761962 | 396 | 123.2 | - | 744780276 | major facilitator superfamily protein | S. hyicus | 0E+00 |
| 1735 | 1762157 | 1762849 | 231 | 110.8 | - | 744780275 | ThiJ/PfpI family protein | S. hyicus | 2E-168 |
| 1736 | 1762969 | 1763133 | 55 | 16.7 | - | 744780274 | hypothetical protein SHYC_01335 | S. hyicus | 1E-27 |
| 1737 | 1763147 | 1764427 | 427 | 227.5 | - | 744780273 | O-acetylhomoserine aminocarboxypropyltransferase | S. hyicus | 0E+00 |
| 1738 | 1764828 | 1765940 | 371 | 157.5 | + | 744780272 | pseudouridine kinase | S. hyicus | 0E+00 |
| 1739 | 1765924 | 1766835 | 304 | 188.7 | + | 744780271 | pseudouridine-5'-phosphate glycosidase | S. hyicus | 0E+00 |
| 1740 | 1766845 | 1768041 | 399 | 168.4 | + | 744780270 | pyrimidine nucleoside transporter NupC | S. hyicus | 0E+00 |
| 1741 | 1768200 | 1769546 | 449 | 199.1 | - | 744780269 | PTS transporter subunit IIC | S. hyicus | 0E+00 |
| 1742 | 1769563 | 1769847 | 95 | 65.6 | - | 744780268 | PTS transporter subunit IIB | S. hyicus | 1E-56 |
| 1743 | 1769849 | 1770292 | 148 | 74.7 | - | 744780267 | PTS transporter subunit IIA | S. hyicus | 8E-88 |
| 1744 | 1770283 | 1772235 | 651 | 251.8 | - | 744780266 | PTS multidomain regulator | S. hyicus | 0E+00 |
| 1745 | 1772432 | 1773847 | 472 | 162.7 | + | 744780265 | putative ethanolamine sensory transduction histidine kinase | S. hyicus | 0E+00 |
| 1746 | 1773849 | 1774424 | 192 | 93.1 | + | 744780264 | response regulator protein | S. hyicus | 2E-127 |
| 1747 | 1774506 | 1775738 | 411 | 180 | + | 744780263 | ethanolamine utilization protein EutH | S. hyicus | 0E+00 |
| 1748 | 1776027 | 1776794 | 256 | 133.6 | - | 744780262 | short-chain dehydrogenase | S. hyicus | 0E+00 |
| 1749 | 1776823 | 1778223 | 467 | 226.6 | - | P0C7B6 | Type-1 glutamine synthetase 2 | Dictyostelium discoideum | 4E-73 |
| 1750 | 1778240 | 1779667 | 476 | 230.9 | - | 744780261 | succinate-semialdehyde dehydrogenase | S. hyicus | 0E+00 |
| 1751 | 1779854 | 1780921 | 356 | 133 | + | 744780260 | ArgE/DapE family protein | S. hyicus | 0E+00 |
| 1752 | 1781043 | 1782422 | 460 | 204.7 | - | 744780259 | membrane protein | S. hyicus | 0E+00 |
| 1753 | 1782850 | 1784853 | 668 | 312.3 | - | 744780258 | NhaP-type Na+/H+ and K+/H+ antiporter | S. hyicus | 0E+00 |
| 1754 | 1784905 | 1785369 | 155 | 72.7 | - | 744780256 | methionine sulfoxide reductase A | S. hyicus | 5E-107 |
| 1755 | 1786257 | 1787171 | 305 | 162.7 | - | 744780255 | ribose ABC transporter ribose-binding lipoprotein rbsB | S. hyicus | 0E+00 |
| 1756 | 1787191 | 1788129 | 313 | 163.9 | - | 744780254 | ribose ABC transporter permease | S. hyicus | 0E+00 |
| 1757 | 1788110 | 1789606 | 499 | 289.6 | - | 744780253 | D-ribose ABC transporter ATP-binding protein | S. hyicus | 0E+00 |
| 1758 | 1789619 | 1790008 | 130 | 50.4 | - | 744780252 | D-ribose pyranase | S. hyicus | 2E-77 |
| 1759 | 1790001 | 1790882 | 294 | 172.8 | - | 744780251 | ribokinase | S. hyicus | 0E+00 |
| 1760 | 1790879 | 1791850 | 324 | 147.2 | - | 744780250 | LacI family transcriptional regulator | S. hyicus | 0E+00 |
| 1761 | 1791987 | 1793330 | 448 | 239.1 | - | 744780249 | metallo-beta-lactamase superfamily protein | S. hyicus | 0E+00 |
| 1762 | 1793343 | 1794422 | 360 | 200.6 | - | 744780248 | DsrE/DsrF/DrsH-like family protein | S. hyicus | 0E+00 |
| 1763 | 1794560 | 1794820 | 87 | 55.6 | + | 744780247 | putative transcriptional regulator | S. hyicus | 7E-51 |
| 1764 | 1794823 | 1795575 | 251 | 103.5 | + | 744780246 | membrane protein | S. hyicus | 7E-170 |
| 1765 | 1795635 | 1796957 | 441 | 257.5 | - | 744780245 | pyridine nucleotide-disulfide oxidoreductase protein | S. hyicus | 0E+00 |
| 1766 | 1796954 | 1797382 | 143 | 47.1 | - | 744780244 | transcriptional regulator | S. hyicus | 3E-101 |
| 1767 | 1797879 | 1798337 | 153 | 56.9 | - | 744780243 | putative DNA topology modulation protein FlaR | S. hyicus | 4E-109 |
| 1768 | 1798348 | 1799013 | 222 | 113.6 | - | 744780242 | peptide ABC transporter ATP-binding protein | S. hyicus | 5E-147 |
| 1769 | 1799025 | 1800074 | 350 | 138.8 | - | 744780241 | peptide ABC transporter permease | S. hyicus | 0E+00 |
| 1770 | 1800277 | 1801851 | 525 | 226.2 | + | 744780240 | acyl-CoA transferase FadX | S. hyicus | 0E+00 |
| 1771 | 1801927 | 1803432 | 502 | 266.7 | + | 744780239 | long-chain fatty acid--CoA ligase | S. hyicus | 0E+00 |
| 1772 | 1803492 | 1804700 | 403 | 241.5 | + | 744780238 | glutaryl-CoA dehydrogenase | S. hyicus | 0E+00 |
| 1773 | 1804728 | 1806989 | 754 | 386.2 | + | 744780237 | 3-hydroxyacyl-CoA dehydrogenase | S. hyicus | 0E+00 |
| 1774 | 1807012 | 1808196 | 395 | 248.7 | + | 744780236 | acetyl-CoA acetyltransferase | S. hyicus | 0E+00 |
| 1775 | 1808255 | 1809145 | 297 | 139.5 | - | 744780235 | isomerase | S. hyicus | 1E-170 |
| 1776 | 1809413 | 1810753 | 447 | 202.9 | - | 744780234 | accessory Sec system glycosyltransferase GtfB | S. hyicus | 0E+00 |
| 1777 | 1810746 | 1812254 | 503 | 254.6 | - | 744780233 | accessory Sec system glycosylation protein GtfA | S. hyicus | 0E+00 |
| 1778 | 1812270 | 1814660 | 797 | 469.4 | - | 744780232 | accessory Sec system translocase SecA2 | S. hyicus | 0E+00 |
| 1779 | 1814650 | 1815555 | 302 | 77.6 | - | 744780231 | accessory secretory protein Asp3 | S. hyicus | 1E-157 |
| 1780 | 1815542 | 1817104 | 521 | 212.8 | - | 744780230 | accessory secretory protein Asp2 | S. hyicus | 0E+00 |
| 1781 | 1817094 | 1818650 | 519 | 200.2 | - | 744780229 | accessory secretory protein Asp1 | S. hyicus | 0E+00 |
| 1782 | 1818661 | 1819884 | 408 | 133.9 | - | 744780228 | accessory Sec system translocase SecY2 | S. hyicus | 0E+00 |
| 1783 | 1820226 | 1823033 | 936 | 560.2 | + |  | No Significant Match |  |  |
| 1784 | 1825742 | 1827334 | 531 | 264.1 | + | 744780226 | L-lactate permease | S. hyicus | 0E+00 |
| 1785 | 1827370 | 1828050 | 227 | 86.5 | - | 744780225 | 6-carboxyhexanoate--CoA ligase | S. hyicus | 5E-165 |
| 1786 | 1828043 | 1829155 | 371 | 110.1 | - | 744780224 | 8-amino-7-oxononanoate synthase | S. hyicus | 0E+00 |
| 1787 | 1829158 | 1830507 | 450 | 213.9 | - | 744780223 | adenosylmethionine-8-amino-7-oxononanoate aminotransferase | S. hyicus | 0E+00 |
| 1788 | 1830482 | 1831177 | 232 | 98.6 | - | 744780222 | ATP-dependent dethiobiotin synthetase | S. hyicus | 9E-163 |
| 1789 | 1831569 | 1831892 | 108 | 6.1 | - | P37248 | Transposase for insertion sequence element IS1086 | Ralstonia metallidurans | 4E-18 |
| 1790 | 1832077 | 1832589 | 171 | 76.3 | - |  | No Significant Match |  |  |
| 1791 | 1832700 | 1833053 | 118 | 69.3 | - |  | No Significant Match |  |  |
| 1792 | 1833113 | 1833703 | 197 | 90.6 | - |  | No Significant Match |  |  |
| 1793 | 1833708 | 1834757 | 350 | 176.6 | - | 746621932 | hypothetical protein, partial | S. hyicus | 1E-23 |
| 1794 | 1834747 | 1836642 | 632 | 217.6 | - | P96644 | Uncharacterized membrane protein YddG | Bacillus subtilis | 2E-40 |
| 1795 | 1836647 | 1836910 | 88 | 29.8 | - |  | No Significant Match |  |  |
| 1796 | 1836950 | 1837177 | 76 | 28 | - |  | No Significant Match |  |  |
| 1797 | 1837208 | 1838578 | 457 | 202.6 | - | P96634 | Ftsk domain-containing protein YdcQ | Bacillus subtilis | 1E-59 |
| 1798 | 1838605 | 1838883 | 93 | 21.9 | - |  | No Significant Match |  |  |
| 1799 | 1839154 | 1840332 | 393 | 182.2 | - | O31943 | SPBc2 prophage-derived uncharacterized HTH-type transcriptional regulator YonR | Bacillus subtilis | 6E-05 |
| 1800 | 1840355 | 1841092 | 246 | 119 | - |  | No Significant Match |  |  |
| 1801 | 1841206 | 1843701 | 832 | 406.8 | - | P96642 | Uncharacterized protein YddE | Bacillus subtilis | 0E+00 |
| 1802 | 1843738 | 1844121 | 128 | 24.9 | - |  | No Significant Match |  |  |
| 1803 | 1844128 | 1844388 | 87 | 51 | - |  | No Significant Match |  |  |
| 1804 | 1844393 | 1845445 | 351 | 146.9 | - | O31491 | Uncharacterized protein YddB | Bacillus subtilis | 5E-31 |
| 1805 | 1845518 | 1846597 | 360 | 106.5 | - | P96635 | Putative DNA relaxase NicK | Bacillus subtilis | 3E-69 |
| 1806 | 1846767 | 1847072 | 102 | 16.3 | - |  | No Significant Match |  |  |
| 1807 | 1847086 | 1847406 | 107 | 56.7 | - |  | No Significant Match |  |  |
| 1808 | 1847553 | 1847837 | 95 | 19.8 | - |  | No Significant Match |  |  |
| 1809 | 1847931 | 1848371 | 147 | 35 | + | 744780221 | LytTR family transcriptional regulator | S. hyicus | 1E-99 |
| 1810 | 1848368 | 1848826 | 153 | 50.8 | + | 744780220 | membrane protein | S. hyicus | 2E-103 |
| 1811 | 1849079 | 1849858 | 260 | 72.1 | + | 744780211 | glycosyl hydrolase | S. hyicus | 4E-173 |
| 1812 | 1850050 | 1850598 | 183 | 57.7 | + | 744780211 | glycosyl hydrolase | S. hyicus | 4E-118 |
| 1813 | 1850816 | 1852138 | 441 | 172.8 | + | 744780210 | manganese transporter | S. hyicus | 0E+00 |
| 1814 | 1852220 | 1853221 | 334 | 121.5 | - | 744780209 | hypothetical protein SHYC_00980 | S. hyicus | 0E+00 |
| 1815 | 1853235 | 1853543 | 103 | 22.1 | - | Q1WVJ7 | CRISPR-associated endoribonuclease Cas2 | Lactobacillus salivarius | 2E-27 |
| 1816 | 1853548 | 1854450 | 301 | 124.4 | - | 744780208 | CRISPR-associated protein | S. hyicus | 0E+00 |
| 1817 | 1854450 | 1857617 | 1056 | 458.8 | - | 744780207 | CRISPR-associated protein Cas9/Csn1 | S. hyicus | 0E+00 |
| 1818 | 1860367 | 1860789 | 141 | 83 | + | 744780206 | small heat shock protein | S. hyicus | 2E-95 |
| 1819 | 1860858 | 1860995 | 46 | 23.2 | - |  | No Significant Match |  |  |
| 1820 | 1861177 | 1862838 | 554 | 255.6 | + | 744780205 | FAD-binding dehydrogenase | S. hyicus | 0E+00 |
| 1821 | 1863262 | 1864851 | 530 | 259 | - | 744780204 | acyl--CoA ligase | S. hyicus | 0E+00 |
| 1822 | 1865358 | 1866365 | 336 | 152.3 | - | Q6GDG8 | Ornithine carbamoyltransferase, catabolic | S. aureus | 2E-144 |
| 1823 | 1866763 | 1867083 | 107 | 67.9 | + | 744780203 | hypothetical protein SHYC_00935 | S. hyicus | 8E-57 |
| 1824 | 1867315 | 1867941 | 209 | 118.2 | + | 744780202 | FMN-dependent NADH-azoreductase | S. hyicus | 6E-152 |
| 1825 | 1868179 | 1870140 | 654 | 267.8 | - | 744780201 | ABC transporter permease | S. hyicus | 0E+00 |
| 1826 | 1870142 | 1870891 | 250 | 127.8 | - | 744780200 | ABC transporter ATP-binding protein | S. hyicus | 1E-169 |
| 1827 | 1870981 | 1871886 | 302 | 110.5 | - | 744780199 | histidine kinase | S. hyicus | 0E+00 |
| 1828 | 1871901 | 1872566 | 222 | 87.1 | - | 744780198 | PhoB family transcriptional regulator | S. hyicus | 5E-151 |
| 1829 | 1872752 | 1873669 | 306 | 94.5 | - | 746621690 | hypothetical protein, partial | S. hyicus | 9E-56 |
| 1830 | 1873687 | 1874439 | 251 | 81.1 | - |  | No Significant Match |  |  |
| 1831 | 1874432 | 1875346 | 305 | 143.6 | - | P42332 | Bacitracin transport ATP-binding protein BcrA | Bacillus licheniformis | 4E-64 |
| 1832 | 1875429 | 1876097 | 223 | 92.6 | - | P45607 | Phosphate regulon transcriptional regulatory protein PhoB | Shigella flexneri | 7E-39 |
| 1833 | 1876343 | 1876969 | 209 | 113.7 | - | 744780196 | hypothetical protein SHYC_00895 | S. hyicus | 2E-87 |
| 1834 | 1877066 | 1877866 | 267 | 67 | - | 744780195 | hypothetical protein SHYC_00890 | S. hyicus | 3E-84 |
| 1835 | 1878488 | 1879288 | 267 | 58.4 | - |  | No Significant Match |  |  |
| 1836 | 1879851 | 1881233 | 461 | 195.9 | + | 744780194 | alanine glycine permease | S. hyicus | 0E+00 |
| 1837 | 1881525 | 1882025 | 167 | 64.5 | + | 744780193 | membrane protein | S. hyicus | 2E-104 |
| 1838 | 1882186 | 1883679 | 498 | 271.1 | + | 744780192 | malate:quinone oxidoreductase | S. hyicus | 0E+00 |
| 1839 | 1883697 | 1883807 | 37 | 19.5 | + | 744780191 | hypothetical protein SHYC_00870 | S. hyicus | 3E-18 |
| 1840 | 1883919 | 1885091 | 391 | 155.7 | - | O31577 | Uncharacterized MFS-type transporter YfhI | Bacillus subtilis | 3E-88 |
| 1841 | 1885392 | 1885850 | 153 | 53.4 | + | 744780190 | ArgR family transcriptional regulator | S. hyicus | 3E-91 |
| 1842 | 1886093 | 1887325 | 411 | 214.1 | + | 744780189 | arginine deiminase | S. hyicus | 0E+00 |
| 1843 | 1887351 | 1888355 | 335 | 200.9 | + | 744780188 | ornithine carbamoyltransferase | S. hyicus | 0E+00 |
| 1844 | 1888444 | 1889862 | 473 | 172.2 | + | 744780187 | putative arginine/ornithine antiporter | S. hyicus | 0E+00 |
| 1845 | 1889879 | 1890820 | 314 | 169.4 | + | 744780186 | carbamate kinase | S. hyicus | 0E+00 |
| 1846 | 1890840 | 1891535 | 232 | 60.1 | + | 744780185 | Crp/Fnr family transcriptional regulator | S. hyicus | 3E-170 |
| 1847 | 1891882 | 1893231 | 450 | 121.6 | - | 744780184 | transporter | S. hyicus | 0E+00 |
| 1848 | 1893225 | 1894334 | 370 | 188.4 | - | 744780183 | pyridine nucleotide-disulfide oxidoreductase | S. hyicus | 0E+00 |
| 1849 | 1894480 | 1895247 | 256 | 126.6 | - | 744780182 | tributyrin esterase | S. hyicus | 8E-162 |
| 1850 | 1895263 | 1895940 | 226 | 112 | - | 744780181 | glycine betaine/carnitine/choline transport system permease protein | S. hyicus | 1E-155 |
| 1851 | 1895940 | 1896884 | 315 | 158.6 | - | 744780180 | glycine/betaine ABC transporter substrate-binding protein | S. hyicus | 0E+00 |
| 1852 | 1896900 | 1897535 | 212 | 101.6 | - | 744780179 | glycine betaine/carnitine/choline ABC transporter permease | S. hyicus | 3E-145 |
| 1853 | 1897532 | 1898713 | 394 | 221.8 | - | 744780178 | putative glycine betaine/carnitine/choline transport ATP-binding protein | S. hyicus | 0E+00 |
| 1854 | 1899041 | 1899607 | 189 | 98.5 | - | P39897 | HTH-type transcriptional regulator MtrR | Neisseria gonorrhoeae | 4E-05 |
| 1855 | 1899754 | 1901094 | 447 | 200.8 | - | P76350 | Shikimate transporter | Escherichia coli | 9E-89 |
| 1856 | 1901409 | 1901579 | 57 | 10.4 | + |  | No Significant Match |  |  |
| 1857 | 1901811 | 1902026 | 72 | 24.6 | - |  | No Significant Match |  |  |
| 1858 | 1902143 | 1902862 | 240 | 90.1 | - | 744780177 | membrane protein | S. hyicus | 3E-36 |
| 1859 | 1902859 | 1903062 | 68 | 33 | - | 744780176 | XRE family transcriptional regulator | S. hyicus | 1E-36 |
| 1860 | 1903186 | 1904382 | 399 | 173.3 | - | 744780175 | pyridine nucleotide-disulfide oxidoreductase | S. hyicus | 0E+00 |
| 1861 | 1904503 | 1905186 | 228 | 114 | + | 744780174 | hypothetical protein SHYC_00785 | S. hyicus | 2E-114 |
| 1862 | 1905431 | 1906774 | 448 | 149.5 | - |  | No Significant Match |  |  |
| 1863 | 1906806 | 1908311 | 502 | 209.2 | - | 744780173 | deoxyribodipyrimidine photolyase | S. hyicus | 0E+00 |
| 1864 | 1908345 | 1908815 | 157 | 24.5 | - | 744780172 | TspO/MBR family protein | S. hyicus | 4E-78 |
| 1865 | 1908862 | 1910286 | 475 | 219.5 | - | 744780171 | NAD dependent epimerase/dehydratase | S. hyicus | 0E+00 |
| 1866 | 1910468 | 1910971 | 168 | 83.6 | - | 744780170 | hypothetical protein SHYC_00765 | S. hyicus | 1E-92 |
| 1867 | 1911156 | 1911653 | 166 | 46.1 | - | 744780169 | hypothetical protein SHYC_00760 | S. hyicus | 2E-46 |
| 1868 | 1911812 | 1912552 | 247 | 121.8 | + | 744780168 | NAD-dependent deacetylase | S. hyicus | 6E-137 |
| 1869 | 1912569 | 1913435 | 289 | 132.7 | - | 744780166 | alpha/beta hydrolase | S. hyicus | 3E-156 |
| 1870 | 1913563 | 1914738 | 392 | 240 | - | 744780348 | phosphopentomutase | S. hyicus | 0E+00 |
| 1871 | 1914768 | 1915430 | 221 | 158.7 | - | 744780164 | deoxyribose-phosphate aldolase | S. hyicus | 7E-154 |
| 1872 | 1915443 | 1916801 | 453 | 196.8 | - | 744780163 | major facilitator transporter | S. hyicus | 0E+00 |
| 1873 | 1916815 | 1917522 | 236 | 119.7 | - | 744780162 | purine-nucleoside phosphorylase | S. hyicus | 1E-172 |
| 1874 | 1917748 | 1918491 | 248 | 101.6 | - | 744780161 | GntR family transcriptional regulator | S. hyicus | 0E+00 |
| 1875 | 1918789 | 1919541 | 251 | 91.3 | - | 744780160 | putative CAAX protease | S. hyicus | 1E-160 |
| 1876 | 1919775 | 1920515 | 247 | 88.7 | - | 744780158 | CAAX amino terminal protease | S. hyicus | 1E-167 |
| 1877 | 1920542 | 1921597 | 352 | 94.9 | - | 744780157 | hypothetical protein SHYC_00700 | S. hyicus | 0E+00 |
| 1878 | 1922145 | 1922621 | 159 | 58.4 | - | 744780156 | putative acetyltransferase | S. hyicus | 9E-111 |
| 1879 | 1922677 | 1923381 | 235 | 100.8 | - | 744780155 | GntR family transcriptional regulator | S. hyicus | 7E-169 |
| 1880 | 1923539 | 1924981 | 481 | 180.1 | + | 744780154 | 6-phospho-beta-glucosidase | S. hyicus | 0E+00 |
| 1881 | 1925169 | 1925375 | 69 | 29.8 | - | 744780153 | XRE family transcriptional regulator | S. hyicus | 3E-40 |
| 1882 | 1925386 | 1925853 | 156 | 66.5 | - | 744780152 | hypothetical protein SHYC_00675 | S. hyicus | 2E-83 |
| 1883 | 1925981 | 1926478 | 166 | 68 | - | 746621660 | hypothetical protein, partial | S. hyicus | 4E-43 |
| 1884 | 1926691 | 1927557 | 289 | 131.6 | - | 744780151 | alpha/beta hydrolase | S. hyicus | 2E-154 |
| 1885 | 1927862 | 1928548 | 229 | 84.4 | + | P42421 | Transcriptional regulatory protein YxdJ | Bacillus subtilis | 4E-65 |
| 1886 | 1928538 | 1929527 | 330 | 121.1 | + | P42422 | Sensor histidine kinase YxdK | Bacillus subtilis | 3E-43 |
| 1887 | 1929714 | 1930469 | 252 | 131.4 | + | P42423 | ABC transporter ATP-binding protein YxdL | Bacillus subtilis | 2E-97 |
| 1888 | 1930456 | 1932309 | 618 | 129.6 | + | P42424 | ABC transporter permease protein YxdM | Bacillus subtilis | 1E-43 |
| 1889 | 1932528 | 1933517 | 330 | 199.1 | + | 744780149 | iron ABC transporter substrate-binding protein | S. hyicus | 0E+00 |
| 1890 | 1933529 | 1934524 | 332 | 121.3 | + | 744780148 | siderophore ABC transporter permease | S. hyicus | 0E+00 |
| 1891 | 1934521 | 1935519 | 333 | 117.2 | + | 744780147 | iron ABC transporter permease | S. hyicus | 0E+00 |
| 1892 | 1935836 | 1937368 | 511 | 260.5 | - | 744780146 | zinc ABC transporter substrate-binding protein | S. hyicus | 0E+00 |
| 1893 | 1937686 | 1939404 | 573 | 296.7 | + | 744780145 | putative IgG-binding protein | S. hyicus | 0E+00 |
| 1894 | 1939515 | 1940894 | 460 | 192.3 | + | 744780145 | putative IgG-binding protein | S. hyicus | 0E+00 |
| 1895 | 1940994 | 1942769 | 592 | 244 | - | 744780144 | hypothetical protein SHYC_00630 | S. hyicus | 0E+00 |
| 1896 | 1942933 | 1943523 | 197 | 66.5 | + | 744780143 | TetR family transcriptional regulator | S. hyicus | 3E-140 |
| 1897 | 1943579 | 1946386 | 936 | 468.5 | - | 744780142 | hypothetical protein, similar to phage infection protein | S. hyicus | 0E+00 |
| 1898 | 1946596 | 1948539 | 648 | 249.8 | + | 744780141 | N-acetylmuramoyl-L-alanine amidase | S. hyicus | 0E+00 |
| 1899 | 1948591 | 1950255 | 555 | 228.9 | - | 744780140 | surface protein | S. hyicus | 0E+00 |
| 1900 | 1950653 | 1951438 | 262 | 98.9 | - | 744780135 | tandem lipoprotein | S. hyicus | 3E-106 |
| 1901 | 1951567 | 1952364 | 266 | 104.5 | - | 744780132 | tandem lipoprotein | S. hyicus | 1E-140 |
| 1902 | 1952734 | 1952847 | 38 | 13.1 | - | 744780139 | hypothetical protein SHYC_00605 | S. hyicus | 5E-06 |
| 1903 | 1952847 | 1953491 | 215 | 70.8 | - | 744780136 | tandem lipoprotein | S. hyicus | 2E-103 |
| 1904 | 1953494 | 1953649 | 52 | 5 | - | 744780128 | hypothetical protein SHYC_00550 | S. hyicus | 3E-08 |
| 1905 | 1954116 | 1954844 | 243 | 105.2 | - | 744780133 | tandem lipoprotein | S. hyicus | 2E-116 |
| 1906 | 1955294 | 1955710 | 139 | 58 | - | 744780127 | cytidine deaminase | S. hyicus | 4E-95 |
| 1907 | 1955723 | 1956301 | 193 | 82.1 | - | 744780126 | transcriptional regulator | S. hyicus | 6E-126 |
| 1908 | 1956884 | 1957582 | 233 | 119.2 | + | 744780124 | superantigen-like protein | S. hyicus | 1E-70 |
| 1909 | 1957610 | 1959703 | 698 | 418.4 | + | 744780123 | surface protein | S. hyicus | 0E+00 |
| 1910 | 1959935 | 1961452 | 506 | 293.8 | + | 744780122 | surface protein | S. hyicus | 0E+00 |
| 1911 | 1961592 | 1962308 | 239 | 95.2 | + | 744780121 | superantigen-like protein | S. hyicus | 3E-131 |
| 1912 | 1962665 | 1966216 | 1184 | 582.1 | + | 746621644 | hypothetical protein, partial | S. hyicus | 0E+00 |
| 1913 | 1966470 | 1967159 | 230 | 121.7 | + | 744780119 | superantigen-like protein | S. hyicus | 6E-140 |
| 1914 | 1967330 | 1968040 | 237 | 89.7 | + | 746621641 | hypothetical protein, partial | S. hyicus | 3E-153 |
| 1915 | 1968245 | 1968958 | 238 | 127 | + | 744780117 | superantigen-like protein | S. hyicus | 4E-164 |
| 1916 | 1969263 | 1969424 | 54 | 8.8 | - |  | No Significant Match |  |  |
| 1917 | 1969450 | 1969995 | 182 | 47.1 | - | 744780116 | ribonucleoside-triphosphate reductase activating protein | S. hyicus | 3E-114 |
| 1918 | 1969970 | 1971832 | 621 | 287.1 | - | 744780115 | ribonucleoside-triphosphate reductase | S. hyicus | 0E+00 |
| 1919 | 1972076 | 1973002 | 309 | 183.3 | - | 744780114 | L-lactate dehydrogenase | S. hyicus | 0E+00 |
| 1920 | 1973419 | 1974309 | 297 | 172.6 | - | 744780110 | acid phosphatase | S. hyicus | 0E+00 |
| 1921 | 1974665 | 1975165 | 167 | 38.1 | + | 744780109 | hypothetical protein SHYC_00430 | S. hyicus | 6E-118 |
| 1922 | 1975259 | 1977487 | 743 | 252.6 | + | 744780108 | AraC family transcriptional regulator | S. hyicus | 0E+00 |
| 1923 | 1977626 | 1978813 | 396 | 236.4 | + | 744780107 | amidohydrolase | S. hyicus | 0E+00 |
| 1924 | 1978806 | 1980194 | 463 | 208 | + | 744780106 | Major Facilitator Superfamily protein | S. hyicus | 0E+00 |
| 1925 | 1980407 | 1981165 | 253 | 166.5 | + | 744780103 | 3-ketoacyl-ACP reductase | S. hyicus | 1E-173 |
| 1926 | 1981276 | 1982040 | 255 | 85.5 | - | 744780102 | siderophore biosynthesis protein SbnI | S. hyicus | 0E+00 |
| 1927 | 1982000 | 1983286 | 429 | 161 | - | 744780101 | siderophore staphylobactin biosynthesis protein SbnH | S. hyicus | 0E+00 |
| 1928 | 1983283 | 1984056 | 258 | 144.7 | - | 744780100 | siderophore biosynthesis protein SbnG | S. hyicus | 0E+00 |
| 1929 | 1984037 | 1985806 | 590 | 251.7 | - | 744780099 | siderophore biosynthesis protein SbnF | S. hyicus | 0E+00 |
| 1930 | 1985793 | 1987487 | 565 | 196.6 | - | 744780098 | siderophore biosynthesis protein SbnE | S. hyicus | 0E+00 |
| 1931 | 1987468 | 1988736 | 423 | 102 | - | 744780097 | siderophore biosynthesis protein SbnD | S. hyicus | 0E+00 |
| 1932 | 1988729 | 1990489 | 587 | 220.3 | - | 746617730 | siderophore biosynthesis protein SbnC | S. hyicus | 0E+00 |
| 1933 | 1990533 | 1991543 | 337 | 183.7 | - | 744780095 | 2,3-diaminopropionate biosynthesis protein SbnB | S. hyicus | 0E+00 |
| 1934 | 1991656 | 1992642 | 329 | 159.7 | - | 744780094 | 2,3-diaminopropionate biosynthesis protein SbnA | S. hyicus | 0E+00 |
| 1935 | 1992894 | 1993595 | 234 | 85.5 | - | 744780093 | two-component system, OmpR family, KDP operon response regulator KdpE | S. hyicus | 6E-172 |
| 1936 | 1993592 | 1996102 | 837 | 326.6 | - | 744780092 | Osmosensitive K+ channel histidine kinase KdpD | S. hyicus | 0E+00 |
| 1937 | 1996275 | 1996379 | 35 | 14.2 | + |  | No Significant Match |  |  |
| 1938 | 1996471 | 1998141 | 557 | 174.6 | + | 744780091 | potassium-transporting ATPase subunit A | S. hyicus | 0E+00 |
| 1939 | 1998138 | 2000132 | 665 | 295.8 | + | 744780090 | potassium-transporting ATPase subunit B | S. hyicus | 0E+00 |
| 1940 | 2000147 | 2000728 | 194 | 59.6 | + | 744780089 | potassium-transporting ATPase subunit C | S. hyicus | 2E-135 |
| 1941 | 2001246 | 2002679 | 478 | 249.1 | + | 744780088 | immunoglobulin G-binding protein | S. hyicus | 0E+00 |
| 1942 | 2003069 | 2003245 | 59 | 17.9 | - |  | No Significant Match |  |  |
| 1943 | 2003214 | 2003462 | 83 | 48.6 | - | 746621660 | hypothetical protein, partial | S. hyicus | 2E-26 |
| 1944 | 2003836 | 2004174 | 113 | 43.5 | - | Q9QY93 | dCTP pyrophosphatase 1 | Mus musculus | 9E-18 |
| 1945 | 2004167 | 2004502 | 112 | 22.2 | - | Q12179 | Uncharacterized protein YPL245W | Saccharomyces cerevisiae | 8E-14 |
| 1946 | 2004701 | 2005420 | 240 | 39.6 | - | Q45592 | Probable peptide export permease protein YydJ | Bacillus subtilis | 3E-81 |
| 1947 | 2005440 | 2006066 | 209 | 60.4 | - | Q45593 | Probable peptide export ATP-binding protein YydI | Bacillus subtilis | 1E-65 |
| 1948 | 2006357 | 2006944 | 196 | 61.8 | + |  | No Significant Match |  |  |
| 1949 | 2007239 | 2007481 | 81 | 25 | + |  | No Significant Match |  |  |
| 1950 | 2008038 | 2011160 | 1041 | 547.2 | - | 744780073 | type I restriction-modification system endonuclease HsdR | S. hyicus | 0E+00 |
| 1951 | 2011144 | 2012439 | 432 | 167.9 | - | 744780072 | type I restriction-modification system specificity determinant protein | S. hyicus | 3E-63 |
| 1952 | 2012429 | 2013940 | 504 | 298.6 | - | 744780071 | type I restriction-modification system, M subunit | S. hyicus | 0E+00 |
| 1953 | 2014101 | 2015495 | 465 | 166.1 | + | 744780070 | hypothetical protein SHYC_00225 | S. hyicus | 0E+00 |
| 1954 | 2015789 | 2016076 | 96 | 3.1 | - | 746617687 | hypothetical protein | S. hyicus | 2E-42 |
| 1955 | 2016414 | 2016806 | 131 | 11.3 | - |  | No Significant Match |  |  |
| 1956 | 2017034 | 2017513 | 160 | 75.8 | - | 744780069 | ribosomal RNA large subunit methyltransferase H | S. hyicus | 1E-112 |
| 1957 | 2017910 | 2018308 | 133 | 44.7 | - | 744780068 | arsenate reductase | S. hyicus | 8E-94 |
| 1958 | 2018326 | 2019615 | 430 | 151 | - | 744780067 | arsenical pump membrane protein ArsB | S. hyicus | 0E+00 |
| 1959 | 2019612 | 2019932 | 107 | 34.4 | - | 744780066 | ArsR family transcriptional regulator | S. hyicus | 8E-67 |
| 1960 | 2020045 | 2020827 | 261 | 105.4 | - | 744780065 | metallo-beta-lactamase family protein YycJ | S. hyicus | 0E+00 |
| 1961 | 2020853 | 2021629 | 259 | 101.1 | - | 744780064 | YycI family protein | S. hyicus | 0E+00 |
| 1962 | 2021626 | 2022912 | 429 | 165.8 | - | 744780063 | YycH family protein | S. hyicus | 0E+00 |
| 1963 | 2022953 | 2024785 | 611 | 314.7 | - | 744780062 | sensor histidine kinase | S. hyicus | 0E+00 |
| 1964 | 2024798 | 2025499 | 234 | 131.9 | - | 744780061 | PhoP family transcriptional regulator | S. hyicus | 5E-169 |
| 1965 | 2026208 | 2026321 | 38 | 10.3 | + | 744780060 | hypothetical protein SHYC_00155 | S. hyicus | 6E-15 |
| 1966 | 2026570 | 2027856 | 429 | 286.6 | - | 744780059 | adenylosuccinate synthetase | S. hyicus | 0E+00 |
| 1967 | 2028161 | 2029561 | 467 | 270.1 | - | 744780058 | replicative DNA helicase | S. hyicus | 0E+00 |
| 1968 | 2029853 | 2030299 | 149 | 107.2 | - | 744780057 | 50S ribosomal protein L9 | S. hyicus | 1E-100 |
| 1969 | 2030296 | 2032263 | 656 | 371.4 | - | 744780056 | DHH family phosphoesterase | S. hyicus | 0E+00 |
| 1970 | 2032275 | 2033180 | 302 | 87.1 | - | 746617656 | membrane protein | S. hyicus | 0E+00 |
| 1971 | 2033520 | 2034557 | 346 | 113.3 | - | 744780054 | homoserine O-acetyltransferase | S. hyicus | 0E+00 |
| 1972 | 2034719 | 2035048 | 110 | 39.4 | - | 744780053 | putative branched-chain amino acid transport protein | S. hyicus | 1E-68 |
| 1973 | 2035045 | 2035737 | 231 | 103.7 | - | 744780052 | putative branched-chain amino acid permease | S. hyicus | 9E-162 |
| 1974 | 2036038 | 2036421 | 128 | 80.4 | - | 744780051 | gas vesicle protein GvpU | S. hyicus | 5E-86 |
| 1975 | 2036440 | 2037846 | 469 | 370.8 | - | 744780050 | hypothetical protein SHYC_00105 | S. hyicus | 9E-10 |
| 1976 | 2037852 | 2038703 | 284 | 103.6 | - | 744780049 | hypothetical protein SHYC_00100 | S. hyicus | 3E-123 |
| 1977 | 2038672 | 2039085 | 138 | 99.7 | - | 744780048 | gas vesicle protein GvpJ | S. hyicus | 5E-90 |
| 1978 | 2039111 | 2039401 | 97 | 67 | - | 744780047 | gas vesicle protein GvpK | S. hyicus | 1E-58 |
| 1979 | 2039340 | 2039645 | 102 | 55.4 | - | 744780046 | gas vesicle protein GvpS | S. hyicus | 3E-64 |
| 1980 | 2039664 | 2040473 | 270 | 135.5 | - | 744780045 | gas vesicle protein GvpL | S. hyicus | 0E+00 |
| 1981 | 2040475 | 2040729 | 85 | 60 | - | 744780044 | gas vesicle protein GvpG | S. hyicus | 1E-51 |
| 1982 | 2040729 | 2041496 | 256 | 155.8 | - | 744780043 | gas vesicle protein GvpL/GvpF | S. hyicus | 0E+00 |
| 1983 | 2041595 | 2041873 | 93 | 55.3 | - | 744780042 | gas vesicle protein GvpR | S. hyicus | 3E-60 |
| 1984 | 2041958 | 2042305 | 116 | 85 | - | 744780041 | gas vesicle synthesis protein GvpA | S. hyicus | 6E-74 |
| 1985 | 2042397 | 2042885 | 163 | 83.5 | - | 744780040 | gas vesicle protein GvpQ | S. hyicus | 4E-96 |
| 1986 | 2043770 | 2045050 | 427 | 283.1 | - | 744780039 | seryl-tRNA synthetase | S. hyicus | 0E+00 |
| 1987 | 2045356 | 2046858 | 501 | 290.4 | - | 744780038 | histidine ammonia-lyase | S. hyicus | 0E+00 |
| 1988 | 2047129 | 2047941 | 271 | 155.8 | + | 744780037 | carbohydrate kinase | S. hyicus | 0E+00 |
| 1989 | 2047984 | 2050644 | 887 | 562.7 | - | 744780036 | DNA gyrase subunit A | S. hyicus | 0E+00 |
| 1990 | 2050681 | 2052603 | 641 | 380.9 | - | 746617625 | DNA gyrase subunit B | S. hyicus | 0E+00 |
| 1991 | 2052625 | 2053740 | 372 | 158.2 | - | 744780034 | recombinase RecF | S. hyicus | 0E+00 |
| 1992 | 2053737 | 2053976 | 80 | 43.8 | - | 744780033 | S4 domain-containing protein | S. hyicus | 2E-50 |
| 1993 | 2054083 | 2054787 | 235 | 112.5 | - | 744780032 | protein-disulfide isomerase | S. hyicus | 2E-127 |
| 1994 | 2054933 | 2056066 | 378 | 197.4 | - | 744780031 | DNA polymerase III subunit beta | S. hyicus | 0E+00 |
| 1995 | 2056247 | 2057587 | 447 | 220.6 | - | 744780030 | chromosomal replication initiation protein | S. hyicus | 0E+00 |
| 1996 | 2058127 | 2058264 | 46 | 20.9 | + | Q49UI2 | 50S ribosomal protein L34 | S. saprophyticus | 3E-23 |
| 1997 | 2058416 | 2058763 | 116 | 32.4 | + | 744782306 | ribonuclease P protein component | S. hyicus | 1E-71 |
| 1998 | 2058947 | 2060329 | 461 | 297.8 | + | 744782305 | tRNA modification GTPase MnmE | S. hyicus | 0E+00 |
| 1999 | 2060346 | 2062220 | 625 | 403.7 | + | 744782304 | tRNA uridine 5-carboxymethylaminomethyl modification enzyme MnmG | S. hyicus | 0E+00 |
| 2000 | 2062221 | 2062940 | 240 | 128.6 | + | 744782303 | ribosomal RNA small subunit methyltransferase G | S. hyicus | 2E-166 |
| 2001 | 2062980 | 2063837 | 286 | 160.3 | + | 744782302 | nucleoid occlusion protein | S. hyicus | 0E+00 |
| 2002 | 2064189 | 2064716 | 176 | 93.6 | - | 744782301 | carboxylesterase/hydrolase MhqD | S. hyicus | 1E-115 |
| 2003 | 2064717 | 2065352 | 212 | 85.2 | - | 744782300 | hypothetical protein SHYC_12010 | S. hyicus | 2E-143 |
| 2004 | 2065377 | 2066336 | 320 | 197 | - | 744782299 | putative ring-cleaving dioxygenase MhqA | S. hyicus | 0E+00 |
| 2005 | 2066412 | 2066840 | 143 | 73.5 | - | 744782298 | HTH-type transcriptional regulator MhqR | S. hyicus | 2E-97 |
| 2006 | 2067117 | 2068034 | 306 | 157.9 | + | 744782297 | glycyl-glycine endopeptidase LytM | S. hyicus | 0E+00 |
| 2007 | 2068199 | 2068594 | 132 | 73 | + | 744782295 | hypothetical protein SHYC_11985 | S. hyicus | 6E-77 |
| 2008 | 2068871 | 2069368 | 166 | 46.3 | - | 744782294 | hypothetical protein SHYC_11980 | S. hyicus | 5E-115 |
| 2009 | 2069687 | 2069791 | 35 | 14.3 | - | 744782294 | hypothetical protein SHYC_11980 | S. hyicus | 3E-13 |
| 2010 | 2070024 | 2070539 | 172 | 109.4 | - | 744782293 | YceI domain protein | S. hyicus | 1E-116 |
| 2011 | 2070969 | 2072426 | 486 | 199.3 | + | O86233 | Uncharacterized membrane protein HI_1126.1 | Haemophilus influenzae | 5E-87 |
| 2012 | 2072604 | 2073056 | 151 | 99.9 | + | 744782292 | urease accessory protein UreE | S. hyicus | 1E-97 |
| 2013 | 2073049 | 2073738 | 230 | 117.8 | + | 744782291 | urease accessory protein UreF | S. hyicus | 4E-160 |
| 2014 | 2073750 | 2074361 | 204 | 129.7 | + | 744782290 | urease accessory protein UreG | S. hyicus | 4E-144 |
| 2015 | 2074363 | 2075196 | 278 | 116.8 | + | 744782289 | urease accessory protein UreD | S. hyicus | 0E+00 |
| 2016 | 2075318 | 2076532 | 405 | 197.6 | - | 744782287 | hypothetical protein SHYC_11945 | S. hyicus | 0E+00 |
| 2017 | 2076791 | 2077414 | 208 | 65.3 | - | 744782286 | LysE family L-lysine exporter | S. hyicus | 2E-128 |
| 2018 | 2077411 | 2077674 | 88 | 16.3 | - |  | No Significant Match |  |  |
| 2019 | 2077817 | 2078044 | 76 | 22.7 | - | 744782285 | membrane protein | S. hyicus | 3E-43 |
| 2020 | 2078364 | 2079107 | 248 | 113.9 | - | 744782284 | cyclase family protein | S. hyicus | 1E-173 |
| 2021 | 2079132 | 2081375 | 748 | 462.9 | - | 744782283 | 5-methyltetrahydropteroyltriglutamate-- homocysteine methyltransferase | S. hyicus | 0E+00 |
| 2022 | 2081372 | 2083213 | 614 | 251.7 | - | 744782282 | bifunctional homocysteine S-methyltransferase/5,10-methylenetetrahydrofolate reductase protein | S. hyicus | 0E+00 |
| 2023 | 2083167 | 2084342 | 392 | 169.6 | - | 744782281 | cystathionine beta-lyase MetC | S. hyicus | 0E+00 |
| 2024 | 2084339 | 2085442 | 368 | 191.3 | - | 744782280 | cystathionine gamma-synthase/O-acetylhomoserine (thiol)-lyase | S. hyicus | 0E+00 |
| 2025 | 2086001 | 2086828 | 276 | 127.5 | + | 744782279 | chromosome partitioning protein ParB | S. hyicus | 3E-174 |
| 2026 | 2086856 | 2087746 | 297 | 145.2 | + | 744782278 | mechanosensitive ion channel protein MscS | S. hyicus | 0E+00 |
| 2027 | 2087778 | 2087984 | 69 | 26.6 | + | 744782277 | hypothetical protein SHYC_11895 | S. hyicus | 1E-42 |
| 2028 | 2087991 | 2089088 | 366 | 267.8 | + | 744782276 | ribosome-binding ATPase YchF | S. hyicus | 0E+00 |
| 2029 | 2089135 | 2089356 | 74 | 11 | - | 744782275 | hypothetical protein SHYC_11888 | S. hyicus | 7E-43 |
| 2030 | 2089748 | 2090383 | 212 | 126.5 | - | 744782274 | NADH-flavin reductase family protein | S. hyicus | 7E-130 |
| 2031 | 2090593 | 2090889 | 99 | 57.8 | + | 744782273 | 30S ribosomal protein S6 | S. hyicus | 1E-63 |
| 2032 | 2090913 | 2091419 | 169 | 77 | + | 744782272 | single-stranded DNA-binding protein | S. hyicus | 1E-119 |
| 2033 | 2091465 | 2091707 | 81 | 55.2 | + | 744782271 | 30S ribosomal protein S18 | S. hyicus | 2E-52 |
| 2034 | 2091864 | 2092565 | 234 | 81.7 | + | 744782270 | putative metal-dependent membrane protease | S. hyicus | 3E-108 |
| 2035 | 2092638 | 2093015 | 126 | 61.9 | - | 744782269 | hypothetical protein SHYC_11860 | S. hyicus | 2E-70 |
| 2036 | 2093170 | 2093472 | 101 | 46.5 | + | 744782268 | PadR family transcriptional regulator | S. hyicus | 3E-61 |
| 2037 | 2093485 | 2094642 | 386 | 166 | + | 744782267 | putative chromate transporter protein ChrA | S. hyicus | 0E+00 |
| 2038 | 2094766 | 2095962 | 399 | 233.2 | + | P42100 | Glycerate kinase | Bacillus subtilis | 1E-94 |
| 2039 | 2095964 | 2097361 | 466 | 130.3 | + | 744780484 | transporter | S. hyicus | 1E-24 |
| 2040 | 2097371 | 2098048 | 226 | 133.3 | + | O34903 | Uncharacterized transcriptional regulatory protein YkoG | Bacillus subtilis | 1E-84 |
| 2041 | 2098053 | 2099423 | 457 | 181.9 | + | O34638 | Sensor histidine kinase YkoH | Bacillus subtilis | 6E-91 |
| 2042 | 2099407 | 2100105 | 233 | 94.8 | + |  | No Significant Match |  |  |
| 2043 | 2100240 | 2101232 | 331 | 173.2 | + | 744782266 | biotin synthase | S. hyicus | 0E+00 |
| 2044 | 2101380 | 2102330 | 317 | 185.9 | + | 744782265 | hypothetical protein SHYC_11840 | S. hyicus | 0E+00 |
| 2045 | 2102451 | 2102924 | 158 | 32.9 | - | 744782264 | BioX protein | S. hyicus | 3E-92 |
| 2046 | 2103136 | 2104659 | 508 | 321 | - | 744782263 | alkyl hydroperoxide reductase subunit F | S. hyicus | 0E+00 |
| 2047 | 2105001 | 2105570 | 190 | 116.3 | - | 744782262 | alkyl hydroperoxide reductase subunit C | S. hyicus | 2E-139 |
| 2048 | 2105940 | 2106716 | 259 | 127 | - | 744782261 | putative 2-keto-4-pentenoate hydratase | S. hyicus | 4E-164 |
| 2049 | 2106858 | 2107613 | 252 | 144.1 | + | 744782260 | NADPH-dependent oxidoreductase | S. hyicus | 7E-173 |
| 2050 | 2107654 | 2109045 | 464 | 230.1 | - | 744782259 | L-cystine uptake protein TcyP | S. hyicus | 0E+00 |
| 2051 | 2109120 | 2110226 | 369 | 163.7 | - | 744782258 | glycine oxidase | S. hyicus | 0E+00 |
| 2052 | 2110438 | 2111040 | 201 | 121.9 | - | 744782257 | hypothetical protein SHYC_11800 | S. hyicus | 5E-136 |
| 2053 | 2111153 | 2111827 | 225 | 112.9 | - | 744782256 | hypothetical protein SHYC_11795 | S. hyicus | 1E-154 |
| 2054 | 2111972 | 2112382 | 137 | 95.9 | - | 744782255 | hypothetical protein SHYC_11790 | S. hyicus | 4E-92 |
| 2055 | 2112976 | 2113557 | 194 | 95.1 | + | 744782254 | xanthine phosphoribosyltransferase | S. hyicus | 7E-137 |
| 2056 | 2113557 | 2114825 | 423 | 216.8 | + | 744782253 | xanthine permease | S. hyicus | 0E+00 |
| 2057 | 2114861 | 2116327 | 489 | 347.1 | + | 744782252 | inosine-5-monophosphate dehydrogenase | S. hyicus | 0E+00 |
| 2058 | 2116348 | 2117889 | 514 | 312.1 | + | 744782251 | GMP synthase | S. hyicus | 0E+00 |
| 2059 | 2118053 | 2118355 | 101 | 49.4 | + |  | No Significant Match |  |  |
| 2060 | 2118817 | 2119374 | 186 | 106 | + |  | No Significant Match |  |  |
| 2061 | 2119392 | 2119583 | 64 | 8.2 | + |  | No Significant Match |  |  |
| 2062 | 2119771 | 2120094 | 108 | 40.9 | + |  | No Significant Match |  |  |
| 2063 | 2121219 | 2121683 | 155 | 82.3 | + |  | No Significant Match |  |  |
| 2064 | 2121903 | 2122427 | 175 | 38.9 | + |  | No Significant Match |  |  |
| 2065 | 2122424 | 2122513 | 30 | 2.9 | + |  | No Significant Match |  |  |
| 2066 | 2122696 | 2123616 | 307 | 79.4 | - | 9757655 | exfoliative toxin A | S. hyicus | 0E+00 |
| 2067 | 2123953 | 2124228 | 92 | 41.4 | + | 744782247 | hypothetical protein SHYC_11750 | S. hyicus | 3E-49 |
| 2068 | 2124579 | 2125274 | 232 | 64.2 | - |  | No Significant Match |  |  |
| 2069 | 2125632 | 2125733 | 34 | 4.6 | - |  | No Significant Match |  |  |
| 2070 | 2125687 | 2126241 | 185 | 90.1 | - |  | No Significant Match |  |  |
| 2071 | 2126483 | 2127457 | 325 | 148.5 | + | 744782244 | catabolite control protein A | S. hyicus | 0E+00 |
| 2072 | 2127559 | 2128521 | 321 | 168.2 | + | 744782243 | putative fructokinase | S. hyicus | 0E+00 |
| 2073 | 2128538 | 2129956 | 473 | 240 | + | 744782242 | PTS sugar transporter subunit IIBC | S. hyicus | 0E+00 |
| 2074 | 2130061 | 2130669 | 203 | 50 | - | 744782241 | membrane protein | S. hyicus | 8E-142 |
| 2075 | 2130840 | 2133239 | 800 | 359.1 | + | 744782240 | hyaluronate lyase | S. hyicus | 0E+00 |
| 2076 | 2133489 | 2133587 | 33 | 0.8 | - |  | No Significant Match |  |  |
| 2077 | 2133712 | 2133963 | 84 | 42.1 | + |  | No Significant Match |  |  |
| 2078 | 2134232 | 2134570 | 113 | 47.6 | + | 744782239 | membrane protein | S. hyicus | 1E-60 |
| 2079 | 2134684 | 2135061 | 126 | 28.9 | + | 744782238 | hypothetical protein SHYC_11700 | S. hyicus | 2E-61 |
| 2080 | 2135065 | 2135385 | 107 | 59.7 | + | 744782237 | heme-degrading monooxygenase IsdI | S. hyicus | 6E-74 |
| 2081 | 2135689 | 2136471 | 261 | 112.2 | + | 744782235 | iron complex transport system substrate-binding protein | S. hyicus | 0E+00 |
| 2082 | 2136738 | 2137529 | 264 | 97.8 | + | 744782234 | putative glutamyl-endopeptidase | S. hyicus | 4E-135 |
| 2083 | 2137823 | 2139316 | 498 | 255.9 | + | 744782233 | coagulase-related protein | S. hyicus | 0E+00 |
| 2084 | 2139878 | 2141380 | 501 | 255.2 | + | 744782232 | coagulase-related protein | S. hyicus | 1E-102 |
| 2085 | 2141632 | 2142315 | 228 | 87.2 | + | 744782231 | YjjG/YfnB family hydrolase | S. hyicus | 2E-154 |
| 2086 | 2142408 | 2142989 | 194 | 113 | - | 746621474 | hypothetical protein | S. hyicus | 3E-67 |
| 2087 | 2143252 | 2144061 | 270 | 83.1 | + | 744782230 | hypothetical protein SHYC_11655 | S. hyicus | 0E+00 |
| 2088 | 2144071 | 2144820 | 250 | 125.8 | + | 744782229 | hypothetical protein SHYC_11650 | S. hyicus | 2E-175 |
| 2089 | 2144893 | 2146185 | 431 | 170.8 | + | 744782228 | hypothetical protein SHYC_11645 | S. hyicus | 0E+00 |
| 2090 | 2146214 | 2147812 | 533 | 313.7 | + | 744782227 | ABC-type dipeptide/oligopeptide/nickel transport systems, substrate binding lipoprotein | S. hyicus | 0E+00 |
| 2091 | 2147824 | 2148756 | 311 | 109 | + | 744782226 | ABC-type dipeptide/oligopeptide/nickel transport systems, permease component | S. hyicus | 0E+00 |
| 2092 | 2148757 | 2149623 | 289 | 121.3 | + | 744782225 | ABC-type dipeptide/oligopeptide/nickel transport systems, permease component | S. hyicus | 0E+00 |
| 2093 | 2149620 | 2150438 | 273 | 107.7 | + | 744782224 | ABC-type dipeptide/oligopeptide/nickel transport systems, ATP-binding protein | S. hyicus | 0E+00 |
| 2094 | 2150432 | 2151172 | 247 | 127.9 | + | 744782223 | ABC-type dipeptide/oligopeptide/nickel transport systems, ATP-binding protein | S. hyicus | 0E+00 |
| 2095 | 2151184 | 2152374 | 397 | 185.6 | + | 744782222 | major facilitator superfamily transporter | S. hyicus | 0E+00 |
| 2096 | 2152443 | 2153480 | 346 | 179.3 | - |  | No Significant Match |  |  |
| 2097 | 2153502 | 2154152 | 217 | 89.4 | - | 744782221 | hypothetical protein SHYC_11610 | S. hyicus | 2E-07 |
| 2098 | 2154932 | 2155252 | 107 | 45.7 | + | 744782220 | small multidrug resistance protein SMR | S. hyicus | 3E-49 |
| 2099 | 2155253 | 2155567 | 105 | 46 | + | 744782219 | transporter related protein | S. hyicus | 3E-52 |
| 2100 | 2155598 | 2155783 | 62 | 24.8 | + | 744782218 | hypothetical protein SHYC_11595 | S. hyicus | 9E-30 |
| 2101 | 2155860 | 2156417 | 186 | 78.8 | + | 744782217 | membrane protein | S. hyicus | 8E-128 |
| 2102 | 2156545 | 2157414 | 290 | 101.8 | - | 744782216 | 2-dehydropantoate 2-reductase | S. hyicus | 1E-173 |
| 2103 | 2157481 | 2158293 | 271 | 148.9 | + | 744782215 | 3-methyl-2-oxobutanoate hydroxymethyltransferase | S. hyicus | 0E+00 |
| 2104 | 2158286 | 2159143 | 286 | 115.1 | + | 744782214 | pantoate--beta-alanine ligase | S. hyicus | 0E+00 |
| 2105 | 2159140 | 2159523 | 128 | 73.7 | + | 744782213 | aspartate alpha-decarboxylase | S. hyicus | 1E-82 |
| 2106 | 2159572 | 2160447 | 292 | 89.3 | - | 744782212 | hypothetical protein SHYC_11565 | S. hyicus | 0E+00 |
| 2107 | 2160751 | 2161044 | 98 | 68 | + | 744782211 | type VII secretion protein EsxA | S. hyicus | 3E-63 |
| 2108 | 2161246 | 2164323 | 1026 | 514.7 | + | 744782210 | type VII secretion protein EsaA | S. hyicus | 0E+00 |
| 2109 | 2164323 | 2164757 | 145 | 45.1 | + | 744782209 | type VII secretion protein EssA | S. hyicus | 1E-96 |
| 2110 | 2164735 | 2164986 | 84 | 18.4 | + | 744782208 | type VII secretion protein EsaB | S. hyicus | 8E-51 |
| 2111 | 2164999 | 2166315 | 439 | 238.9 | + | 744782207 | type VII secretion protein EssB | S. hyicus | 0E+00 |
| 2112 | 2166339 | 2170772 | 1478 | 743.3 | + | 744782206 | cell division protein FtsK | S. hyicus | 0E+00 |
| 2113 | 2170778 | 2171077 | 100 | 40.7 | + |  | No Significant Match |  |  |
| 2114 | 2171087 | 2171488 | 134 | 67.9 | + |  | No Significant Match |  |  |
| 2115 | 2171502 | 2173100 | 533 | 223.7 | + | 744782203 | transposase | S. hyicus | 4E-44 |
| 2116 | 2173112 | 2173792 | 227 | 49.5 | + |  | No Significant Match |  |  |
| 2117 | 2173815 | 2174264 | 150 | 67.3 | + | 744782201 | hypothetical protein SHYC_11510 | S. hyicus | 4E-34 |
| 2118 | 2174257 | 2174706 | 150 | 63.9 | + | 744782200 | hypothetical protein SHYC_11505 | S. hyicus | 3E-29 |
| 2119 | 2174720 | 2175328 | 203 | 87.5 | + | 744782198 | membrane protein | S. hyicus | 3E-08 |
| 2120 | 2175467 | 2176066 | 200 | 76.6 | + | 744782198 | membrane protein | S. hyicus | 5E-135 |
| 2121 | 2176435 | 2177085 | 217 | 62.2 | + | 744782202 | hypothetical protein SHYC_11515 | S. hyicus | 3E-24 |
| 2122 | 2177212 | 2177877 | 222 | 47.9 | + | 744782202 | hypothetical protein SHYC_11515 | S. hyicus | 1E-115 |
| 2123 | 2177889 | 2178554 | 222 | 49.4 | + | 744782202 | hypothetical protein SHYC_11515 | S. hyicus | 4E-64 |
| 2124 | 2178566 | 2179231 | 222 | 70.6 | + | 744782202 | hypothetical protein SHYC_11515 | S. hyicus | 3E-57 |
| 2125 | 2179519 | 2182143 | 875 | 549.5 | + | 744782197 | pyruvate phosphate dikinase | S. hyicus | 0E+00 |
| 2126 | 2182145 | 2182951 | 269 | 106.2 | + | 744782196 | kinase/pyrophosphorylase | S. hyicus | 0E+00 |
| 2127 | 2183160 | 2184488 | 443 | 190.4 | + | 744782195 | sodium-dependent transporter | S. hyicus | 0E+00 |
| 2128 | 2184596 | 2185933 | 446 | 131.3 | - | 744782194 | carboxylesterase | S. hyicus | 0E+00 |
| 2129 | 2185945 | 2186364 | 140 | 41.6 | - | 746621972 | GCN5 family acetyltransferase | S. hyicus | 4E-94 |
| 2130 | 2186524 | 2187435 | 304 | 165.8 | + | 744782192 | O-acetylserine dependent cystathionine beta-synthase | S. hyicus | 0E+00 |
| 2131 | 2187428 | 2188567 | 380 | 219 | + | 744782191 | cystathionine gamma-synthase | S. hyicus | 0E+00 |
| 2132 | 2188973 | 2189989 | 339 | 144.2 | + | 744782190 | N-acetylmuramoyl-L-alanine amidase Sle1 | S. hyicus | 0E+00 |
| 2133 | 2190211 | 2190483 | 91 | 37.2 | - | 744782189 | hypothetical protein SHYC_11440 | S. hyicus | 7E-56 |
| 2134 | 2190710 | 2191216 | 169 | 103.2 | + | 744782188 | putative immunodominant staphylococcal antigen B | S. hyicus | 3E-104 |
| 2135 | 2191322 | 2193580 | 753 | 285.3 | - | 744782187 | excinuclease ABC subunit A related protein | S. hyicus | 0E+00 |
| 2136 | 2193621 | 2194403 | 261 | 90.1 | - | 744782186 | YibE/YibF family protein | S. hyicus | 9E-164 |
| 2137 | 2194400 | 2195512 | 371 | 134.7 | - | 744782185 | YibE/YibF family protein | S. hyicus | 0E+00 |
| 2138 | 2195525 | 2196427 | 301 | 116.1 | - | 744782184 | transcriptional regulatory protein GltC | S. hyicus | 0E+00 |
| 2139 | 2196526 | 2201028 | 1501 | 822.6 | + | 744782183 | glutamate synthase large subunit | S. hyicus | 0E+00 |
| 2140 | 2201042 | 2202499 | 486 | 269.4 | + | 744782182 | glutamate synthase subunit beta | S. hyicus | 0E+00 |
| 2141 | 2203013 | 2204449 | 479 | 256.9 | + | 744782181 | PTS system trehalose-specific transporter subunit IIBC | S. hyicus | 0E+00 |
| 2142 | 2204468 | 2206108 | 547 | 226.6 | + | 744782180 | trehalose-6-phosphate hydrolase | S. hyicus | 0E+00 |
| 2143 | 2206124 | 2206849 | 242 | 122.3 | + | 744782179 | trehalose operon transcriptional repressor | S. hyicus | 2E-170 |
| 2144 | 2207372 | 2207896 | 175 | 96.4 | + | 744782178 | acetyltransferase | S. hyicus | 1E-96 |
| 2145 | 2207962 | 2209644 | 561 | 299.4 | + | 744782177 | DNA polymerase III subunit gamma/tau | S. hyicus | 0E+00 |
| 2146 | 2209742 | 2210062 | 107 | 99.3 | + | 744782176 | YbaB/EbfC family DNA-binding protein | S. hyicus | 2E-68 |
| 2147 | 2210064 | 2210660 | 199 | 93.5 | + | 744782175 | recombination protein RecR | S. hyicus | 9E-143 |
| 2148 | 2216267 | 2216458 | 64 | 31.3 | - | 744782174 | hypothetical protein SHYC_11345 | S. hyicus | 6E-39 |
| 2149 | 2216598 | 2217938 | 447 | 180.5 | + | 744782173 | Orn Lys Arg decarboxylase family protein | S. hyicus | 0E+00 |
| 2150 | 2217940 | 2218554 | 205 | 143.8 | + | 744782172 | thymidylate kinase | S. hyicus | 1E-126 |
| 2151 | 2218702 | 2219031 | 110 | 52.9 | + | 744782171 | hypothetical protein SHYC_11330 | S. hyicus | 6E-74 |
| 2152 | 2219176 | 2220105 | 310 | 146.1 | + | 744782170 | DNA polymerase III subunit delta' | S. hyicus | 0E+00 |
| 2153 | 2220111 | 2220914 | 268 | 147.7 | + | 744782169 | signal peptidase II-like protein | S. hyicus | 2E-180 |
| 2154 | 2220927 | 2221283 | 119 | 60 | + | 744782168 | DNA replication protein YabA | S. hyicus | 2E-79 |
| 2155 | 2221326 | 2222051 | 242 | 95.4 | + | 744782167 | putative O-methyltransferase | S. hyicus | 7E-168 |
| 2156 | 2222044 | 2222298 | 85 | 45.2 | + | 744782166 | GIY-YIG nuclease family protein | S. hyicus | 1E-53 |
| 2157 | 2222295 | 2223131 | 279 | 130 | + | 744782165 | ribosomal RNA small subunit methyltransferase I | S. hyicus | 7E-180 |
| 2158 | 2223229 | 2225196 | 656 | 417 | + | 744782164 | methionyl-tRNA synthetase | S. hyicus | 0E+00 |
| 2159 | 2225360 | 2226130 | 257 | 161.7 | + | 744782163 | TatD family hydrolase | S. hyicus | 0E+00 |
| 2160 | 2226145 | 2226684 | 180 | 108.1 | + | 744782162 | ribonuclease M5 | S. hyicus | 3E-112 |
| 2161 | 2226691 | 2227581 | 297 | 157.7 | + | 744782161 | ribosomal RNA small subunit methyltransferase A | S. hyicus | 0E+00 |
| 2162 | 2227681 | 2227947 | 89 | 48.5 | + | 744782160 | Veg-related protein | S. hyicus | 6E-59 |
| 2163 | 2228207 | 2229055 | 283 | 140.5 | + | 744782159 | 4-diphosphocytidyl-2C-methyl-D-erythritol kinase | S. hyicus | 0E+00 |
| 2164 | 2229079 | 2229906 | 276 | 132.7 | + | 744782158 | purine operon repressor | S. hyicus | 0E+00 |
| 2165 | 2229925 | 2230305 | 127 | 80.6 | + | 744782157 | enamine/imine deaminase | S. hyicus | 3E-87 |
| 2166 | 2230370 | 2230684 | 105 | 53.5 | + | 744782156 | regulatory protein SpoVG | S. hyicus | 6E-69 |
| 2167 | 2231050 | 2232414 | 455 | 271.5 | + | 744782155 | bifunctional N-acetylglucosamine-1-phosphate uridyltransferase/glucosamine-1-phosphate acetyltransferase | S. hyicus | 0E+00 |
| 2168 | 2232484 | 2233449 | 322 | 210.1 | + | 744782154 | ribose-phosphate pyrophosphokinase | S. hyicus | 0E+00 |
| 2169 | 2233588 | 2234262 | 225 | 165.8 | + | 744782153 | 50S ribosomal protein L25 | S. hyicus | 3E-149 |
| 2170 | 2234643 | 2235212 | 190 | 113.8 | + | 744782152 | peptidyl-tRNA hydrolase | S. hyicus | 1E-129 |
| 2171 | 2235209 | 2238721 | 1171 | 647.8 | + | 744782151 | transcription-repair coupling factor | S. hyicus | 0E+00 |
| 2172 | 2238711 | 2240240 | 510 | 154.2 | + | 744782150 | MATE family protein | S. hyicus | 0E+00 |
| 2173 | 2240237 | 2241424 | 396 | 232.7 | + | 744782149 | tetrapyrrole methyltransferase/nucleoside triphosphate pyrophosphohydrolase | S. hyicus | 0E+00 |
| 2174 | 2241411 | 2241668 | 86 | 57.3 | + | 744782148 | RNA-binding S4 domain-containing protein | S. hyicus | 1E-51 |
| 2175 | 2241693 | 2242079 | 129 | 75.8 | + | 744782147 | cell division protein DivIC | S. hyicus | 1E-83 |
| 2176 | 2242283 | 2242660 | 126 | 74.3 | + | 744782146 | hypothetical protein SHYC_11205 | S. hyicus | 1E-83 |
| 2177 | 2242733 | 2244013 | 427 | 145.5 | + | 744782145 | tRNA(Ile)-lysidine synthase | S. hyicus | 0E+00 |
| 2178 | 2244003 | 2244542 | 180 | 121.3 | + | 744782144 | hypoxanthine phosphoribosyltransferase | S. hyicus | 2E-124 |
| 2179 | 2244667 | 2246781 | 705 | 491.7 | + | 744782143 | ATP-dependent zinc metalloprotease FtsH | S. hyicus | 0E+00 |
| 2180 | 2246927 | 2247817 | 297 | 186.7 | + | 744782142 | heat shock protein Hsp33 | S. hyicus | 0E+00 |
| 2181 | 2247984 | 2248913 | 310 | 246.3 | + | 744782141 | cysteine synthase | S. hyicus | 0E+00 |
| 2182 | 2249309 | 2250100 | 264 | 177 | + | 744782140 | dihydropteroate synthase | S. hyicus | 0E+00 |
| 2183 | 2250090 | 2250461 | 124 | 60.8 | + | 744782139 | dihydroneopterin aldolase | S. hyicus | 4E-82 |
| 2184 | 2250454 | 2250933 | 160 | 84.4 | + | 744782138 | 2-amino-4-hydroxy-6- hydroxymethyldihydropteridine pyrophosphokinase | S. hyicus | 4E-103 |
| 2185 | 2251019 | 2252014 | 332 | 221.9 | + | 744782137 | tRNA-dihydrouridine synthase | S. hyicus | 0E+00 |
| 2186 | 2252034 | 2253521 | 496 | 335.2 | + | 744782136 | lysyl-tRNA synthetase | S. hyicus | 0E+00 |
| 2187 | 2260247 | 2262190 | 648 | 279.4 | + | 744782135 | membrane protein | S. hyicus | 0E+00 |
| 2188 | 2262273 | 2263688 | 472 | 170.6 | - | 744782134 | GntR family transcriptional regulator | S. hyicus | 0E+00 |
| 2189 | 2263798 | 2264685 | 296 | 211 | + | 744782133 | pyridoxal biosynthesis lyase PdxS | S. hyicus | 0E+00 |
| 2190 | 2264687 | 2265247 | 187 | 99.3 | + | 744782132 | glutamine amidotransferase subunit PdxT | S. hyicus | 2E-125 |
| 2191 | 2265442 | 2266647 | 402 | 223 | - | 744782131 | pyrimidine nucleoside transporter NupC | S. hyicus | 0E+00 |
| 2192 | 2266812 | 2267273 | 154 | 81.9 | + | 744782130 | transcriptional regulator CtsR | S. hyicus | 3E-108 |
| 2193 | 2267285 | 2267803 | 173 | 88.6 | + | 744782129 | hypothetical protein SHYC_11035 | S. hyicus | 8E-109 |
| 2194 | 2267796 | 2268800 | 335 | 173.5 | + | 746621292 | ATP:guanido phosphotransferase | S. hyicus | 0E+00 |
| 2195 | 2268813 | 2271275 | 821 | 596.1 | + | 744782127 | ATP-dependent Clp protease ATP-binding subunit ClpC | S. hyicus | 0E+00 |
| 2196 | 2271477 | 2272847 | 457 | 226.7 | + | 744782126 | DNA repair protein RadA | S. hyicus | 0E+00 |
| 2197 | 2272862 | 2273923 | 354 | 179.3 | + | 744782125 | PIN domain-containing membrane protein | S. hyicus | 0E+00 |
| 2198 | 2273926 | 2274621 | 232 | 133.4 | + | 744782124 | 2-C-methyl-D-erythritol 4-phosphate cytidylyltransferase | S. hyicus | 7E-170 |
| 2199 | 2274614 | 2275090 | 159 | 109.1 | + | 744782123 | 2-C-methyl-D-erythritol 2,4-cyclodiphosphate synthase | S. hyicus | 2E-109 |
| 2200 | 2275179 | 2276633 | 485 | 327.6 | + | 744782122 | glutamyl-tRNA synthetase | S. hyicus | 0E+00 |
| 2201 | 2276948 | 2277589 | 214 | 86.4 | + | 744782121 | serine acetyltransferase | S. hyicus | 3E-155 |
| 2202 | 2277573 | 2278973 | 467 | 267.9 | + | 744782120 | cysteinyl-tRNA synthetase | S. hyicus | 0E+00 |
| 2203 | 2278966 | 2279367 | 134 | 58.2 | + | 744782119 | mini-ribonuclease 3 | S. hyicus | 8E-92 |
| 2204 | 2279373 | 2280125 | 251 | 144.5 | + | 744782118 | TrmH family RNA methyltransferase | S. hyicus | 0E+00 |
| 2205 | 2280125 | 2280652 | 176 | 71.9 | + | 744782117 | NYN-YacP family protein | S. hyicus | 2E-125 |
| 2206 | 2280724 | 2281296 | 191 | 48.2 | + | 744782116 | putative RNA polymerase sigma factor | S. hyicus | 1E-132 |
| 2207 | 2281606 | 2281785 | 60 | 29.5 | + | 744782114 | preprotein translocase subunit SecE | S. hyicus | 1E-34 |
| 2208 | 2281804 | 2282352 | 183 | 131.3 | + | 744782113 | transcription termination/antitermination factor NusG | S. hyicus | 2E-129 |
| 2209 | 2282684 | 2283106 | 141 | 96 | + | 744782112 | 50S ribosomal protein L11 | S. hyicus | 2E-95 |
| 2210 | 2283319 | 2284017 | 233 | 158.8 | + | 744782111 | 50S ribosomal protein L1 | S. hyicus | 3E-167 |
| 2211 | 2284311 | 2284811 | 167 | 120.5 | + | 744782110 | 50S ribosomal protein L10 | S. hyicus | 8E-116 |
| 2212 | 2284865 | 2285230 | 122 | 113.6 | + | 744782109 | 50S ribosomal protein L7/L12 | S. hyicus | 5E-75 |
| 2213 | 2285309 | 2285917 | 203 | 76.7 | + | 744782108 | RNA methyltransferase | S. hyicus | 1E-139 |
| 2214 | 2286129 | 2289680 | 1184 | 707.7 | + | 744782107 | DNA-directed RNA polymerase subunit beta | S. hyicus | 0E+00 |
| 2215 | 2289855 | 2293472 | 1206 | 751.8 | + | 744782106 | DNA-directed RNA polymerase subunit beta' | S. hyicus | 0E+00 |
| 2216 | 2293617 | 2293871 | 85 | 32.5 | + | 744782105 | ribosomal protein L7Ae | S. hyicus | 4E-51 |
| 2217 | 2293970 | 2294383 | 138 | 71.3 | + | 744782104 | 30S ribosomal protein S12 | S. hyicus | 1E-95 |
| 2218 | 2294465 | 2294935 | 157 | 105.4 | + | 744782103 | 30S ribosomal protein S7 | S. hyicus | 1E-111 |
| 2219 | 2295079 | 2297160 | 694 | 429.8 | + | 744782102 | translation elongation factor G | S. hyicus | 0E+00 |
| 2220 | 2297352 | 2298539 | 396 | 256.9 | + | 744782101 | translation elongation factor Tu | S. hyicus | 0E+00 |
| 2221 | 2298937 | 2300100 | 388 | 197.6 | - | 744782100 | putative hydrolase | S. hyicus | 0E+00 |
| 2222 | 2300209 | 2301399 | 397 | 255.8 | + | 744782099 | 2-amino-3-ketobutyrate CoA ligase | S. hyicus | 0E+00 |
| 2223 | 2301563 | 2302528 | 322 | 185.5 | + | 744782098 | NAD dependent epimerase/dehydratase family protein | S. hyicus | 0E+00 |
| 2224 | 2302822 | 2303898 | 359 | 224 | + | 744782097 | branched-chain amino acid aminotransferase | S. hyicus | 0E+00 |
| 2225 | 2303949 | 2304416 | 156 | 63.9 | - | Q45584 | Uncharacterized protein YbbK | Bacillus subtilis | 1E-41 |
| 2226 | 2304619 | 2304954 | 112 | 27 | - | 744782095 | membrane protein | S. hyicus | 2E-34 |
| 2227 | 2305202 | 2305444 | 81 | 20 | + | P59291 | Metallothiol transferase FosB | S. epidermidis | 2E-26 |
| 2228 | 2305592 | 2306290 | 233 | 114.9 | + | 744782094 | HAD family hydrolase | S. hyicus | 1E-161 |
| 2229 | 2306320 | 2306985 | 222 | 119.7 | - | 744782093 | deoxyadenosine/deoxycytidine kinase | S. hyicus | 1E-164 |
| 2230 | 2306978 | 2307595 | 206 | 95.1 | - | 744782092 | deoxyguanosine kinase | S. hyicus | 4E-148 |
| 2231 | 2307683 | 2308159 | 159 | 51.1 | + | 744782091 | tRNA-specific adenosine deaminase | S. hyicus | 3E-108 |
| 2232 | 2308298 | 2309167 | 290 | 170.3 | + | 744782090 | HAD family hydrolase | S. hyicus | 0E+00 |
| 2233 | 2309185 | 2309751 | 189 | 95.5 | + | 744782089 | FMN-dependent NADH-azoreductase | S. hyicus | 1E-131 |
| 2234 | 2309859 | 2310737 | 293 | 162.3 | - | 744782088 | GTP cyclohydrolase | S. hyicus | 0E+00 |
| 2235 | 2310816 | 2311493 | 226 | 136.6 | - | 744782087 | deacetylase | S. hyicus | 1E-158 |
| 2236 | 2311506 | 2311865 | 120 | 58.5 | - | 744782086 | hypothetical protein SHYC_10820 | S. hyicus | 2E-76 |
| 2237 | 2311975 | 2312784 | 270 | 129.5 | - | 744782085 | RpiR family transcriptional regulator | S. hyicus | 7E-169 |
| 2238 | 2312911 | 2314437 | 509 | 215.2 | + | 744782084 | PTS transporter subunit IIB | S. hyicus | 0E+00 |
| 2239 | 2314455 | 2315126 | 224 | 159 | + | 744782083 | N-acetylmannosamine-6-phosphate 2-epimerase | S. hyicus | 2E-161 |
| 2240 | 2315132 | 2315860 | 243 | 120.1 | + | 744782082 | glucosamine-6-phosphate deaminase | S. hyicus | 5E-175 |
| 2241 | 2315946 | 2316584 | 213 | 174.2 | + | 744782081 | 3-hexulose-6-phosphate synthase | S. hyicus | 1E-145 |
| 2242 | 2316526 | 2317131 | 202 | 94.1 | + | 744782080 | 6-phospho 3-hexuloisomerase | S. hyicus | 3E-128 |
| 2243 | 2317258 | 2318616 | 453 | 169.4 | + | 744782079 | long chain fatty acid-CoA ligase | S. hyicus | 0E+00 |
| 2244 | 2318609 | 2319748 | 380 | 137.7 | + | 744782078 | acetyl-CoA acetyltransferase | S. hyicus | 0E+00 |
| 2245 | 2319723 | 2320079 | 119 | 32.1 | + | 746621216 | hypothetical protein | S. hyicus | 2E-41 |
| 2246 | 2320076 | 2320336 | 87 | 30.2 | + | 744782077 | hypothetical protein SHYC_10770 | S. hyicus | 2E-57 |
| 2247 | 2320719 | 2321015 | 99 | 49.2 | + |  | No Significant Match |  |  |
| 2248 | 2321329 | 2322216 | 296 | 147.9 | + | 744782076 | lipoprotein | S. hyicus | 0E+00 |
| 2249 | 2322475 | 2323305 | 277 | 181.7 | - | 744782075 | pyridoxal kinase | S. hyicus | 0E+00 |
| 2250 | 2323438 | 2324100 | 221 | 99.9 | + | 744782074 | uracil-DNA glycosylase | S. hyicus | 2E-163 |
| 2251 | 2324097 | 2324444 | 116 | 59.7 | + | 744782073 | hypothetical protein SHYC_10745 | S. hyicus | 9E-71 |
| 2252 | 2324466 | 2324828 | 121 | 63.5 | + | 744782072 | membrane protein | S. hyicus | 2E-76 |
| 2253 | 2324956 | 2326407 | 484 | 188.7 | + | 744782071 | amino acid permease | S. hyicus | 0E+00 |
| 2254 | 2326733 | 2327284 | 184 | 84.7 | + | 744782070 | acetyltransferase | S. hyicus | 1E-132 |
| 2255 | 2327571 | 2328128 | 186 | 28.7 | - | 744782069 | TetR family transcriptional regulator | S. hyicus | 2E-129 |
| 2256 | 2328257 | 2328898 | 214 | 135.9 | + | 744782068 | efflux protein | S. hyicus | 1E-146 |
| 2257 | 2328914 | 2330656 | 581 | 225.9 | + | 744782067 | EmrB/QacA family MFS transporter | S. hyicus | 0E+00 |
| 2258 | 2330823 | 2331680 | 286 | 138 | - | 746621192 | lipoate--protein ligase | S. hyicus | 0E+00 |
| 2259 | 2331680 | 2332669 | 330 | 244.2 | - | 744782065 | phosphotransacetylase | S. hyicus | 0E+00 |
| 2260 | 2333029 | 2333778 | 250 | 136.9 | + | 744782064 | putative heme peroxidase | S. hyicus | 5E-177 |
| 2261 | 2334034 | 2335383 | 450 | 198.8 | + | 744782063 | serine/threonine exchanger SteT | S. hyicus | 0E+00 |
| 2262 | 2335410 | 2336171 | 254 | 104.8 | + | 744782062 | hypothetical protein SHYC_10690 | S. hyicus | 4E-174 |
| 2263 | 2336175 | 2336642 | 156 | 48 | + | 744782061 | membrane protein | S. hyicus | 4E-102 |
| 2264 | 2336865 | 2338406 | 514 | 246.4 | + | 744782060 | sodium-dependent dicarboxylate transporter SdcS | S. hyicus | 0E+00 |
| 2265 | 2338507 | 2338848 | 114 | 45.3 | + | 744782059 | hypothetical protein SHYC_10675 | S. hyicus | 5E-74 |
| 2266 | 2339064 | 2339612 | 183 | 84.9 | + | 744782058 | NAD(P)H-dependent oxidoreductase | S. hyicus | 2E-126 |
| 2267 | 2339759 | 2340241 | 161 | 92.9 | + | 744782057 | acetyltransferase | S. hyicus | 2E-103 |
| 2268 | 2340263 | 2341564 | 434 | 211.6 | + | 744782056 | HD superfamily phosphohydrolase | S. hyicus | 0E+00 |
| 2269 | 2341609 | 2342142 | 178 | 97.4 | + | 744782055 | YwhD family protein | S. hyicus | 3E-113 |
| 2270 | 2342616 | 2343029 | 138 | 62.8 | + | 744782054 | hypothetical protein SHYC_10650 | S. hyicus | 4E-91 |
| 2271 | 2343016 | 2344674 | 553 | 347.1 | + | 744782053 | arginyl-tRNA synthetase | S. hyicus | 0E+00 |
| 2272 | 2344810 | 2345691 | 294 | 167.6 | + | 744782052 | putative iron compound ABC transporter substrate-binding lipoprotein | S. hyicus | 0E+00 |
| 2273 | 2345681 | 2346658 | 326 | 97 | + | 744782051 | putative iron compound ABC transporter permease | S. hyicus | 7E-166 |
| 2274 | 2346821 | 2347525 | 235 | 125.6 | + | 744782050 | HAD family hydrolase | S. hyicus | 2E-156 |
| 2275 | 2347525 | 2348325 | 267 | 134.1 | + | 744782049 | alpha/beta hydrolase | S. hyicus | 0E+00 |
| 2276 | 2348417 | 2348923 | 169 | 105.6 | + | 744782048 | hypothetical protein SHYC_10620 | S. hyicus | 6E-115 |
| 2277 | 2349101 | 2349817 | 239 | 102.4 | + | 744782047 | membrane protein | S. hyicus | 5E-166 |
| 2278 | 2349978 | 2350349 | 124 | 43.4 | - | 744782046 | staphylococcal accessory transcriptional regulator family protein | S. hyicus | 4E-84 |
| 2279 | 2350643 | 2351539 | 299 | 84.8 | - | 744782045 | hypothetical protein SHYC_10605 | S. hyicus | 2E-161 |
| 2280 | 2352062 | 2352988 | 309 | 67.7 | - |  | No Significant Match |  |  |
| 2281 | 2353203 | 2353430 | 76 | 6.1 | + | 744782044 | hypothetical protein SHYC_10600 | S. hyicus | 8E-34 |
| 2282 | 2353683 | 2353898 | 72 | 36.3 | - | 744782043 | hypothetical protein SHYC_10595 | S. hyicus | 7E-37 |
| 2283 | 2353914 | 2354117 | 68 | 34.2 | - | 744782042 | hypothetical protein SHYC_10590 | S. hyicus | 3E-29 |
| 2284 | 2354261 | 2354812 | 184 | 68.8 | + | 744782041 | DNA integration/recombination/inversion protein | S. hyicus | 2E-123 |
| 2285 | 2354836 | 2357220 | 795 | 283.2 | + | 744782040 | monovalent cation/H+ antiporter subunit A | S. hyicus | 0E+00 |
| 2286 | 2357207 | 2357632 | 142 | 61.2 | + | 744782039 | monovalent cation/H+ antiporter subunit B | S. hyicus | 4E-89 |
| 2287 | 2357629 | 2357976 | 116 | 62.8 | + | 744782038 | monovalent cation/H+ antiporter subunit C | S. hyicus | 5E-73 |
| 2288 | 2357966 | 2359468 | 501 | 221.4 | + | 744782037 | monovalent cation/H+ antiporter subunit D | S. hyicus | 0E+00 |
| 2289 | 2359465 | 2359947 | 161 | 69 | + | 744782036 | monovalent cation/H+ antiporter subunit E | S. hyicus | 3E-100 |
| 2290 | 2359944 | 2360246 | 101 | 42.4 | + | 744782035 | monovalent cation/H+ antiporter subunit F | S. hyicus | 1E-60 |
| 2291 | 2360212 | 2360664 | 151 | 63.3 | + | 744782034 | monovalent cation/H+ antiporter subunit G | S. hyicus | 3E-98 |
| 2292 | 2360941 | 2361576 | 212 | 85.6 | + |  | No Significant Match |  |  |
| 2293 | 2361681 | 2362598 | 306 | 169.8 | - | 744782033 | putative manganese ABC transporter substrate-binding lipoprotein | S. hyicus | 0E+00 |
| 2294 | 2362595 | 2363434 | 280 | 88.4 | - | 744782032 | putative manganese ABC transporter permease | S. hyicus | 0E+00 |
| 2295 | 2363409 | 2364188 | 260 | 114.3 | - | 744782031 | ABC transporter ATP-binding protein | S. hyicus | 0E+00 |
| 2296 | 2364309 | 2364959 | 217 | 114.7 | + | 744782030 | DtxR family transcriptional regulator | S. hyicus | 1E-150 |
| 2297 | 2365626 | 2366372 | 249 | 71.2 | - | 744782029 | membrane protein | S. hyicus | 3E-156 |
| 2298 | 2366940 | 2367764 | 275 | 142 | + | 744782028 | UDP-N-acetyl-D-mannosamine transferase | S. hyicus | 0E+00 |
| 2299 | 2367826 | 2368620 | 265 | 113 | - | 744782027 | teichoic acids export protein ATP-binding subunit | S. hyicus | 0E+00 |
| 2300 | 2369004 | 2369834 | 277 | 65.3 | + | 744782026 | teichoic acid translocation permease protein TagG | S. hyicus | 0E+00 |
| 2301 | 2370017 | 2371108 | 364 | 141.2 | + | 744782025 | teichoic acid biosynthesis protein B | S. hyicus | 0E+00 |
| 2302 | 2371238 | 2371636 | 133 | 73.6 | + | 744782024 | glycerol-3-phosphate cytidylyltransferase | S. hyicus | 4E-91 |
| 2303 | 2371676 | 2372956 | 427 | 190.1 | - | 744782023 | D-alanyl-D-alanine carboxypeptidase | S. hyicus | 0E+00 |
| 2304 | 2373174 | 2374898 | 575 | 283.3 | + | 744782022 | ABC transporter ATP-binding and permease protein | S. hyicus | 0E+00 |
| 2305 | 2374961 | 2375122 | 54 | 5.7 | - | 744782021 | hypothetical protein SHYC_10485 | S. hyicus | 2E-26 |
| 2306 | 2375133 | 2377127 | 665 | 308.5 | - | 744782020 | ferrous iron transport protein B | S. hyicus | 0E+00 |
| 2307 | 2377120 | 2377356 | 79 | 24.1 | - | 744782019 | FeoA domain protein | S. hyicus | 8E-46 |
| 2308 | 2377624 | 2378850 | 409 | 201.2 | + | 744782018 | pyrimidine nucleoside transport protein | S. hyicus | 0E+00 |
| 2309 | 2379342 | 2380196 | 285 | 147.9 | + | 744782017 | membrane protein | S. hyicus | 0E+00 |
| 2310 | 2380396 | 2381202 | 269 | 152.5 | + | 744782016 | ferrichrome transport ATP-binding protein FhuC | S. hyicus | 0E+00 |
| 2311 | 2381224 | 2382225 | 334 | 154.4 | + | 744782015 | ferrichrome transport system permease FhuB | S. hyicus | 0E+00 |
| 2312 | 2382222 | 2383238 | 339 | 138.4 | + | 744782014 | ferrichrome transport system permease FhuG | S. hyicus | 0E+00 |
| 2313 | 2383379 | 2383879 | 167 | 92.8 | + | 744782013 | hypothetical protein SHYC_10445 | S. hyicus | 2E-115 |
| 2314 | 2384260 | 2385327 | 356 | 178.2 | + | 744782010 | membrane protein | S. hyicus | 0E+00 |
| 2315 | 2385370 | 2386407 | 346 | 148.4 | + | 744782009 | lipase | S. hyicus | 0E+00 |
| 2316 | 2386596 | 2387102 | 169 | 67.7 | - | 744782008 | GNAT family acetyltransferase | S. hyicus | 2E-116 |
| 2317 | 2387215 | 2388147 | 311 | 100.9 | + | 744782007 | hypothetical protein SHYC_10420 | S. hyicus | 0E+00 |
| 2318 | 2388904 | 2389521 | 206 | 110.5 | + | 744782006 | putative phosphate transport regulator | S. hyicus | 3E-149 |
| 2319 | 2389536 | 2390537 | 334 | 147.6 | + | 744782005 | inorganic phosphate transporter | S. hyicus | 0E+00 |
| 2320 | 2390826 | 2391647 | 274 | 132.6 | - | 744782002 | hypothetical protein SHYC_10385 | S. hyicus | 0E+00 |
| 2321 | 2391925 | 2392563 | 213 | 52.5 | + | 744782001 | membrane protein | S. hyicus | 6E-110 |
| 2322 | 2392750 | 2393334 | 195 | 58.7 | + | 744782000 | thiamine monophosphate synthase | S. hyicus | 7E-77 |
| 2323 | 2393331 | 2393531 | 67 | 33.5 | + | 744781999 | sulfur carrier protein ThiS | S. hyicus | 3E-24 |
| 2324 | 2393533 | 2394300 | 256 | 149.6 | + | 744781998 | thiazole synthase | S. hyicus | 6E-166 |
| 2325 | 2394300 | 2395292 | 331 | 104.5 | + | 744781997 | HesA/MoeB/ThiF family protein | S. hyicus | 0E+00 |
| 2326 | 2395372 | 2396085 | 238 | 155.9 | + | 744781996 | YebC/PmpR family DNA-binding regulatory protein | S. hyicus | 1E-167 |
| 2327 | 2396387 | 2396950 | 188 | 63.5 | + | 744781995 | putative RNA-binding protein | S. hyicus | 3E-129 |
| 2328 | 2397054 | 2397923 | 290 | 136.2 | + | 744781994 | LysR family transcriptional regulator | S. hyicus | 0E+00 |
| 2329 | 2397937 | 2399139 | 401 | 168.6 | + | 744781993 | MFS sugar transporter | S. hyicus | 0E+00 |
| 2330 | 2399157 | 2399648 | 164 | 36.7 | + | 744781992 | membrane protein | S. hyicus | 1E-90 |
| 2331 | 2399800 | 2400507 | 236 | 80.2 | - | 744781991 | hypothetical protein SHYC_10330 | S. hyicus | 4E-151 |
| 2332 | 2400594 | 2401064 | 157 | 56.2 | + | 744781990 | putative acetyltransferase | S. hyicus | 2E-61 |
| 2333 | 2401130 | 2401513 | 128 | 72 | + | 744781989 | hypothetical protein SHYC_10320 | S. hyicus | 3E-37 |
| 2334 | 2401613 | 2401912 | 100 | 70.6 | + | 744781988 | hypothetical protein SHYC_10315 | S. hyicus | 1E-62 |
| 2335 | 2401989 | 2402540 | 184 | 102.8 | + | 744781987 | GNAT family acetyltransferase | S. hyicus | 5E-116 |
| 2336 | 2402622 | 2403191 | 190 | 111.2 | - | 744781986 | decarboxylase family protein | S. hyicus | 1E-117 |
| 2337 | 2403184 | 2403654 | 157 | 45.6 | - | 744781985 | hypothetical protein SHYC_10300 | S. hyicus | 4E-88 |
| 2338 | 2403656 | 2404942 | 429 | 253.5 | - | 744781984 | hypothetical protein SHYC_10295 | S. hyicus | 0E+00 |
| 2339 | 2405148 | 2405504 | 119 | 55.1 | - | 744781983 | hypothetical protein SHYC_10290 | S. hyicus | 3E-78 |
| 2340 | 2405536 | 2406183 | 216 | 39.8 | - | 744781982 | CAAX amino terminal protease family protein | S. hyicus | 8E-111 |
| 2341 | 2406590 | 2407444 | 285 | 147 | - | 744781981 | undecaprenyl pyrophosphate phosphatase | S. hyicus | 0E+00 |
| 2342 | 2407738 | 2409297 | 520 | 248.6 | + | 744781980 | ABC transporter ATP-binding protein | S. hyicus | 0E+00 |
| 2343 | 2409369 | 2411018 | 550 | 249 | + | 744781979 | ABC transporter ATP-binding protein | S. hyicus | 0E+00 |
| 2344 | 2411228 | 2411665 | 146 | 71.9 | - | 744781978 | HTH-type transcriptional regulator MgrA | S. hyicus | 4E-102 |
| 2345 | 2411887 | 2412807 | 307 | 114.1 | + | 744781977 | hypothetical protein SHYC_10260 | S. hyicus | 0E+00 |
| 2346 | 2412831 | 2413739 | 303 | 136.4 | + | 744781976 | oxidoreductase, aldo/keto reductase family | S. hyicus | 0E+00 |
| 2347 | 2413762 | 2414046 | 95 | 31.3 | + | 744781975 | hypothetical protein SHYC_10250 | S. hyicus | 6E-51 |
| 2348 | 2414198 | 2415142 | 315 | 175.5 | + | 744781974 | malate dehydrogenase | S. hyicus | 0E+00 |
| 2349 | 2415279 | 2415998 | 240 | 59.8 | - | 744781973 | metal-dependent membrane protease | S. hyicus | 8E-117 |
| 2350 | 2416028 | 2416291 | 88 | 0.8 | - | 744781972 | hypothetical protein SHYC_10235 | S. hyicus | 2E-24 |
| 2351 | 2416288 | 2416905 | 206 | 57.5 | - | 744781971 | membrane protein | S. hyicus | 4E-127 |
| 2352 | 2417114 | 2418271 | 386 | 176.4 | + | 744781970 | MFS transporter | S. hyicus | 0E+00 |
| 2353 | 2418334 | 2420661 | 776 | 311.4 | + | 744781969 | CDP-glycerol:glycerophosphate glycerophosphotransferase | S. hyicus | 0E+00 |
| 2354 | 2420675 | 2421154 | 160 | 61.9 | + | 744781968 | Cys-tRNA(Pro)/Cys-tRNA(Cys) deacylase YbaK | S. hyicus | 3E-80 |
| 2355 | 2421309 | 2422064 | 252 | 142.6 | + | 744781967 | DeoR faimly transcriptional regulator | S. hyicus | 4E-169 |
| 2356 | 2422061 | 2422984 | 308 | 166.3 | + | 744781966 | 1-phosphofructokinase | S. hyicus | 0E+00 |
| 2357 | 2422986 | 2424899 | 638 | 414.9 | + | 744781965 | PTS system fructose-specific IIABC component | S. hyicus | 0E+00 |
| 2358 | 2425307 | 2426668 | 454 | 244 | + | 744781964 | CBS domains-containing protein | S. hyicus | 0E+00 |
| 2359 | 2426949 | 2427914 | 322 | 145.5 | + | 744781963 | glycosyltransferase/CsbB stress response protein | S. hyicus | 0E+00 |
| 2360 | 2428075 | 2428221 | 49 | 0.3 | + |  | No Significant Match |  |  |
| 2361 | 2428346 | 2428603 | 86 | 68.5 | - | 744781962 | membrane protein | S. hyicus | 3E-45 |
| 2362 | 2428680 | 2429267 | 196 | 44.2 | - | 744781961 | hypothetical protein SHYC_10180 | S. hyicus | 4E-114 |
| 2363 | 2429367 | 2430080 | 238 | 112.3 | - | 744781960 | 7-carboxy-7-deazaguanine synthase | S. hyicus | 5E-168 |
| 2364 | 2430073 | 2430504 | 144 | 55.9 | - | 744781959 | 6-carboxy-5,6,7,8-tetrahydropterin synthase | S. hyicus | 4E-99 |
| 2365 | 2430504 | 2431172 | 223 | 103.3 | - | 744781958 | 7-cyano-7-deazaguanine synthase | S. hyicus | 2E-153 |
| 2366 | 2431629 | 2432222 | 198 | 66.6 | + | 744781957 | para-aminobenzoate synthase subunit II | S. hyicus | 5E-120 |
| 2367 | 2432206 | 2433339 | 378 | 155.8 | + | 744781956 | para-aminobenzoate synthase component I | S. hyicus | 0E+00 |
| 2368 | 2433343 | 2433951 | 203 | 79.9 | + | 744781955 | aminodeoxychorismate lyase | S. hyicus | 2E-94 |
| 2369 | 2434185 | 2434868 | 228 | 123.8 | + | 744781953 | allophanate hydrolase subunit 1 | S. hyicus | 2E-158 |
| 2370 | 2434865 | 2435869 | 335 | 188.4 | + | 744781952 | allophanate hydrolase subunit 2 | S. hyicus | 0E+00 |
| 2371 | 2436391 | 2438328 | 646 | 324 | + | 744781951 | glycerol phosphate lipoteichoic acid synthase | S. hyicus | 0E+00 |
| 2372 | 2438401 | 2439216 | 272 | 120.7 | - | 744781950 | ZIP family metal transporter | S. hyicus | 0E+00 |
| 2373 | 2439329 | 2441206 | 626 | 332.3 | + | 744781949 | ABC transporter ATP-binding protein | S. hyicus | 0E+00 |
| 2374 | 2441217 | 2443007 | 597 | 260.8 | + | 744781948 | ATP-dependent DNA helicase RecQ-2 | S. hyicus | 0E+00 |
| 2375 | 2443185 | 2444138 | 318 | 140.8 | + | 744781947 | glycine betaine/carnitine/choline transport ATP-binding protein | S. hyicus | 0E+00 |
| 2376 | 2444135 | 2445649 | 505 | 287.8 | + | 744781946 | glycine betaine/carnitine/choline transport system permease protein | S. hyicus | 0E+00 |
| 2377 | 2445955 | 2447004 | 350 | 200.5 | + | 744781945 | histidinol-phosphate aminotransferase | S. hyicus | 0E+00 |
| 2378 | 2447079 | 2447609 | 177 | 99.9 | + | 746621942 | 5'-3'-deoxyribonucleotidase | S. hyicus | 9E-124 |
| 2379 | 2447670 | 2448599 | 310 | 135 | - | 744781943 | lipid kinase | S. hyicus | 0E+00 |
| 2380 | 2448919 | 2450430 | 504 | 218.3 | - | 744781942 | dipeptide/tripeptide permease | S. hyicus | 0E+00 |
| 2381 | 2450842 | 2451342 | 167 | 73.7 | - | 744781941 | 7-cyano-7-deazaguanine reductase | S. hyicus | 2E-120 |
| 2382 | 2451356 | 2452240 | 295 | 117 | - | 744781940 | putative permease | S. hyicus | 0E+00 |
| 2383 | 2452951 | 2453340 | 130 | 51.7 | + | 744781939 | ribonucleotide reductase stimulatory protein | S. hyicus | 3E-88 |
| 2384 | 2453312 | 2455417 | 702 | 384.1 | + | 744781938 | ribonucleotide-diphosphate reductase subunit alpha | S. hyicus | 0E+00 |
| 2385 | 2455632 | 2456603 | 324 | 190.1 | + | 744781937 | ribonucleotide-diphosphate reductase subunit beta | S. hyicus | 0E+00 |
| 2386 | 2456660 | 2456974 | 105 | 20.8 | - | 744781936 | zinc finger CHY domain-containing protein | S. hyicus | 3E-70 |
| 2387 | 2456988 | 2457914 | 309 | 183.6 | - | 744781935 | UDP-N-acetylenolpyruvoylglucosamine reductase | S. hyicus | 0E+00 |
| 2388 | 2457987 | 2458508 | 174 | 59.6 | - | 744781934 | hypothetical protein SHYC_10045 | S. hyicus | 6E-102 |
| 2389 | 2458607 | 2459503 | 299 | 173.2 | + | 744781933 | hypothetical protein SHYC_10040 | S. hyicus | 6E-107 |
| 2390 | 2459644 | 2459961 | 106 | 48.4 | + | 744781932 | hypothetical protein SHYC_10035 | S. hyicus | 6E-72 |
| 2391 | 2460081 | 2461193 | 371 | 198.8 | + | 744781931 | glycerate kinase | S. hyicus | 0E+00 |
| 2392 | 2461246 | 2462472 | 409 | 216.6 | - | 744781930 | peptidase T | S. hyicus | 0E+00 |
| 2393 | 2462485 | 2462988 | 168 | 53.8 | - | 744781929 | membrane protein | S. hyicus | 3E-116 |
| 2394 | 2463004 | 2463765 | 254 | 129.3 | - | 744781928 | membrane protein | S. hyicus | 1E-178 |
| 2395 | 2464139 | 2465209 | 357 | 159.8 | - | 744781927 | diguanylate cyclase (GGDEF) domain protein | S. hyicus | 0E+00 |
| 2396 | 2465573 | 2465893 | 107 | 27.1 | - | 744781926 | staphostatin A family protein | S. hyicus | 1E-37 |
| 2397 | 2465924 | 2467090 | 389 | 176.1 | - | 744781925 | staphopain A cysteine protease | S. hyicus | 0E+00 |
| 2398 | 2467796 | 2468842 | 349 | 123.1 | + | 746620942 | UDP-phosphate N-acetylglucosaminyl 1-phosphate transferase | S. hyicus | 0E+00 |
| 2399 | 2468889 | 2469527 | 213 | 100.7 | - | 746620940 | ABC transporter | S. hyicus | 2E-142 |
| 2400 | 2469844 | 2470707 | 288 | 152.3 | + | 746620939 | DegV family EDD domain-containing protein | S. hyicus | 0E+00 |
| 2401 | 2470850 | 2472136 | 429 | 128.5 | + | 746620936 | competence protein ComF | S. hyicus | 0E+00 |
| 2402 | 2472136 | 2472795 | 220 | 34.3 | + | 746620934 | Predicted amidophosphoribosyltransferases | S. hyicus | 2E-111 |
| 2403 | 2472858 | 2473427 | 190 | 109.9 | + | 739714069 | sigma-54 modulation protein | S. hyicus | 1E-133 |
| 2404 | 2473954 | 2474433 | 160 | 117.9 | + | 746620931 | preprotein translocase subunit SecA | S. hyicus | 3E-101 |
