# Supplementary material for: Genome Analysis of Staphylococcus agnetis, an Agent of Lameness in Broiler Chickens
Source: PLoS One. 2015 Nov 25;10(11):e0143336. doi: 10.1371/journal.pone.0143336 (PMC4659636; doi:10.1371/journal.pone.0143336)
Supplement: S2 Table — ORF is the Open Reading Frame as indicated in S1 Table. For each ORF are indicated the most significant match in VFDB the Virulence Category, Gene Index (GI), Description, and Top Hit Species (and Strain), E-value of the match in VFDB, Description of best match for species that is NOT S. hyicus in Tremble database, with description and E-value. (DOCX) [file pone.0143336.s002.docx]

| **ORF** | **Category** | **GI** | **VFDB Description** | **VFDB Top Hit Species** | **VFDB E-Value** | **Tremble Description** | **Trembl Top Hit Species (Non-hyicus)** | **Trembl Top Hit Evalue** |
| --- | --- | --- | --- | --- | --- | --- | --- | --- |
| 154 | Adherence | 15928081 | fnbB(SA2290)-hypotheticalprotein | S. aureus str.N315 | 8.00E-151 | S2YL33_9STAP YSIRK family gram-positive signal peptide | Staphylococcus sp. HGB0015 | 5.17E-179 |
| 263 | Adherence | 161509237 | atl(USA300HOU_0997)-bifunctionalN-acetylmuramoyl-L-alanineamidase/mannosyl-glycoproteinendo-beta-N-acetylglucosaminidase | S. aureus str.USA300_TCH1516 | 0.00E+00 | F0P7U5_STAPE Autolysin | S. pseudintermedius (strain ED99) | 0.00E+00 |
| 660 | Adherence | 148267964 | ebp(SaurJH9_1537)-Peptidoglycan-bindingLysM | S. aureus str.JH9 | 1.00E-43 | A0A012DHF2_STAAU Elastin-binding protein ebpS | S. aureus DAR5816 | 1.19E-39 |
| 675 | Adherence | 49487471 | cna(SAS2578)-collagenadhesinprecursor | S. aureus str.MSSA476 | 0.00E+00 | H3YKL3_STAAU Collagen adhesin | S. aureus subsp. aureus IS-105 | 0.00E+00 |
| 933 | Adherence | 73663050 | atl(SSP1741)-bifunctionalautolysinprecursor | S. saprophyticus str.ATCC15305 | 2.00E-62 | S2Y1B9_9STAP Uncharacterized protein | Staphylococcus sp. HGB0015 | 0.00E+00 |
| 1044 | Adherence | 15928081 | fnbB(SA2290)-hypotheticalprotein | S. aureus str.N315 | 5.00E-112 | A0A035U501_STAAU Uncharacterized protein | S. aureus VET0256R | 5.78E-117 |
| 1046 | Adherence | 49487279 | fnbB(SAS2387)-fibronectin-bindingproteinprecursor | S. aureus str.MSSA476 | 1.00E-49 | S2YL33_9STAP YSIRK family gram-positive signal peptide | Staphylococcus sp. HGB0015 | 4.30E-52 |
| 1047 | Adherence | 21284149 | fnbB(MW2420)-hypotheticalprotein | S. aureus str.MW2 | 3.00E-55 | A0A034H8Y5_STAAU Uncharacterized protein (Fragment) | S. aureus R0611 | 1.21E-63 |
| 1048 | Adherence | 49484704 | fnbA(SAR2580)-fibronectin-bindingproteinprecursor | S. aureus str.MRSA252 | 1.00E-125 | H4G1G0_STAAU Fibronectin-binding protein A (Fragment) | S. aureus subsp. aureus IS-160 | 1.76E-127 |
| 1413 | Adherence | 27467668 | atl(SE0750)-N-acetylmuramoyl-L-alanineamidase | S. epidermidis str.ATCC12228 | 2.00E-51 | S2Y8G2_9STAP Uncharacterized protein | Staphylococcus sp. HGB0015 | 2.05E-113 |
| 1909 | Adherence | 161508803 | sdrE(USA300HOU_0557)-Ser-Asprichfibrinogen/bonesialoprotein-bindingproteinSdrE | S. aureus str.USA300_TCH1516 | 2.00E-139 | K8NQI5_STASI YSIRK family Gram-positive signal peptide (Fragment) | S. simulans ACS-120-V-Sch1 | 0.00E+00 |
| 529 | ExoEzyme | 27467922 | nuc(SE1004)-thermonuclease | S. epidermidis str.ATCC12228 | 2.00E-62 | C0STM5_STAHY Thermonuclease | S. hyicus | 2.07E-118 |
| 1620 | ExoEzyme | 57865740 | lip(SERP2297)-lipase | S. epidermidis str.RP62A | 4.00E-161 | J1ADA0_STAEP Triacylglycerol lipase | S. epidermidis NIHLM023 | 4.75E-159 |
| 2075 | ExoEzyme | 15927782 | hysA(SA2003)-hyaluronatelyaseprecursor | S. aureus str.N315 | 0.00E+00 | Z6PYA2_STAAU Hyaluronate lyase | S. aureus H87668 | 0.00E+00 |
| 2397 | ExoEzyme | 57865777 | sspB(SERP2390)-cysteineproteaseprecursorSspB | S. epidermidis str.RP62A | 7.00E-169 | H0AQR0_STAAU Staphopain A | S. aureus subsp. aureus 21202 | 0.00E+00 |
| 268 | Host Immune Invasion | 49482406 | capO(SAR0165)-capsularpolysaccharidesynthesisenzyme | S. aureus str.MRSA252 | 0.00E+00 | F0P7T9_STAPE UDP-N-acetyl-D-mannosamine dehydrogenase | S. pseudintermedius (strain ED99) | 0.00E+00 |
| 1245 | Host Immune Invasion | 49482407 | capP(SAR0166)-capsularpolysaccharidesynthesisenzyme | S. aureus str.MRSA252 | 2.00E-174 | F0P4C5_STAPE UDP-N-acetylglucosamine 2-epimerase | S. pseudintermedius (strain ED99) | 0.00E+00 |
| 1418 | Host Immune Invasion | 70725401 | capM(SH0400)-hypotheticalprotein | S. haemolyticus str.JCSC1435 | 6.00E-96 | F0P6F8_STAPE Transcriptional regulator | S. pseudintermedius (strain ED99) | 1.93E-173 |
| 1559 | Host Immune Invasion | 49485038 | SAS0137-capsularpolysaccharidesynthesisenzyme | S. aureus str.MSSA476 | 1.00E-89 | G7ZTN1_STAAU Capsular polysaccharide synthesis enzyme | S. aureus subsp. aureus MSHR1132 | 2.46E-89 |
| 1560 | Host Immune Invasion | 49482404 | capM(SAR0163)-capsularpolysaccharidesynthesisenzyme | S. aureus str.MRSA252 | 1.00E-104 | A0A019Z5I3_STAAU Capsular polysaccharide biosynthesis protein Cap5M | S. aureus M21126 | 9.51E-101 |
| 1561 | Host Immune Invasion | 49482403 | capL(SAR0162)-capsularpolysaccharidesynthesisenzyme | S. aureus str.MRSA252 | 0.00E+00 | F8KJG9_STALN Capsular polysaccharide synthesis enzyme | S. lugdunensis (strain N920143) | 0.00E+00 |
| 1562 | Host Immune Invasion | 49482402 | cap8K(SAR0161)-capsularpolysaccharidesynthesisenzyme | S. aureus str.MRSA252 | 2.00E-161 | G7ZTM8_STAAU Capsular polysaccharide synthesis enzyme | S. aureus subsp. aureus MSHR1132 | 4.21E-177 |
| 1563 | Host Immune Invasion | 82749871 | cap8J(SAB0099)-capsularpolysaccharidesynthesisenzymeCapJ | S. aureus str.RF122 | 2.00E-97 | Y8KUV0_STALU Uncharacterized protein | S. lugdunensis UCIM6116 | 1.65E-99 |
| 1564 | Host Immune Invasion | 49482400 | cap8I(SAR0159)-capsularpolysaccharidesynthesisenzyme | S. aureus str.MRSA252 | 3.00E-153 | F0D3U3_STAAU Capsular polysaccharide synthesis enzyme CapI | S. aureus O11 | 1.33E-149 |
| 1565 | Host Immune Invasion | 21281860 | cap8H(MW0131)-capsularpolysaccharidesynthesisenzymeCap8H | S. aureus str.MW2 | 2.00E-126 | G7ZTM5_STAAU Capsular polysaccharide synthesis enzyme | S. aureus subsp. aureus MSHR1132 | 3.16E-127 |
| 1566 | Host Immune Invasion | 151220313 | capG(NWMN_0101)-capsularpolysaccharidesynthesisenzymeCapG | S. aureus str.Newman | 0.00E+00 | G7ZTM4_STAAU Capsular polysaccharide synthesis enzyme | S. aureus subsp. aureus MSHR1132 | 0.00E+00 |
| 1567 | Host Immune Invasion | 70725394 | capF(SH0393)-capsularpolysaccharidesynthesisenzymeCapF | S. haemolyticus str.JCSC1435 | 0.00E+00 | Q4L9H3_STAHJ Capsular polysaccharide synthesis enzyme CapF | S. haemolyticus (strain JCSC1435) | 0.00E+00 |
| 1568 | Host Immune Invasion | 70725393 | capE(SH0392)-capsularpolysaccharidesynthesisenzymeCapE | S. haemolyticus str.JCSC1435 | 0.00E+00 | Q4L9H4_STAHJ Capsular polysaccharide synthesis enzyme CapE | S. haemolyticus (strain JCSC1435) | 0.00E+00 |
| 1569 | Host Immune Invasion | 70725392 | capD(SH0391)-capsularpolysaccharidebiosynthesisproteinCapD | S. haemolyticus str.JCSC1435 | 0.00E+00 | E5CJ50_STAHO Capsular polysaccharide biosynthesis protein Cap5D | S. hominis subsp. hominis C80 | 0.00E+00 |
| 1570 | Host Immune Invasion | 70725391 | capC(SH0390)-capsularpolysaccharidesynthesisenzymeCapC | S. haemolyticus str.JCSC1435 | 1.00E-89 | U1RLG0_9STAP Capsular polysaccharide biosynthesis protein Cap8C | S. equorum UMC-CNS-924 | 3.10E-86 |
| 1571 | Host Immune Invasion | 151220308 | capB(NWMN_0096)-capsularpolysaccharidesynthesisenzymeCapB | S. aureus str.Newman | 6.00E-84 | A0A037M2U0_STAAU Uncharacterized protein | S. aureus VET1877R | 8.41E-89 |
| 1572 | Host Immune Invasion | 73661369 | SSP0060-capsularpolysaccharidebiosynthesisprotein | S. saprophyticus str.ATCC15305 | 6.00E-77 | E5CJ47_STAHO Capsular polysaccharide biosynthesis protein Cap1A | S. hominis subsp. hominis C80 | 2.29E-78 |
| 2107 | Secretion System | 161508548 | esxA(USA300HOU_0297)-ESAT-6familyvirulenceprotein | S. aureus str.USA300_TCH1516 | 4.00E-60 | C5QQK4_9STAP WXG100 family type VII secretion target | S. caprae M23864:W1 | 2.23E-57 |
| 2108 | Secretion System | 49482520 | esaA(SAR0280)-hypotheticalprotein | S. aureus str.MRSA252 | 0.00E+00 | C5QQK5_9STAP Type VII secretion protein EsaA | S. caprae M23864:W1 | 0.00E+00 |
| 2109 | Secretion System | 161508550 | essA(USA300HOU_0299)-virulenceproteinEssA | S. aureus str.USA300_TCH1516 | 1.00E-42 | T1Y6U1_STAAU Type VII secretion protein EssA | S. aureus subsp. aureus CN1 | 5.41E-39 |
| 2110 | Secretion System | 49482523 | essB(SAR0283)-hypotheticalprotein | S. aureus str.MRSA252 | 0.00E+00 | D2UJ57_STAAU Virulence protein EssB | S. aureus subsp. aureus H19 | 0.00E+00 |
| 2111 | Secretion System | 49482524 | essC(SAR0284)-hypotheticalprotein | S. aureus str.MRSA252 | 0.00E+00 | H0DWP9_STAEP Type VII secretion protein EssC | S. epidermidis 14.1.R1.SE | 0.00E+00 |
| 1683 | Toxin | 57865922 | hlb(SERP2544)-beta-hemolysin | S. epidermidis str.RP62A | 5.00E-122 | F9L782_STACP Sphingomyelin phosphodiesterase | S. capitis VCU116 | 4.47E-123 |
| 1908 | Toxin | 15926111 | set15(SA0393)-exotoxin15 | S. aureus str.N315 | 2.00E-57 | Q6GJN6_STAAR Exotoxin | S. aureus (strain MRSA252) | 4.62E-55 |
| 1911 | Toxin | 21282111 | set16(MW0382)-hypotheticalprotein | S. aureus str.MW2 | 2.00E-54 | D9RE87_STAAJ Superantigen-like protein | S. aureus (strain JKD6159) | 5.89E-51 |
| 1913 | Toxin | 82750136 | set16(SAB0376)-staphylococcalexotoxin11 | S. aureus str.RF122 | 2.00E-45 | G0LRB3_STAAU Exotoxin | S. aureus subsp. aureus LGA251 | 1.36E-43 |
| 1914 | Toxin | 82750136 | set16(SAB0376)-staphylococcalexotoxin11 | S. aureus str.RF122 | 3.00E-41 | D9RE87_STAAJ Superantigen-like protein | S. aureus (strain JKD6159) | 1.90E-40 |
| 1915 | Toxin | 87160249 | set34(SAUSA300_0399)-exotoxin | S. aureus str.USA300 | 3.00E-46 | F5WAA5_STAAU Superantigen-like protein | S. aureus subsp. aureus 21305 | 1.25E-44 |
| 2066 | Toxin | 151221294 | eta(NWMN_1082)-exfoliativetoxinA | S. aureus str.Newman | 2.00E-163 | Q9FDT4_STAHY Exfoliative toxin A | S. hyicus | 2.04E-179 |
