# Supplementary material for: Genome Analysis of Staphylococcus agnetis, an Agent of Lameness in Broiler Chickens
Source: PLoS One. 2015 Nov 25;10(11):e0143336. doi: 10.1371/journal.pone.0143336 (PMC4659636; doi:10.1371/journal.pone.0143336)
Supplement: S3 Table — Details are as for S1 Table. (DOCX) [file pone.0143336.s003.docx]

| **ORF** | **Start** | **End** | **Len** | **Scr** | **Str** | **GI** | **Top Hit** | **Top Hit Species** | **Hsp_evalue** |
| --- | --- | --- | --- | --- | --- | --- | --- | --- | --- |
| 1 | 466 | 1035 | 190 | 138.3 | - | 702226826 | hypothetical protein | S. intermedius | 2E-33 |
| 2 | 2339 | 2905 | 189 | 111.1 | - | 686383629 | replication protein | S. aureus | 3E-106 |
| 3 | 3094 | 3561 | 156 | 115.1 | + | 686340339 | integrase | S. aureus | 3E-91 |
| 4 | 3880 | 4056 | 59 | -14.6 | - | 686253846 | hypothetical protein | S. aureus | 2E-13 |
| 5 | 4137 | 4763 | 209 | 151.6 | + | 612729079 | hypothetical protein V053_02705 | S. aureus | 9E-145 |
| 6 | 5127 | 5627 | 167 | 99.9 | + | 810978821 | truncated transposase | S. aureus | 2E-94 |
| 7 | 5777 | 6046 | 90 | 52.5 | + | 686304418 | hypothetical protein | S. aureus | 8E-29 |
| 8 | 6627 | 6884 | 86 | 58.7 | + | 686253828 | prevent-host-death protein | S. aureus | 1E-53 |
| 9 | 6884 | 7150 | 89 | 53.3 | + | 686253829 | addiction module protein | S. aureus | 2E-53 |
| 10 | 7581 | 8135 | 185 | 115.2 | + | 446985335 | transposon Tn917 resolvase | S. aureus | 5E-130 |
| 11 | 8146 | 8394 | 83 | 48.9 | + | 505192374 | hypothetical protein | S. aureus | 1E-48 |
| 12 | 8640 | 8831 | 64 | 14.2 | + | 727739863 | hypothetical protein | S. aureus | 3E-15 |
| 13 | 9230 | 9913 | 228 | 104.8 | - | 446199789 | hypothetical protein | S. aureus | 7E-152 |
| 14 | 10088 | 10426 | 113 | 71.2 | - | 446945179 | growth inhibitor PemK | S. aureus | 3E-74 |
| 15 | 10423 | 10692 | 90 | 62.5 | - | 447052681 | hypothetical protein | S. aureus | 4E-55 |
| 16 | 10993 | 11586 | 198 | 148.9 | - | 505192382 | transposase | S. aureus | 1E-138 |
| 17 | 11839 | 12363 | 175 | 111.7 | + | 612916268 | hypothetical protein U931_02710 | S. aureus | 4E-122 |
| 18 | 12381 | 12998 | 206 | 116.2 | + | 612916267 | hypothetical protein U931_02709 | S. aureus | 2E-149 |
| 19 | 13302 | 14033 | 244 | 119 | - | 446676583 | hypothetical protein | S. aureus | 3E-166 |
| 20 | 14146 | 14955 | 270 | 158.7 | - | 505192384 | lysophospholipase | S. aureus | 0E+00 |
| 21 | 15050 | 15562 | 171 | 81.3 | - | 686253853 | hypothetical protein | S. aureus | 3E-107 |
| 22 | 15611 | 16183 | 191 | 85.1 | - | 686253852 | hypothetical protein | S. aureus | 1E-125 |
| 23 | 16649 | 17296 | 216 | 197.4 | - | 735685252 | integrase | Listeria monocytogenes | 2E-141 |
| 24 | 17326 | 17457 | 44 | 8.8 | - |  | No Significant Match |  |  |
| 25 | 17499 | 19283 | 595 | 318.7 | + | 670014980 | transposase | S. microti | 0E+00 |
| 26 | 19390 | 19695 | 102 | 51.8 | - | 686387212 | hypothetical protein | S. aureus | 8E-34 |
| 27 | 19701 | 20492 | 264 | 154.9 | - | 686387211 | cobalamin biosynthesis protein CobQ | S. aureus | 3E-152 |
| 28 | 20980 | 21957 | 326 | 194.7 | + | 643339039 | hypothetical protein SCHR_11159 | S. chromogenes | 4E-173 |
| 29 | 22042 | 22380 | 113 | 24.7 | + | 517997224 | hypothetical protein | S. intermedius | 1E-49 |
| 30 | 22410 | 22631 | 74 | 15.9 | + | 517997224 | hypothetical protein | S. intermedius | 1E-29 |
| 31 | 24415 | 24735 | 107 | 47.5 | + | 739723653 | hypothetical protein | S. chromogenes | 1E-27 |
| 32 | 24781 | 24996 | 72 | 2.1 | + | 757461052 | hypothetical protein | S. aureus | 5E-30 |
| 33 | 25016 | 25135 | 40 | 5.4 | + | 739723656 | hypothetical protein | S. chromogenes | 2E-13 |
| 34 | 25197 | 25382 | 62 | 3.9 | - |  | No Significant Match |  |  |
| 35 | 25823 | 26041 | 73 | 8.7 | - |  | No Significant Match |  |  |
| 36 | 26201 | 26710 | 170 | 74.4 | - | 528169738 | hypothetical protein L895_13300 | S. aureus | 3E-40 |
| 37 | 27238 | 27741 | 168 | 129.6 | - | 735685252 | integrase | Listeria monocytogenes | 2E-108 |
| 38 | 28026 | 28271 | 82 | 23.5 | + | 686253845 | hypothetical protein | S. aureus | 1E-39 |
| 39 | 28301 | 28486 | 62 | 59.3 | + | 686151506 | transposase | S. aureus | 2E-34 |
| 40 | 28685 | 28963 | 93 | 79.6 | + | 686253938 | integrase | S. aureus | 4E-60 |
